# RetINaBox: A Hands-On Learning Tool for Experimental Neuroscience

**DOI:** 10.1523/ENEURO.0349-25.2025

**Published:** 2026-01-02

**Authors:** Brune Bettler, Flavia Arias Armas, Erica Cianfarano, Vanessa Bordonaro, Megan Q. Liu, Matthew Loukine, Mingyu Wan, Aude Villemain, Blake A. Richards, Stuart Trenholm

**Affiliations:** ^1^Montreal Neurological Institute, McGill University, Montreal, Quebec H3A 2B4, Canada; ^2^Mila, Montreal, Quebec H2S 3H1, Canada; ^3^School of Computer Science, McGill University, Montreal, Quebec H3A 0E9, Canada; ^4^Learning in Machines and Brains Program, CIFAR, Toronto, Ontario M5G 1M1, Canada

**Keywords:** center-surround, direction selectivity, discovery, education and outreach, learning tool, open-source, orientation selectivity, receptive field, visual neuroscience

## Abstract

An exciting aspect of neuroscience is developing and testing hypotheses via experimentation. However, due to logistical and financial hurdles, the experiment and discovery component of neuroscience is generally lacking in classroom and outreach settings. To address this issue, here we introduce RetINaBox: a low-cost open–source electronic visual system simulator that provides users with a hands-on tool to discover how the visual system builds feature detectors. RetINaBox includes an LED array for generating visual stimuli and photodiodes that act as an array of model photoreceptors. Custom software on a Raspberry Pi computer reads out responses from model photoreceptors and allows users to control the polarity and delay of the signal transfer from model photoreceptors to model retinal ganglion cells. Interactive lesson plans are provided, guiding users to discover different types of visual feature detectors—including ON/OFF, center-surround, orientation-selective, and direction-selective receptive fields—as well as their underlying circuit computations.

## Graphical abstract

**Figure:**
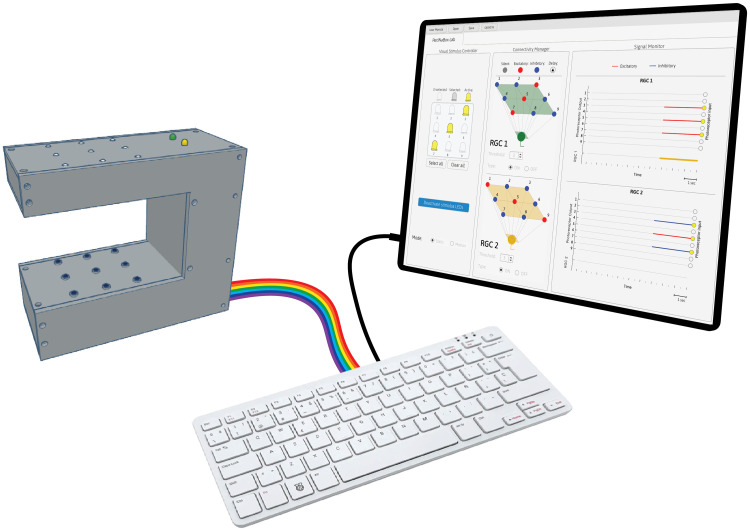
Visual Abstract

## Significance Statement

RetINaBox represents a new conceptual way to teach high-level neuroscience ideas, via the joy of discovery. It provides users with an interactive, hands-on system in which to discover how the brain implements visual feature-selective computations like center-surround, orientation selectivity, and direction selectivity. RetINaBox recreates the experience of being an experimental visual neuroscientist by letting users discover the visual stimuli that activate model neurons and the circuits that enable such feature-selective responses.

## Introduction

The manner in which the brain encodes sensory stimuli varies across brain areas and is often far from predictable (Hubel and Wiesel, 2005; Gefter, 2015; Trenholm and Krishnaswamy, 2020). Thus, to discover how sensory inputs are represented in the brain, we need to record from neurons while providing sensory stimulation. A challenge in neuroscience education and outreach is how to incorporate such experimental work into lesson plans, so that instead of solely learning lists of facts, students get hands-on experience that captures the excitement of discovery. Laboratory classes have long been used to address this challenge, but their scope is often limited by financial, infrastructural, technical, ethical, and training constraints. For example, in vivo single-unit recordings from animal brains during sensory stimulation are widely used to capture the neural code but are too difficult to recapitulate in pedagogical settings.

To this end, we developed RetINaBox as a neuroscience educational/outreach tool that provides users with an interactive, hands-on system with which to discover how the visual system implements several important feature-selective computations that were originally discovered through single-cell neurophysiological recordings. RetINaBox consists of a low-cost computer, simple electronic components (LEDs and photodiodes), 3D-printed parts, and custom-written open–source software. Through several lesson plans, RetINaBox exposes users to numerous computations in the visual system, including ON/OFF processing (Hartline, 1938; Famiglietti and Kolb, 1976; Slaughter and Miller, 1981), center-surround receptive fields (RFs; Barlow, 1953; Kuffler, 1953), orientation selectivity (Hubel and Wiesel, 1959, 1962), and direction selectivity (Barlow et al., 1964; Barlow and Levick, 1965). RetINaBox also includes Discovery Mode, which recreates the experience of being an experimental visual neuroscientist: users load a preset model neuron into RetINaBox but are not shown the wiring scheme of its inputs; users then need to test different visual stimuli until they discover the specific stimulus that activates the neuron; finally, users are tasked with discovering the circuit wiring scheme that underlies the neuron's stimulus selectivity.

## Materials and Methods

The user manual (see Extended Data 1 or GitHub) provides a parts list and guide for building RetINaBox. RetINaBox uses a Raspberry Pi 500 computer, with easy access to GPIO pins for sending and receiving signals. We provide custom-written open–source software for running RetINaBox (see Extended Data 1 or GitHub). At the time of publication, the cost for all components, including a Raspberry Pi and a monitor, was <$350 USD. The build time is ∼4 h.

10.1523/ENEURO.0349-25.2025.d1 Data 1 Download Data 1, ZIP file.

## Results

### Simplified model retina

**Figure 1.**
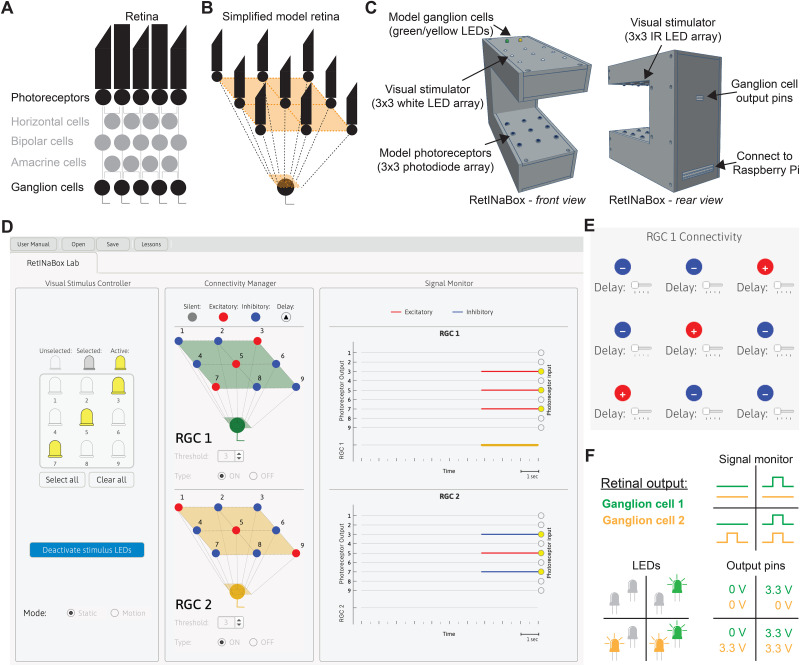
“RetINaBox design and GUI overview.” A schematic of retinal circuitry (***A***) and the simplified model circuitry of RetINaBox (***B***). ***C***, An overview of RetINaBox, including the 3D-printed case, the visual stimulator (3 × 3 LED array), and photoreceptor array (3 × 3 photodiode array), along with the connectivity port between RetINaBox and the Raspberry Pi, and the output signals of the two model RGCs (2 color LEDs on top of RetINaBox and 3.3 V output pins on the back of RetINaBox). ***D***, The RetINaBox GUI, showing the Stimulus Controller, Connectivity Manager, and Signal Monitor. ***E***, The Connectivity Manager pop-up window, which allows users to set the connectivity (silent, +, or −) and delay (none, short, medium, or long) of the connection between each of the nine model photoreceptors and each of the two RGCs. The Connectivity Manager allows users to set the RGC threshold (i.e., how many + inputs an RGC needs to receive to respond) and type (ON or OFF). ***F***, Methods for monitoring RGC activity, either by watching the RGC responses in the GUI's Signal Monitor, or by monitoring the yellow and green LEDs on the top of RetINaBox, or by connecting external electronic devices to the 3.3 V output pins on the back of RetINaBox.

To build RetINaBox, we sacrificed some biological details, since our main goal was to introduce users to general principles without requiring them to first learn about specific implementations of these feature-selective computations in specific cell types of the visual system. As such, and as outlined in more detail below and in Figure 1, *A* and *B*, RetINaBox does not include model horizontal cells, bipolar cells, or amacrine cells. To provide a specific example of the pedagogical philosophy behind RetINaBox: instead of having five separate lessons outlining the exact biological details behind how center-surround is differentially implemented in photoreceptors, horizontal cells, bipolar cells, amacrine cells, and ganglion cells, RetINaBox includes a single lesson focused on the general concept of center-surround.

Seeing starts in photoreceptors. In the vertebrate retina, photoreceptors form a single layer of cell bodies and are spatially organized as a lattice, such that the light-receptive outer segments of neighboring photoreceptors detect changes in light intensity in neighboring regions of visual space (Ahnelt, 1998; Ahnelt and Kolb, 2000). To model photoreceptors, we used photodiodes—semiconductor diodes sensitive to changes in photon flux. To model an array of photoreceptors, we built a 3 × 3 array of photodiodes (Fig. 1*B*,*C*). This is the smallest photoreceptor array that can implement all the feature-selective computations we sought to explore with RetINaBox: ON/OFF, center-surround, orientation selectivity, and direction selectivity.

Next, while the vertebrate retina is a complex tissue comprised of multiple cell types located in specific anatomical layers (Krishnaswamy and Trenholm, 2023; Fig. 1*A*), here we designed a simplified model retina whereby the array of nine model photoreceptors can be connected to two model retinal ganglion cells (RGCs; Fig. 1*B*). Users should note that this is a simplification—in the actual vertebrate retina photoreceptors do not directly connect to RGCs (Fig. 1*A*). The contribution from other retinal neurons to visual computations is incorporated into RetINaBox's connectivity functions that transform signals passing from photoreceptors to RGCs. The photodiodes are powered by the Raspberry Pi, and the output of each photodiode is sampled by a different Raspberry Pi GPIO pin.

### Visual stimulation

So that each photodiode can be independently activated, we aligned the photodiode array with a 3 × 3 LED array (Fig. 1*C*), with each LED being independently controlled by its own Raspberry Pi GPIO pin. To ensure that photodiode activation is not modulated by variations in ambient light levels, we used infrared (IR) stimulation LEDs and IR-sensitive photodiodes. However, so that users can see the pattern and timing of RetINaBox's stimulus LEDs (the 940 nm IR LEDs being invisible to the human eye), we added a second 3 × 3 array of visible (white light) LEDs pointing upward (i.e., away from the photodiodes; Fig. 1*C*).

### Graphical user interface

To control LED stimulation, monitor model photoreceptor responses, connect model photoreceptors to model RGCs, and monitor RGC output responses, we designed a graphical user interface (GUI) to control RetINaBox (Fig. 1*D*,*E*). The GUI has three panels: (1) Visual Stimulus Controller; (2) Connectivity Manager; and (3) Signal Monitor (Fig. 1*D*).

The Visual Stimulus Controller (Fig. 1*D*) allows the user to independently control activation of each LED in the 3 × 3 array. Visual stimuli can be displayed in a static manner or can be made to move either leftward or rightward at three different speeds. Visual stimuli can also be delivered by turning on all stimulus LEDs and then placing shapes in between the stimulation LEDs and photodiodes to deliver specific patterns of light to the photoreceptor array (see the user manual for details).

The Connectivity Manager (Fig. 1*D*,*E*) allows users to connect the output from each model photoreceptor to a model RGC. The GUI features two model RGCs. By clicking on a model RGC (Fig. 1*D*), a pop-up allows users to modify the connectivity of each photoreceptor to that RGC (Fig. 1*E*). Each model RGC can receive input from all nine model photoreceptors. For each connection, the polarity (silent, +, or −, which corresponds to the photoreceptor sending a 0, +1, or −1, respectively, to the RGC) and time delay (none, short, medium, long) can be adjusted. The response threshold for each RGC can be set between 1 and 9, indicating how many + photoreceptor inputs need to be received for the RGC to respond. Additionally, RGCs can be set as either ON or OFF type (Fig. 1*D*), modeling ON/OFF retinal processing. ON RGCs receive signals from photoreceptors that are being stimulated by light. OFF RGCs receive signals from photoreceptors that are not being stimulated by light. RGC output is binary: ON or OFF.

The Signal Monitor (Fig. 1*D*) plots when each model photoreceptor (i.e., photodiode) is activated by the visual stimulus, the polarity of the signal transfer from each photoreceptor to each model RGC, and each model RGC's output response (Fig. 1*D*,*F*). In addition, the output signals of the two model RGCs are indicated by two colored LEDs (RGC1, green; RGC2, yellow) on the top of RetINaBox (Fig. 1*C*,*F*). Furthermore, the output of the RGCs can be used to drive external electronic components via output pins on the rear of RetINaBox that send out 3.3 V signals when a given model RGC is activated (Fig. 1*C*,*F*).

### Lesson 1—explore ON/OFF processing and crack a code with center-surround RFs

Following experiments that described light-evoked spiking in the optic nerve (Adrian and Matthews, 1927), RGCs were found to have spatially localized RFs (i.e., they only “see” within a small part of the visual scene), with some RGCs responding specifically to either increases or decreases in luminance over their RF (Hartline, 1938). Such ON versus OFF responses were subsequently found to arise in bipolar cells, with “sign-conserving” OFF bipolar cells possessing ionotropic glutamate receptors in their dendrites and “sign-inverting” ON bipolar cells possessing metabotropic glutamate receptors in their dendrites (Nelson and Connaughton, 1995; Krishnaswamy and Trenholm, 2023). ON/OFF processing helps with contrast sensitivity and enables robust detection of both increases and decreases in luminance (Schiller et al., 1986).

It was also discovered that RGCs care about the pattern of visual stimuli falling within their RFs, due to a center-surround RF organization (Barlow, 1953; Kuffler, 1953; Trenholm and Krishnaswamy, 2020). For an ON-center RGC, increasing luminance with a spot of light located directly above the cell—usually corresponding to the location of its cell body and most of its dendritic tree—maximally activates the cell. However, if the size of the visual stimulus was increased beyond the RF center, the cell's response decreases due to activation of an inhibitory surround. While first described in RGCs, surround inhibition was subsequently described at each level of retinal processing—in photoreceptors (Baylor et al., 1971), horizontal cells (Kawai, 2022), bipolar cells (Werblin and Dowling, 1969), and amacrine cells (Nelson, 1982)—via inhibitory lateral connections from horizontal cells and amacrine cells. Center-surround RFs mean that RGCs are optimized to respond to local luminance contrast (Trenholm and Krishnaswamy, 2020)—meaning that most visual neurons are not strongly activated by homogeneous, spatially redundant scenes—with the RF size relating to the optimal spatial frequency of luminance contrast that activates a cell.

**Figure 2.**
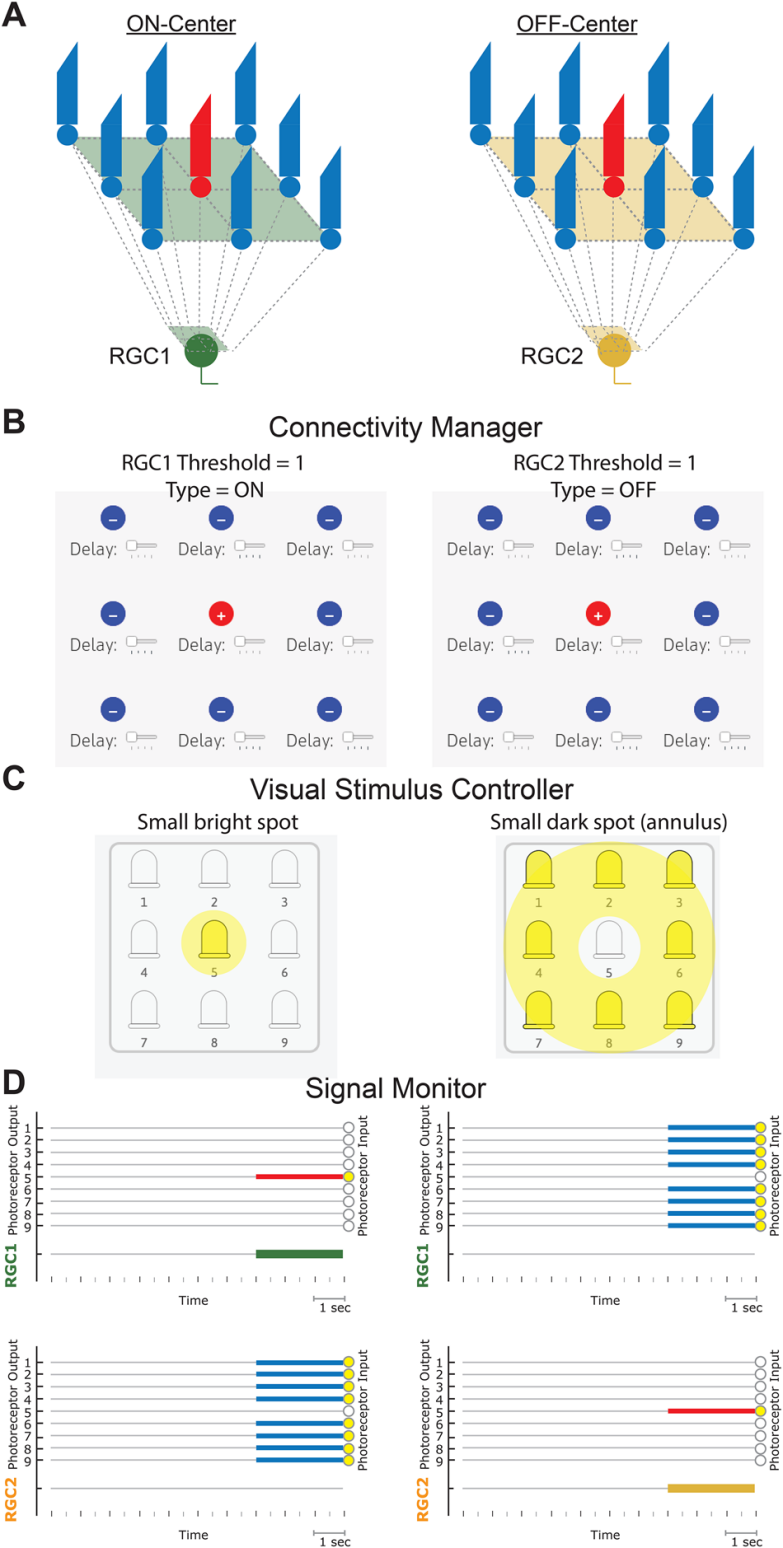
Lesson 1—ON versus OFF processing. ***A***, Example circuits for generating RGCs with center-surround ON (left) and OFF (right) RFs. Example settings for the Connectivity Manager (***B***) and the Visual Stimulus Controller (***C***). ***D***, Example Signal Monitor output for the settings outlined above, with the stimulus LEDs turned on for 3 s (small light spot, left; small dark spot, right).

**Figure 3.**
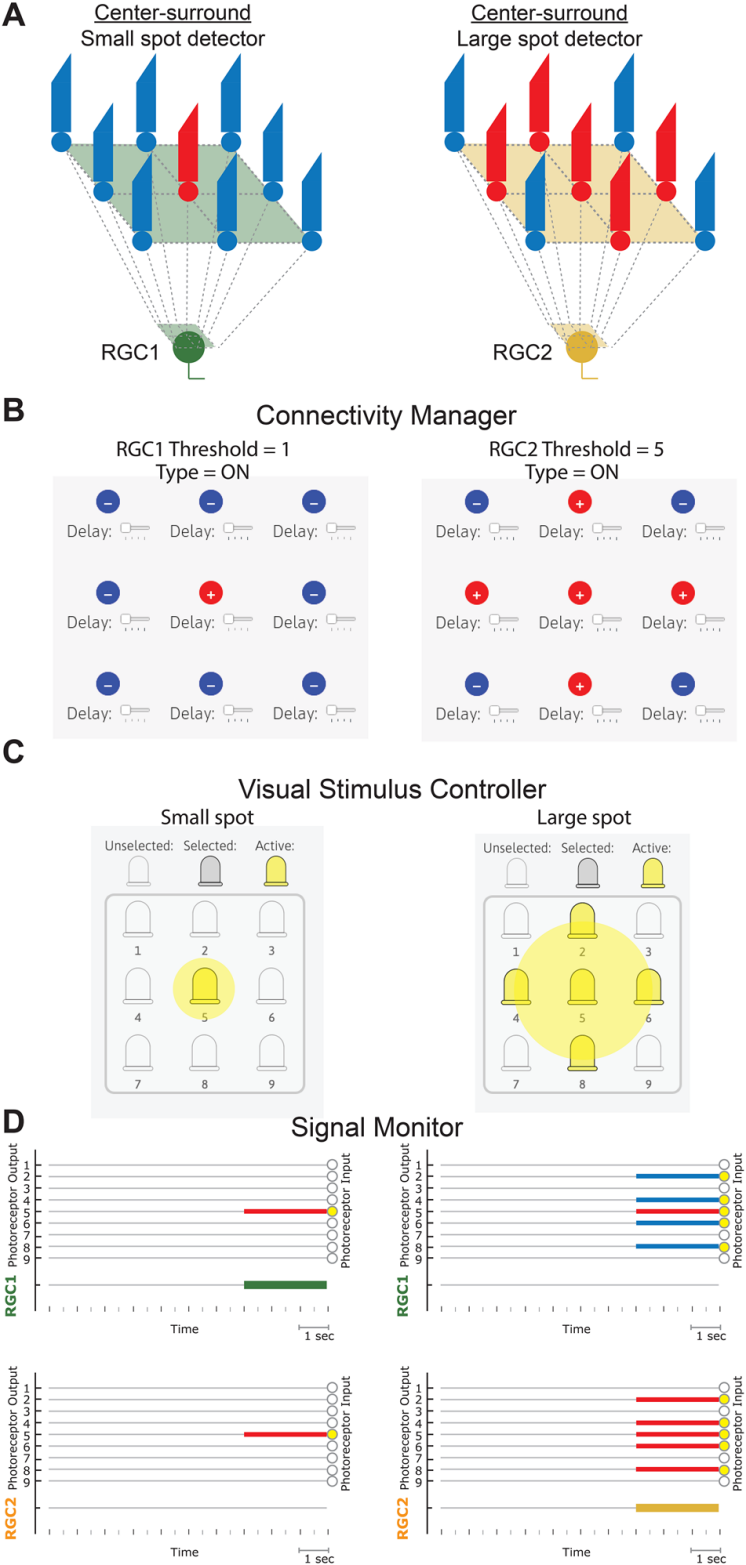
Lesson 1—center-surround RFs. ***A***, Example circuits for ON center-surround RFs with preferences for small (left) and large (right) spots. Example settings for the Connectivity Manager (***B***) and the Visual Stimulus Controller (***C***). ***D***, Example Signal Monitor output for the settings outlined above, with the stimulus LEDs turned on for 3 s (small spot, left; large spot, right).

Lesson 1 of RetINaBox has users explore ON/OFF and center-surround RFs. The first goal is to connect the photoreceptors to RGC1 so that it will respond when a single model photoreceptor is activated, but not when that photoreceptor is activated at the same time as other photoreceptors in the array (Fig. 2*A*–*D*; e.g., generate an ON-center RGC that is selective to a small spot of light centered on the middle of the photoreceptor array). The next goal is to generate a second RGC with the same feature selectivity but located in a different part of the visual field (i.e., users will have two ON-center RGCs with RF centers in different locations). By generating two RGCs with RFs centered in different parts of the visual field, RetINaBox elucidates the point that an individual photoreceptor can contribute to distinct parts of different downstream neurons’ RFs (in this example, the photoreceptor that is responsible for one RGC's RF center contributes to the other RGC's RF surround). The next goal involves switching one of the RGCs from ON-center to OFF-center. This will help users learn about how the visual system differentially processes increases versus decreases in luminance (Fig. 2*A*–*D*). Finally, users are tasked with generating two ON-center RGCs with the same RF center location but with preferences for spots of different sizes (Fig. 3*A*–*D*). This will show users how the RF size controls the spatial frequency of luminance contrast that activates each RGC. For all lessons, if users need assistance solving the activities, they can consult the lesson plan document or check out example Connectivity Manager solutions which can be loaded as presets from the “Lessons” tab in the menu bar. In addition, the GUI allows users to save their solutions and access them later.

**Figure 4.**
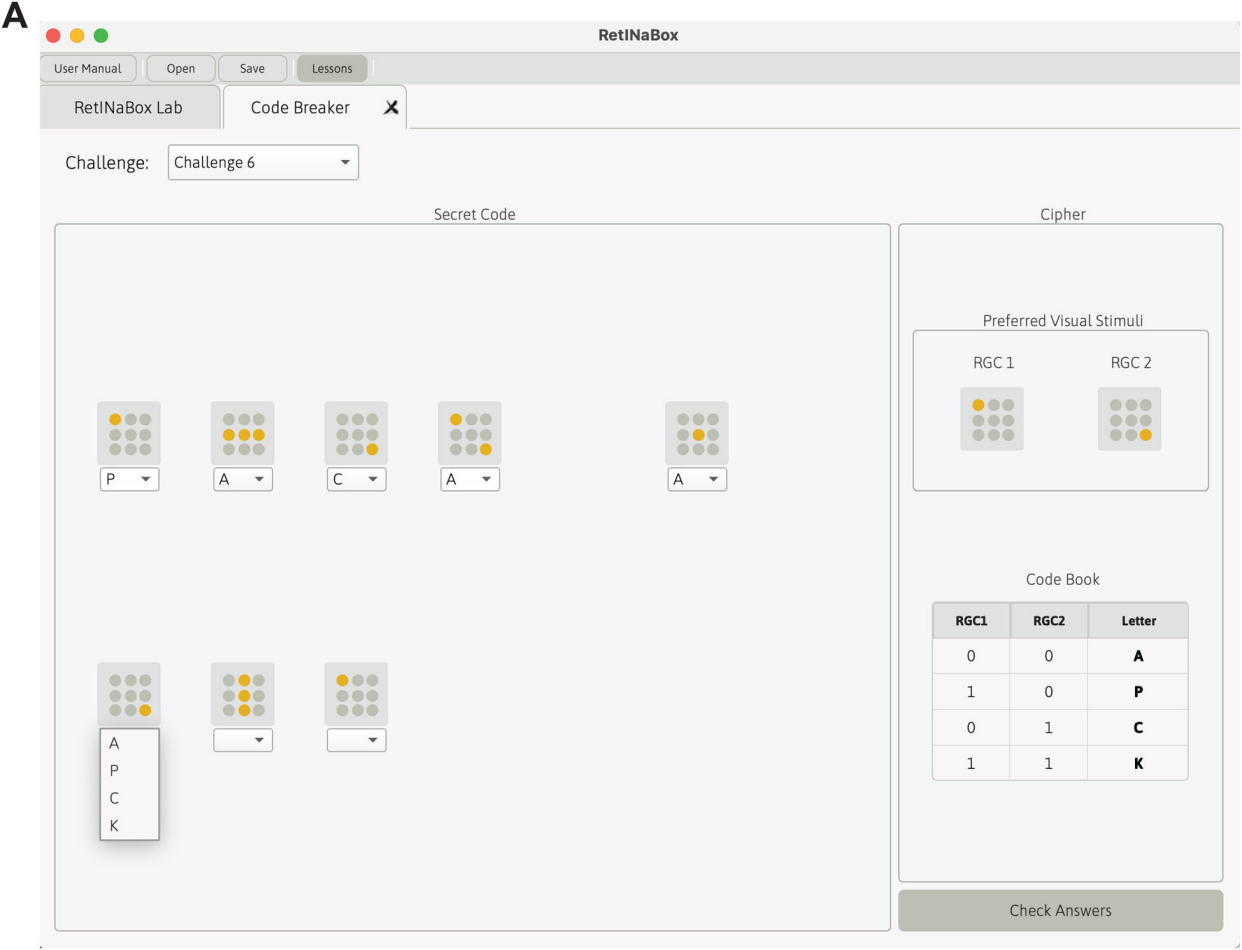
Lesson 1—code breaker activity with center-surround RFs. ***A***, A visualization of the code breaker GUI tab related to Lesson 1. Users must use the cipher (right) to correctly set up the Connectivity Manager for the two RGCs to obtain the indicated feature-selective responses. Next, users place the visual stimuli indicated on the left into RetINaBox and use the cipher instructions (bottom right) to transcribe the RetINaBox output into the correct letter for each visual stimulus in the code.

Lesson 1 ends with a code breaking game, which gets users to apply concepts related to center-surround RFs to solve puzzles. Upon selecting a code breaking challenge, users are provided with a code in the form of a series of visual stimuli, with each visual stimulus representing a letter from the alphabet (Fig. 4). Users are also provided with a cipher containing information they need to solve the problem (Fig. 4). The cipher indicates what the feature preferences should be for the two model RGCs and which letters of the alphabet correspond to neither RGC being activated, one or the other RGC being activated in isolation, or both RGCs being activated together. Once the Connectivity Manager is set according to the cipher, users present RetINaBox with the indicated visual stimuli. Based on the output of the RGCs to each visual stimulus, users enter the corresponding letters into the GUI to solve the code (Fig. 4).

### Lesson 2—build a shape detector with orientation-selective RFs

Despite primary visual cortex (V1) receiving its main sensory input from RGCs—via LGN relay neurons that also tend to possess center-surround RFs (Hubel and Wiesel, 1961; Singer and Creutzfeldt, 1970)—recordings showed that most V1 neurons do not possess center-surround RFs. Instead, many V1 neurons exhibit orientation-selective tuning (Hubel and Wiesel, 1962, 1968), meaning that they are optimally activated by an extended edge (or line) in a specific part of the visual field, aligned in a specific orientation. It was posited that such orientation-selective responses arise when a single V1 neuron pools inputs from multiple center-surround LGN neurons whose RFs are spatially offset along a line (Hubel and Wiesel, 1962; Angelucci and Trenholm, 2024). While early work in cats and primates found that orientation selectivity appears first in V1 (Hubel and Wiesel, 1962, 1968), subsequent work in other species, including rabbits (Levick, 1967) and mice (Baden et al., 2016; Nath and Schwartz, 2016), found that orientation selectivity can also arise at the level of RGCs. Such orientation-selective RFs are thought to be an efficient way to sample visual statistics in the natural world (Olshausen and Field, 1996; Coppola et al., 1998). Orientation-selective signals can then be combined in downstream neurons in various ways, for instance resulting in spatially invariant orientation-selective cells (i.e., a complex cell that receives input from multiple simple cells, with the simple cells all being tuned to the same orientation but exhibiting RFs that are spatially offset from one another; Hubel and Wiesel, 1962) and in cells that respond to combinations of edges/lines, which can in turn serve as building blocks for visual object detectors (Hesse and Tsao, 2020).

**Figure 5.**
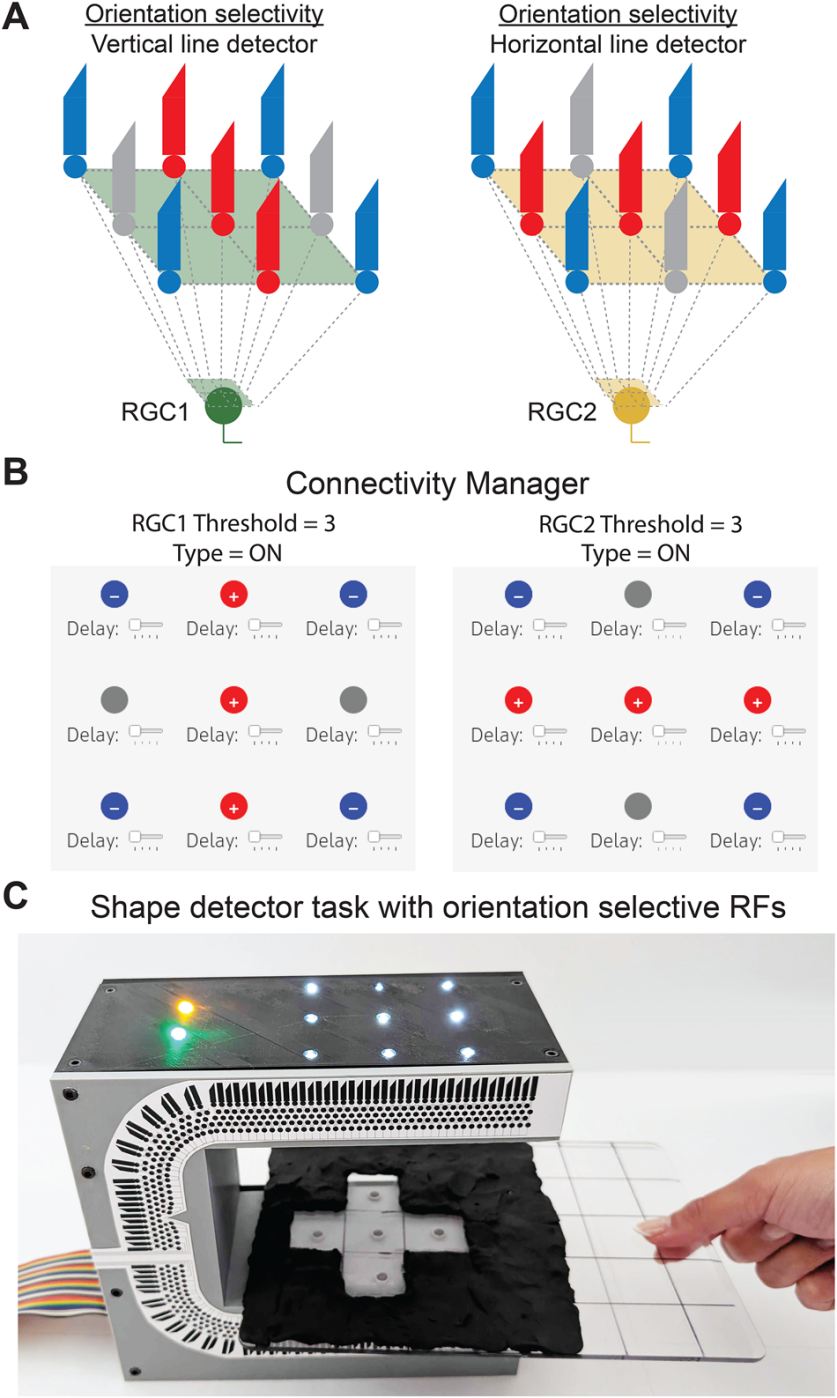
Lesson 2—shape detector activity with orientation-selective RFs. ***A***, Example circuits for generating RGCs with orientation-selective RFs but preferring lines of different orientations. ***B***, Example settings for the Connectivity Manager. ***C***, Demonstration of the shape detector activity: the settings in ***B*** were entered into the Connectivity Manager. A shape corresponding to that created by joining the preferred stimuli of RGC1 and RGC2 was made with the Visual Stimulus Tool, which was then placed in the correct spatial location between RetINaBox's stimulation LEDs and photoreceptor array. Both RGCs are activated at the same time: both yellow and green LEDs are activated.

Lesson 2 of RetINaBox begins by asking users to build an RGC with an orientation-selective RF. The first goal is to connect the photoreceptors to RGC1 in such a way that it will respond when a specific set of three adjacent photoreceptors are activated (i.e., a three-photodiode-long line) but not when any other arrangements of photoreceptors are activated (Fig. 5*A*,*B*). Then, users are asked to generate a second orientation-selective cell with a preference for the same line location as RGC1 but with a preference for a dark line. Next, users are tasked with setting up RGC2 to exhibit a preference for a line of a different orientation than RGC1 (Fig. 5*A*,*B*). The next goal is to generate two RGCs with preferences for lines of the same orientation but of different thicknesses. Then, users are tasked with generating two orientation-selective cells with preferences for a bright line of the same orientation and same spatial location but of different lengths. This helps users learn about the concept of end-stopping (Hubel and Wiesel, 1965), which is thought to be important for enabling encoding of high curvature features in visual scenes (Ponce et al., 2017).

Lesson 2 ends with users being asked to build a shape detector, which gets users to apply concepts related to orientation-selective RFs to solve the problem. First, users need to optimize two ON RGCs to be selective for lines of different orientations at specific locations over the photoreceptor array, with the combination of the two lines representing a shape (Fig. 5*C*). Then, users are instructed in basic electronics to connect the digital outputs of the two RGCs (3.3 V outputs located on the rear of RetINaBox) to generate a buzzer that is activated via an AND gate, meaning that both RGCs need to be activated for the buzzer to sound. This is meant to be analogous to the way that some neuroscientists work when they listen to their experiments in real time by playing their electrophysiological recordings through a speaker.

### Lesson 3—play a video game with direction-selective RFs

Hubel and Wiesel found that some V1 cells, aside from being orientation selective, were also direction selective (Hubel and Wiesel, 1962). Soon afterward, direction-selective RGCs were described in the rabbit retina (Barlow et al., 1964; Barlow and Levick, 1965). Direction-selective RGCs were subsequently found in a variety of species, including mice (Weng et al., 2005; Sun et al., 2006) and primates (Kim et al., 2022; Wang et al., 2023). Direction-selective visual responses have been particularly well studied in the fly visual system (Borst et al., 2020). Direction-selective responses can help to stabilize eye/head movements with respect to the visual scene (Oyster et al., 1972; Yoshida et al., 2001; Yonehara et al., 2016) and help an animal distinguish between external and self-generated movements in the visual scene (Britten, 2008; Sabbah et al., 2017). Direction-selective responses can arise from spatially offset excitation/inhibition and spatially asymmetric delays in inputs along the preferred-null axis (Mauss et al., 2017).

**Figure 6.**
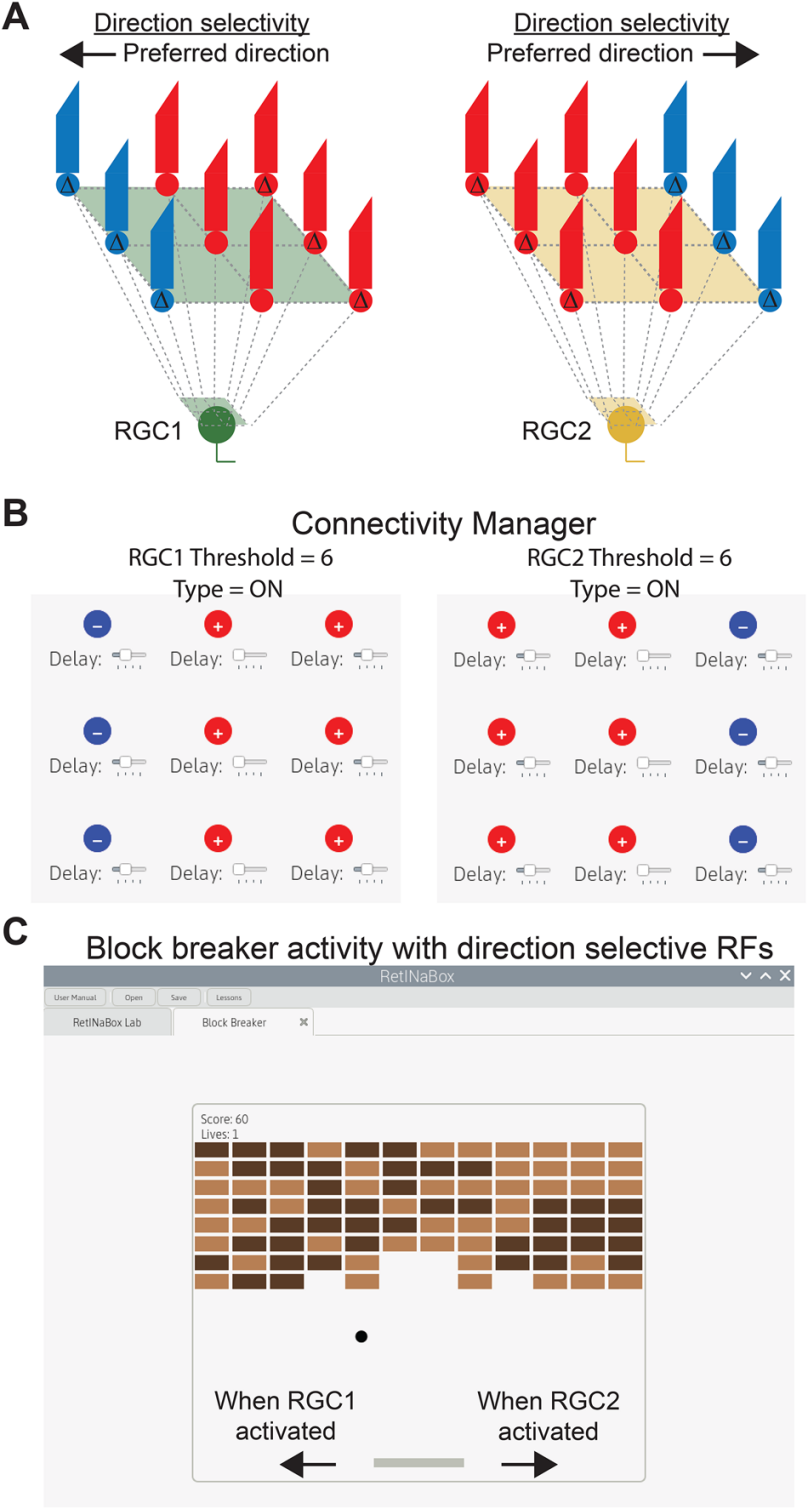
Lesson 3—block breaker video game with direction-selective RFs. ***A***, Example circuits for generating direction-selective RGCs with preferred directions for leftward (left) and rightward (right) motion. ***B***, Example settings for the Connectivity Manager. ***C***, The RetINaBox GUI can be used to load a block breaker video game that takes its left and right input commands from the activity of RGC1 and RGC2, respectively.

Lesson 3 of RetINaBox asks users to build RGCs with direction-selective RFs. The goal is to connect the photoreceptors to an RGC so that it will respond when the user moves a visual stimulus (e.g., the user's hand) in one direction over the photoreceptor array, but not when the same visual stimulus is moved in the opposite direction (Fig. 6*A*,*B*). The next goal is to generate a second RGC with a preference for motion in the opposite direction (Fig. 6*A*,*B*). Next, users are instructed to generate two RGCs, each with a preference for the same visual stimulus moving in the same direction, but at different speeds. This exposes users to the concept of temporal frequency tuning.

Lesson 3 ends with users building a virtual brain–computer interface, which tasks users with generating robust direction-selective detectors and applying the output of RetINaBox’s RGCs to control a video game, via left and right commands (Fig. 6).

### Lesson 4—discovery mode: what does RetINaBox want to see?

**Figure 7.**
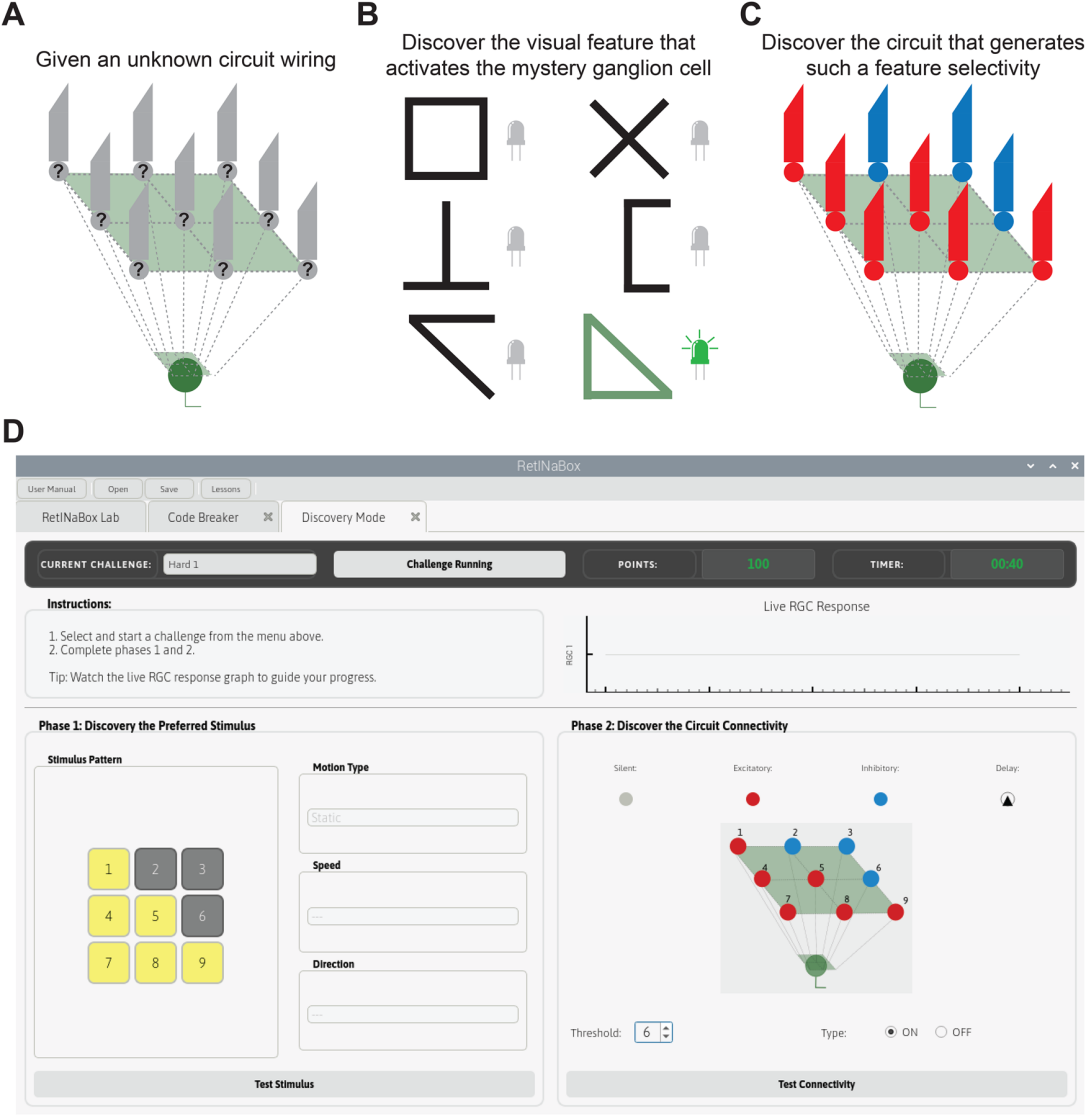
Lesson 4—discovery mode*.* Users select a mystery ganglion cell (***A***), test various visual stimuli to discover the preferred stimulus of the mystery ganglion cell (***B***), and discover the circuit connectivity that generates such a feature selectivity (***C***), all using the Discovery Mode GUI tab (***D***).

To replicate the discovery aspect of scientific exploration where neuroscientists try to figure out what visual stimuli drive visual neurons, Lesson 4 tasks users with discovering the visual features that drive mystery RGCs in RetINaBox and uncovering the circuits that underlie their feature-selective responses (Fig. 7*A*–*C*). Users start by selecting a mystery RGC from a drop-down list in the GUI. In Phase 1, users are tasked with discovering which visual stimulus activates the mystery RGC. Upon identifying the preferred visual stimulus for a mystery RGC, users can verify in the software that they have correctly identified the target visual stimulus (Fig. 7*D*). In Phase 2, users must discover the connectivity settings that enable the mystery RGC to be selective for the target visual stimulus they just discovered (Fig. 7*D*).

## Discussion

Taking inspiration from groups that developed interactive learning tools for neuroscience education (Dragly et al., 2017; Gage and Marzullo, 2022) and that made electronic models of the visual system (Delbrück and Liu, 2004; Li et al., 2010), we developed RetINaBox. However, unlike these previous systems, we tailored RetINaBox to let users learn about several concepts in visual neuroscience—ON/OFF, center-surround, orientation-selective, and direction-selective feature-selective tuning properties—through the act of exploration and discovery. Our goal was to recreate, in a classroom setting, the lab experience of discovering the specific visual stimuli that activate visual neurons.

RetINaBox is an electronic visual stimulation/detection device paired with a computer. RetINaBox comes with four detailed lesson plans in which users are guided to develop and test hypotheses while working toward circuit models for different feature detectors. While going through the lessons and building/testing circuits, users learn important concepts in neuroscience, including excitatory and inhibitory synaptic connectivity, response thresholds, spatiotemporal processing, and parallel processing.

It is important to note that RetINaBox represents a simplification of actual biological circuits (e.g., we bypass bipolar/horizontal/amacrine cells in the retina and replace them with sign, delay and ON/OFF functions). Nonetheless, we believe that RetINaBox provides a useful heuristic for approximating visual neuroscience experiments for educational purposes insomuch as it allows users to focus on general concepts related to feature-selective tuning preferences. These concepts can then be applied toward understanding how such feature-selective computations are implemented in various ways in different parts of the visual system. To ensure that RetINaBox users are not left with an incorrect understanding of how the actual visual system works, the lesson plans include details about the biological circuits that underlie the various visual feature detectors covered in Lessons 1–3.

We designed RetINaBox for neuroscience outreach events, whether it be with high school or undergraduate students, or the general public. Additionally, while someone who is specifically studying circuit processing in the retina or visual cortex may find RetINaBox simplistic, we believe it can be a useful tool for conveying concepts in visual processing to graduate students working in molecular, genetic, and clinical aspects of vision, who often do not have backgrounds in circuits and systems neuroscience. To aid in its use, in addition to lesson plans, we also provide teaching slides (see Extended Data 1 or GitHub), meaning that RetINaBox can be easily incorporated into existing neuroscience classes or quickly set up as an outreach activity.

Seeing as RetINaBox is designed from simple electronic components (photodiodes and LEDs) and a few 3D-printed parts, it can be further expanded or altered by users for their own specific use cases. Along these lines, while RetINaBox uses most of the Raspberry Pi's GPIO ports, several remain unused and could be leveraged for additional purposes as users see fit. Furthermore, while we tried to keep RetINaBox as simple as possible while also providing a versatile system that could be used to explore the topics of ON/OFF, center-surround, orientation-selective, and direction-selective processing, the GUI software is open-source and thus can be edited as users see fit if they wish to add new features. As one example, if a user wanted to include additional details about temporal processing, they could add time constant variables to enable responses to be either sustained or transient. As another example, if a user wanted to add more RGCs to better highlight parallel processing and population codes, this would be possible. As such, while RetINaBox is a powerful learning tool as is, it could be expanded upon in various ways as users see fit.

## Data Availability

Custom code, 3D-print files, the user manual, and the lesson plans can be found here: https://github.com/Trenholm-Lab/RetINaBox.

## Synthesis

Reviewing Editor: Arianna Maffei, Stony Brook University Decisions are customarily a result of the Reviewing Editor and the peer reviewers coming together and discussing their recommendations until a consensus is reached. When revisions are invited, a fact-based synthesis statement explaining their decision and outlining what is needed to prepare a revision will be listed below. The following reviewer(s) agreed to reveal their identity: Richard Born.

There is a agreement among the reviewers that the work is potentially impactful and that the tools presented here can be useful for engaging students in learning about the retina. There are also several important concerns regarding the oversimplification of some aspects of retina function in the tool design. While some simplification of function may be necessary for teaching, the reviewers agree that in the current form the reduced complexity may promote incorrect conceptual understanding of retina function. Specific comments are provided below.

Major concerns

The creators of "RetINaBox" have tackled a very important area in which there is a great need for pedagogical innovation. As they beautifully put it in their Introduction, "A challenge in neuroscience education is how to incorporate such experimental work into lesson plans, so that instead of solely learning lists of facts, students get hands-on experience that captures the excitement of discovery." The introduction raises the appropriate excitement about the tool and the goals of the study. However, the current form of device does not meet the expectation.

When reading the details of the tool the realization was that it may be excessively simplified: 9 photoreceptors hard-wired (essentially) to 2 retinal ganglion cells seems insufficient to provide insight into fundamental aspects of retinal function. While it is clear that the creators have put much hard work and love into their creation and some simplification is necessary and, often, pedagogically useful, there is the concern that students would come away from the lessons with a distorted view of how the retina actually works. Just to take one example, the elegant circuitry of the inner nuclear layer enables every photoreceptor to contribute to both 'on' and 'off' channels (using the ingenious device of sign-conserving vs. sign-inverting glutamate receptors in bipolar cells) and to participate in the 'center' and 'surround' of different retinal ganglion cells. Moreover, because the horizontal cells provide inhibitory feedback to the photoreceptors, the circuitry allows the same neurons to provide opponent spatial surrounds for both 'on' and 'off' channels. The concern here is that students would come away thinking that each photoreceptor has a direct connection to an RGC and that it has a dedicated role-i.e. either center or surround. Much of the beauty of the retina is in the details of the circuitry.

What educational level this tool would be appropriate for? In their closing sentence, the authors assert that it would be useful for students in high school to graduate school. In the current configuration it seems it may being useful for younger students, but graduate students would likely find it overly simplistic.

One important component of retina function that typically raises students' engagement comes from freely available movies showing that a large spot of light fails to elicit much response from the 'on' RGC, which is further deepened when the students see the somewhat puzzling suppression by the annulus of light. This provides a great opportunity to then delve into the RF structure and the underlying circuitry. The typical reaction to the movie of the 'end stopped' cell is even better. The point here is that these movies are freely available, and may do a better job of conveying the excitement of discovery than does RetINaBox.

It would be helpful to note whether the design could be scaled (e.g., by using GPIO expanders or multiplexing) to support more complex receptive field demonstrations. Even if the authors do not intend to pursue this now, acknowledging scalability as a possibility could broaden interest and highlight the flexibility of the platform.

Minor issues.

What is the actual estimated cost and the amount of time necessary to make the device. From looking over the user's manual, it seemed that it would be a fairly time-consuming task. It would be helpful if the authors included a general cost estimate and some idea of how long it might take, say, a high school science teacher to build.

There were also a couple of minor things that raises concerns, such as the statement that center-surround RFs of different sizes were for detecting objects of different sizes. Most vision scientists think of center-surround as a mechanism for reducing redundancy and signaling contrast. Different center sizes are important for conveying information about different spatial frequencies, but "object" is a much more complex and nuanced computation. Another was the question setting up lesson 3: "How do we know if and how objects are moving relative to us?" The kind of simple motion detector being built up with pure excitation and phase delays is a far cry from the circuits that detect relative motion.

## Author Response

Response to reviewers Synthesis Statement for Author (Required):

There is a agreement among the reviewers that the work is potentially impactful and that the tools presented here can be useful for engaging students in learning about the retina. There are also several important concerns regarding the oversimpliﬁcation of some aspects of retina function in the tool design. While some simpliﬁcation of function may be necessary for teaching, the reviewers agree that in the current form the reduced complexity may promote incorrect conceptual understanding of retina function. Speciﬁc comments are provided below.

Major concerns The creators of "RetINaBox" have tackled a very important area in which there is a great need for pedagogical innovation. As they beautifully put it in their Introduction, "A challenge in neuroscience education is how to incorporate such experimental work into lesson plans, so that instead of solely learning lists of facts, students get hands-on experience that captures the excitement of discovery." The introduction raises the appropriate excitement about the tool and the goals of the study. However, the current form of device does not meet the expectation.

We thank the editor and reviewers for acknowledging the importance of the topic that our paper addresses. As you will see outlined below in our detailed responses to speciﬁc comments, we believe that the revised version of RetINaBox now much better 'meets the expectations' set up in the introduction.

When reading the details of the tool the realization was that it may be excessively simpliﬁed:

9 photoreceptors hard-wired (essentially) to 2 retinal ganglion cells seems insuOicient to provide insight into fundamental aspects of retinal function. While it is clear that the creators have put much hard work and love into their creation and some simpliﬁcation is necessary and, often, pedagogically useful, there is the concern that students would come away from the lessons with a distorted view of how the retina actually works. Just to take one example, the elegant circuitry of the inner nuclear layer enables every photoreceptor to contribute to both 'on' and 'oO' channels (using the ingenious device of sign-conserving vs. sign-inverting glutamate receptors in bipolar cells) and to participate in the 'center' and 'surround' of diOerent retinal ganglion cells. Moreover, because the horizontal cells provide inhibitory feedback to the photoreceptors, the circuitry allows the same neurons to provide opponent spatial surrounds for both 'on' and 'oO' channels. The concern here is that students would come away thinking that each photoreceptor has a direct connection to an RGC and that it has a dedicated role-i.e. either center or surround. Much of the beauty of the retina is in the details of the circuitry.

We thank the reviewers for their concern about possible oversimpliﬁcation and their concern students could be misled. Below, we respond to these points in detail.

First, while agree with the statement above about the beauty of the retina and about the importance of inner retinal neurons, we do not agree that the simpliﬁcations we have made impede learning. Instead, we believe the simpliﬁcations we made should aid learning of various concepts in visual neuroscience. To make an analogy: the leaky integrate-and-ﬁre neuron model ignores the beauty of the cell's membrane, ignores the biophysical complexity of ions and ion channels, ignores trying to model the action potential, and ignores many other complexities of actual neurons. Despite all these simpliﬁcations, this model has been incredibly useful to the ﬁeld due to its simplicity.

Second, in the manuscript we now more clearly outline the pedagogical rationale behind RetINaBox, and the speciﬁc reasons for the simpliﬁcations that were made. We are not trying to build a biologically realistic electronic retina with one-for-one electronic components representing speciﬁc retinal cell types. If we were to design a more complex system, it is unclear where we would draw the line between inclusion and exclusion. For example, the reviewers suggest a version where we include ON and OFF bipolar cells and horizontal cells. But then we would need to include amacrine cells for completeness. And it's known that there are multiple types of ON and OFF bipolar cells, amacrine, and ganglion cells, so it's not clear where we would draw the line on how many subtypes of each cell type to include. And the density of many retinal cell types can vary laterally across the retina, so it's unclear how we would incorporate that into a model. Lastly, these myriad cell types form complex cell- type speciﬁc circuits and including all of these would be very complicated. Thus, we believe that making RetINaBox much more complex would make it diOicult to design and assemble, and make it much less practical as a teaching tool. With RetINaBox, we sought to develop a simple tool that would allow users to explore and learn several important concepts related to computations in the visual system. We decided to focus on ON/OFF, center-surround, orientation selectivity, and direction selectivity, as these are canonical and well-studied, and are often taught in classes and appear in many textbooks. Given that seeing starts in spatially oOset light detectors (i.e. photoreceptors), we asked what the minimal set of photoreceptors would be to enable a description of the concepts of ON/OFF, center-surround, orientation selectivity and direction selectivity. This led us to a 3 x 3 array of light detectors. We then developed lessons to let users explore these concepts, via trial-and-error experimentation and discovery, so that we could bring something approximating 'doing experiments in the lab' to the classroom setting. We then ended each lesson with an activity that had users apply the feature selective tuning properties they just learned towards a particular goal (e.g. cracking a code, building a shape detector, etc.). We did so in hope of maximally engaging RetINaBox users to facilitate and reinforce the learning experience. We now more clearly outline this framing of RetINaBox in the manuscript.

Third, to address this reviewer concerns about oversimpliﬁcation and possible misunderstandings by students, we have overhauled the lesson plans and teaching slides. We now provide speciﬁc examples of how the feature selective concepts we explore with RetINaBox (ON/OFF, center-surround, orientation selectivity, direction selectivity) are implemented in real biological circuits, to ensure that users get exposed to the actual circuits in addition to our simpliﬁed ones.

Fourth, the reviewers argue that RetINaBox could give the false impression that photoreceptors directly connect to RGCs. We clearly state that this is not the case, and we are transparent about how we have simpliﬁed the circuitry. In the updated manuscript and lesson plans we now more frequently emphasize that photoreceptors do not directly connect to RGCs in the real retina.

Fifth, the reviewers argue that RetINaBox could give the false impression that each photoreceptor has a single speciﬁc ﬁxed role in the RFs of all downstream neurons. Note that RetINaBox contains 2 RGC output neurons, in large part to make it clear to users that it is the details of the connectivity from photoreceptor to RGC that dictate the RGC's feature selectivity, and that the signal from a single photoreceptor can lead to diOerent outcomes in diOerent downstream neurons. Throughout the lesson plans we frequently ask users to have the 2 RGCs compute similar things at the same time, but in diOerent manners (e.g. in the center-surround lesson we have users make 2 center-surround neurons, but with diOerent center and surround locations; in the orientation selectivity lesson, we have users make 2 orientation selective neurons preferring same line but at diOerent orientations; in the direction selectivity lesson we have users make 2 cells with the opposite preference for a moving stimulus). In each of these cases outlined above, RetINaBox requires that users connect the same 9 photoreceptors to both ganglion cells, but with the connectivity proﬁle (i.e. +/-, delay/no-delay) of each photoreceptor diOering between the 2 RGCs. As such, we do not think that users will come away thinking that each photoreceptor can only inform center or surround exclusively. Nonetheless, to ensure that this important point is not overlooked, we now explicitly make this point in the manuscript and the lesson plans. Furthermore, in the lesson plans we now summarize concepts that are explored with each speciﬁc activity.

Sixth, the reviewers point out that RetINaBox failed to capture ON vs. OFF visual processing. To address this gap, we have implemented ON and OFF visual processing into RetINaBox. We have added this functionality to the software by allowing users to toggle RGCs between ON and OFF types within the Connectivity Manager. Accordingly, we have also added a ﬁgure to the manuscript (new Figure 2), and added a section about ON and OFF processing to Lessons 1 and 2. Furthermore, in the Lesson 1 lesson plan and teaching slides we now outline ON and OFF processing, including details about how the real retina implements sign- conserving OFF and sign-inverting ON responses via OFF and ON bipolar cells. Thus, RetINaBox now directly models how "every photoreceptor [can] contribute to both 'on' and 'oO' channels... and to participate in the 'center' and 'surround' of diOerent retinal ganglion cells." What educational level this tool would be appropriate for? In their closing sentence, the authors assert that it would be useful for students in high school to graduate school. In the current conﬁguration it seems it may being useful for younger students, but graduate students would likely ﬁnd it overly simplistic.

We speciﬁcally designed RetINaBox to be used for neuroscience outreach events, whether it be with students (high school or undergrad) or the general public. Regarding graduate students, while someone who is speciﬁcally studying circuit processing in the retina or visual cortex may ﬁnd RetINaBox to be overly simplistic, we have found that it is engaging for graduate students working in molecular, genetic, and clinical aspects of vision, who often do not have backgrounds in circuits and systems neuroscience. We now more clearly make these points in the manuscript.

One important component of retina function that typically raises students' engagement comes from freely available movies showing that a large spot of light fails to elicit much response from the 'on' RGC, which is further deepened when the students see the somewhat puzzling suppression by the annulus of light. This provides a great opportunity to then delve into the RF structure and the underlying circuitry. The typical reaction to the movie of the 'end stopped' cell is even better. The point here is that these movies are freely available, and may do a better job of conveying the excitement of discovery than does RetINaBox.

DiOerent people learn in diOerent ways and we are not arguing that RetINaBox is the only way to engage with students. In fact, our lab is also working on more 'traditional' neuroscience learning tools (e.g. see www.theopenbrain.org). Furthermore, while we agree that videos are also great learning tools, we ﬁnd it diOicult to deﬁnitively state that one teaching method is "better" than another, as it likely depends on the speciﬁc student and the exact topic. We developed RetINaBox as we felt that a new learning tool that was focused on hands-on user- driven exploration and discovery could be a useful instrument to add to the existing repertoire of pedagogic tools, and be particularly engaging in outreach settings. We now more clearly outline this in the discussion.

The reviewers mention end stopped cells. We thank the reviewers for pointing out this gap. We have added an activity on end stopping to the orientation selectivity lesson (lesson 2).

It would be helpful to note whether the design could be scaled (e.g., by using GPIO expanders or multiplexing) to support more complex receptive ﬁeld demonstrations. Even if the authors do not intend to pursue this now, acknowledging scalability as a possibility could broaden interest and highlight the ﬂexibility of the platform.

We now outline that there are a few remaining GPIO ports available if users want to use them for additional purposes. We also now better emphasize how the software is completely open-source, and so if users are interested in expanding or scaling the functionality of RetINaBox, this is possible.

Minor issues.

What is the actual estimated cost and the amount of time necessary to make the device. From looking over the user's manual, it seemed that it would be a fairly time-consuming task. It would be helpful if the authors included a general cost estimate and some idea of how long it might take, say, a high school science teacher to build.

In the methods we now provide the cost (< $350 USD, including a Raspberry Pi and a monitor) and the time it takes to build RetINaBox (~4hrs, once you have the 3D printed parts and electronics components in hand).

There were also a couple of minor things that raises concerns, such as the statement that center-surround RFs of diOerent sizes were for detecting objects of diOerent sizes. Most vision scientists think of center-surround as a mechanism for reducing redundancy and signaling contrast. DiOerent center sizes are important for conveying information about diOerent spatial frequencies, but "object" is a much more complex and nuanced computation. Another was the question setting up lesson 3: "How do we know if and how objects are moving relative to us?" The kind of simple motion detector being built up with pure excitation and phase delays is a far cry from the circuits that detect relative motion.

We thank the reviewers for these notes. Throughout the manuscript, lesson plans, and teaching slides, for each of the 4 concepts that we explore (ON/OFF, center-surround, orientation selectivity, and direction selectivity) we now more clearly provide background info, example biological implementations, and ethological implications.

Speciﬁcally related to these reviewer comments:

First, we have reworded the sections of the lesson plan and teaching slides where we discussed objects, to make sure that what we are saying is both technically correct while also not being too technical for people ﬁrst being exposed to these topics (in short, we removed the word "object" and now speak more generally of visual stimuli).

Second, regarding the role of center-surround receptive ﬁelds, we now more clearly outline that receptive ﬁelds relate to the part of the visual ﬁeld a neuron 'sees', and that center- surround has two main functions: a) making neurons selectively respond to luminance contrast within their RF (meaning most visual neurons do not respond well to homogeneous (spatially redundant) images); b) the size of the center-surround RF dictates the spatial frequency of luminance contrast that a neuron is selective for.

Third, regarding differentiating between different types of motion, we agree with the statement above, but felt it was important to point out different ethological uses for direction selectivity. We have kept the point about differentiating self-generated vs. externally- generated motion since it's an ethological reason for motion detection, but we now more clearly state in the lesson plan that RetINaBox provides only a simple version of motion detection.
