# Supplementary material for: RetINaBox: A Hands-On Learning Tool for Experimental Neuroscience
Source: eNeuro. 2026 Jan 2;13(1):ENEURO.0349-25.2025. doi: 10.1523/ENEURO.0349-25.2025 (PMC12813302; doi:10.1523/ENEURO.0349-25.2025)
Supplement: Data 1 — Download Data 1, ZIP file. [file eneuro-13-ENEURO.0349-25.2025-s003.zip › RetINaBox_LessonPlans.pdf]

RetINaBox

Lesson Plans

Version 1.2

Overview of the visual system: How do we see?

Visual processing starts in the ***retina***, a thin sheet of light-sensitive brain tissue at the back of your eye (yes, the retina is part of your brain).

Inside the retina, neurons called ***photoreceptors*** detect light and turn it into electrical-chemical signals. Through a process called ***phototransduction***, light intensity changes alter the photoreceptor's membrane potential—that is, the voltage, or difference in electric potential, between the cell's inside and outside. This change in membrane potential affects how much of the neurotransmitter ***glutamate*** the photoreceptor releases. Many neurons 'talk' to each other by releasing glutamate, which can be detected by neighbouring neurons.

These photoreceptor-mediated signals are transmitted by helper neurons called ***bipolar cells*** onto ***ganglion cells***. The signals passing from photoreceptor→bipolar cell→ganglion cell are modulated by ***horizontal cells*** and ***amacrine cells*** (**Figure 1**). The result is that retinal ganglion cells respond only when specific visual features are present in the world. For instance, one ganglion cell might only respond when a spot of light of a particular size appears, and another ganglion cell might only respond when a visual stimulus moves from left to right across the visual field. Finally, ganglion cells pass visual signals out of the eye and to the rest of the brain via the ***optic nerve*** (**Figure 1**).

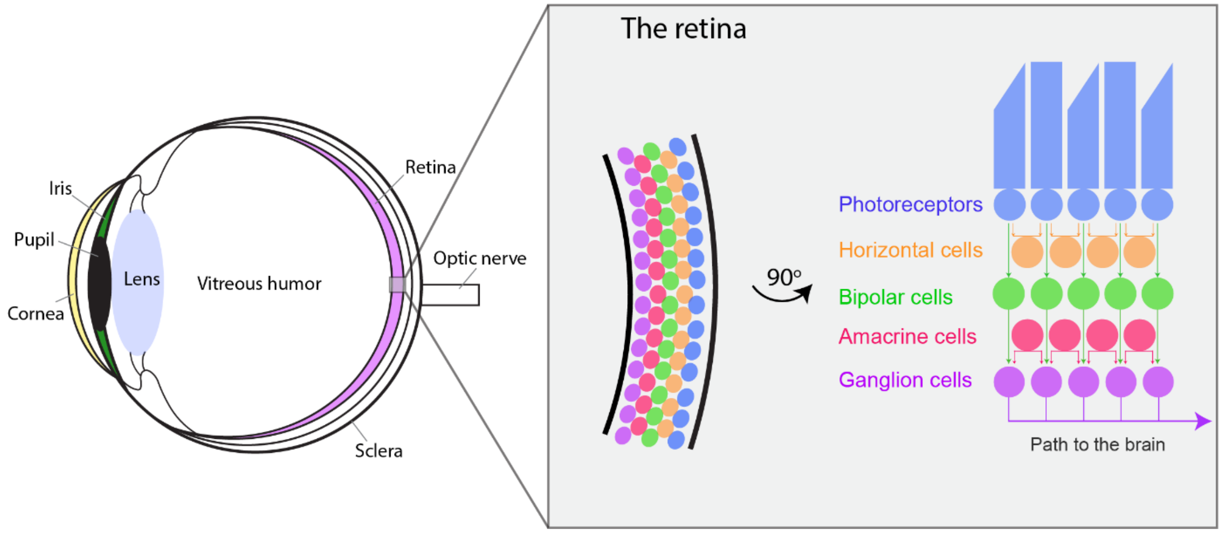

Figure 1. *The eye and retina. Light enters the eye, passing through the cornea, the pupil, the aqueous humor, the lens, and the vitreous humor before falling on the retina. In the retina, light is detected by photoreceptors, which send signals on to ganglion cells, via a network of other retinal cells. Ganglion cells in turn pass visual signals onto higher visual areas in the brain.*

However, retinal anatomy is a bit more complex than outlined above. Photoreceptors (and all the other cell types pictured above) are arranged in a dense, 3D arrangement (**Figure 2**). Within a given retinal layer, cells form a 2D ***mosaic*** (**Figure 2**). For example, in the photoreceptor layer, each individual photoreceptor captures changes in luminance from a tiny portion of the visual scene, but together all the photoreceptors encode the complete image that represents your visual field.

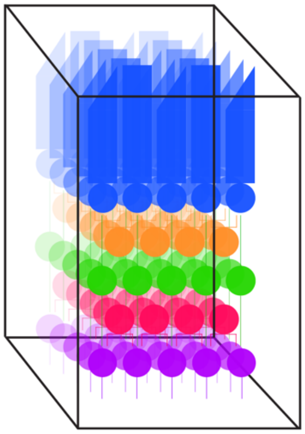

Figure 2. *Diagram of the retina illustrating the mosaic-like arrangement of cells in the retina.*

**For a more detailed overview of the retina, visit theopenbrain.org and https://www.webvision.pitt.edu/.*

RetINaBox: a simplified model of the early visual system

As you've seen, the real retina is a complex tissue, with many cell types intricately connected to form myriad circuits. In contrast to the complex real retina, ***RetINaBox*** is a simplified model of the retina (**Figure 3**) that preserves the key principles of visual processing but provides a hands-on tool that you can use to wire, tweak, and test out visual computations. In other words, it was designed to let you discover how feature selective responses arise in the visual system. RetINaBox contains a 3 x 3 array of model photoreceptors, which directly connect to two model retinal ganglion cells. **Note that real photoreceptors do not directly synapse with ganglion cells.*

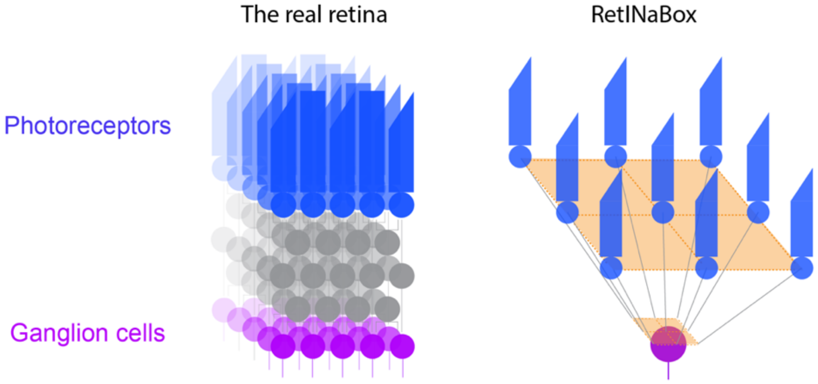

Figure 3. *RetINaBox – a simplified model system for studying feature selective responses in the visual system. Left, In the real retina, light is detected by photoreceptors, and these signals eventually make their way to ganglion cells. Right, For RetINaBox, we modeled the photoreceptor mosaic with a 3 x 3 array of model photoreceptors (photodiodes) and connect these directly to a model ganglion cell. The contributions from other retinal neurons are modeled as positive (+) / negative (-), delay, and ON/OFF functions that can be applied to the signal transfer from each photoreceptor to the ganglion cell.*

RetINaBox has a few key components (**Figure 4**):

- A 3 x 3 array of light-sensitive ***photodiodes*** that act as ***model photoreceptors*** and detect changes in infrared light (from RetINaBox's visual stimulus IR LEDs) and convert these visual signals into electrical signals that they then send to 2 model retinal ganglion cells.
- Two ***model retinal ganglion cells (RGCs)*** that integrate signals from the model photoreceptors.

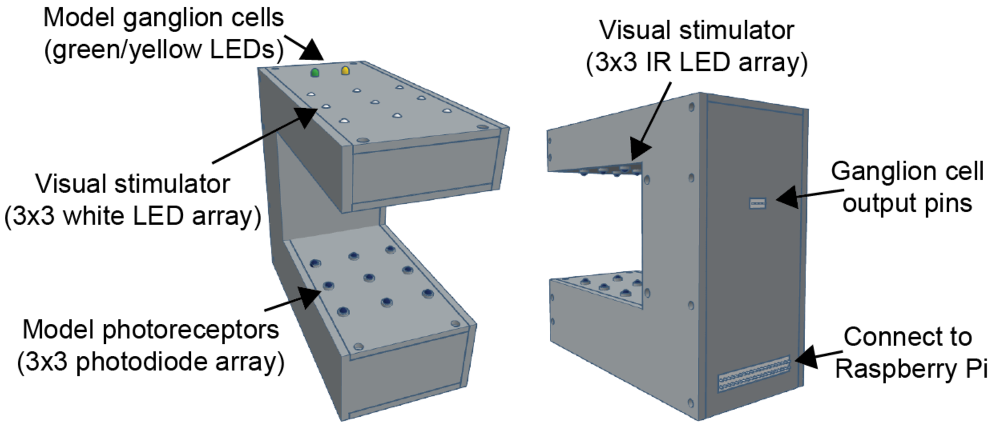

Figure 4. *Annotated diagram of RetINaBox (front view (left), rear view (right)).*

Each ganglion cell responds when it receives positive inputs from a sufficient number of photoreceptors, based on a user-defined threshold. To model the contribution from other retinal neurons (e.g. horizontal cells, bipolar cells and amacrine cells), users can modify the nature of the signal transferred from each photoreceptor to each ganglion cell. Each photoreceptor can connect to either or both ganglion cells. **You get to decide:**

- **Polarity:** whether each photoreceptor activates (excitatory, +1) or silences (inhibitory, -1) the ganglion cell to which it is connected, or whether it remains silent.
- **Time delay:** how long the photoreceptor signal takes to reach the ganglion cell (this can be used to model asymmetric circuit connectivity that can be helpful for implementing motion processing).
- **Threshold:** the number of positive photoreceptor inputs that a ganglion cell needs to receive to respond. If the combined input from all the connected photoreceptors does not sum past the assigned threshold, the ganglion cell does not respond.
- **Type:** whether the RGCs are ON or OFF type cells; ON RGCs receive inputs from photoreceptors (photodiodes) that are activated by light; OFF RGCs receive inputs from photoreceptors that are not activated by light.

To help you perform experiments with RetINaBox, we have designed an easy-to-use ***Graphical User Interface (GUI)***! It has three components (**Figure 5**):

(1) **Visual Stimulus Controller** (see **Figure 5**, left panel; **Figure. 6**): controls the activation of a 3 x 3 LED array, which allows precise control of which model photoreceptors get

activated. You decide which LEDs are activated and sending light to their respective photoreceptors. LED activation arises once you select an LED and then turn the Visual Stimulus Controller on. LEDs can be activated in either '***Static'*** mode (stationary stimuli) or '***Motion'*** mode (stimuli moving left or right at slow, medium, or fast speeds). Use the '***Activate Stimulus LEDs***' button to toggle LED activation.

(2) **Connectivity Manager** (see **Figure 5**, middle panel): allows users to connect each model photoreceptor to one (or both) ganglion cell(s), specifying the signal **polarity** (silent, excitatory (+), or inhibitory (-)) and **delay** (none, short, medium, or long**)**. Users also get to set a **threshold** for the ganglion cell, and its type (either **ON** or **OFF**).

(3) **Signal Monitor** (see **Figure 5**, right panel): displays the input to and output from each photoreceptor, and the output of each ganglion cell (ganglion cell activation is also displayed via the green and yellow LEDs on the top of RetINaBox).

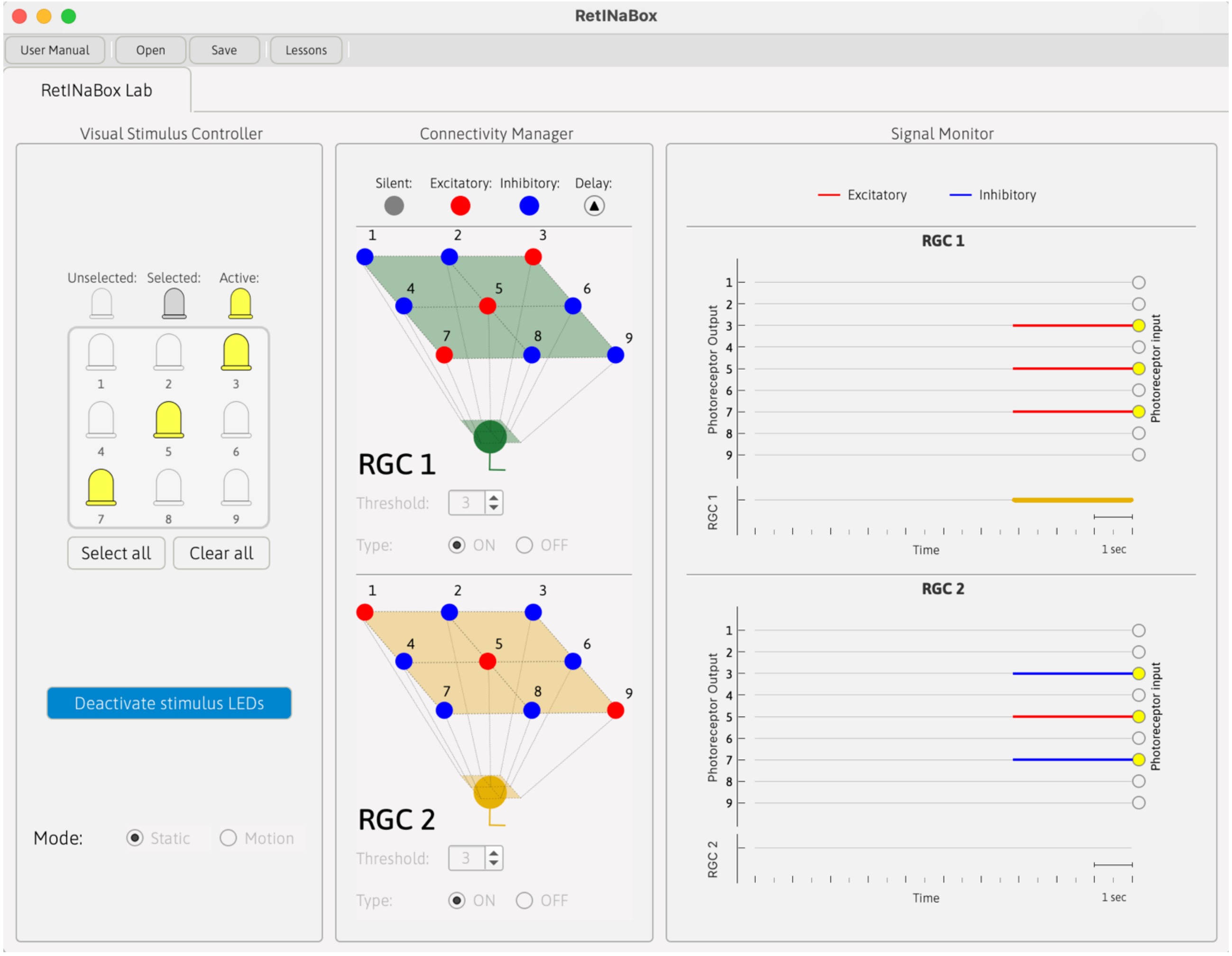

Figure 5. *RetINaBox GUI, which includes the Visual Stimulus Controller (left), Connectivity Manager (middle), and Signal Monitor (right).*

This setup is sufficient to build model ganglion cells with ON/OFF, center-surround, orientation selective, and direction selective receptive fields—just like in the real retina!

Please refer to the* ***User Manual* *if you need further assistance with the software.*

How to test your circuits

Each lesson (outlined below) includes hands-on activities that challenge you to build retinal circuits responsive to different types of visual stimuli. For every circuit you build, you'll have two ways to test its visual selectivity:

(1) **Patterned LED activation** (see **Figure 6**): activate different combinations of LEDs in the **Visual Stimulus Controller** to stimulate the model photoreceptors with different patterns of light. This is a quick way to check that your circuit behaves as expected (i.e. that your ganglion cells respond selectively to specific visual stimuli). Use the 'Activate Stimulus LEDs' / 'Deactivate Stimulus LEDs' button to toggle LED activation.

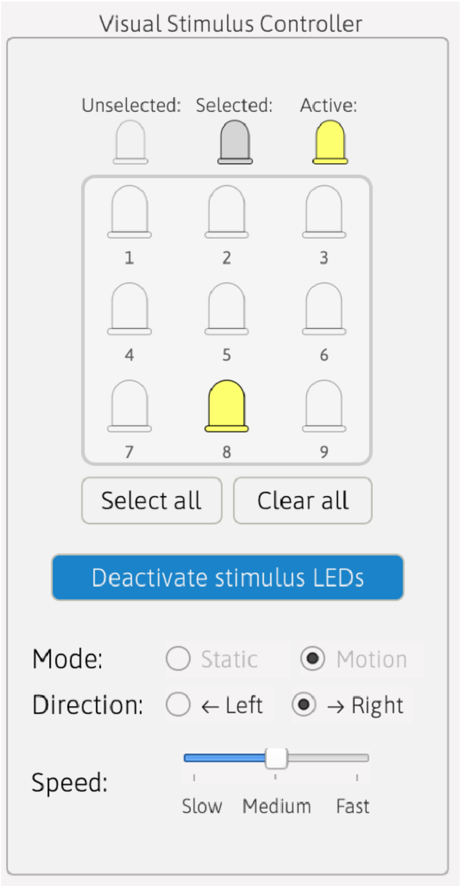

Figure 6. *Visual Stimulus Controller interface for selecting and activating LEDs in the 3 x 3 array to generate custom patterns of light stimulation.*

(2) **Manual visual stimulation** (see **Figure 7**): activate the entire LED array. Then, create patterns with your Visual Stimulus Tool—modeling clay on a clear plastic board. Pass this board between the LED and photoreceptor arrays to selectively block light from reaching certain photoreceptors. This is an effective way to test that your visual circuit responds selectively to specific static visual inputs. Alternatively, if you're dextrous, you can just

use your hands. Or, you can cut out some shapes with pieces of paper/cardboard and use these to control the pattern of light that falls on RetINaBox's photoreceptor array. For moving stimuli, we recommend simply sweeping your hand leftward and rightward across RetINaBox's LED array.

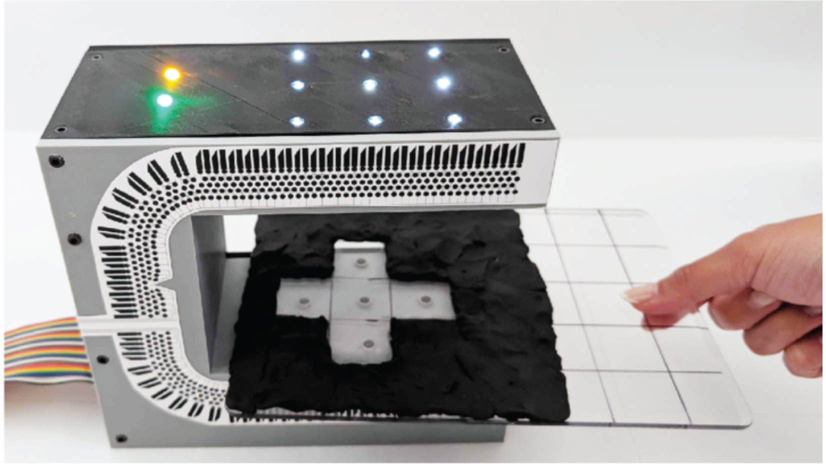

Figure 7. *Placing the Visual Stimulus Tool between the LED array and model photoreceptors selectively blocks light and delivers precise visual patterns to RetINaBox's photoreceptor array.*

Lesson 1: ON/OFF and Center-Surround

The visual world is made up of things in places—different shapes, colours, and patterns located in specific regions of space (**Figure 8**). And it turns out that your visual system has evolved to be particularly sensitive to things that are distinct from large homogeneous backgrounds.

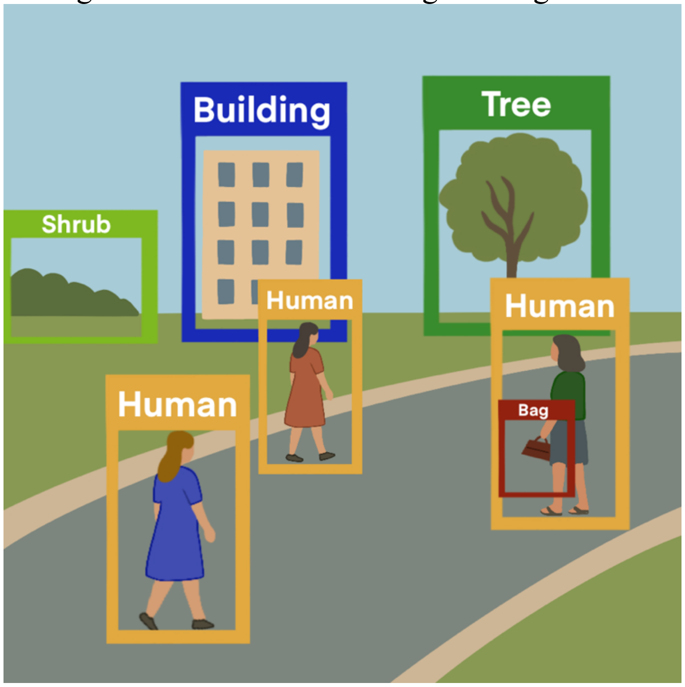

Figure 8. *Your visual system is optimized for parsing things out of visual scenes that differ from the background.*

Receptive fields

A simple way your visual system breaks down the visual world is by endowing each visual neuron with a ***receptive field***. This means that each neuron can only see within a specific region of your visual field (**Figure 9**). You can think of a receptive field like a *spotlight*: each neuron's "spotlight" allows it to only see things in a specific part of the world. Since receptive fields from many different neurons tile your visual field of view, the collection of responses from neurons with different receptive field positions helps your brain figure out where things are in the world.

However, when a neuron is activated, it doesn't just mean that something is in its receptive field. Most neurons also have a preference for what they want to see. In other words, each neuron only responds (or at least only maximally responds) when the specific thing it wants to see happens to fall within its receptive field. Throughout these lesson plans, we're going to spend a lot of time discussing how individual neurons acquire preferences for different types of visual features. However, for the time being, imagine 4 visual neurons. Neurons 1 and 2 have overlapping receptive fields and only see within location A. Neurons 3 and 4 have overlapping receptive fields and only see within location B. Neurons 1 and 3 only respond when apples are in their receptive fields, and Neurons 2 and 4 only respond when pears are in their receptive fields. **Figure 9** outlines this diagrammatically, indicating that a neuron responds only when the thing it wants to see falls within its receptive field.

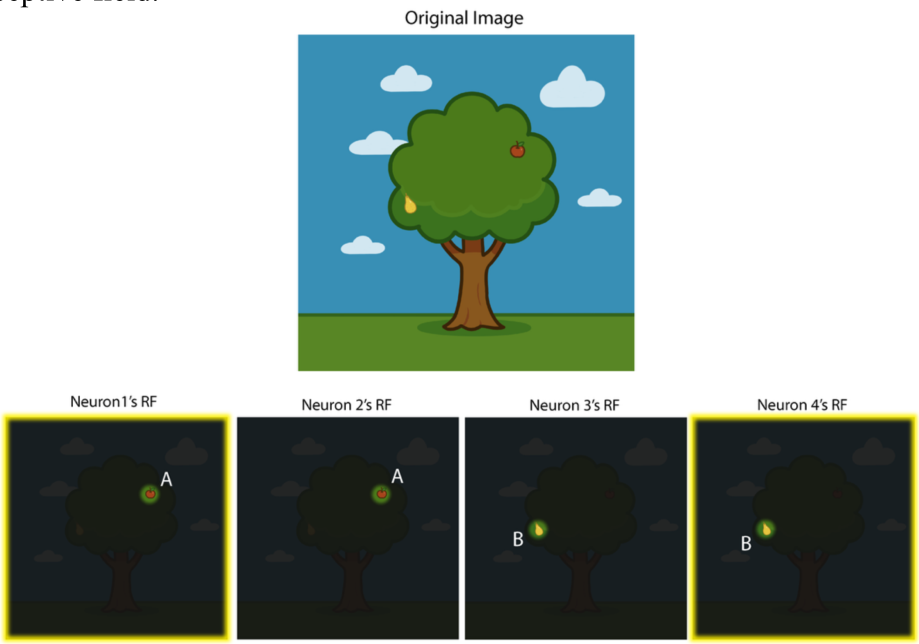

Figure 9. *Different neurons have different receptive fields and therefore only 'see' within small portions of your entire visual field of view. Different neurons can also have preferences for different visual features. In this example, Neuron 1 and Neuron 2 have the same receptive field location, but only Neuron 1 is selective to a circular spot of red light, so Neuron 1 is the only cell to respond to the presence of an apple in its receptive field. However, different neurons can also have preferences for the same visual feature, but different receptive field locations. In this example, Neuron 2 and Neuron 4 have a preference for a circular spot of yellow light, but here only Neuron 4 will fire as the pear is only present in Neuron 4's receptive field. *The yellow bounding boxes indicate that Neurons 1 and 4 are active.*

What visual features best activate visual neurons?

<u>ON/OFF</u>: A quick look across a visual scene—for instance the monochromatic scene shown in **Figure 10**—should make it clear that the luminance (i.e. how light or dark different parts of the visual scene are) often varies significantly from one location to another. And it turns out that your visual system evolved to parse visual scenes in part based on their bright and dim components via ON and OFF receptive fields (**Figure 10**). An ON visual neuron responds when the luminance increases within its receptive field, whereas an OFF neuron responds when the luminance decreases within its receptive field.

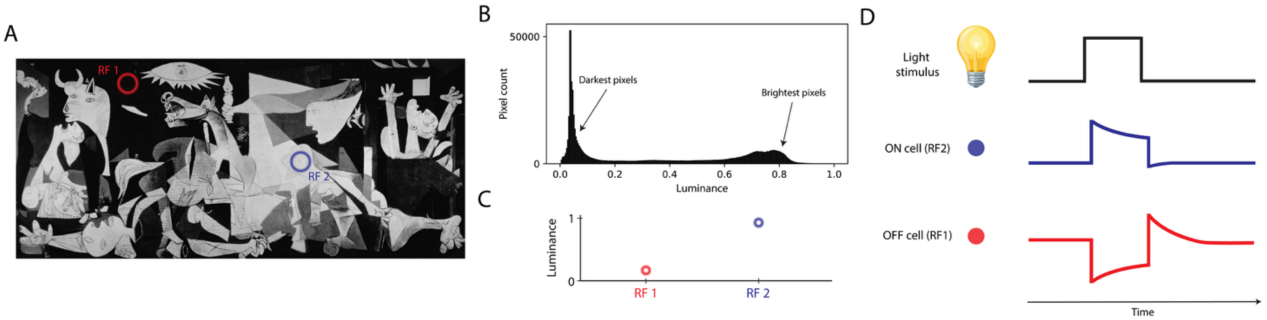

Figure 10. (A) *Pablo Picasso's* Guernica *(1937) with receptive fields of two example neurons highlighted in red and blue.* (B) *Histogram of luminance (brightness) values in* Guernica*, ranging from 0 to 1, showing two peaks corresponding to very bright and very dark pixels in the painting.* (C) *Normalized luminance values within the receptive fields of Neurons 1 and 2.* (D) *Response of Neuron 1 (ON cell) and Neuron 2 (OFF cell) in response to a light stimulus. The ON cell depolarizes in response to the light stimulus, while the OFF cell hyperpolarizes in response to the light stimulus, and depolarizes when it turns off.*

<u>*How do ON vs. OFF responses arise in the visual system?*</u> Photoreceptors are the first cells in the visual pathway that respond to light. In darkness, their resting membrane potential is relatively ***depolarized*** and they continuously release the neurotransmitter ***glutamate***. When luminance within a photoreceptor's receptive field increases and the photoreceptor absorbs photons of light, its membrane potential ***hyperpolarizes***, leading to a decrease in glutamate release (**Figure 11**). In this way, photoreceptors convert light into chemical signals that downstream neurons can interpret.

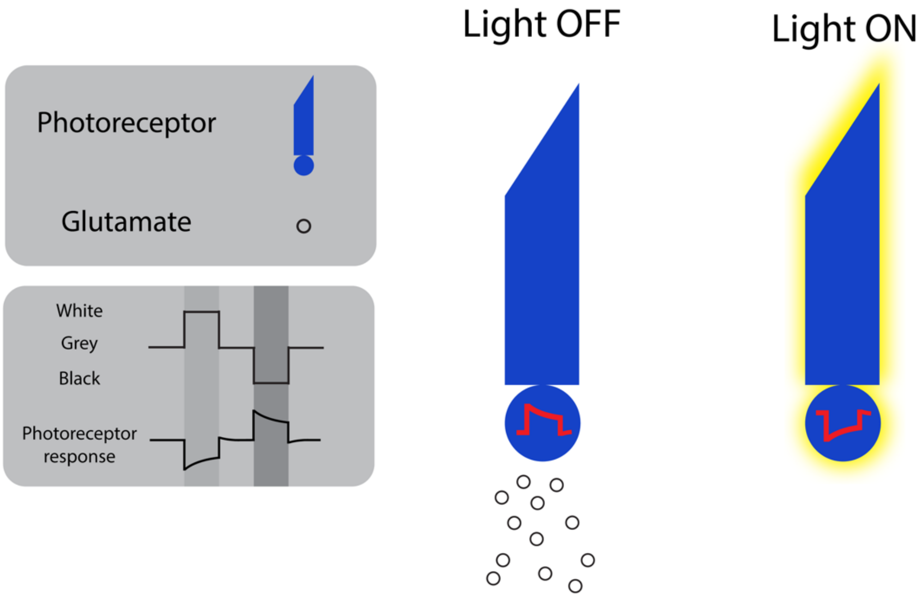

Figure 11. *In darkness, photoreceptors depolarize and release glutamate. When luminance increases, photoreceptors hyperpolarize, and glutamate release decreases.*

A decrease in glutamate release from photoreceptors is the signal that indicates to bipolar cells (BCs) that light is present (horizontal cells also sense glutamate release from photoreceptors and provide feedback, but we won't focus on that point yet). It's at the level of bipolar cells that the visual system starts sorting responses based on whether the luminance in a cell's receptive field increased or decreased. This happens because there are two major classes of bipolar cells: ON and OFF. Both respond to glutamate, but they express different types of glutamate receptors in their dendrites, which allows ON and OFF bipolar cells to differentially respond to the same change in photoreceptor glutamate release that occurs when the light levels change.

ON BCs have metabotropic glutamate receptors in their dendrites. In the dark, glutamate released by photoreceptors binds to these receptors, activating a signaling cascade that closes cation channels, preventing Na^+ ions from entering the cell, and causing ON BCs to hyperpolarize (which means they, in turn, stop releasing glutamate from their axons). In the light, photoreceptors stop releasing glutamate. The ON BC's metabotropic glutamate receptors become unbound, which leads to the opening of cationic channels, which allows Na^+ ions to enter, which depolarizes the cell. This means that ON bipolar cells depolarize when photoreceptors hyperpolarize (and vice versa), and this is termed as 'sign-inverting' (**Figure 12**).

OFF BCs have ionotropic glutamate receptors in their dendrites. In the dark, glutamate released from photoreceptors binds to these receptors, which directly opens the receptor-associated cationic channel, allowing Na^+ ions to enter and depolarize the cell. In the light, the photoreceptors stop releasing glutamate. The OFF BC's ionotropic glutamate receptors no longer bind to glutamate, and the cationic channels close, Na^+ ions no longer enter the cell, and the cell hyperpolarizes. This means that OFF bipolar cells hyperpolarize when photoreceptors hyperpolarize (and vice versa), and this is termed as 'sign-conserving' (**Figure 12**).

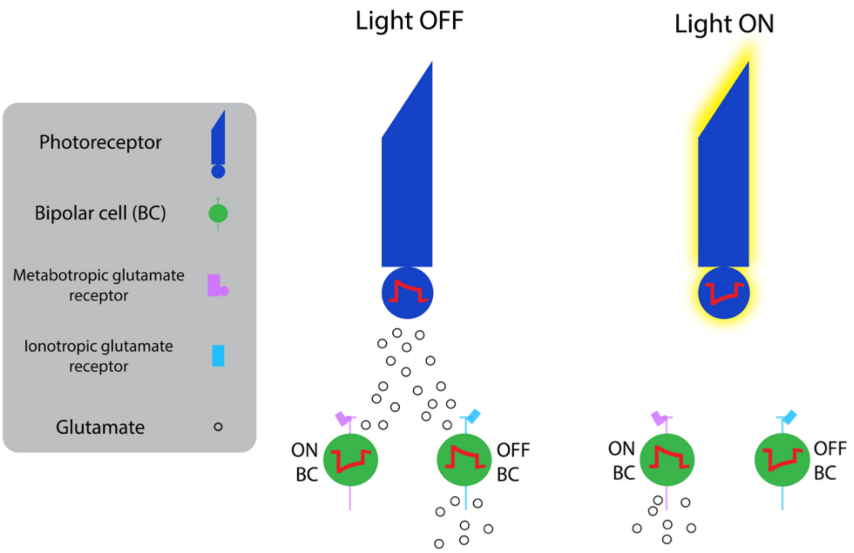

Figure 12. *ON and OFF bipolar cells respond differently to light due to their distinct glutamate receptors. OFF BCs have sign-conserving ionotropic glutamate receptors, and ON BCs have sign-inverting metabotropic glutamate receptors. In darkness, photoreceptors release glutamate, depolarizing OFF BCs and hyperpolarizing ON BCs. When light is present, photoreceptor glutamate release decreases, depolarizing ON cells and hyperpolarizing OFF cells.*

Condition	Photoreceptor	Glutamate Release	ON BC	OFF BC
Dark	Depolarized	High	Hyperpolarized	Depolarized
Light	Hyperpolarized	Low	Depolarized	Hyperpolarized

Table 1. *Summary of photoreceptor and bipolar cell responses to light and dark conditions.*

Center-Surround: In natural scenes, some sections (such as walls, or a clear sky) have little change in luminance over a large spatial area. These are regions of low contrast, with high spatial redundancy. Other parts of the visual scene have tightly packed variations in luminance within a small spatial area. These are regions of high contrast. It turns out that your visual system evolved to largely filter out redundant scenes while encoding local luminance contrast. One way your visual system accomplishes this is with center-surround receptive fields (**Figure 13**).

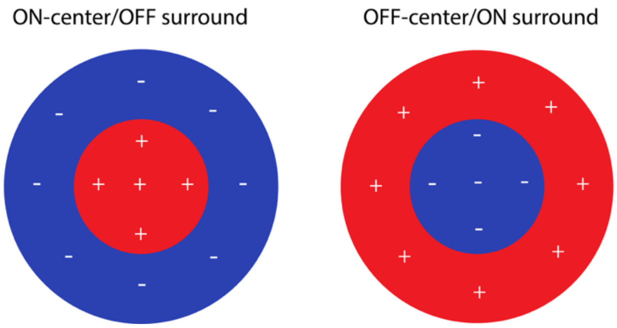

Figure 13. *ON-center/OFF-surround cells are excited by light in the center of their receptive fields but inhibited by light in the surround. OFF-center/ON-surround cells are excited by darkness in their center but inhibited by darkness in their surround.*

With center-surround receptive fields, a cell's receptive field is split into 2 regions: a central area, that when activated excites the neuron; a surrounding area, immediately flanking the center, that when activated inhibits the neuron. Center-surround receptive fields can come in both ON and OFF types. For an ON-center ganglion cell with a low spontaneous baseline firing rate (**Figure 14**), putting a small spot of light in the center that underfills the center will result in a slight increase in firing rate. Increasing the size of the spot of light so that it matches the size of the center but doesn't active the surround will result in the highest firing rate. Further increasing the size of the spot of light will reduce the firing rate, as surround activation will inhibit firing (**Figure 14**).

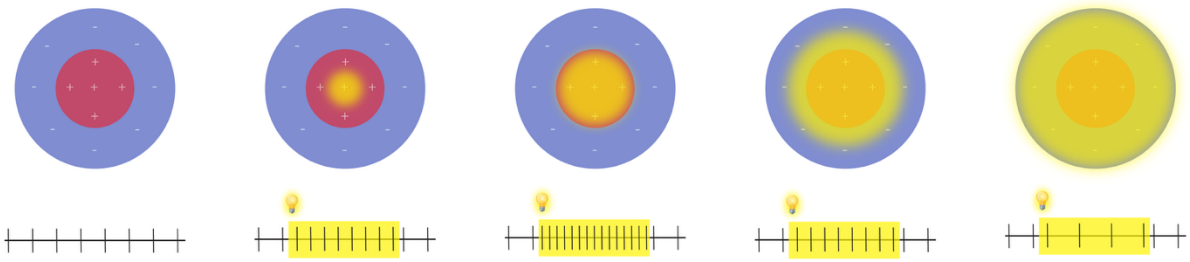

Figure 14. *Response of an ON-center/OFF-surround cell in response to a spot of light of increasing size.*

If we consider a visual scene, the spacing between bright and dim elements can vary widely. In other words, in some places the luminance contrast changes over a very small portion of visual space, whereas in other places the luminance contrast varies over wider portions of visual space. The term we use for the size in the visual field that it takes an element to go from light to dark is referred to as spatial frequency. Natural scenes are composed of many different spatial frequencies, from low to high (**Figure 15**).

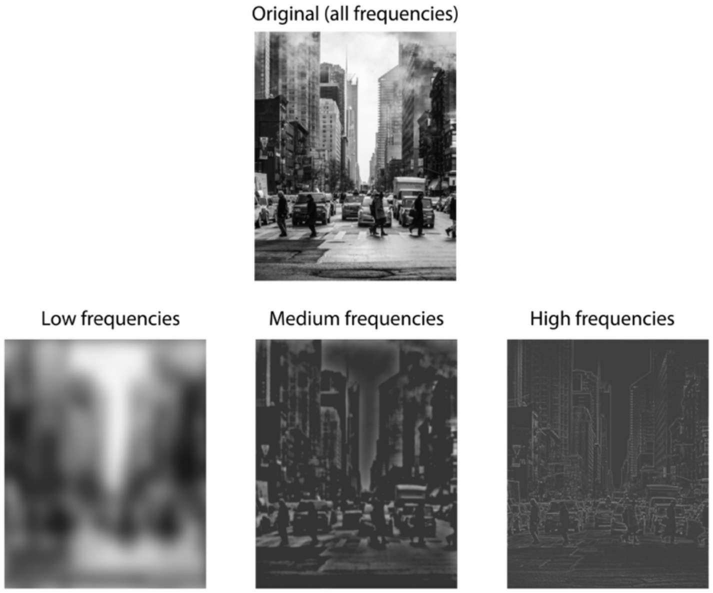

Figure 15. *Visual scenes contain a large variety of spatial frequencies and can be decomposed into different spatial frequency bands. Low spatial frequencies capture coarse features, while higher frequencies contain fine details.*

This is where center-surround receptive fields come in. As described above, center-surround receptive fields help visual neurons selectively respond to local luminance contrast—that is, differences in light intensity between nearby parts of the visual field. Center-surround receptive fields can vary in size, and cells with smaller receptive fields are optimized for detecting fine details (**Figure 16**; right panel; high spatial frequencies), while those with larger receptive fields respond best to broader patterns (**Figure 16**; left panel; low spatial frequencies).

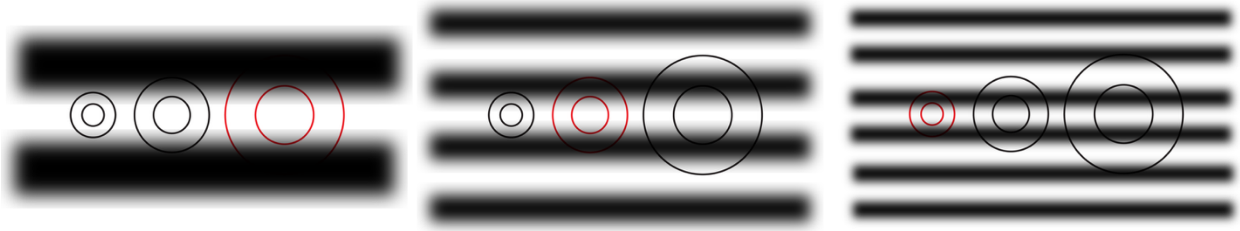

Figure 16. *Examples of 3 ON-center RFs with preferences for different spatial frequencies. Center-surround receptive fields mean that visual neurons tend to respond maximally when the contrast (bright vs. dark) is properly aligned between center and surround. The size of the center-surround receptive field is responsible for the spatial frequency of the luminance contrast that best activates a cell. The cell best activated by a given spatial frequency is shown in red.*

How is center-surround tuning implemented?

Center-surround receptive fields are present to some extent in most retinal neurons. Even photoreceptors have center-surround receptive fields. Photoreceptors connect to bipolar cells, which in turn connect to ganglion cells, which in turn pass visual signals out of the eye to higher visual signals. But photoreceptors also connect to horizontal cells. Horizontal cells are large, laterally projecting neurons. Each horizontal cell receives inputs from many photoreceptors, and horizontal cells are also laterally connected with other horizontal cells. Regarding center-surround, the important feature is that horizontal cells provide negative feedback to photoreceptors. In other words, when a photoreceptor activates a horizontal cell, the horizontal cell feeds back to the photoreceptor, making the photoreceptor less active. But a single photoreceptor only provides a weak input to a horizontal cell, meaning that for a small spot of light that activates a single photoreceptor, the horizontal cell is only weakly activated and provides minimal feedback to the photoreceptor. In contrast, when a visual stimulus is large, it activates many neighbouring photoreceptors, which strongly activate horizontal cells, which now provide strong negative feedback to photoreceptors (**Figure 17**).

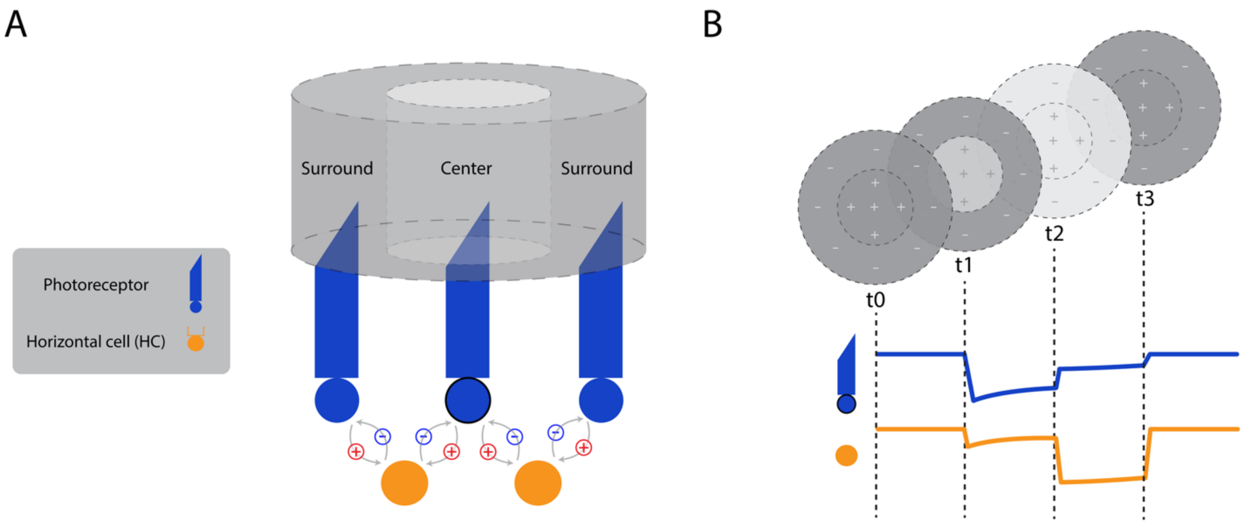

Figure 17. *Horizontal cells (HCs) mediate surround responses in photoreceptors. A: HCs provide negative feedback to photoreceptors, which is maximal during large (surround) visual stimulation. B: Light in the center alone (t1) hyperpolarizes only the central photoreceptor, which weakly hyperpolarizes the downstream HCs. Light in the center and surround (t2) hyperpolarizes central and surround photoreceptors, strongly hyperpolarizing downstream HCs which pool signals from many photoreceptors, which in turn provide strong negative feedback to the photoreceptors, causing them to slightly depolarize, suppressing the overall photoreceptor light response (compared to center illumination alone).*

Exactly how center-surround is implemented varies across different types of retinal neurons. In the retina, center-surround was first described in retinal ganglion cells. However, while (as described above) even photoreceptors exhibit center-surround receptive fields, it appears that for ganglion cells the most significant portion of their receptive field surround arises from amacrine cells, retinal interneurons that can provide inhibition to bipolar and ganglion cells (as well as other amacrine cells). See **Figure 18** for an example of center-surround in retinal ganglion cells.

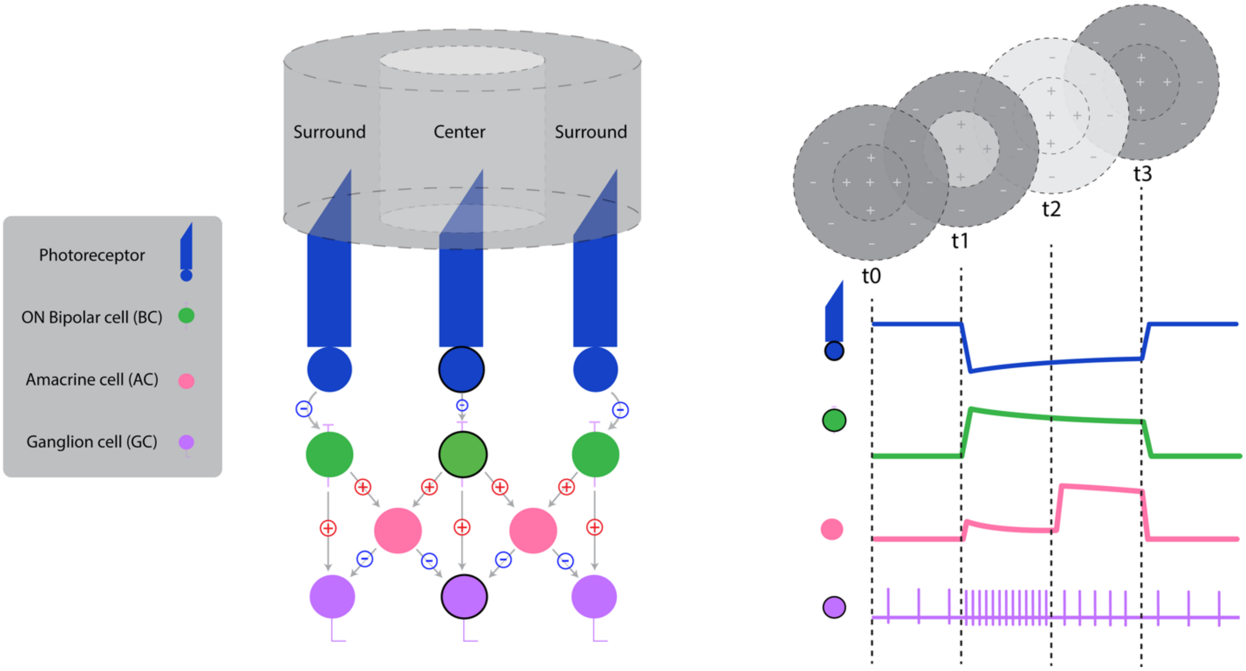

Figure 18. *Amacrine cells (ACs) provide inhibitory input that mediates RGC surround responses. *In this example, we focus specifically on the impact of amacrine cells on ganglion cell surrounds—as such, here we ignore surround responses in photoreceptors, bipolar cells or amacrine cells. A: ACs provide inhibitory input to ganglion cells (GCs) to modulate their response to a surround stimulus. B: Light in the center alone (t1) hyperpolarizes only the center photoreceptor, depolarizing the center ON bipolar cell (BC), which excites both a downstream center ON GC, while also weakly depolarizing some ACs. Weak AC activation has very little effect on the center GC, which fires maximally in response to light in the center. Light in the center+surround (t2) hyperpolarizes center and surround photoreceptors, depolarizing the center and surround ON BCs. The center ON BC provides similar excitation to the center ON RGC in both downstream center and center+surround stimulation conditions, but in the latter condition the surround BCs are also activated, so the amacrine cells which pool from many BCs are much more strongly activated in the center+surround condition. Strong AC activation has a strong suppressive effect on the center GC, reducing its overall spiking output (compared to center illumination alone).*

In summary:

- Each visual neuron responds to light in a specific region of space—its ***receptive field***—helping the brain determine *where* things are in space.
- Separate ON and OFF pathways in the retina enable the system to detect both increases and decreases in luminance, supporting contrast detection.
- ON-center/OFF-surround and OFF-center/ON-surround receptive fields help neurons encode *what* is in their receptive field: neurons respond to patterns with specific spatial frequencies (how quickly light and dark patterns change across space), allowing the visual system to capture both fine and coarse details in the visual scene.

Lesson 1 Objective

Build a simple circuit that mimics a center-surround retinal ganglion cell to explore how the visual system detects a localized spot of light. Then, test your circuit by activating different LEDs (in ‘static’ mode) to check the ganglion cell’s selectivity. Finally, test RetINaBox with real-world visual stimuli to see how robust your center-surround circuit is.

Keep in mind that photoreceptors don’t actually connect directly to ganglion cells, despite the simplified model presented in RetINaBox. Ganglion cells receive input from bipolar cells, and modulatory input from amacrine cells which RetINaBox models with +/- and ON/OFF connectivity functions between photoreceptors and ganglion cells.

Activity #1: build a spot detector with an ON-center/OFF-surround receptive field

Neurons with **ON-center/OFF-surround** receptive fields are *activated* by light in the *center* of their receptive field but *inhibited* by light in the *surrounding* area.

Use RetINaBox’s GUI to build a circuit for RGC1 with an ON-center/OFF-surround ganglion cell (also known as an *ON cell*) that responds only when the center model photoreceptor of the 3 x 3 array is activated by light**,** but not when any of the surrounding model photoreceptors are also activated, and not when a small spot is located above any other model photoreceptor**.**

This activity emphasizes that center-surround neurons (in this case, an ON-center/OFF-surround cell) respond selectively to specific spatial locations and spatial frequencies (in this case a small spot of light located in the center of the photoreceptor array).

Activity #2: build a second spot detector with a different receptive field location

Your next task is to build an ON-ganglion cell (RGC2) with the same size preference as in Activity #1, but whose receptive field is in a **different position** on the photoreceptor array. By moving around the small spot visual stimulus, you should be able to independently activate RGC1 and RGC2 based on the stimulus position.

This activity emphasizes that the same photoreceptors can contribute to distinct parts of different downstream neurons’ receptive fields (in this case, the photoreceptor that contributes to one RGC’s center contributes to the other RGC’s surround), and that by tiling the visual field with spatially offset receptive fields the visual system is able to encode the location of a stimulus via population activity.

Activity #3: build a second spot detector with the opposite polarity

Next, re-wire RGC2 such that it detects a *dark spot* instead of a light spot (i.e., make it an OFF cell). This cell should have the same receptive field center/surround as RGC1 built in *Activity #1*, but with the opposite polarity, meaning that when light activates the center photoreceptor, it now inhibits RGC2 instead of exciting it. More specifically, RGC2 should be activated by light in the

surround and an *absence* of light in the very center of its receptive field (i.e., it should be activated by a small dark spot in the center of the photoreceptor array).

This activity emphasizes that having both ON and OFF responses allows the visual system to detect both increases and decreases in light intensity, helping the brain detect edges and contrast (changes in luminance). It also emphasizes that the same photoreceptors can lead to different receptive field components in ganglion cells that have overlapping receptive fields, dependent on the specific inputs to the ganglion cells.

Activity #4: build two spot detectors with preferences for spots of different sizes

Your next task is to generate **two different** ON ganglion cells (RGC1 and RGC2) with the same receptive field location (center) but tuned to spots of **different sizes.** One ganglion cell should detect a small spot of light, while the other should detect a **larger** spot of light. If you make two spots of different sizes, you should only be able to activate RGC1 with the small spot, and only activate RGC2 with the larger spot.

This activity emphasizes that the size of a neuron's receptive field determines its spatial frequency preference: small fields detect fine details while large fields respond to broader patterns.

If you have difficulty with any of these activities, you can load presets in the RetINaBox: Lessons > Lesson 1, or consult the answer key at the end of this document.

Challenge: Codebreaking with center-surround receptive fields

Now that you've learned how to make ganglion cells that selectively respond to spots of specific sizes in specific locations, we challenge you to apply what you've learned about center-surround receptive fields to **decode a hidden message**.

You will be given a series of visual stimuli, each represented as a 3 × 3 grid of photoreceptors. Some photoreceptors are activated (yellow), while others are not activated (black). Each stimulus corresponds to **one letter** of the secret message.

You will also be provided with a **cipher**, which will help you decode the message. The cipher will tell you the visual feature preferences for RetINaBox's two ganglion cells, RGC1 and RGC2. The cipher will also provide you with a way to decode the activity of RetINaBox ganglion cell activity into four letters (0 means a ganglion cell is inactive; 1 means a ganglion cell is active).

Your task:

1. From the Menu tab in the GUI, navigate to 'Code Breaker' (Lessons > Lesson 1 > Code Breaker).
2. In the Connectivity Manager, wire RetINaBox so that the two ganglion cells respond to the indicated visual stimuli. One important note: *make sure that it is possible for both ganglion cells to be activated simultaneously by a single visual stimulus*!

3. Use the Visual Stimulus tool or some shapes cut out with paper to present visual stimuli from the code to RetINaBox and monitor the responses of the ganglion cells.
4. For each visual stimulus, use the cipher to translate the RGC1+RGC2 output into a letter.
5. Repeat for each stimulus and piece together the full secret message.

See the example below (**Figure 19**):

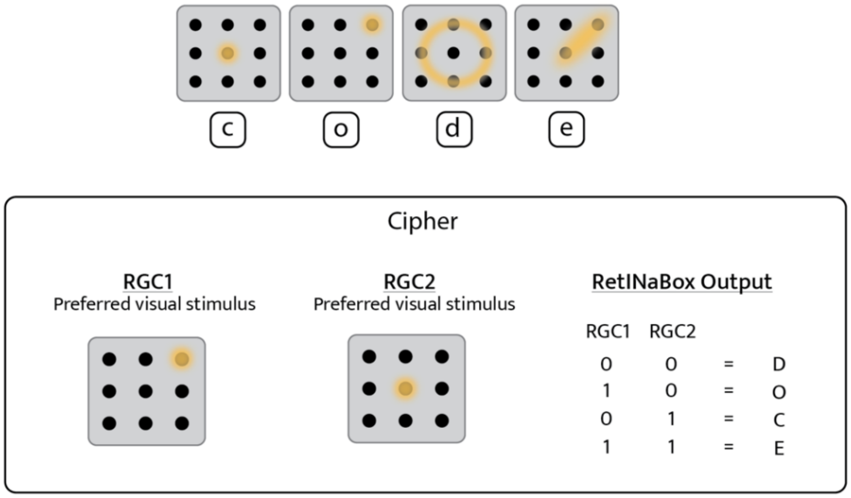

Figure 19. *Example of the code breaking game.*

Challenge #1 of the code breaking activity

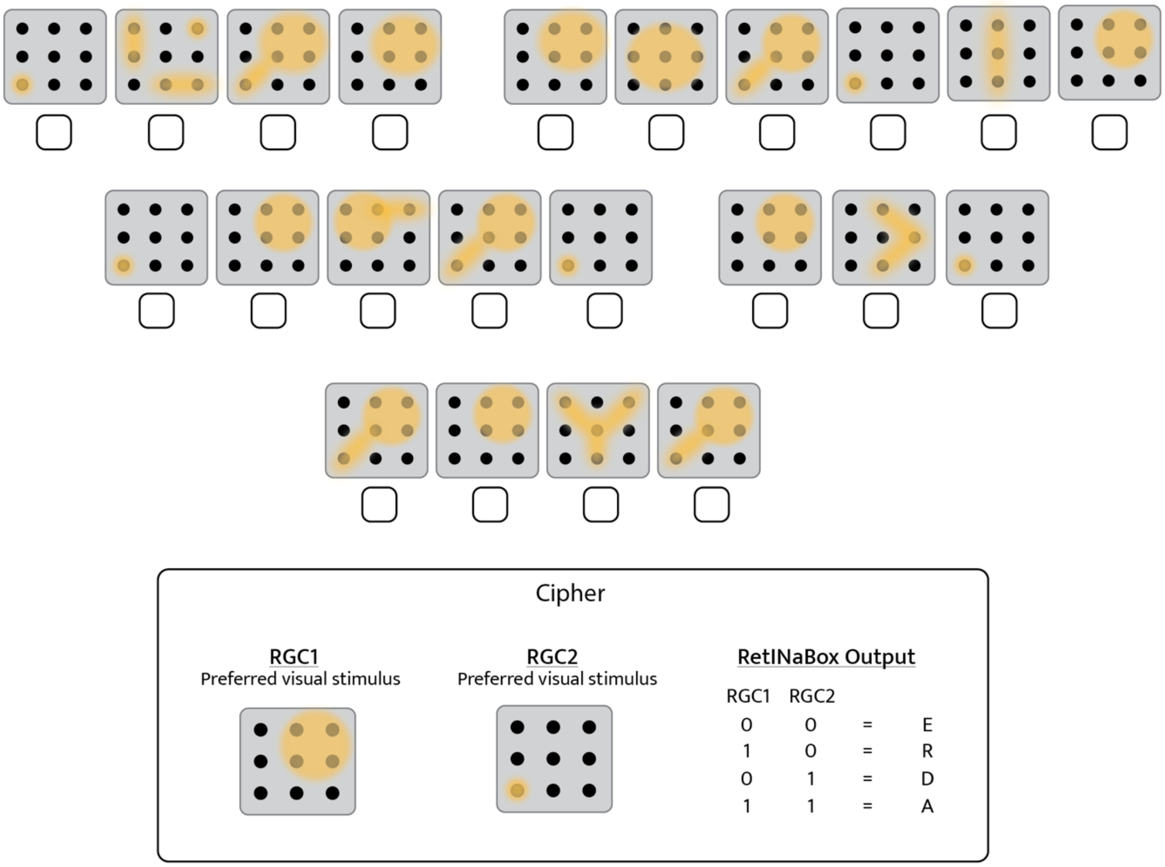

Figure 20. *Challenge 1 for the code breaking game.*

**See the Lesson Plan's Appendix for the solution to this and additional codebreaking challenges.*

Lesson 2: Orientation selectivity

Instead of being a random jumble of patches of different luminance contrast, the visual world contains an abundance of extended lines and edges—extended boundaries along which luminance changes in a consistent manner, such as the outline of a tree or the edge of a building. In fact, most things in the visual world can be decomposed into a combination of lines of different orientations (**Figure 21**).

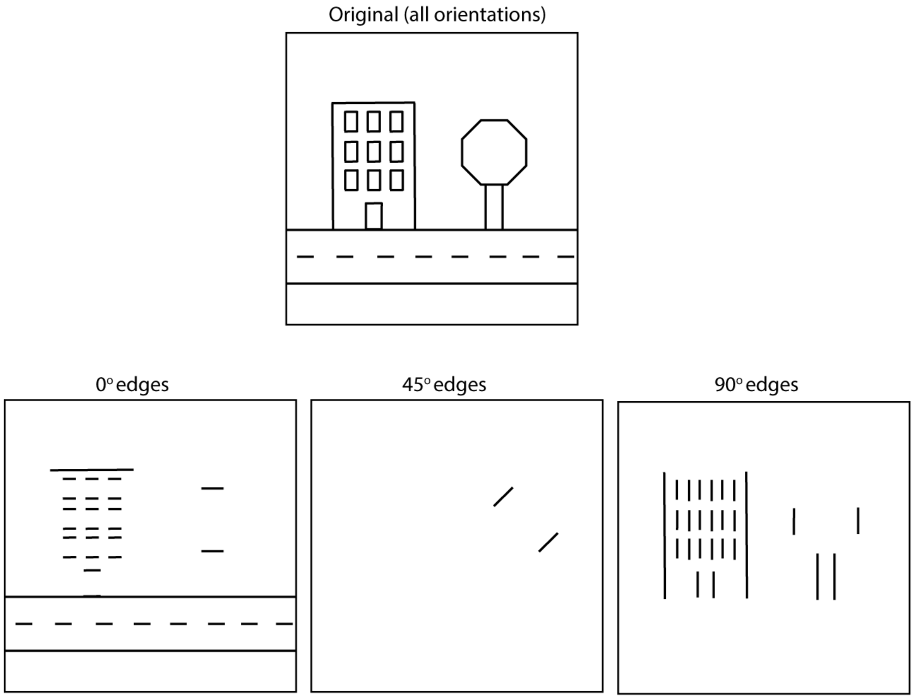

Figure 21. *Natural visual scenes can be broken down into their fundamental building blocks—lines/edges of varying orientations. Each image in the bottom row shows the same scene, but filtered to reveal edges at 0º, 45º, or 90º.*

Because natural scenes contain so many edges of various orientations, the visual system has evolved neurons that respond to lines oriented at specific angles (**Figure 22**). In other words, it decomposes the visual world into its fundamental building blocks: oriented lines of varying orientation.

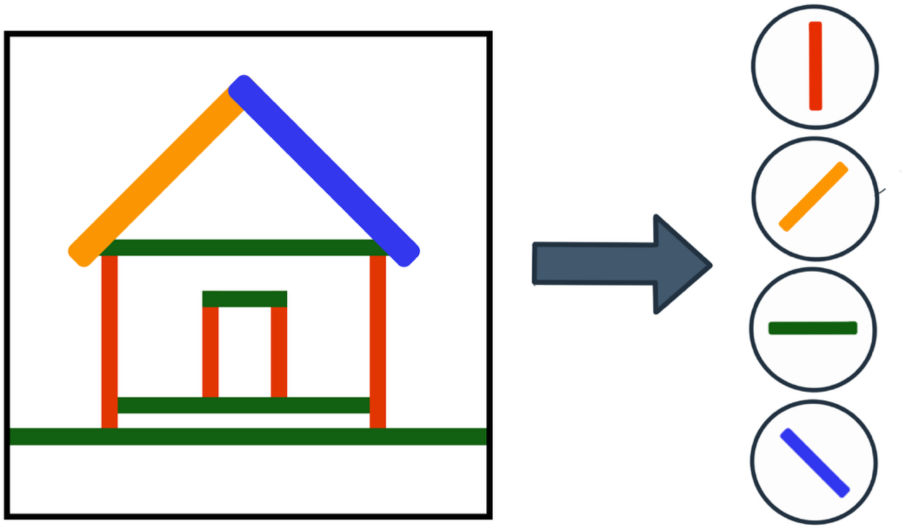

Figure 22. *Some neurons in your visual system are tuned to seeing edges or lines of particular orientations in the visual world. These orientation selective cells (right) decompose the world into series of line elements of different orientations and locations.*

How are orientation selective receptive fields implemented in the visual system?

Signals from retinal ganglion cells (RGCs) are sent through the optic nerve to the lateral geniculate nucleus (LGN) and then relayed to the primary visual cortex (V1). Early recordings from LGN neurons suggested that, much like RGCs, they tend to have center-surround receptive fields. However, the first recordings from primary visual cortex (V1), which receives its dominant visual input from the LGN, found that instead of center-surround receptive fields, most V1 neurons showed orientation selective receptive fields—they respond to extended edges or bars of light at a particular angle.

Visual scientists David Hubel and Torsten Wiesel proposed that this property arises when a single V1 neuron receives input from several LGN neurons whose center-surround receptive fields are spatially offset but aligned along a particular orientation in visual space (**Figure 23** (left)). Together, these inputs form an elongated receptive field that best responds to a bar orientated along the same axis (**Figure 23** (right)).

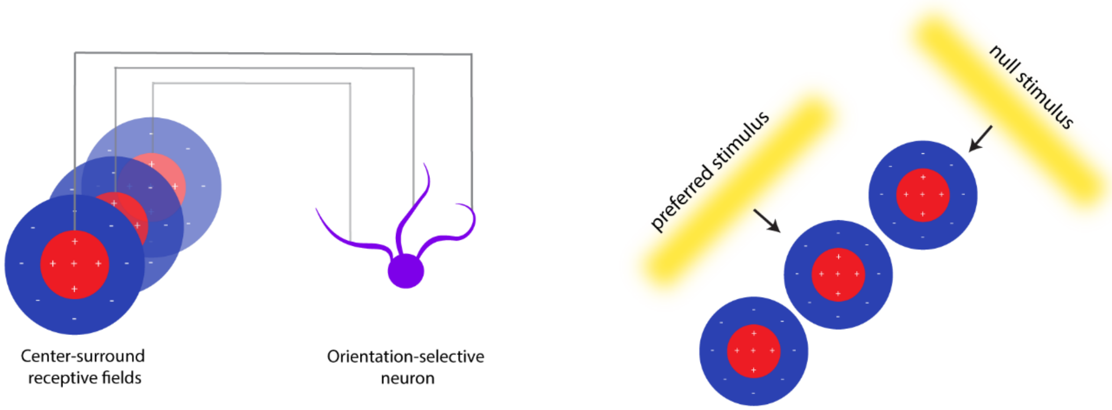

Figure 23. *Left, Hubel and Wiesel's model of orientation selectivity in V1. Several spatially-offset LGN neurons with center-surround receptive fields provide input to a single V1 neuron. The aligned inputs cause the V1 neuron to respond selectively to an elongated bar of light oriented along the same axis. Right, A bar of light aligned with the receptive-field centers of multiple LGN neurons (preferred stimulus) activates all inputs simultaneously, strongly exciting the downstream V1 neuron. In contrast, a bar of light oriented perpendicularly (null stimulus) activates only one center at a time, preventing the LGN inputs from summating and producing no response in the downstream V1 neuron.*

In this way, the brain can build complex feature detectors from simple ones—it combines multiple center-surround inputs to create orientation-selective neurons that detect edges, bars, and contours. Higher-order downstream neurons can then combine inputs from various orientation-selective cells to represent various shapes.

While orientation selectivity can also arise in retinal ganglion cells in some species, via different biological implementations than described above, Hubel and Wiesel's model remains one of the most influential and widely taught examples of how orientation selectivity emerges in the visual system.

In lesson 2, you'll explore how ganglion cells in your model retina can become selective to lines of specific orientations and how you can use these feature detectors to build a shape detector. **Remember, photoreceptors don't connect directly to ganglion cells to generate orientation selective receptive fields, despite the simplified model presented in RetINaBox!* Also, if it helps you conceptually, while we call our tool RetINaBox, here, if you like, you can imagine that the model RGCs are actually model V1 orientation selective neurons.

In summary:

- Our visual system interprets visual scenes by decomposing them into simpler components: lines and edges of different orientations located in different parts of the visual world.
- Cortical V1 neurons integrate inputs from multiple center-surround neurons to become selective for lines of specific orientations, allowing the brain to piece together a rudimentary representation of the visual scene built from shapes and contours.

Lesson 2 Objective

Build and test two retinal ganglion cells to model orientation selectivity and use the combined tuning of these two cells to generate a shape detector. First, test your circuits by activating different combinations of LEDs (in the GUI's 'static' mode). Then, perform real-world visual stimulation by making a shape that is the combination of the two orientated lines that stimulate RGC1 and RGC2.

Activity #1: build an ON-ganglion cell that detects a vertical line

Configure a circuit for RGC1 such that it only responds to a thin *vertical* line of light located in a specific part of the photoreceptor array. The ganglion cell should not respond to a spot of light, nor should it respond to a line of the same length of any other orientation/thickness or located in a different part of the photoreceptor array.

This activity emphasizes that orientation selective neurons respond selectively to bars oriented at specific angles located in a specific part of the visual field.

Activity #2: build an OFF-ganglion cell that detects a vertical line

Configure a circuit for RGC2 such that it has the same receptive field as RGC1 in *Activity #1* (i.e., responds to a thin *vertical* line). However, rather than responding to a bright vertical bar, this ganglion cell should respond to a dark vertical bar—an absence of light in the same region—and should be inhibited by light in this area.

This activity emphasizes that ON and OFF pathways work in parallel to encode increases and decreases in luminance. This organization extends to orientation selective receptive fields, allowing the visual system to detect both bright and dark lines of specific orientations.

Activity #3: build a second ON ganglion cell that detects a diagonal line

Re-wire RGC2 such that it responds to a line of light of the same thickness as Activity #1 but only when the line is in a diagonal orientation. This second ganglion cell should not respond to a line

of any other orientation or thickness, nor to a line of the same orientation centered on a different part of the photoreceptor array.

This activity emphasizes that each photoreceptor can contribute to distinct parts of multiple RGCs' receptive fields and that cells with similar receptive field positions can prefer lines of different orientations.

Activity #4: build two ON ganglion cells that detect vertical lines of different thicknesses

Configure RGC1 and RGC2 such that both cells respond to vertical lines of light, but with one selectively responding to a thin line and the other selectively responding to a thick line.

This activity emphasizes that receptive field size determines a neuron's spatial frequency preference. Cells with smaller receptive fields respond to thin, fine line features, while those with larger receptive fields respond to broader, thicker line patterns.

Activity #5: build two ON ganglion cells that detect vertical lines with different lengths

Configure RGC1 and RGC2 such that both cells respond to vertical lines of light with the same approximate spatial location, but with different lengths.

This activity emphasizes the concept of **end-stopping**, in which an orientation selective neuron's response is inhibited when a visual stimulus of the correct orientation extends beyond a preferred length. In other words, many orientation selective neurons only respond to an oriented line of a specific length. End-stopping is thought to help the visual system encode corners, line endings, and regions of high curvature, which are important for detecting boundaries and shapes.

If you have difficulty with any of these activities, you can load presets in the RetINaBox: Lessons > Lesson 2, or consult the answer key at the end of this document.

Challenge: build a shape detector with orientation selective receptive fields

Your task is to combine the outputs of two orientation selective ganglion cells to detect a specific shape: the shape arising from the combination of lines that activate RGC1 and RGC2. For example, you can build a detector for an X, T, L, or +.

To make this even more exciting, you'll first have to build a buzzer that sounds only when the target shape is present, meaning that the buzzer will only sound when both ganglion cells are activated (this is an example of an AND gate). To build the buzzer circuit, please consult the RetINaBox User Manual. Once you have built the buzzer circuit, connect the outputs of the two ganglion cells (the 3.3V digital output pins on the back of RetINaBox) and one of the grounds, to the buzzer circuit (see **Figure 24**). If you have connected the buzzer circuit correctly, it will only sound when you present your target shape. This is similar to how many neuroscientists perform their experiments—for instance, David Hubel and Torsten Wiesel, who discovered orientation selective tuning in cat visual cortex, often played their electrophysiological recording through a speaker, and listened to the neurons responding as they presented visual stimuli.

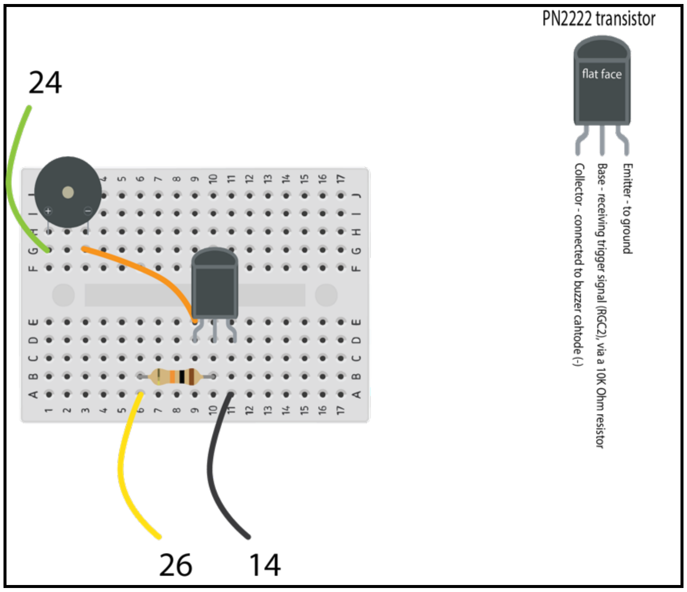

Figure 24. *Circuit wiring of the buzzer. Please see the RetINaBox User Manual for detailed instructions.*

This challenge emphasizes that the visual system builds complex feature detectors from simpler ones. In this activity, students combine two orientation selective receptive fields to create a shape detector, illustrating how downstream neurons can integrate lower-level features to represent complex visual patterns.

Lesson 3: Direction Selectivity

So far, we've seen that some visual neurons have receptive fields, and within these receptive fields some are tuned to specific spatial frequencies of luminance contrast (thanks to ON/OFF and center-surround receptive fields, as outlined in lesson 1) and some are tuned to lines of specific orientations and lengths (thanks to orientation selective receptive fields, as outlined in lesson 2). But one important issue we haven't considered yet is that things in the visual world are constantly moving. The wind blows trees, birds fly across the sky, cars zip by on the street. Additionally, we self-generate a lot of movement in our visual field every time we move our eyes or head, or walk around. To deal with all these movements of our visual scene, it turns out that some visual neurons have evolved to be tuned to the **direction of motion**—they respond best when something moves in a particular direction within the visual field.

Visual neurons with direction selective receptive fields help our brain know if something is approaching, moving away, or moving tangentially to us (see **Figure 25**). This ability is vital for survival—it helps animals track prey, avoid predators, and navigate through the world.

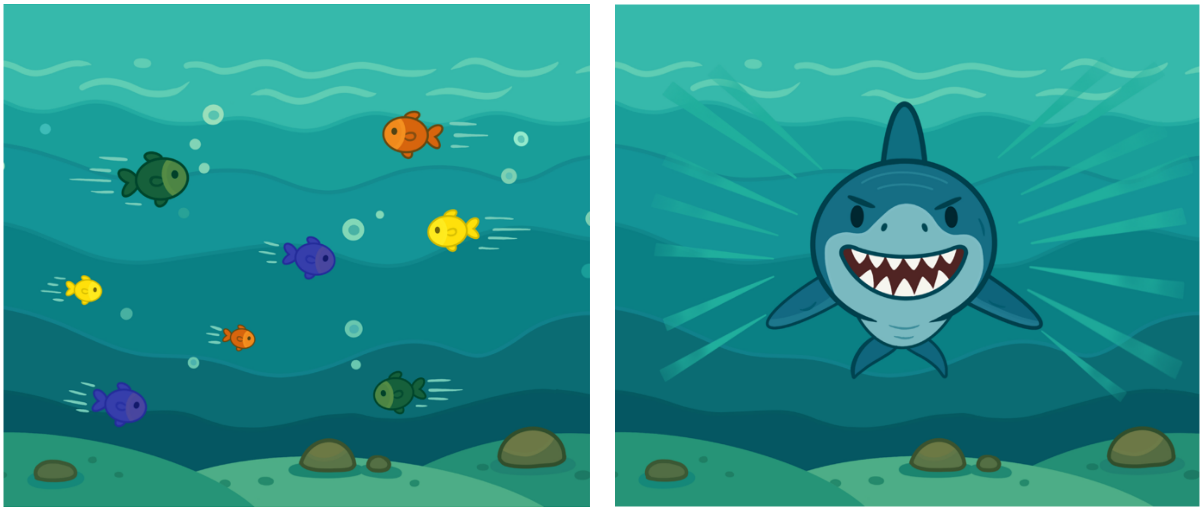

Figure 25. *Detecting motion in the visual world can be important for many tasks. Animals, like fish in the example here, rely on this ability to distinguish potential prey and other fish (e.g., left; small fish swimming in random directions) from predators (right; large looming shark quickly approaching).*

Direction selective responses can also help us differentiate movement in the visual world that we generate (by moving our bodies/heads/eyes) from motion that is external to us (like a bird flying in the sky; **Figure 26**). The visual system needs to be able to distinguish both types of motion to appropriately navigate through our environment and interact with the external world. **However, please note that the direction selective circuits you'll build with RetINaBox are simple motion detectors that won't be able to differentiate between self-generated and external motion.* The direction selective cells you'll make with RetINaBox can only detect the direction that patterns move across its visual field.

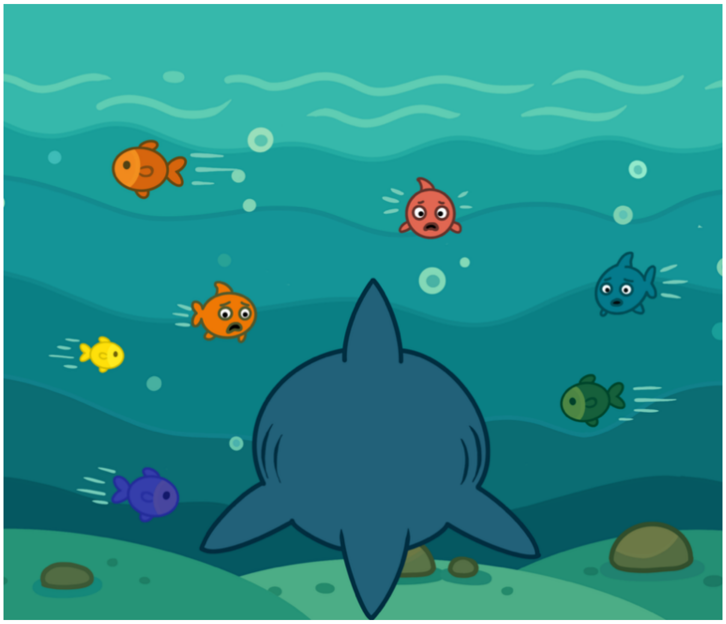

Figure 26. *Distinguishing self-generated motion from external motion is important for many tasks. In this example, a shark approaching its prey must distinguish optic flow generated by its own movement from the movement of the fish it wishes to eat, which are also in motion. To successfully reach its target, the shark must integrate both self-motion and external motion signals.*

To generate preferences for stimuli moving in specific directions, our visual system takes advantage of the fact that a moving stimulus will activate spatially-offset photoreceptors that connect to a single downstream visual neuron in temporal sequence along the trajectory of movement—that is, photoreceptors on the leading edge of the moving stimulus get activated first, while photoreceptors on the trailing edge of the moving stimulus get activated last. This means that, for a downstream neuron receiving signals from multiple spatially adjacent photoreceptors, when a stimulus moves there is a time delay between when the downstream neuron receives signals from photoreceptors on leading versus trailing edges of its receptive field (see **Figure 27**).

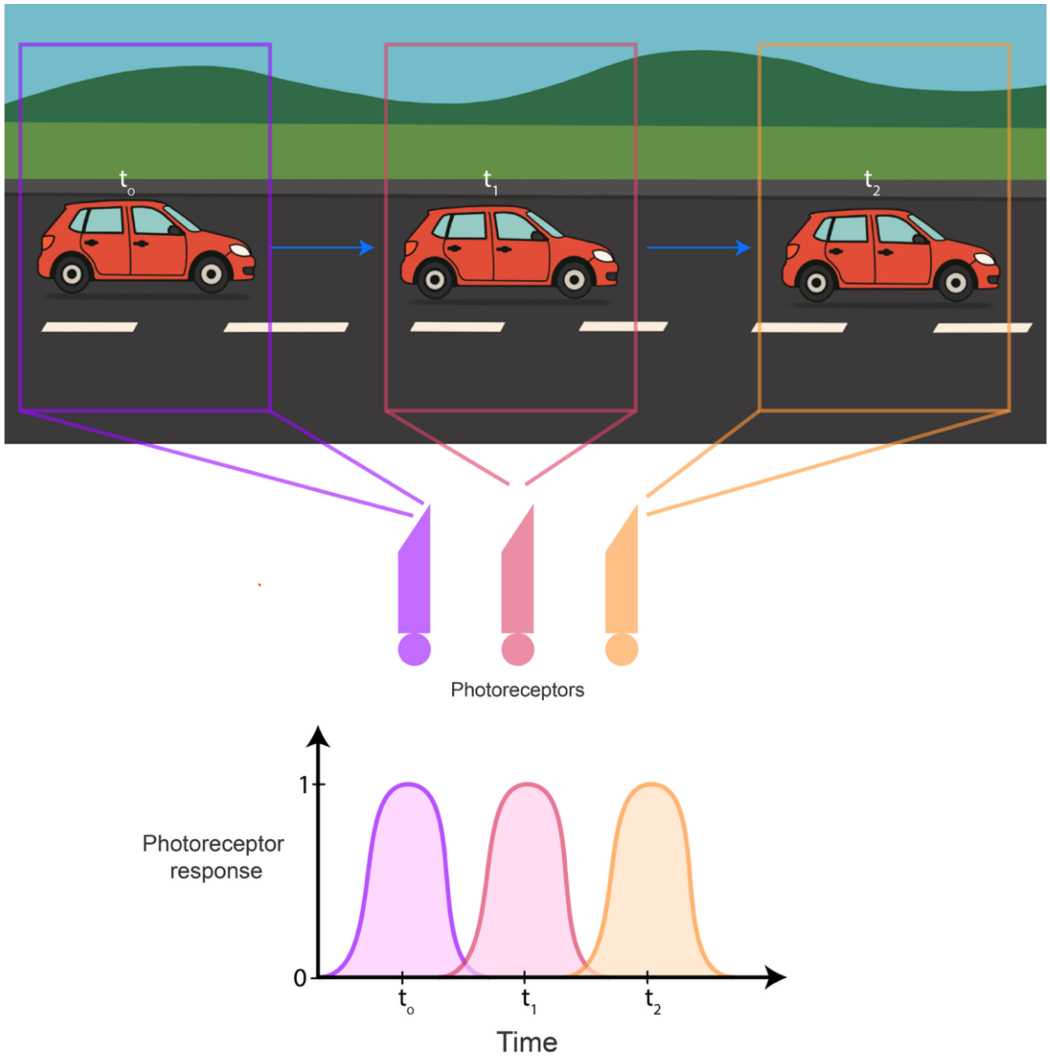

Figure 27. *As something moves across your visual field, different photoreceptors with spatially offset receptive fields are activated in temporal sequence. Imagine an RGC that, via bipolar cells, receives input from the 3 spatially offset photoreceptors shown above. As a moving stimulus enters the RGC's receptive field, moving leftward, the photoreceptor on the left responds first, followed by the middle photoreceptor, and the photoreceptor on the right responds last. This sequential activation produces time-delayed signals that the visual system can use to detect the direction of motion.*

How are direction selective receptive fields implemented in the visual system?

Direction selectivity arises when neurons selectively respond to motion in a particular direction. Below we outline some methods the brain uses to implement direction selective receptive fields from spatially offset photoreceptor-mediated signals.

In **Figure 28**, two neurons with adjacent receptive fields (blue and pink) both synapse onto a downstream neuron (purple). The downstream neuron fires only if the excitatory signals from both inputs arrive simultaneously. A stimulus moving rightward will first activate the blue neuron, whose signal reaches the downstream neuron slightly earlier than the pink neuron's signal. However, if a time delay (Δt) is introduced to the signal transmission from the blue cell to the purple cell, then motion in one specific direction (rightwards) causes both excitatory inputs to arrive together, summating and driving the downstream neuron to fire—this is the downstream neuron's *preferred direction*. Motion in the opposite direction (leftwards) activates the pink cell first, and then the time delay with the blue cell means its input will not summate with that of the pink cell—this is the *null direction*.

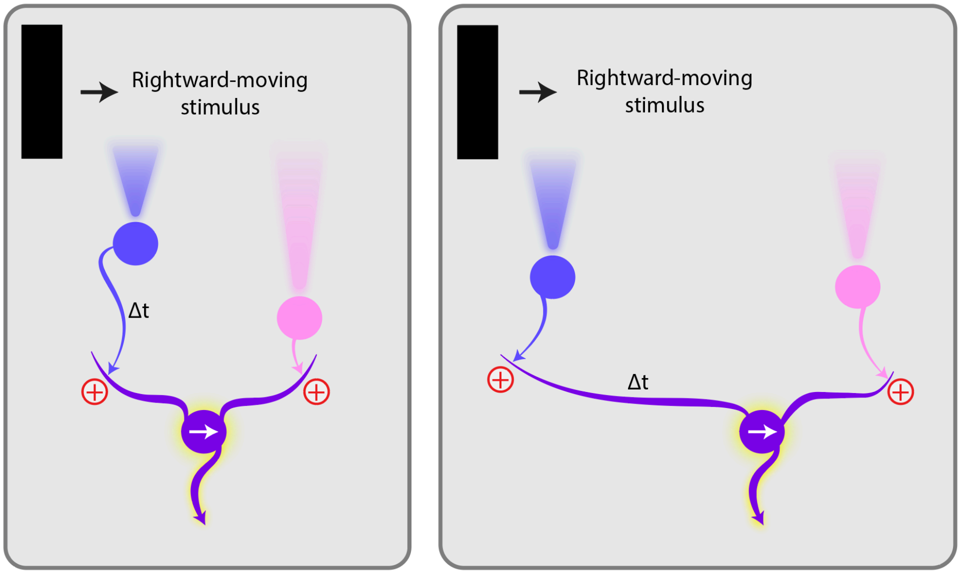

Figure 28. *Circuitry underlying direction selective response of a neuron that maximally responds to rightward motion. Two possible mechanisms could generate this delay in a purely excitatory circuit. First, introducing a delay in the presynaptic (blue) neuron, so that there is a lag between when it first fires and when its signal reaches the downstream neuron (left). Alternatively, the delay could occur within the postsynaptic (purple) neuron's dendrites, where the signal from the blue neuron takes slightly longer to reach the cell body (right). Both these mechanisms ensure that the excitatory signals from the pink neuron and blue neuron arrive at the downstream purple neuron simultaneously and summate only for rightward motion to drive firing of the downstream neuron.*

While the circuit above only utilized a time delay between two excitatory inputs to generate direction selective responses, another mechanism involves using a time delay of an inhibitory input neuron. In **Figure 29**, two neurons with adjacent receptive fields (blue and pink) both synapse onto a downstream neuron (purple). A stimulus moving leftward will first activate the pink neuron, whose signal reaches the downstream neuron slightly earlier than the blue neuron's signal.

However, when a time delay (Δt) is introduced to the inhibitory pink neuron's inputs, motion in the null direction (leftwards) causes both excitatory and inhibitory inputs to arrive together, effectively cancelling each other out, and preventing firing of the downstream neuron.

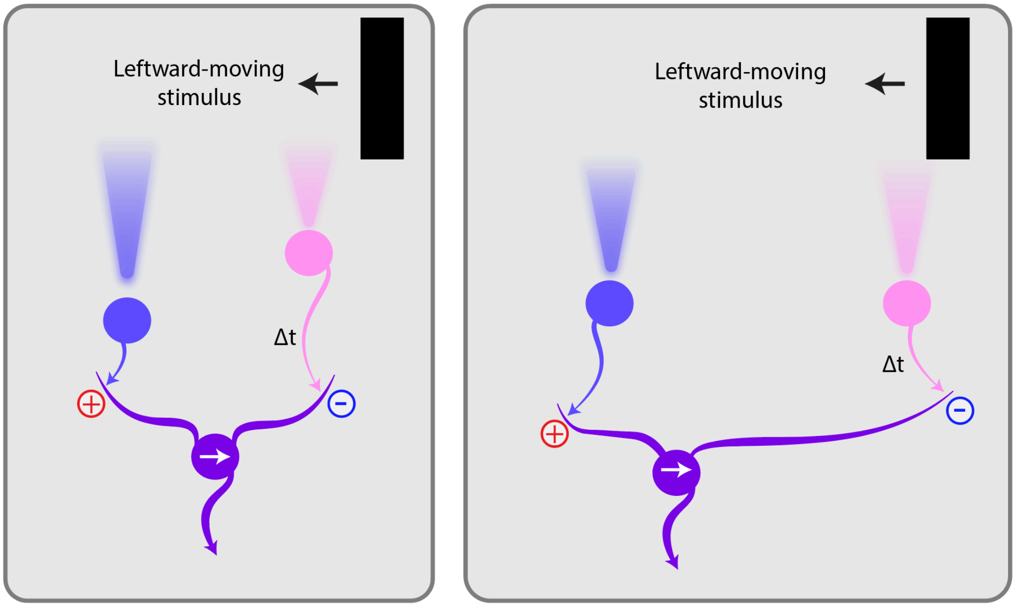

Figure 29. *Alternative circuitry underlying a direction-selective responses of a neuron that only responds to motion moving rightward. Two possible mechanisms could generate this delay in a circuit with mixed excitatory and inhibitory inputs. First, introducing a delay in the presynaptic (pink) neuron, so that there is a lag between when it first fires and when its signal reaches the downstream neuron (left). Alternatively, the delay could occur within the postsynaptic (purple) neuron's dendrites, where the signal from the pink neuron takes slightly longer to reach the cell body (right). Both of these mechanisms ensure that inhibitory signals from the pink neuron and excitatory signals from the blue neuron arrive at the downstream purple neuron simultaneously for leftward motion, cancelling each other out and preventing firing of the downstream neuron to leftward motion.*

Lesson 3 Objective

Build a circuit that models a direction selective retinal ganglion cell to understand how the visual system detects motion. First, test your circuit by activating different combinations of LEDs (use the GUI's 'Motion' mode, and select a speed and direction of motion). Then, sweep your hand across RetINaBox's field of view to test the robustness of your circuit.

The key to direction selectivity lies in asymmetric circuit connectivity that differentially processes visual stimuli moving in one direction vs. the other. In RetINaBox, these asymmetric differences can be implemented with **time delays** (see above for more info on delays) that can be asymmetrically added in the Connectivity Manager along the right-left axis of the 3 x 3 photoreceptor array. These delays ensure that inputs to an RGC only summate (and the ganglion cell fires) when a stimulus moves in the RGC's preferred direction.

Note

- *In this section, wave your hand back and forth between the LEDs and photoreceptors to test your circuit's functionality. *We don't recommend using the Visual Stimulus Tool to test moving stimuli, due to edge artifacts that can drive spurious responses when the outer boundaries of the Visual Stimulus Tool enter and exit RetINaBox's visual field.*

Depending on the time delays you've assigned to each photoreceptor, you may need to test different stimulus speeds (i.e. move your hand at different speeds) to observe the ganglion cell firing specifically to a single direction of movement.

Activity #1: build a leftward motion direction selective ganglion cell ←

Build a circuit where RGC1 responds to a vertical line of light moving leftwards. Test your circuit by moving your hand across your array in both left and right directions.

This activity emphasizes how direction selective circuits detect motion in a specific direction, illustrating how time delays along the preferred-null motion axis and specific circuit wiring determines a neuron's preferred direction of motion.

Activity #2: build a rightward motion direction selective ganglion cell →

Next, build a second circuit so that RGC2 responds to a vertical line of light moving rightwards. Test your circuit by moving your hand across your array in both right and left directions. You should now be able to selectively activate RGC1 when your hand sweeps leftward across the RetINaBox photoreceptor array, whereas only RGC2 gets activated when you sweep your hand rightward.

This activity emphasizes that the same photoreceptors can contribute to multiple direction-selective circuits with different preferred directions, depending on the configuration of the connections to the downstream neuron.

Activity #3: build a slow vs. fast motion preferring direction selective ganglion cell →

Build a circuit where RGC1 responds to a vertical line of light slowly moving rightwards, while RGC2 responds to a vertical line moving in the same direction, but more quickly. Test your circuit by moving your hand rightwards across your array at various speeds or use the Visual Stimulus Controller to run various speeds of a vertical line moving rightward vs. leftward. **Tip – to generate RGCs with preferences for different speeds, think about what changing the time delay will accomplish.*

This activity emphasizes how the timing of inputs can alter a neuron's preferred speed of motion, demonstrating an example of temporal frequency selectivity.

Challenge: Block Breaker with direction selective circuits

Block Breaker is an arcade game where players control a paddle using leftward and rightward inputs to bounce a ball to break several rows of blocks. The goal is to clear all the blocks without letting the ball drop. Your task is to configure the 2 RGCs with opposite direction selectivity (i.e. make two robust direction selective ganglion cells, one for leftward motion, the other for rightward motion) and use them as the input controls for the game inside the RetINaBox GUI.

- **Step 1:** In the Connectivity Manager, configure both ganglion cell circuits so that RGC1 is selective *only* for leftward motion and RGC2 is selective *only* for rightward motion.
- **Step 2:** Load the game. From the Menu tab in the GUI, navigate to 'Block Breaker' (Lessons > Lesson 3 > Block Breaker).
- **Step 3:** You're now ready to play (see **Figure 30**)! The block breaker paddle is controlled by the output responses from RGC1 and RGC2.

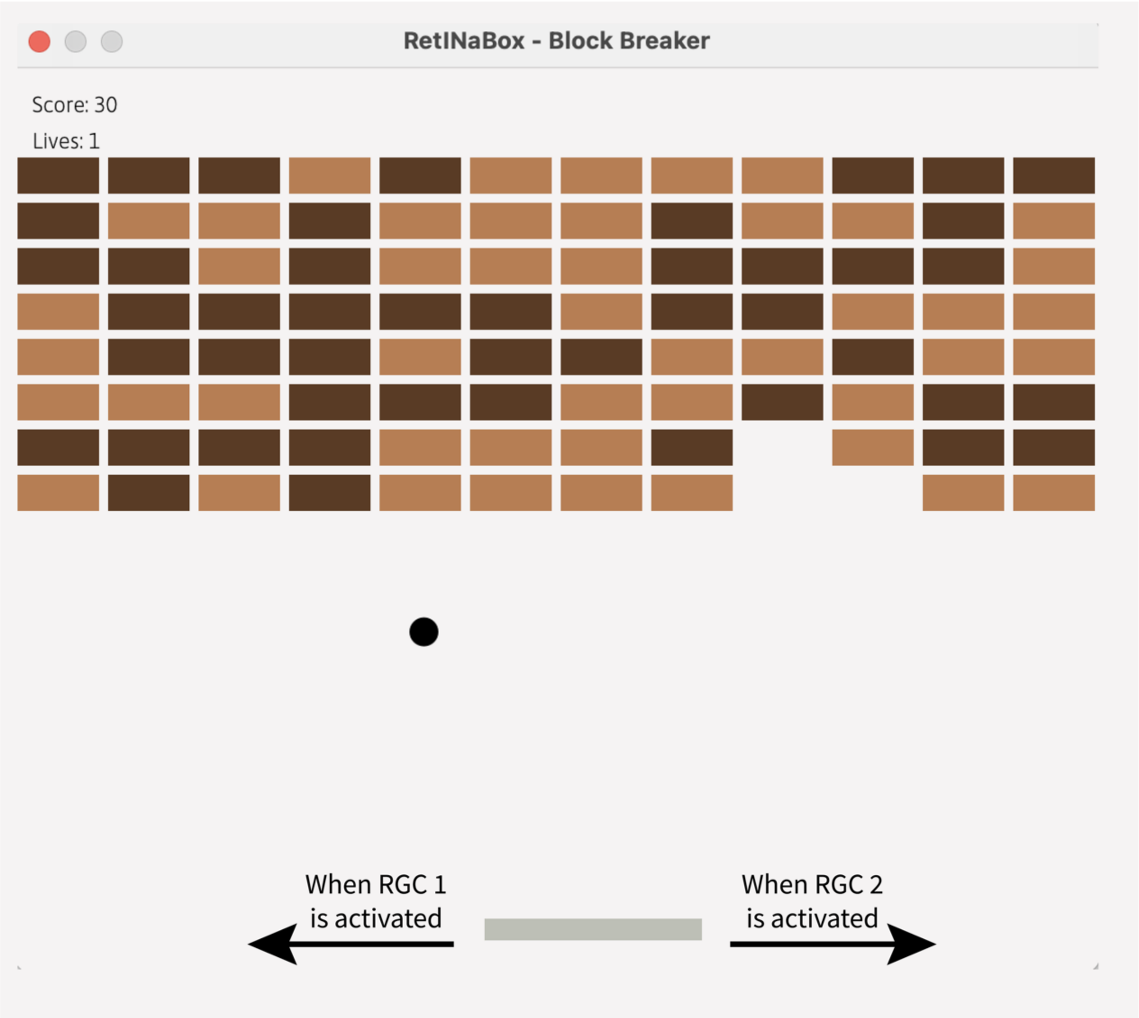

Figure 30. *Block Breaker in the RetINaBox GUI. RGC1 activation moves the paddle to the left, while RGC2 activation moves the paddle to the right.*

This challenge emphasizes how direction-selective circuits allow the visual system to detect motion in specific directions, and how the outputs of RetINaBox can be used to interact with external systems. Furthermore, the point can be emphasized that if we consider RetINaBox to be a model of the brain/retina, and since here we are controlling a computer with the output from RetINaBox, this can be considered as a virtual brain-computer-interface (BCI).

Lesson 4: Discovery Mode

Welcome to Discovery Mode! You've made it this far, which means that you're ready to perform some real experiments! In this section you'll get a taste of what it's really like to be a visual neuroscientist!

So far, you've explored ON/OFF, center-surround, orientation selective, and direction selective receptive fields—all circuits that have already been well characterized by vision scientists. But the quest to understand what neurons in the brain "like to see" is far from over. Even today, neuroscientists are still working to discover which kinds of stimuli best activate visual neurons in various parts of the brain. *Now it's your turn to discover which visual stimuli best activate some newly discovered visual neurons in RetINaBox and then discover what circuit connectivity properties underlie their feature selective responses*.

In Discovery Mode (see **Figure 31**), there are three levels of difficulty (Easy, Medium, Hard), each with its own set of challenges. To complete each challenge, you'll need to discover (i.e. correctly submit an answer) the mystery ganglion cell's target visual stimulus (the visual response that drives the ganglion cell) and the circuit connectivity (the settings in the Connectivity Manager) that underlie this feature selective response. For each challenge, you'll start with 100 points, but incorrect answers cost you 5 points. Your goal is to complete each challenge with the highest score possible.

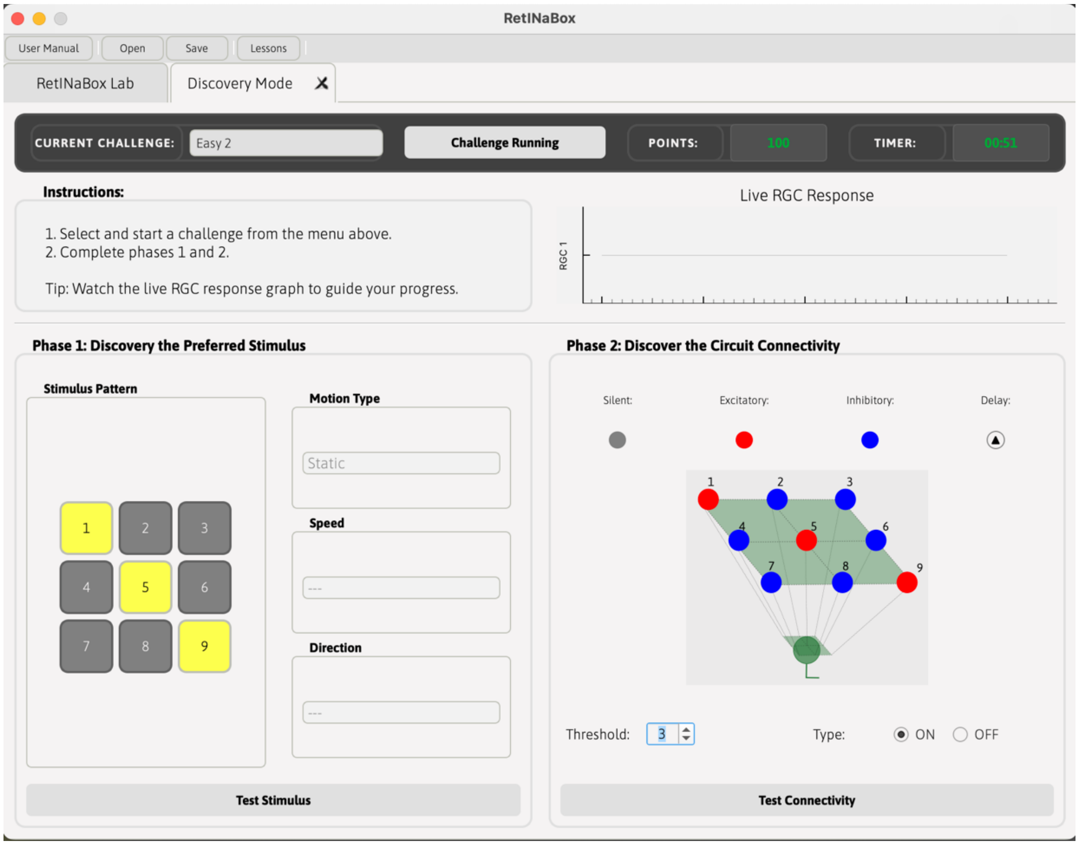

Figure 31. *Discovery Mode GUI. Each challenge has two phases: (1) discovering the preferred stimulus (left) and (2) discovering the circuit connectivity (right)*

Each challenge in Discovery Mode has two steps:

Phase 1: Discover the Preferred Stimulus

From the Menu tab, navigate to 'Discovery Mode' (Lessons > Lesson 4 > Discovery Mode). After selecting a mystery circuit from the drop-down menu in the GUI, your first task is to figure out which visual stimulus activates the RGC. Does it prefer a specific shape? A particular direction of motion? Using your Visual Stimulus Controller, the Visual Stimulus Tool, or some pieces of paper cut out with various shapes (or even your hands if you're dexterous), test different static stimuli. Use your hand to test direction selective stimuli. The mystery circuits in RetINaBox are very selective to specific visual stimuli, so make sure you're certain before submitting your answer.

Phase 2: Discover the Circuit Connectivity

Once you've discovered what the ganglion cell is tuned to, your next challenge is to discover how it obtains this selectivity**.** How are the photoreceptors connected to the ganglion cell? What kinds of delays, polarities, or spatial arrangements of the photoreceptors give rise to the ganglion cell's feature selective response? *Apply the correct settings to the Connectivity Manager to match the feature selectivity you discovered.*

Please note:

- For challenges including moving stimuli, the speed of motion specified in Step 1 must match the time delay applied to the circuit in Step 2 (e.g., faster motion requires a shorter delay)
- Each challenge can be solved using either the ON or OFF pathway (both versions of the circuit solutions should respond to the same stimulus).

Good luck! *Securing grant funding for your lab depends on your success! ;)*

Appendix

Lesson 1 Solutions: ON/OFF and Center-Surround

Activity #1: build a spot detector with an ON-center/OFF-surround receptive field

The center photoreceptor (red) is excitatory (+): it activates the ganglion cell when it detects light.

- Connect the center photoreceptor to a ganglion cell with **positive polarity** (excitatory).

The surround photoreceptors (blue) are inhibitory (-): they suppress the ganglion cell when they are activated.

- Connect each surround photoreceptor to the same ganglion cell with negative polarity (inhibitory).

Set the ganglion cell's threshold to 1. Set the cell type to ON. The ganglion cell will fire **only** when the center photoreceptor receives light. However, if any of the surround photoreceptors are also lit, their inhibition will cancel out the excitation, and the ganglion cell will not fire.

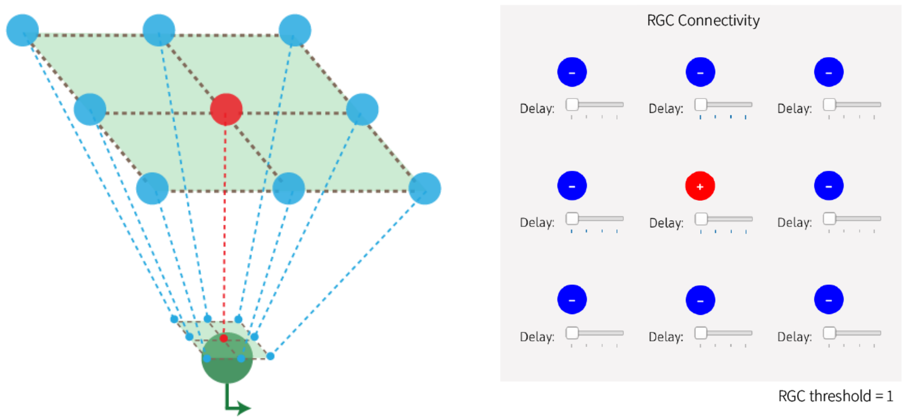

Lesson 1, Activity 1: Inputs & wiring to RGC1 (ON-center/OFF-surround cell with center receptive field)

Activity #2: build a second spot detector with a different receptive field location

Please note that the following are just two possible examples of correct solutions. However, you could have selected any receptive field within the 3 x 3 array for either ganglion cell.

Example solution 1

Inputs to RGC2: A corner photoreceptor (red) is excitatory (+): it activates the ganglion cell when it detects light. All other surround photoreceptors (blue) are inhibitory (–): they suppress the ganglion cell when they are activated. Set the ganglion cell threshold to 1. Set the cell type to ON.

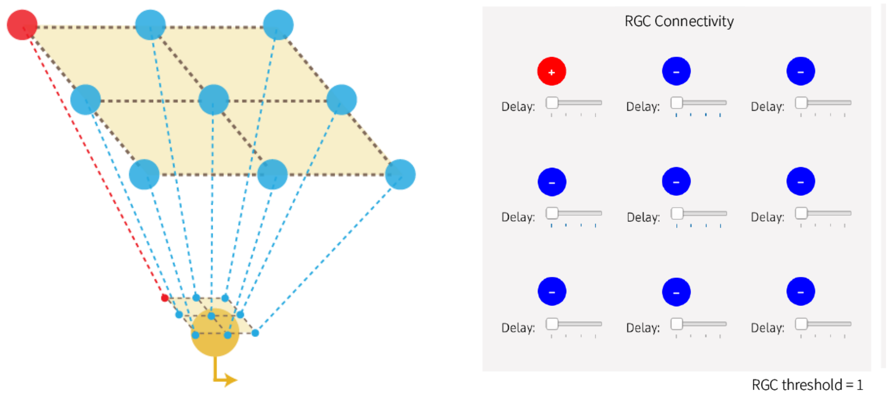

Lesson 1, Activity 2 – Example solution: Inputs and wiring to RGC2 (center-surround cell with a top left corner receptive field)

Example solution 2

Inputs to RGC2: An edge photoreceptor (red) is excitatory (+): it activates the ganglion cell when it detects light. All other surround photoreceptors (blue) are inhibitory (-): they suppress the ganglion cell when they are activated. Set the ganglion cell threshold to 1. Set the cell type to ON.

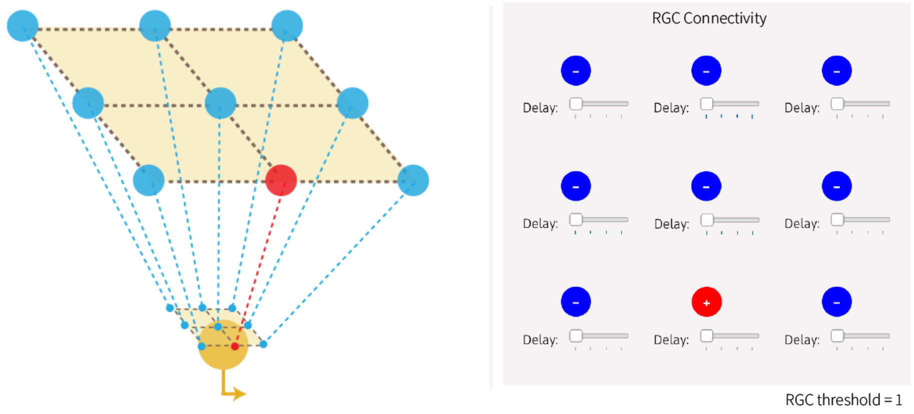

Lesson 1, Activity 2 – Alternative example solution: Inputs and wiring to RGC2, whose receptive field is on the edge of the array (center-surround cell with a bottom edge receptive field)

Activity #3: build a second spot detector with the opposite polarity

Inputs to RGC2: Set the **cell type to OFF**—this inverts the photoreceptor logic. Specifically, the center photoreceptor, which should be OFF excitatory (red, +) activates the ganglion cell when it does *not* detect light. All other surround photoreceptors (blue) are OFF inhibitory (–): they inhibit the ganglion cell when they do not detect light. Set the ganglion cell threshold to 1. This OFF-center/ON-surround cell will only fire when the surround is illuminated while the center remains dark.

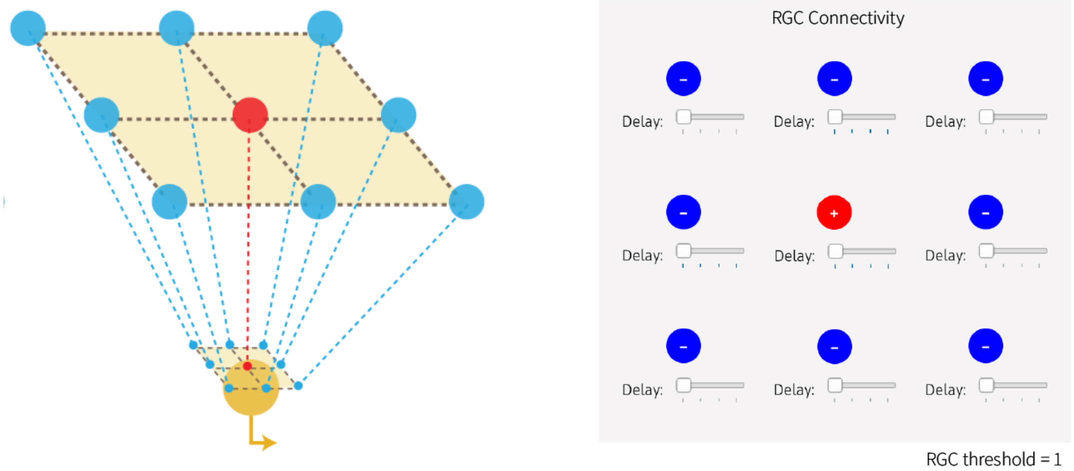

Lesson 1, Activity 3 – Inputs and wiring to RGC2 (OFF-center/ON-surround cell with a central receptive field)

Activity #4: build two spot detectors with preferences for spots of different sizes

Inputs to RGC1 (small spot detector): as described in *Solutions to Activity #1*: The center photoreceptor (red) is excitatory (**positive polarity,** +), while all other surround photoreceptors (blue) are inhibitory (negative polarity, -). Set the ganglion cell threshold to 1. Set the cell type to ON. This ganglion cell will only fire in response to a small spot of light illuminating the center photoreceptor.

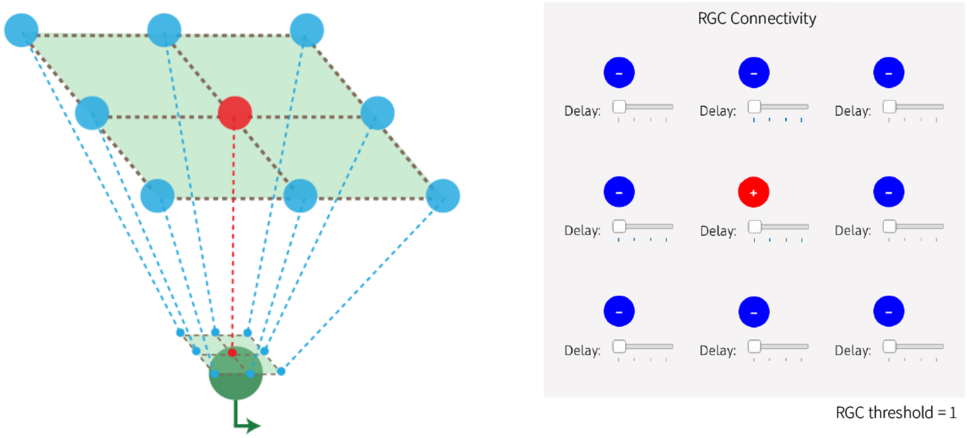

Lesson 1, Activity 4 – RGC1 wiring: Inputs & wiring to RGC1 (small center-surround cell with center receptive field)

Inputs to RGC2 (larger spot detector): The center photoreceptor (red) is excitatory (positive polarity, +) and will activate the ganglion cell when it detects light. However, the nearest surround photoreceptors (directly above, below, and to the sides) are also excitatory (+). The corner photoreceptors (blue) are inhibitory (negative polarity, -): they inhibit the ganglion cell if the stimulus becomes too large. Set the ganglion cell's threshold to 5. Set the cell type to ON. This ganglion cell will fire in response to a larger spot of light illuminating the center photoreceptor

and its side-surround photoreceptors. However, if the spot becomes too large and any of the corner surround photoreceptors are also lit, this inhibition will subtract from the excitation, and the ganglion cell will not reach the threshold to fire.

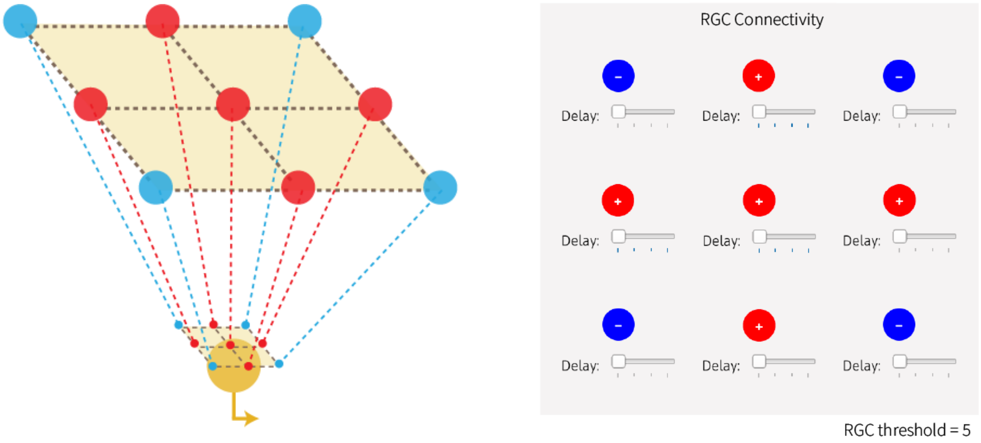

Lesson 1, Activity 4 – RGC2 wiring: Inputs & wiring to RGC2 (larger center-surround cell)

Challenge: Codebreaking with spot detectors

Challenge #1

Inputs to RGC1: This center-surround cell responds only when the four photoreceptors on the top right are activated. These four photoreceptors should have positive polarity (red, excitatory, +). All other surround photoreceptors have negative polarity (blue, inhibitory, -). The bottom left photoreceptor is left unconnected (grey), so that RGC1 and RGC2 can be co-activated. The ganglion cell threshold is set to 4. The cell type is set to ON.

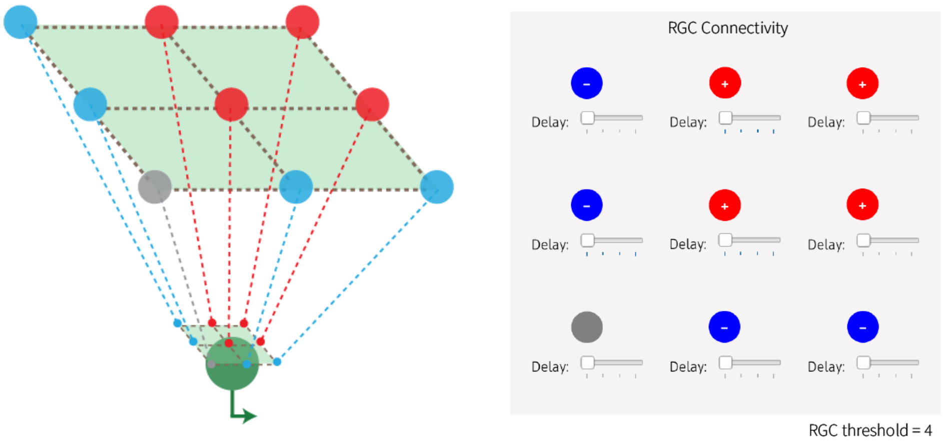

Lesson 1, Challenge 1: Inputs & wiring to RGC1 (large spot detector with top right receptive field position)

Inputs to RGC2: This center-surround cell responds only when the bottom left photoreceptor is activated. This photoreceptor should have positive polarity (red, excitatory, +). All other surround

photoreceptors have negative polarity (blue, inhibitory, -), except for the top 4 photoreceptors, corresponding RGC1's receptive field center (see above), which should be left unconnected (grey). This ensures that RGC1 and RGC2 can be co-activated. The ganglion cell threshold is set to 1. Set the cell type to ON.

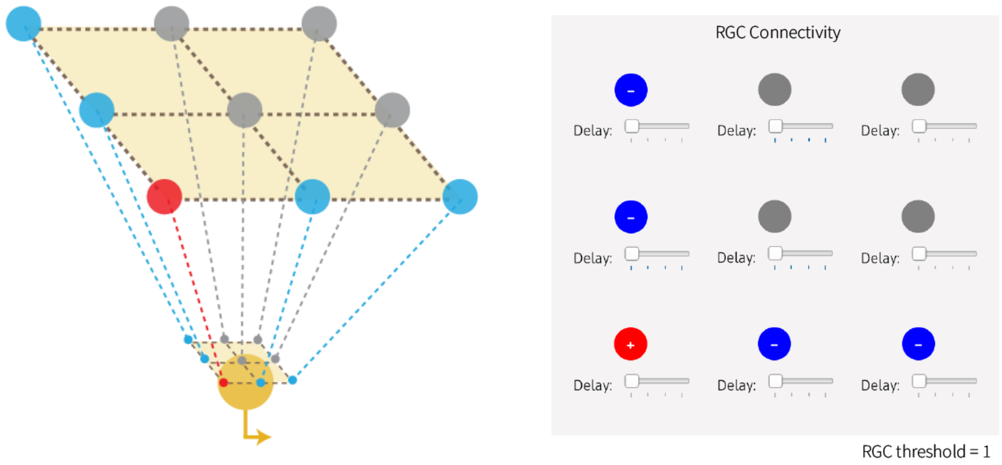

Lesson 1, Challenge 1: Inputs & wiring to RGC2 (small spot detector with bottom left receptive field)

Secret message #1: ***Dear reader, dread red area.***

Extra problems

Challenge #2

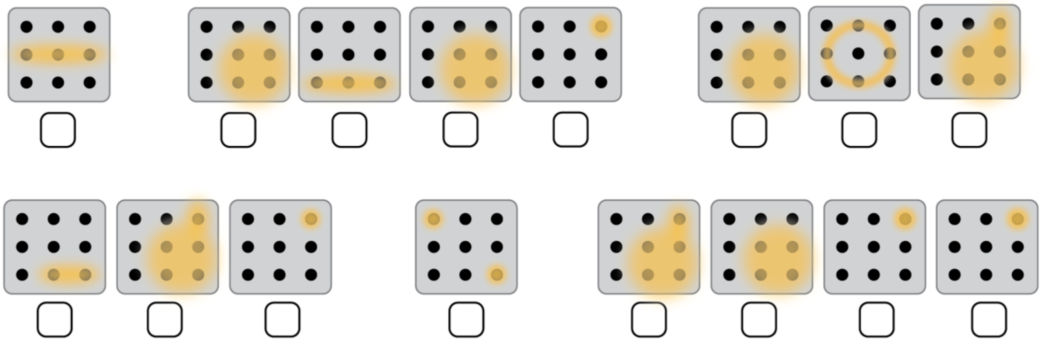

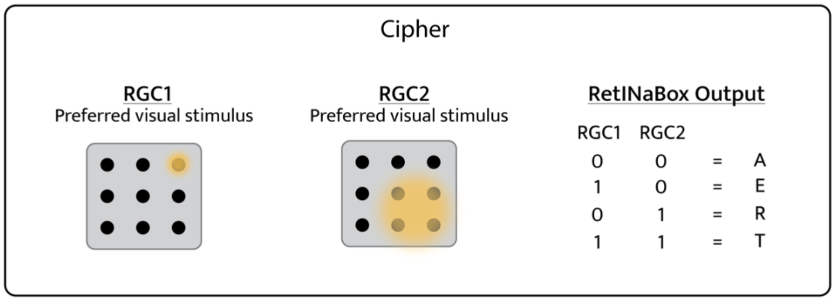

Inputs to RGC1: This center-surround cell responds only when the top right corner photoreceptor is activated. This photoreceptor should have positive polarity (red, excitatory, +). All other surround photoreceptors have negative polarity (blue, inhibitory, -), except for the center and bottom photoreceptors in the middle and right column, which are left silent (grey), so that RGC1 and RGC2 can be co-activated. The ganglion cell threshold is set to 1. The cell type is set to ON.

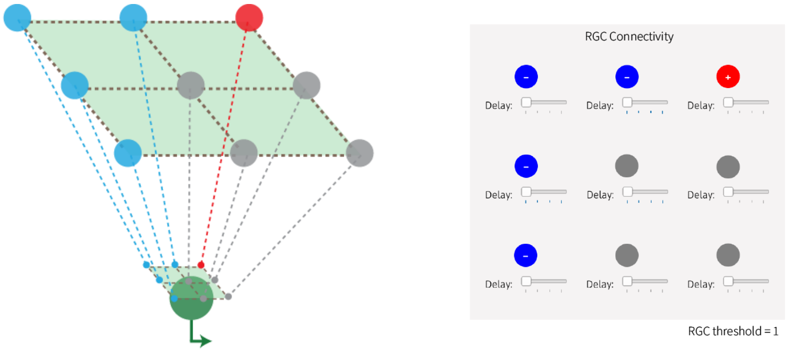

Lesson 1, Challenge 2: Inputs & wiring to RGC1 (small spot detector with top right receptive field)

Inputs to RGC2: This cell is selective to a medium-sized spot in the bottom right corner of the array. It responds only when the four photoreceptors in the bottom right of the array are activated. These photoreceptors should have positive polarity (red, excitatory, +). All other surround photoreceptors have negative polarity (blue, inhibitory, -), except for the photoreceptor connected to RGC1's center, which should be left silent. The ganglion cell threshold is set to 4. Set the cell type to ON.

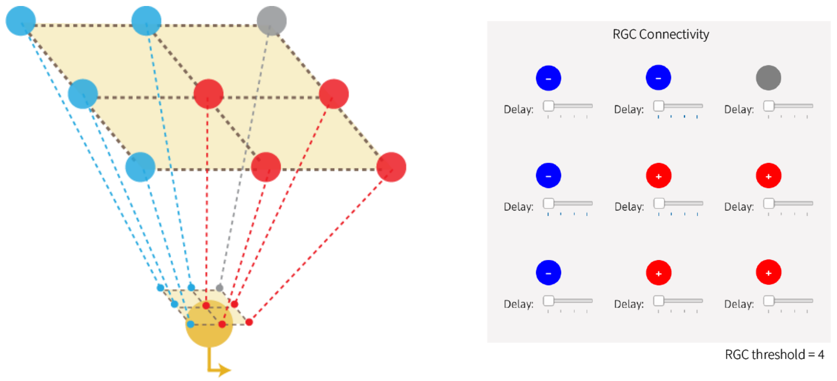

Lesson 1, Challenge 2: Inputs & wiring to RGC2 (large spot detector, bottom right)

Secret message #2: ***A rare rat ate a tree.***

Challenge #3

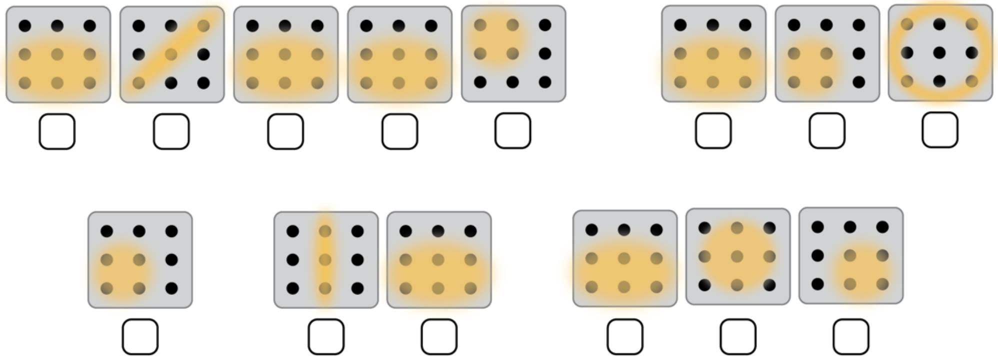

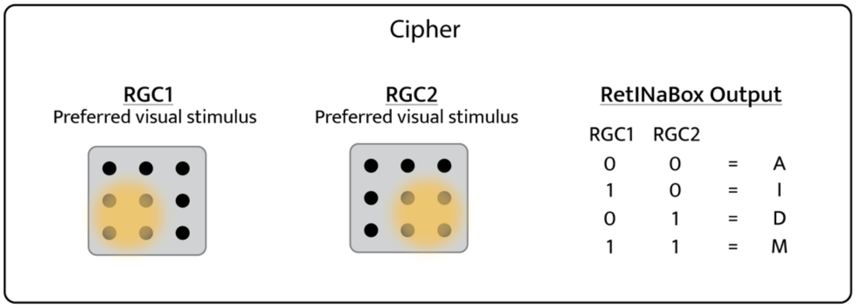

Inputs to RGC1: This cell is selective to a medium sized spot of light in the bottom left corner of the array. It responds only when the four photoreceptors in the bottom left corner are activated. These photoreceptors should have positive polarity (red, excitatory, +). All other surround photoreceptors have negative polarity (blue, inhibitory, -), except for the center and bottom photoreceptors in the right column, which are left silent (grey), so that RGC1 and RGC2 can be co-activated. The ganglion cell threshold is set to 4. The cell type is set to ON.

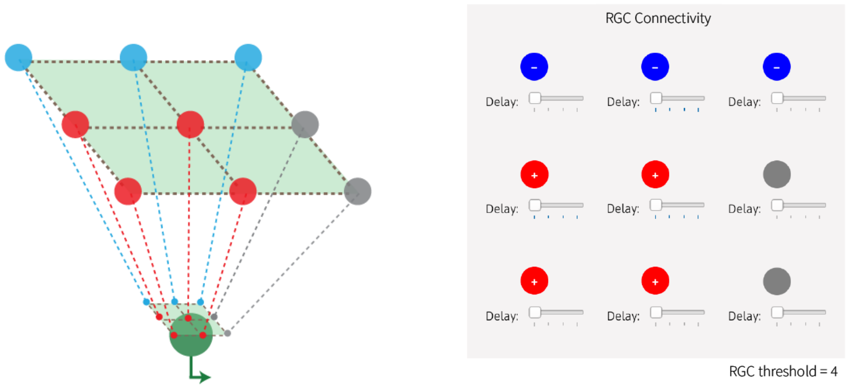

Lesson 1, Challenge 3: Inputs & wiring to RGC1 (large spot detector, bottom left)

Inputs to RGC2: This cell is selective to a medium sized spot of light in the bottom right corner of the array. It responds only when the four photoreceptors in the bottom right corner are activated. These photoreceptors should have positive polarity (red, excitatory, +). All other surround photoreceptors have negative polarity (blue, inhibitory, -), except for the center and bottom photoreceptors in the left column, which are left silent (grey), so that RGC1 and RGC2 can be co-activated. The ganglion cell threshold is set to 4. The cell type is set to ON.

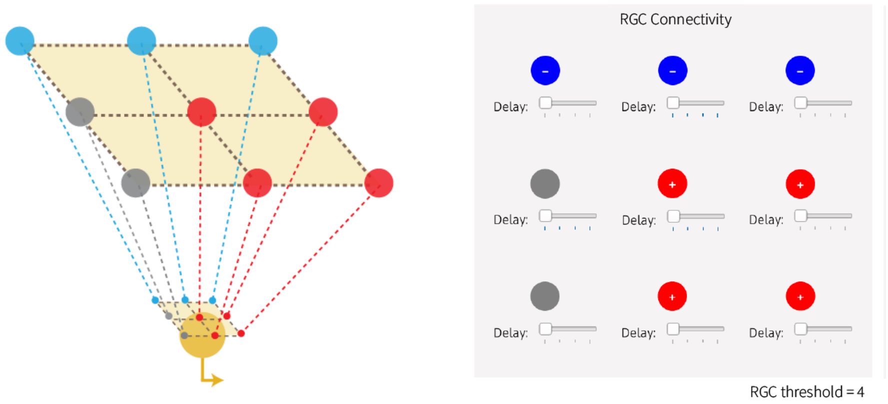

Lesson 1, Challenge 3: Inputs & wiring to RGC2 (large spot detector, bottom right)

Secret message #3: ***Mamma Mia! I am mad.***

Challenge #4

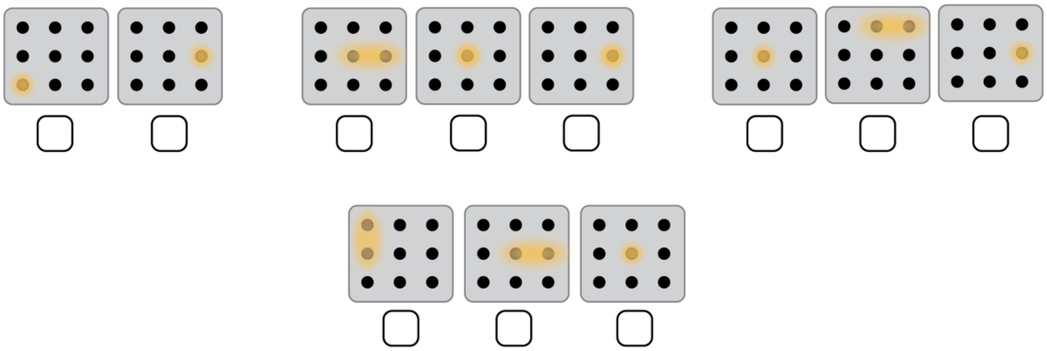

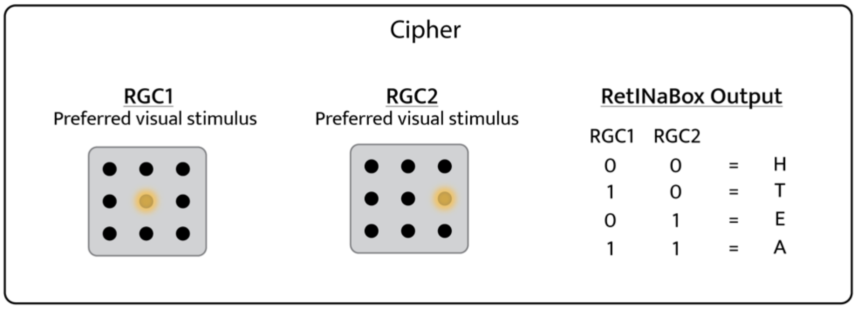

Inputs to RGC1: This center-surround cell is selective to a small spot of light. It responds only when the center photoreceptor is activated. This photoreceptor should have positive polarity (red, excitatory, +). All other surround photoreceptors have negative polarity (blue, inhibitory, -), except for the photoreceptor connected to RGC2's center, which should be left silent. The ganglion cell threshold is set to 1. The cell type is set to ON.

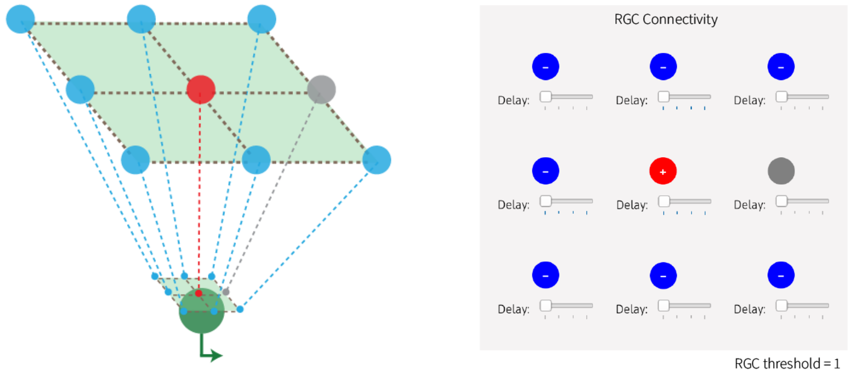

Lesson 1, Challenge 4: Inputs & wiring to RGC1 (small spot detector, center).

Inputs to RGC2: This center-surround cell responds only when the right photoreceptor in the middle row is activated. This photoreceptor should have positive polarity (red, excitatory, +). All other surround photoreceptors have negative polarity (blue, inhibitory, -), except for the center photoreceptor, which is left silent (grey), so that RGC1 and RGC2 can be co-activated. The ganglion cell threshold is set to 1. The cell type is set to ON.

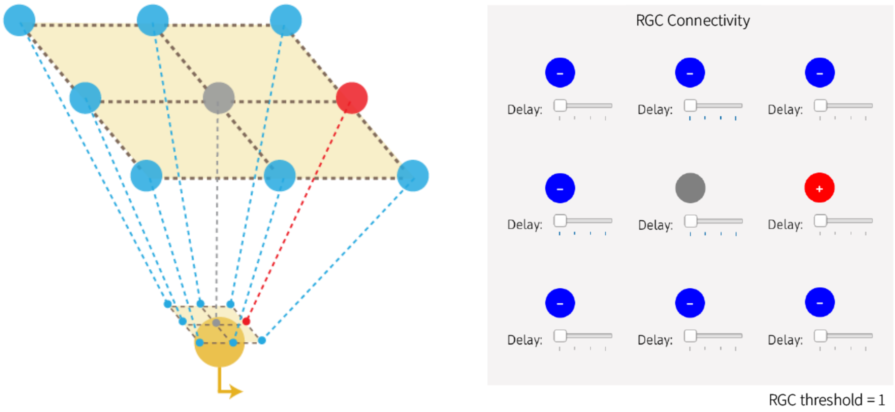

Lesson 1, Challenge 4: Inputs & wiring to RGC2 (small spot detector with right side receptive field).

Secret message #4: ***He ate the hat.***

Challenge #5

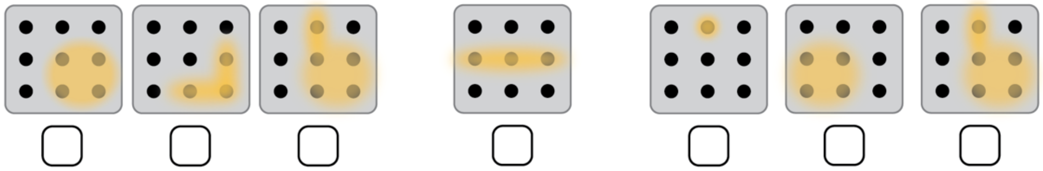

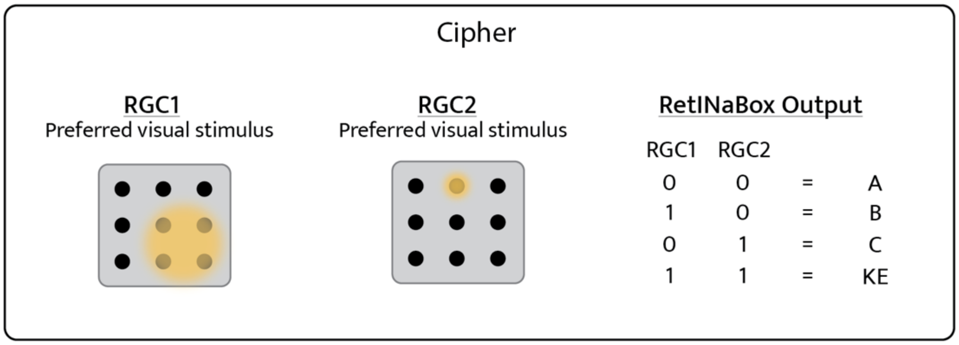

Inputs to RGC1: This center-surround cell responds only when the four photoreceptors on the bottom right are activated. These four photoreceptors should have positive polarity (red, excitatory, +). All other surround photoreceptors have negative polarity (blue, inhibitory, -), except for the photoreceptor connected to RGC2's center, which should be left silent. The ganglion cell threshold is set to 4. The cell type is set to ON.

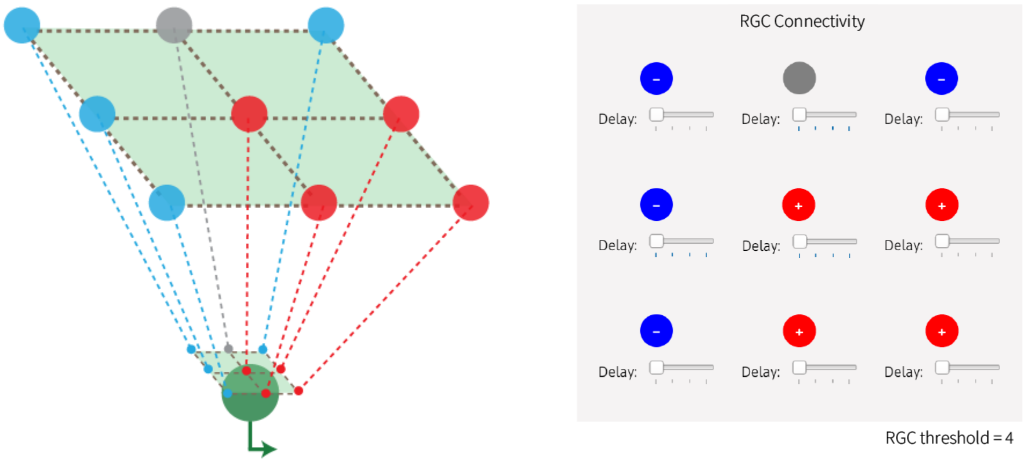

Lesson 1, Challenge 5: Inputs & wiring to RGC1 (larger spot detector with bottom right receptive field)

Inputs to RGC2: This center-surround cell responds only when the middle photoreceptor in the top row is activated. This photoreceptor should have positive polarity (red, excitatory, +). All other surround photoreceptors have negative polarity (blue, inhibitory, -), except for the photoreceptors connected to RGC1's center, which should be left silent. The ganglion cell threshold is set to 1. The cell type is set to ON.

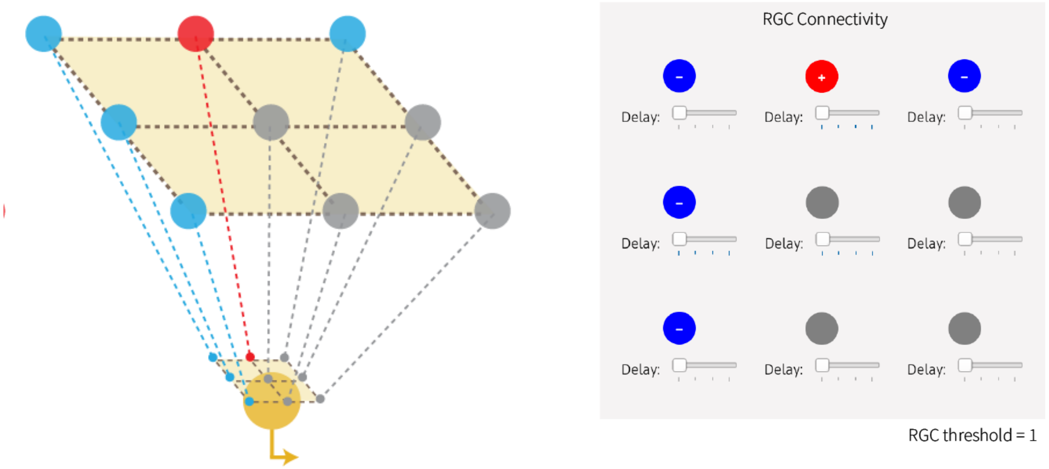

Lesson 1, Challenge 5: Inputs & wiring to RGC2 (spot detector with top center receptive field)

Secret message #5: ***Bake a cake.***

Challenge #6

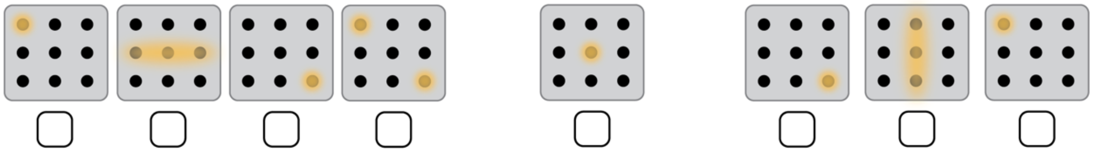

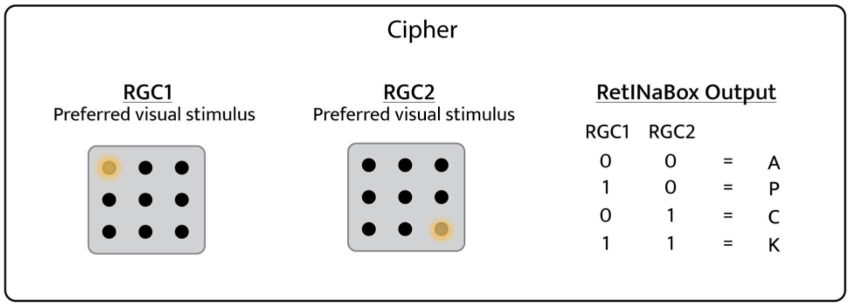

Inputs to RGC1: This center-surround cell responds only when the top left corner photoreceptor is activated. This photoreceptor should have positive polarity (red, excitatory, +). All other surround photoreceptors have negative polarity (blue, inhibitory, -), except for the bottom right photoreceptor, which is left silent (grey), so that RGC1 and RGC2 can be co-activated. The ganglion cell threshold is set to 1. The cell type is set to ON.

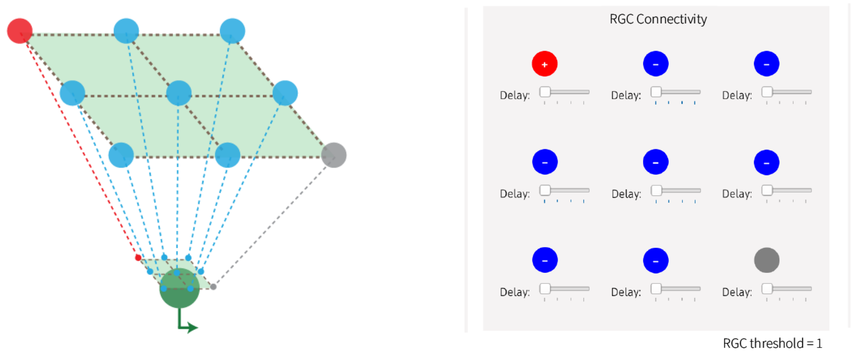

Lesson 1, Challenge 6: Inputs & wiring to RGC1 (spot detector with top left receptive field)

Inputs to RGC 2: This center-surround cell is selective to a spot of light in the bottom right corner of the photoreceptor array. It responds only when the bottom right photoreceptor is activated. This photoreceptor should have positive polarity (red, excitatory, +). All other surround photoreceptors have negative polarity (blue, inhibitory, -), except for the photoreceptor connected to RGC2's center, which should be left silent. The ganglion cell threshold is set to 1. The cell type is set to ON.

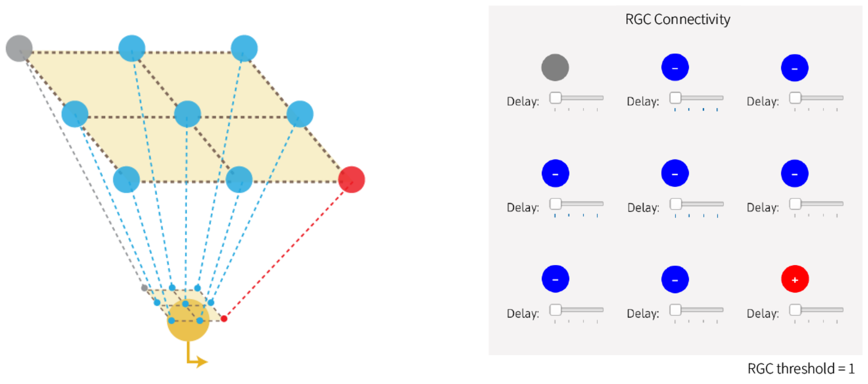

Lesson 1, Challenge 6: Inputs & wiring to RGC2 (spot detector with bottom right receptive field)

Secret message #6: ***Pack a cap.***

Lesson 2 Solutions: Orientation Selectivity

Activity #1: build a ganglion cell that detects a vertical line

Inputs to Ganglion Cell 1: Connect 3 adjacent photoreceptors in a straight vertical line (e.g., middle column) to the ganglion cell, with a positive (excitatory, +) polarity. Connect the 6 other surrounding photoreceptors to the same ganglion cell, with a negative (inhibitory, -) polarity. Set the ganglion cell threshold to 3, and the cell type to ON, so that the ganglion cell will *only* fire when all 3 photoreceptors are activated.

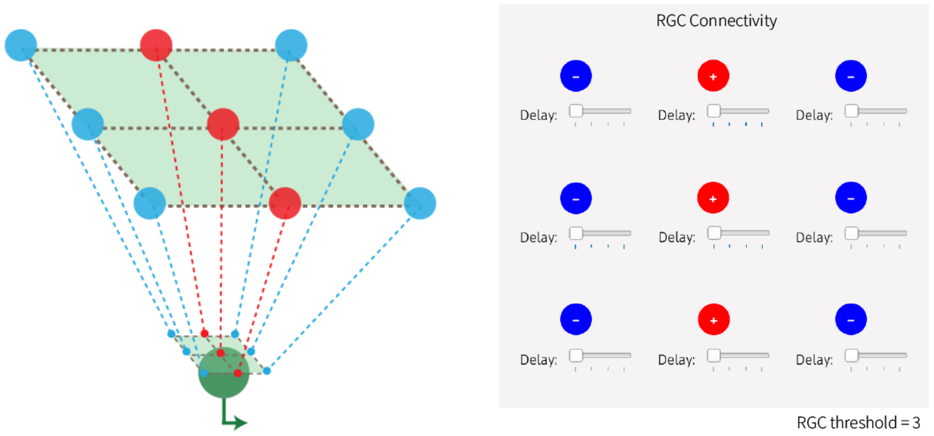

Lesson 2, Activity 1: Inputs & wiring to RGC1 (vertical orientation selective cell)

Please note that the above represents just one of three possible examples of a correct solution. However, you could have selected a vertical receptive field in the left or right column of the 3 x 3 array.

Activity #2: build an OFF-ganglion cell that detects a vertical line

Inputs to Ganglion Cell 2: Set the cell type to OFF. Connect 3 adjacent photoreceptors in a straight vertical line (e.g., middle column) to the ganglion cell, with a positive (excitatory, +) polarity. Connect the 6 other surrounding photoreceptors to the same ganglion cell, with a negative (inhibitory, -) polarity. Set the ganglion cell threshold to 3, so that the ganglion cell will *only* fire when all 3 photoreceptors in the center column are dark, but the surround (left and right columns) is illuminated.

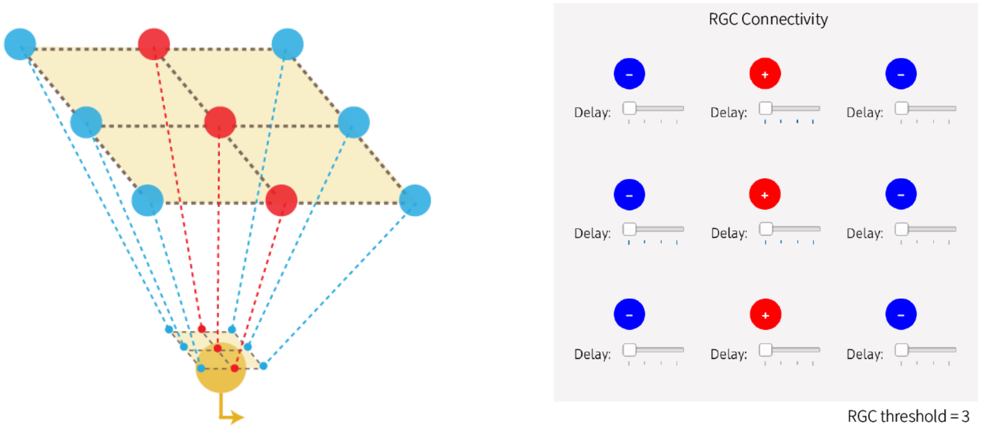

Lesson 2, Activity 2: Inputs & wiring to RGC2 (vertical orientation selective OFF cell)

Activity #3: build a second ganglion cell that detects a diagonal line

Inputs to Ganglion Cell 2: Connect 3 photoreceptors along the diagonal line to the ganglion cell, with a positive (excitatory, +) polarity. Connect the 6 other off-diagonal photoreceptors to the same ganglion cell, with a negative (inhibitory, -) polarity. Set the ganglion cell threshold to 3, and the cell type to ON, so that the ganglion cell will *only* fire when all 3 photoreceptors are activated but will be inhibited if any of the off-diagonal photoreceptors are activated.

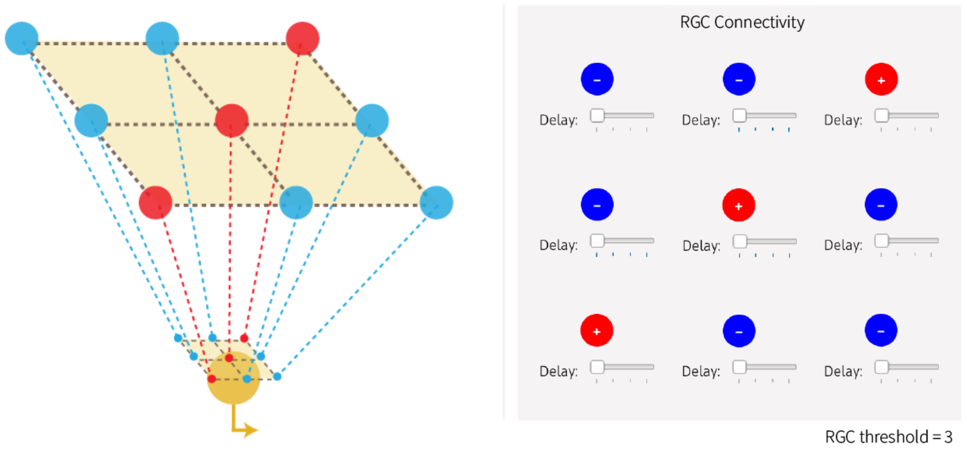

Lesson 2, Activity 3: Inputs & wiring to RGC2 (diagonal orientation selective cell)

Please note that the above represents just one of two possible examples of a correct solution. However, you could have selected a diagonal receptive field oriented at 180º to the solution shown above..

Activity #4: build two ganglion cells that detect vertical lines of different thicknesses

Inputs to Ganglion Cell 1: connect two adjacent columns of 3 photoreceptors in a vertical line (for example, middle column + side column) to Ganglion Cell 1, with a positive (excitatory, -) polarity. Connect the 3 other surround photoreceptors to the same ganglion cell, with a negative (inhibitory, -) polarity. Set the ganglion cell threshold to 6, and the cell type to ON, so that the ganglion cell will *only* fire when all 6 (+) photoreceptors are activated.

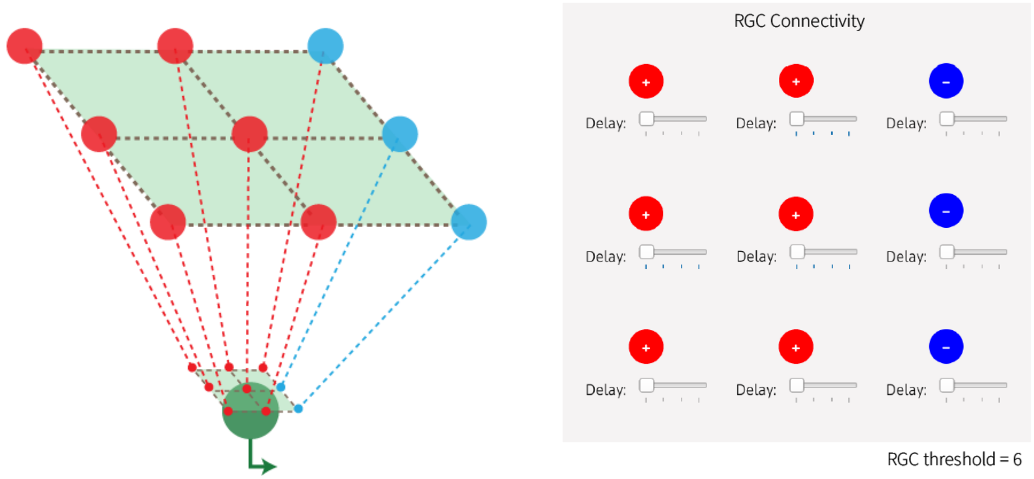

Lesson 2, Activity 4: Inputs & wiring to RGC1 (thick vertical orientation selective cell)

Inputs to Ganglion Cell 2: connect one column of 3 photoreceptors in a vertical line (for example, right column) to Ganglion Cell 2, with a positive (excitatory, +) polarity. Connect the 6 other surrounding photoreceptors to the same ganglion cell, with a negative (inhibitory, -) polarity. Set the ganglion cell threshold to 3, and the cell type to ON, so that the ganglion cell will *only* fire when all 3 (+) photoreceptors are activated.

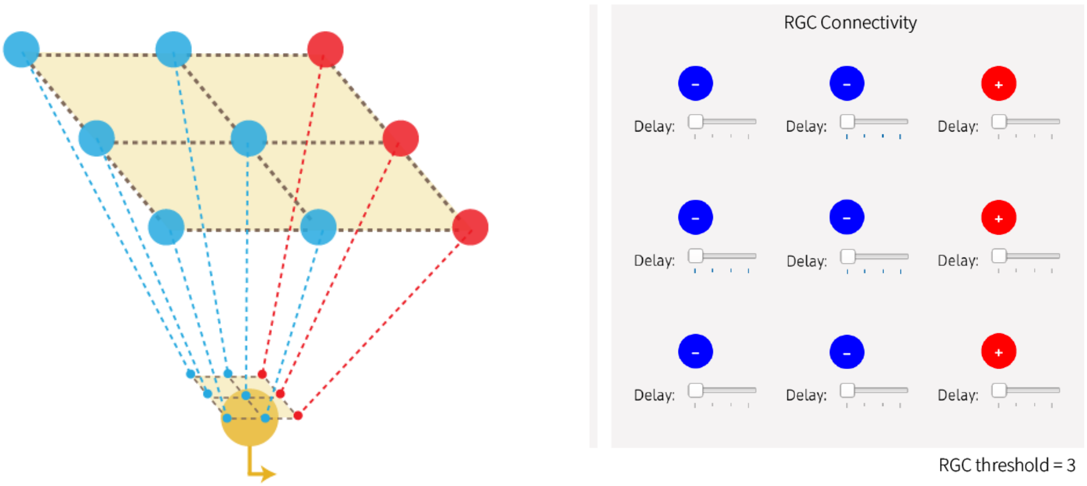

Lesson 2, Activity 4: Inputs & wiring to RGC2 (thin vertical orientation selective cell)

Please note that the above represents one of several possible examples of a correct solution. You could have configured each ganglion cell with vertical receptive field in various locations (i.e., shifted left or right).

Activity #5: build two ganglion cells that detect vertical lines of different lengths (end stopping).

Inputs to Ganglion Cell 1: Connect 3 adjacent photoreceptors in a straight vertical line (e.g., middle column) to the ganglion cell, with a positive (excitatory, +) polarity. Connect the 6 other surrounding photoreceptors to the same ganglion cell, with a negative (inhibitory, -) polarity. Set the ganglion cell threshold to 3, and the cell type to ON, so that the ganglion cell will *only* fire when all 3 (+) photoreceptors are activated by a vertical line of light.

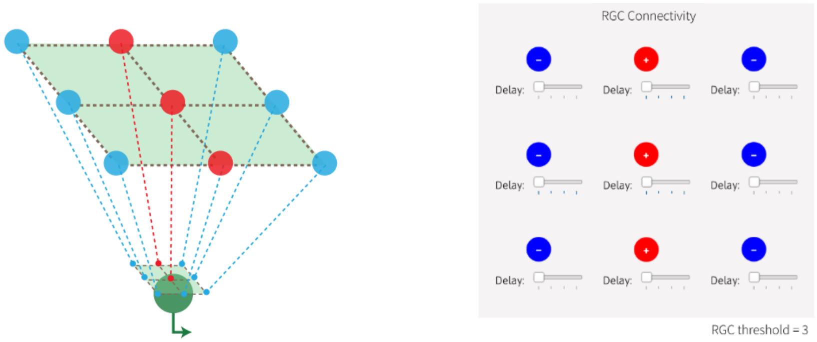

Lesson 2, Activity 5: Inputs & wiring to RGC1 (long vertical orientation selective cell)

Inputs to Ganglion Cell 2: Connect 2 adjacent photoreceptors in a straight vertical line (e.g., middle column) to the ganglion cell, with a positive (excitatory, +) polarity. Connect the 7 other surrounding photoreceptors to the same ganglion cell, with a negative (inhibitory, -) polarity. Set the ganglion cell threshold to 2, and the cell type to ON, so that the ganglion cell will *only* fire

when both (+) photoreceptors are activated by a vertical line of light of the correct length. RGC2 will be inhibited should the line extend past its excitatory receptive field.

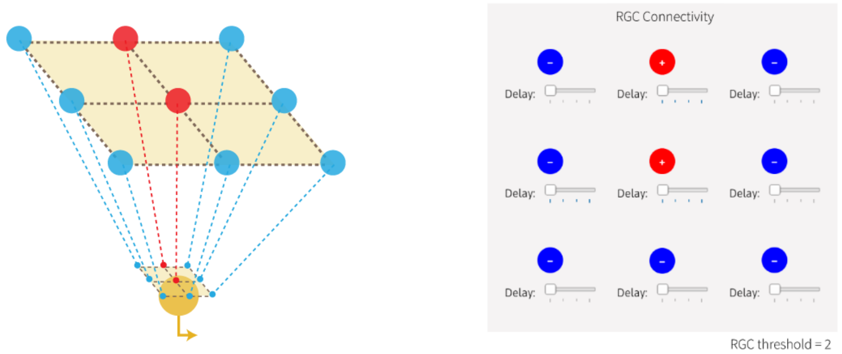

Lesson 2, Activity 5: Inputs & wiring to RGC2 (end-stopped vertical orientation selective cell)

Challenge #1: build a shape detector with orientation selective receptive fields

Please note that the solution below represents one of several possible correct solutions. You could have chosen any potential shape combining two straight lines of any orientation (for example, an X, a T, or an L - in any orientation). The below solution will configure a shape detector responsive to an '+' shape.

Each of the ganglion cells should be responsive to a line in a different position/orientation. They should both fire when the shape is presented (i.e. each ganglion cell must not be silenced when the other ganglion cell is active).

Inputs to Ganglion Cell 1: To make a cross shape, we would like Ganglion Cell 1 to be responsive to a vertical line in the middle of the photoreceptor array. Connect 3 adjacent photoreceptors (e.g. a vertical line) to a ganglion cell with a positive (excitatory, +) polarity, so that it fires only when all 3 (+) photoreceptors are activated (i.e., when a vertical bar of light is present in the center of the array). The cells in the corner of the array should be inhibitory (-), while the two left and right cells in the center row should be inactivated (grey). This will make the cell orientation selective but also continue to respond when Ganglion Cell 2 (see below) is activated.

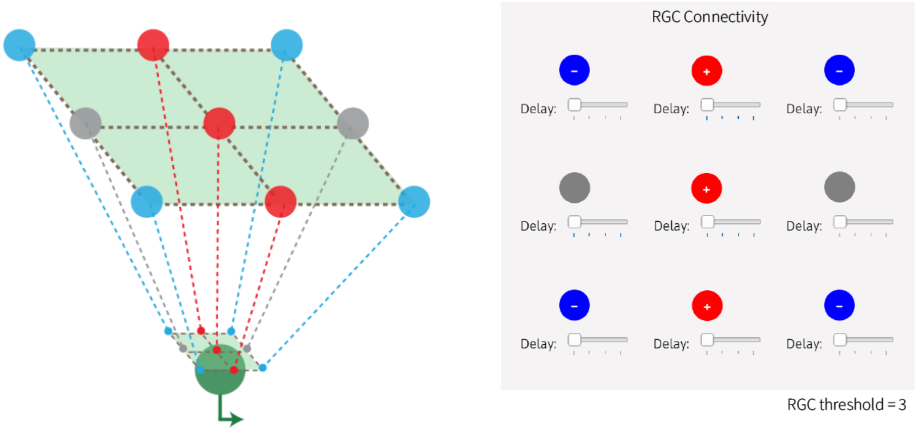

Lesson 2, Challenge 1: Inputs & wiring to RGC1 (*vertical orientation selective cell)*

Inputs to Ganglion Cell 2: To make a cross shape, we would like Ganglion Cell 2 to be responsive to a horizontal line in the middle of the photoreceptor array. Connect 3 adjacent photoreceptors (e.g. a horizontal line) to the second ganglion cell with a positive (excitatory, +) polarity, so that it fires only when all 3 photoreceptors are activated (i.e., when a horizontal bar of light is present in the center of the array). Similar to above, adding inhibition to the corner photoreceptor connections (negative polarity, -) while disconnecting the top and bottom cells in the middle column will make the cell orientation selective, but still allow it to respond when RGC1 is active.

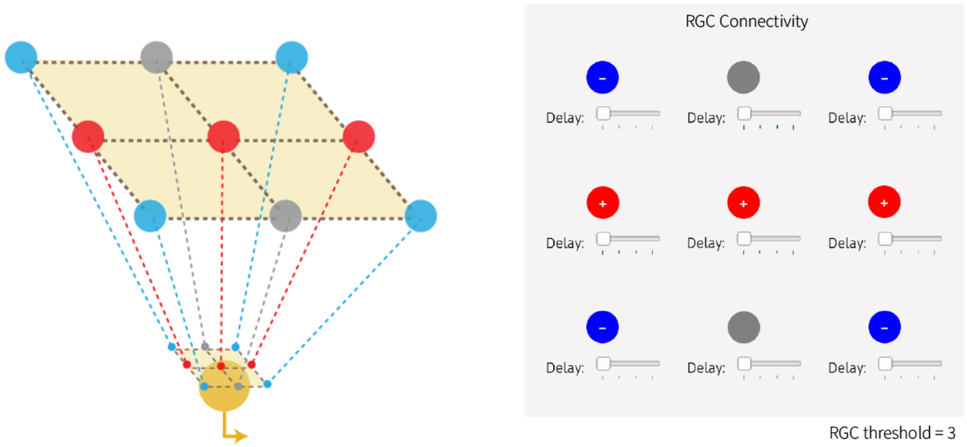

Lesson 2, Challenge 1: Inputs & wiring to RGC2 (horizontal orientation selective cell)

Lesson 3 Solutions: Direction Selectivity

Note: Depending on the time delays you've assigned to each photoreceptor, you may need to test different stimulus speeds to observe the ganglion cell firing specifically in one direction. The circuit will work most robustly when the speed of motion is tailored to the timing of the delays, allowing all preferred-direction inputs to summate at the ganglion cell simultaneously during preferred-direction motion, and allowing null-direction inhibition to effectively cancel excitation during null-direction motion.

Activity #1: build a left-moving direction selective ganglion cell ←

The ganglion cell receives input from two full columns of excitatory photoreceptors (positive polarity) and one full column of inhibitory photoreceptors (negative polarity).

- Left-most photoreceptor column (inhibitory, -): short time delay
- Middle photoreceptor column (excitatory, +): No time delay
- Right-most photoreceptor column (excitatory, +): short time delay

Set the **ganglion cell threshold to 6 and the cell type to ON.** This ensures that the cell will only fire when both columns of excitatory photoreceptors (6 photoreceptors in total) are activated in sync, i.e., when the direction of motion is leftwards, and the timing aligns perfectly for input summation.

This configuration causes the ganglion cell to fire only when a stimulus moves leftward across the array. The stimulus sequentially activates the rightmost, middle, and finally left column photoreceptors. The delays are configured so that the signals from the two excitatory columns arrive at the ganglion cell simultaneously **only** during leftward motion, allowing the cell to reach threshold and fire. The inhibitory column's input, being delayed, arrives too late to interfere with the ganglion cell's response. If the stimulus moves rightward (i.e., the opposite direction to the ganglion cell's preferred direction), the inhibitory column will summate with the middle excitatory column, cancelling out excitation and preventing the ganglion cell from firing. Furthermore, the two columns of excitatory inputs will be activated out of phase with one another for rightward motion. **note that if you simply stimulate the 6 positively connected photoreceptors (while not stimulating the 3 negatively connected photoreceptors) with a static stimulus, the ganglion cell will eventually turn on as well (similar to what happens for many direction selective ganglion cells in the brain which can sometimes be activated by both preferred direction moving and static stimuli).*

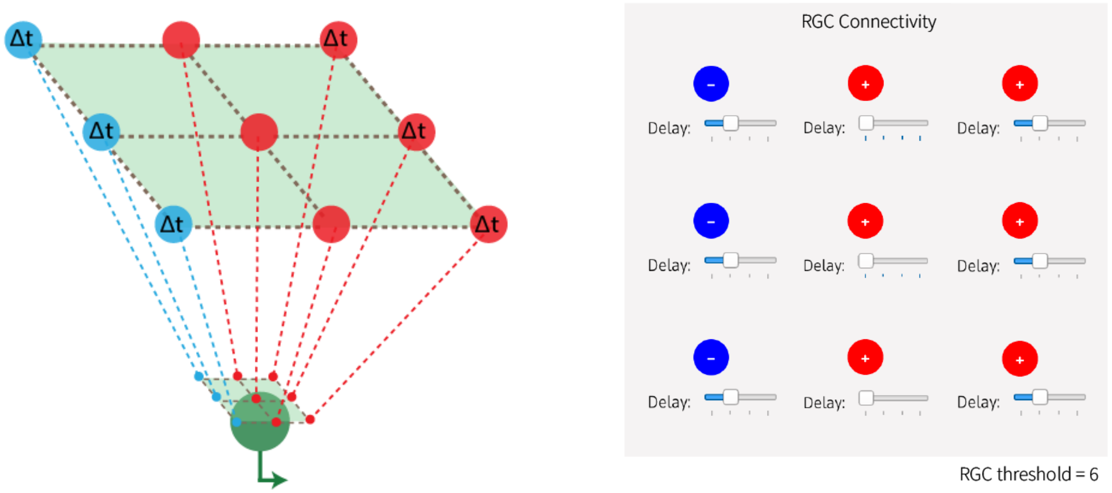

Lesson 3, Activity 1: Inputs & wiring to RGC1 (left-moving direction selective cell)

Activity #2: build a right-moving direction selective ganglion cell →

The ganglion cell receives input from two full columns of excitatory photoreceptors (positive polarity) and one full column of inhibitory photoreceptors (negative polarity).

- Left-most photoreceptor column (excitatory, +): short time delay
- Middle photoreceptor column (excitatory, +): No time delay
- Right-most photoreceptor column (inhibitory, -): short time delay

Set the **ganglion cell threshold to 6 and the cell type to ON.** This ensures that the cell will only fire when both columns of excitatory photoreceptors (6 photoreceptors in total) are activated in sync, i.e., when the direction of motion is rightwards, and the timing aligns perfectly for input summation. Please refer to *Activity #1 Solution* for a detailed explanation of time-aligned input summation.

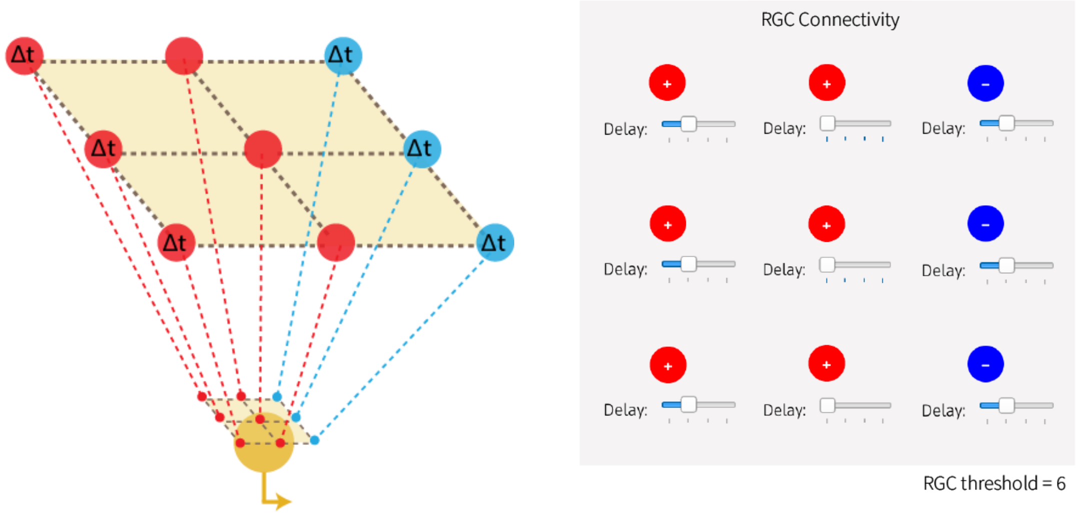

Lesson 3, Activity 2: Inputs & wiring to RGC2 (right-moving direction selective cell)

Activity #3: build a slow vs. fast motion preferring direction selective ganglion cell →

The key to solving this problem is the **magnitude of the time delays** imposed on the circuits seen in Activity #1 (right-direction selective cell).

Each ganglion cell receives input from two full columns of excitatory photoreceptors (positive polarity) and one full column of inhibitory photoreceptors (negative polarity). Set each **ganglion cell threshold to 6 and the cell type to ON.** This ensures that the cell will only fire when both columns of excitatory photoreceptors (6 photoreceptors in total) are activated in sync.

Inputs to RGC1 (slow-moving → directions-selective cell):

- Left-most photoreceptor column (excitatory, +): long time delay
- Middle photoreceptor column (excitatory, +): no time delay

- Right-most photoreceptor column (inhibitory, -): long time delay

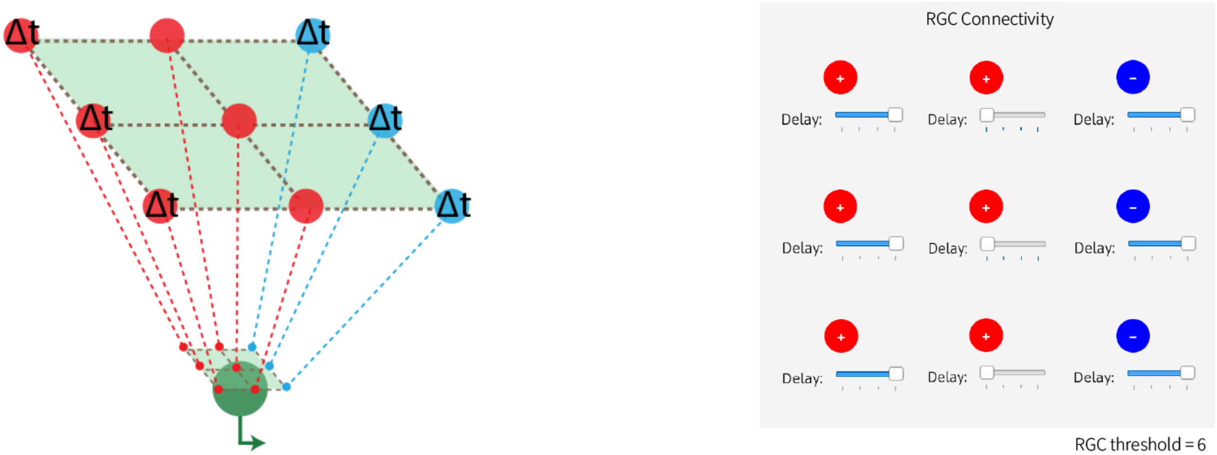

Lesson 3, Activity 3: Inputs & wiring to RGC1 (slow right-moving direction selective cell)

Inputs to RGC2 (fast-moving → directions-selective cell):

- Left-most photoreceptor column (excitatory, +): short time delay
- Middle photoreceptor column (excitatory, +): no time delay
- Right-most photoreceptor column (inhibitory, -): short time delay

A shorter time delay creates a smaller time window for inputs to summate, so the stimulus must move faster to activate the ganglion cell. Conversely, a longer time delay creates a wider summation window, allowing the cell to respond to slower motion. Please refer to *Activity #1 Solution* for a detailed explanation of time-aligned input summation.

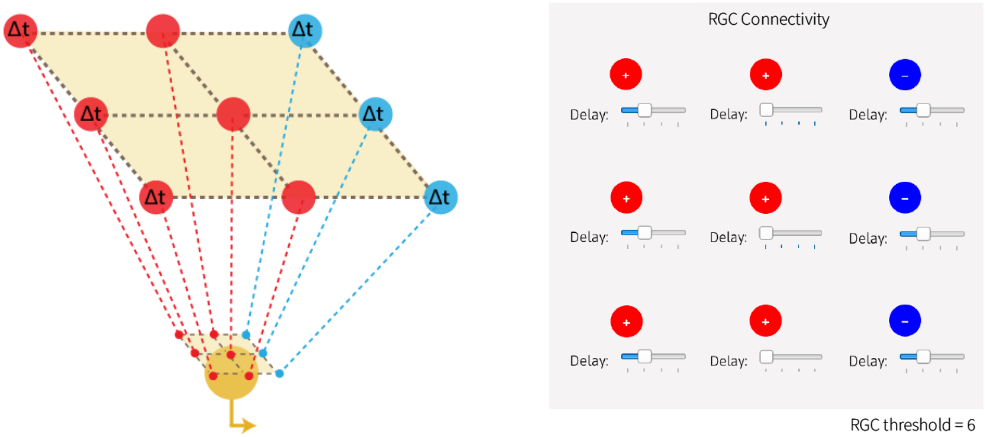

Lesson 3, Activity 3: Inputs & wiring to RGC2 (fast right-moving direction selective cell)

Challenge: Block breaking with direction selective circuits

Inputs to RGC1 ←: the ganglion cell receives input from two full columns of excitatory photoreceptors (positive polarity) and one full column of inhibitory photoreceptors (negative polarity). Set the **ganglion cell threshold to 6 and the cell type to ON.**

- Left-most photoreceptor column (inhibitory, -): short time delay
- Middle photoreceptor column (excitatory, +): No time delay
- Right-most photoreceptor column (excitatory, +): short time delay

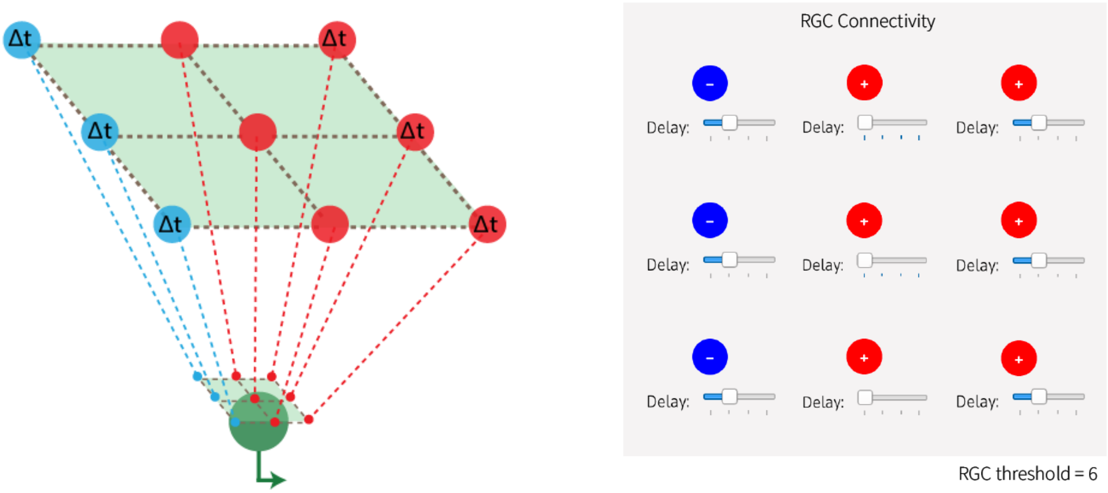

Lesson 3, Challenge 1: Inputs & wiring to RGC1 (left-moving direction selective cell)

Inputs to RGC2 → : the ganglion cell receives input from two full columns of excitatory photoreceptors (positive polarity) and one full column of inhibitory photoreceptors (negative polarity). Set the **ganglion cell threshold to 6 and the cell type to ON.**

- Left-most photoreceptor column (excitatory, +): short time delay
- Middle photoreceptor column (excitatory, +): No time delay
- Right-most photoreceptor column (inhibitory, -): short time delay

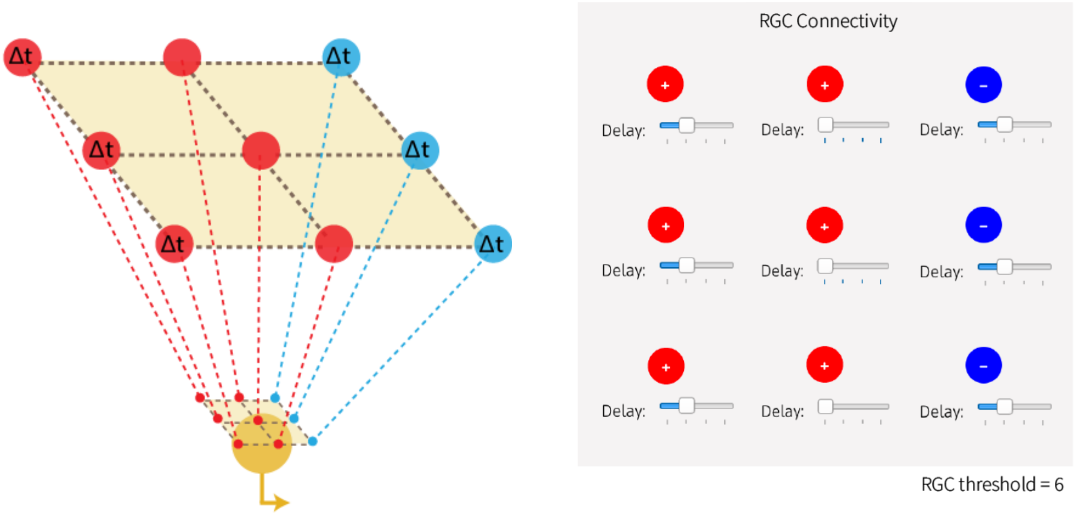

Lesson 3, Challenge 1: Inputs & wiring to RGC2 (right-moving direction selective cell)

Lesson 4 Solutions: Discovery Mode

Easy

Easy 1: Center-surround cell

- Preferred stimulus: static spot of light on the left edge of the visual field
- ON circuit:
    - Ganglion cell threshold: 1
    - Cell type: ON
- OFF circuit:
    - Ganglion cell threshold: 8
    - Cell type: OFF

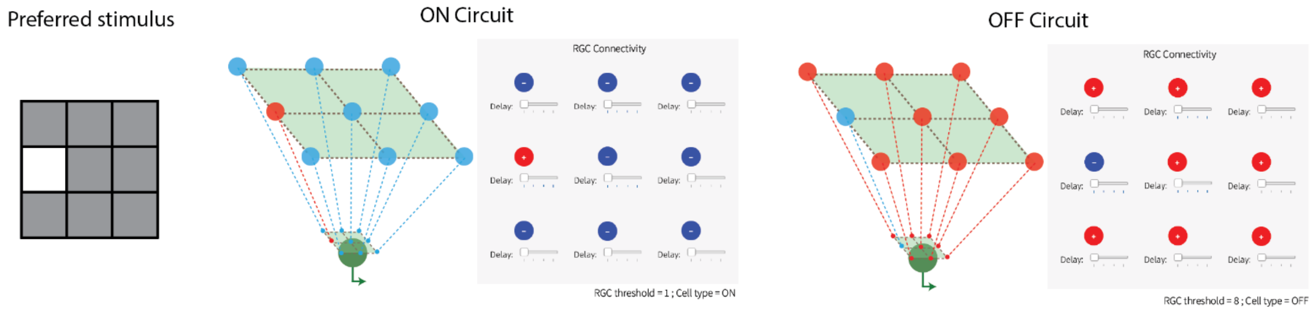

Lesson 4, Easy 1: Preferred stimulus (left) and wiring of ON circuit (middle) and OFF circuit (right) to Easy 1 mystery circuit

Easy 2: Orientation selective cell

- Preferred stimulus: static diagonal line (135°) in the center of the visual field
- ON circuit:
    - Ganglion cell threshold: 3
    - Cell type: ON
- OFF circuit:
    - Ganglion cell threshold: 6
    - Cell type: OFF

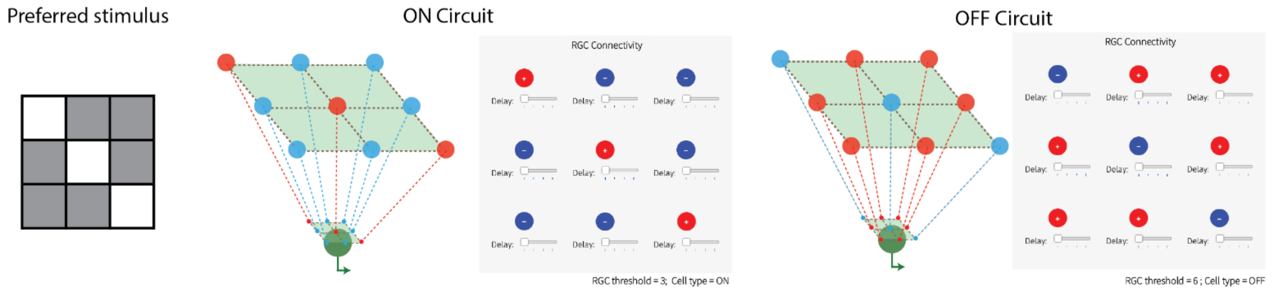

Lesson 4, Easy 2: Preferred stimulus (left) and wiring of ON circuit (middle) and OFF circuit (right) to Easy 2 mystery circuit

Easy 3: Orientation selective cell

- Preferred stimulus: static horizontal line in the center of the visual field
- ON circuit:
    - Ganglion cell threshold: 3
    - Cell type: ON
- OFF circuit:
    - Ganglion cell threshold: 6
    - Cell type: OFF

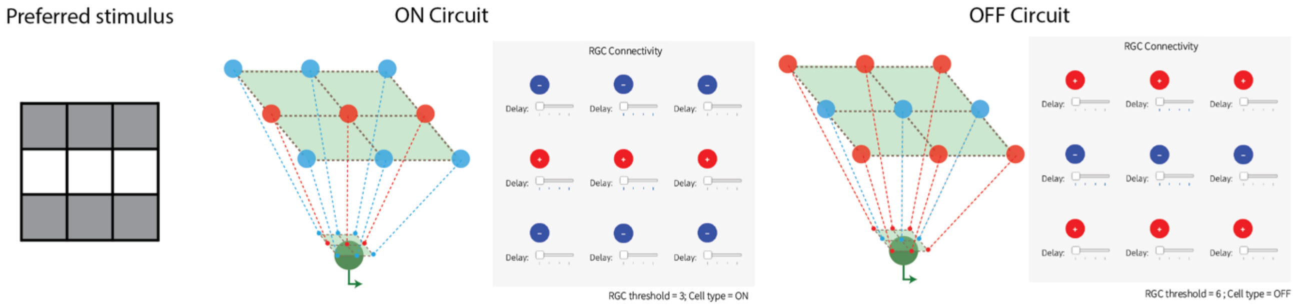

Lesson 4, Easy 3: Preferred stimulus (left) and wiring of ON circuit (middle) and OFF circuit (right) to Easy 3 mystery circuit

<u>*Easy 4:* Center-surround cell</u>

- Preferred stimulus: static spot of light on the top right corner of the visual field
- ON circuit:
    - Ganglion cell threshold: 1
    - Cell type: ON
- OFF circuit:
    - Ganglion cell threshold: 8
    - Cell type: OFF

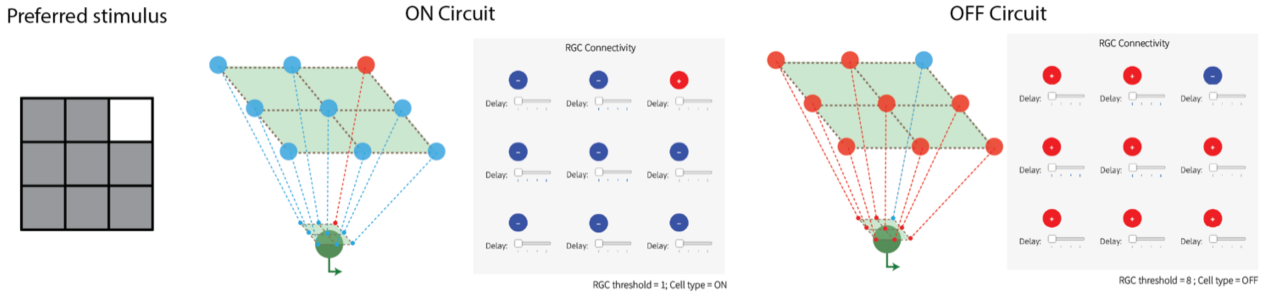

Lesson 4, Easy 4: Preferred stimulus (left) and wiring of ON circuit (middle) and OFF circuit (right) to Easy 4 mystery circuit

<u>*Easy 5:* Orientation selective cell</u>

- Preferred stimulus: static vertical line on the right edge of the visual field
- ON circuit:
    - Ganglion cell threshold: 3
    - Cell type: ON
- OFF circuit:
    - Ganglion cell threshold: 6

o Cell type: OFF

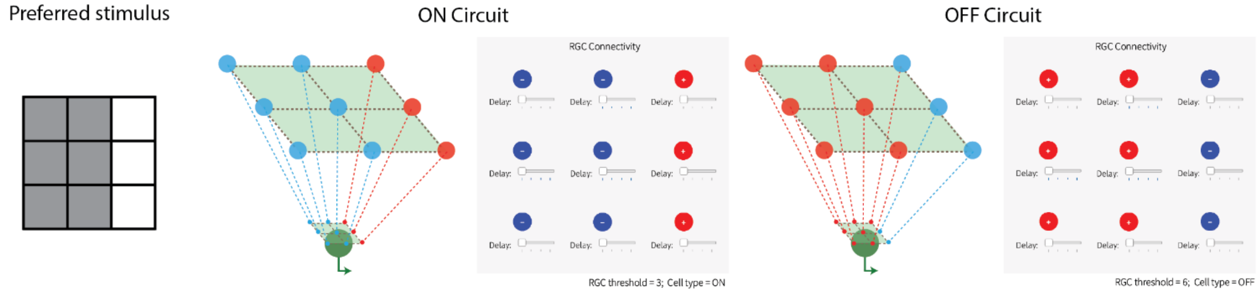

Lesson 4, Easy 5: Preferred stimulus (left) and wiring of ON circuit (middle) and OFF circuit (right) to Easy 5 mystery circuit

<u>*Easy 6:* Orientation selective cell</u>

- Preferred stimulus: static diagonal line (45°) in the center of the visual field
- ON circuit:
  - o Ganglion cell threshold: 3
  - o Cell type: ON
- OFF circuit:
  - o Ganglion cell threshold: 6
  - o Cell type: OFF

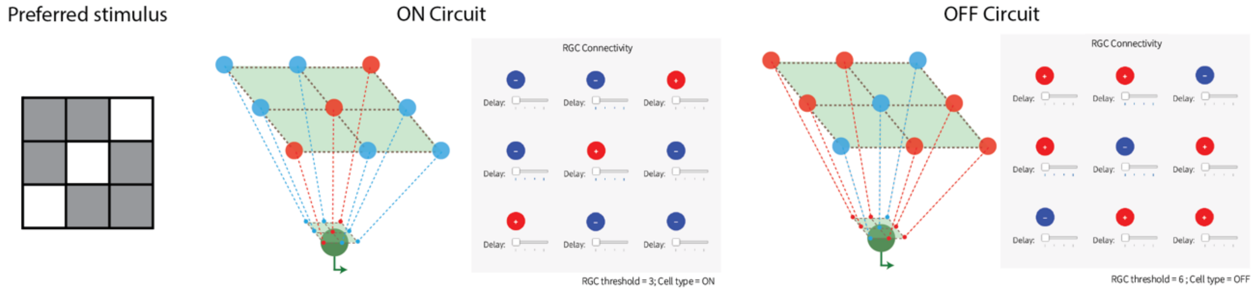

Lesson 4, Easy 6: Preferred stimulus (left) and wiring of ON circuit (middle) and OFF circuit (right) to Easy 6 mystery circuit

<u>*Easy 7:* Orientation selective cell</u>

- Preferred stimulus: static vertical line on the left edge of the visual field
- ON circuit:
  - o Ganglion cell threshold: 3
  - o Cell type: ON
- OFF circuit:
  - o Ganglion cell threshold: 6
  - o Cell type: OFF

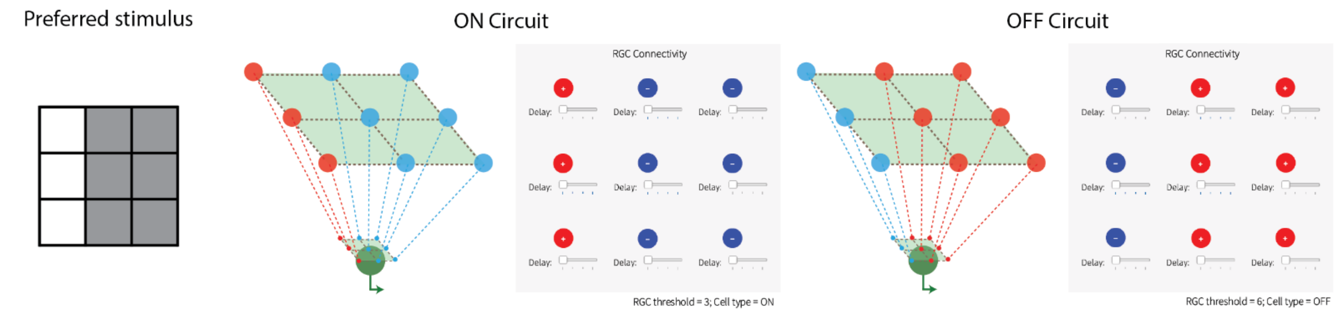

Lesson 4, Easy 7: Preferred stimulus (left) and wiring of ON circuit (middle) and OFF circuit (right) to Easy 7 mystery circuit

Medium

<u>*Medium 1: Ring-selective cell*</u>

- Preferred stimulus: ring of light (no light in the center of the visual field)
- ON circuit:
    - Ganglion cell threshold: 8
    - Cell type: ON
- OFF circuit:
    - Ganglion cell threshold: 1
    - Cell type: OFF

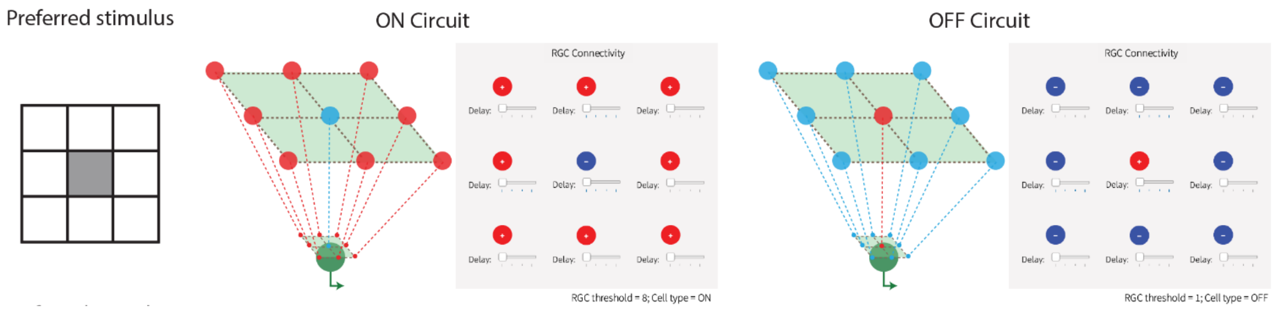

Lesson 4, Medium 1: Preferred stimulus (left) and wiring of ON circuit (middle) and OFF circuit (right) to Medium 1 mystery circuit

<u>*Medium 2:* Shape (T) selective cell</u>

- Preferred stimulus: T-shape
- ON circuit:
    - Ganglion cell threshold: 5
    - Cell type: ON
- OFF circuit:
    - Ganglion cell threshold: 4
    - Cell type: OFF
-

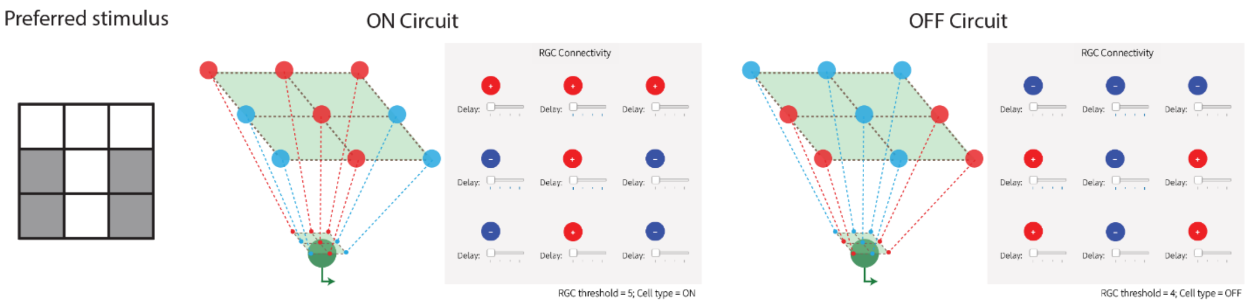

Lesson 4, Medium 2: Preferred stimulus (left) and wiring of ON circuit (middle) and OFF circuit (right) to Medium 2 mystery circuit

<u>*Medium 3:* Shape (X) selective cell</u>

- Preferred stimulus: X-shape in the center of the visual field
- ON circuit:
    - Ganglion cell threshold: 5
    - Cell type: ON
- OFF circuit:
    - Ganglion cell threshold: 4
    - Cell type: OFF
-

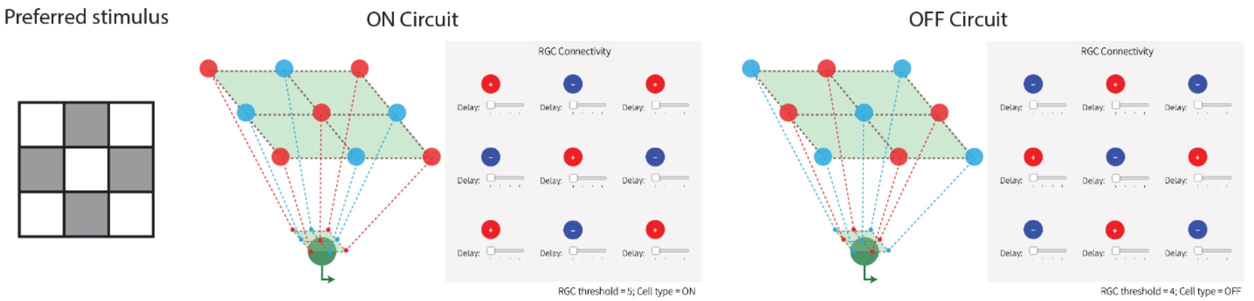

Lesson 4, Medium 3: Preferred stimulus (left) and wiring of ON circuit (middle) and OFF circuit (right) to Medium 3 mystery circuit

<u>*Medium 4:* Shape (+) selective</u>

- Preferred stimulus: + - shape (or a medium-sized spot of light) in the center of the visual field
- ON circuit:
    - Ganglion cell threshold: 5
    - Cell type: ON
- OFF circuit:
    - Ganglion cell threshold: 4
    - Cell type: OFF
-

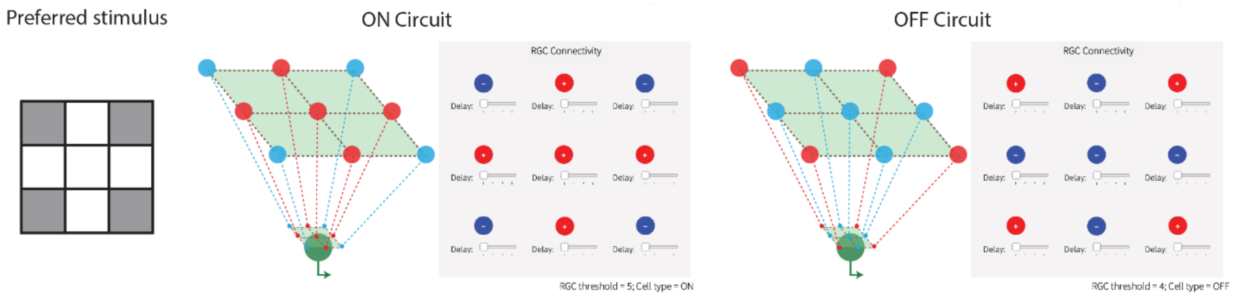

Lesson 4, Medium 4: Preferred stimulus (left) and wiring of ON circuit (middle) and OFF circuit (right) to Medium 4 mystery circuit

Medium 5: <u>Center-surround cell</u>

- Preferred stimulus: Medium-sized spot of light with a bottom-left receptive field
- ON circuit:
  - Ganglion cell threshold: 4
  - Cell type: ON
- OFF circuit:
  - Ganglion cell threshold: 5
  - Cell type: OFF

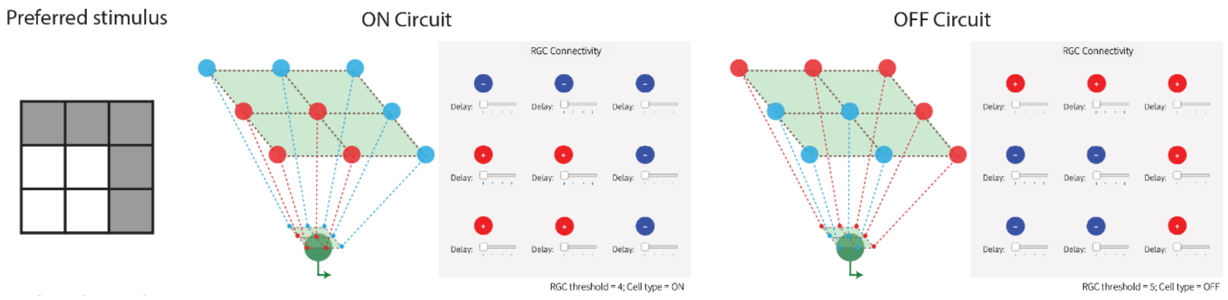

Lesson 4, Medium 5: Preferred stimulus (left) and wiring of ON circuit (middle) and OFF circuit (right) to Medium 5 mystery circuit

Medium 6: <u>Shape (C) selective cell</u>

- Preferred stimulus: C-shape
- ON circuit:
  - Ganglion cell threshold: 7
  - Cell type: ON
- OFF circuit:
  - Ganglion cell threshold: 2
  - Cell type: OFF

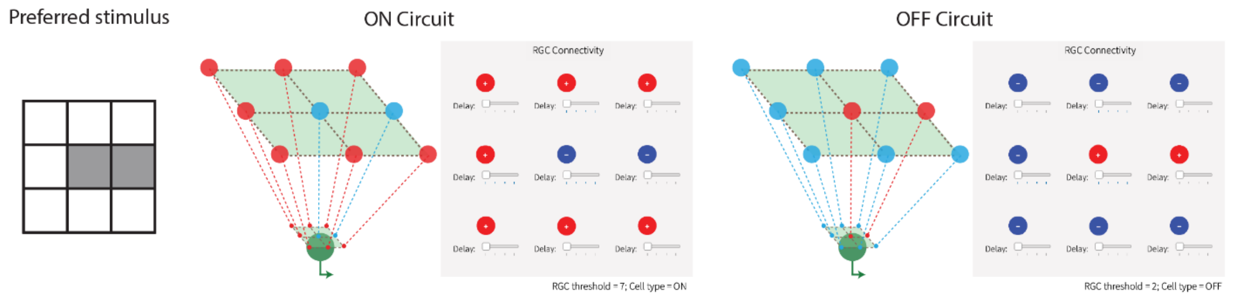

Lesson 4, Medium 6: Preferred stimulus (left) and wiring of ON circuit (middle) and OFF circuit (right) to Medium 6 mystery circuit

Hard

<u>*Hard 1:* Shape-selective cell (triangle)</u>

- Preferred stimulus: right-angle in the bottom-left of the visual field
- ON circuit:
  - Ganglion cell threshold: 6
  - Cell type: ON
- OFF circuit:
  - Ganglion cell threshold: 3
  - Cell type: OFF

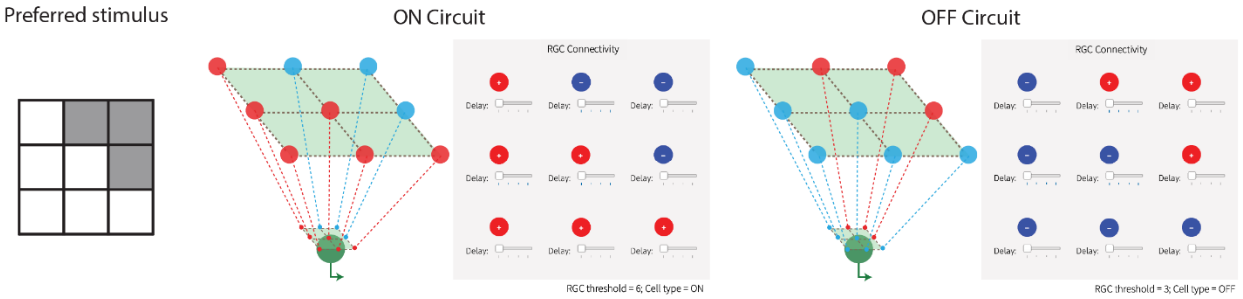

Lesson 4, Hard 1: Preferred stimulus (left) and wiring of ON circuit (middle) and OFF circuit (right) to Hard 1 mystery circuit

<u>*Hard 2:* Direction selective cell</u>

- Preferred stimulus: rightward-moving vertical bar of light
- Photoreceptors in the left and right columns should be configured with medium time delays (please see *Lesson 3, Activity #1 Solution* for a detailed explanation of time-aligned input summation).
- ON circuit:
  - Ganglion cell threshold: 6
  - Cell type: ON

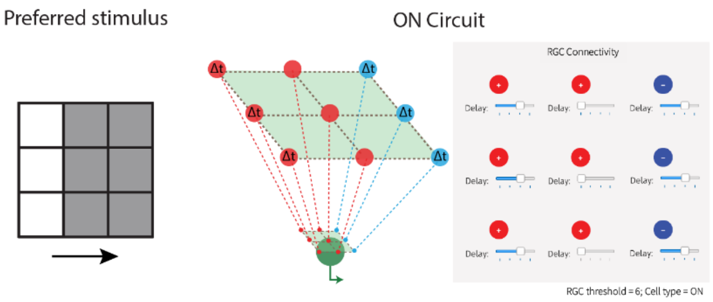

*Lesson 4, Hard 2: Preferred stimulus (left) and wiring of ON circuit (right) to Hard 2 mystery circuit. *Note: because the speed selectivity of RetINaBox direction selective cells is broad, we accept any time delay input as correct in Discovery mode direction selective challenges, but the preferred speed you select in Phase 1 needs to match the delay you select in Phase 2.*

<u>*Hard 3:* Shape (diamond) selective cell</u>

- Preferred stimulus: medium-sized ring of light / diamond (excludes the center)
- ON circuit:
  - Ganglion cell threshold: 4
  - Cell type: ON
- OFF circuit:
  - Ganglion cell threshold: 5
  - Cell type: OFF

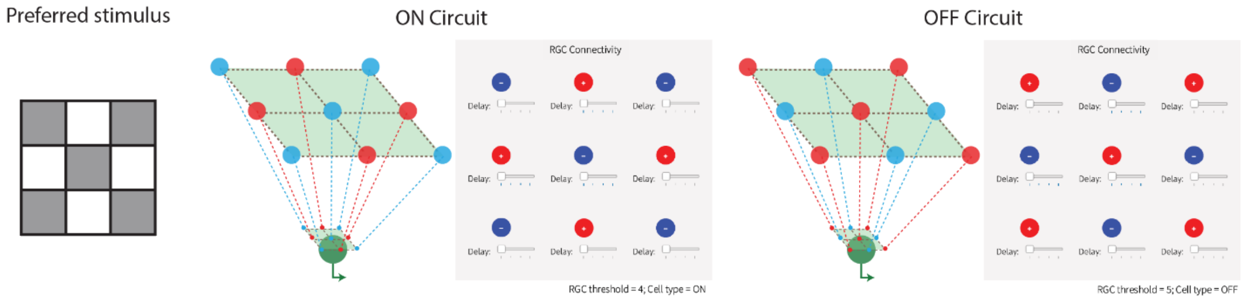

Lesson 4, Hard 3: Preferred stimulus (left) and wiring of ON circuit (middle) and OFF circuit (right) to Hard 3 mystery circuit

<u>*Hard 4:* Direction selective cell</u>

- Preferred stimulus: leftward-moving vertical bar of light
- Photoreceptors in the left and right columns should be configured with a medium time delay (please see *Lesson 3, Activity #3* for a detailed explanation of time-aligned input summation).
- ON circuit:
  - Ganglion cell threshold: 6
  - Cell type: ON

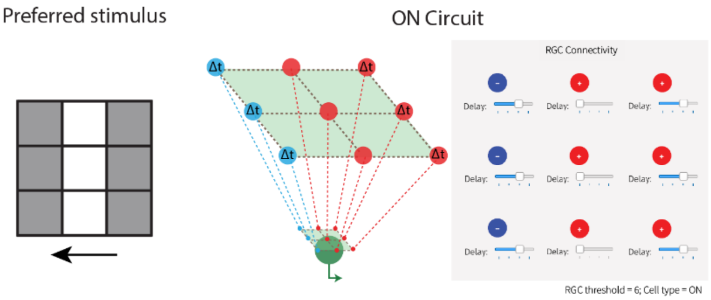

*Lesson 4, Hard 4: Preferred stimulus (left) and wiring of ON circuit (right) to Hard 4 Mystery Circuit. *Note: because the speed selectivity of RetINaBox direction selective cells is broad, we accept any time delay input as correct in Discovery mode direction selective challenges, but the preferred speed you select in Phase 1 needs to match the delay you select in Phase 2.*
