# Supplementary material for: RetINaBox: A Hands-On Learning Tool for Experimental Neuroscience
Source: eNeuro. 2026 Jan 2;13(1):ENEURO.0349-25.2025. doi: 10.1523/ENEURO.0349-25.2025 (PMC12813302; doi:10.1523/ENEURO.0349-25.2025)
Supplement: Data 1 — Download Data 1, ZIP file. [file eneuro-13-ENEURO.0349-25.2025-s003.zip › RetINaBox_TeachingSlides.pdf]

RetINaBox

Lesson Plans

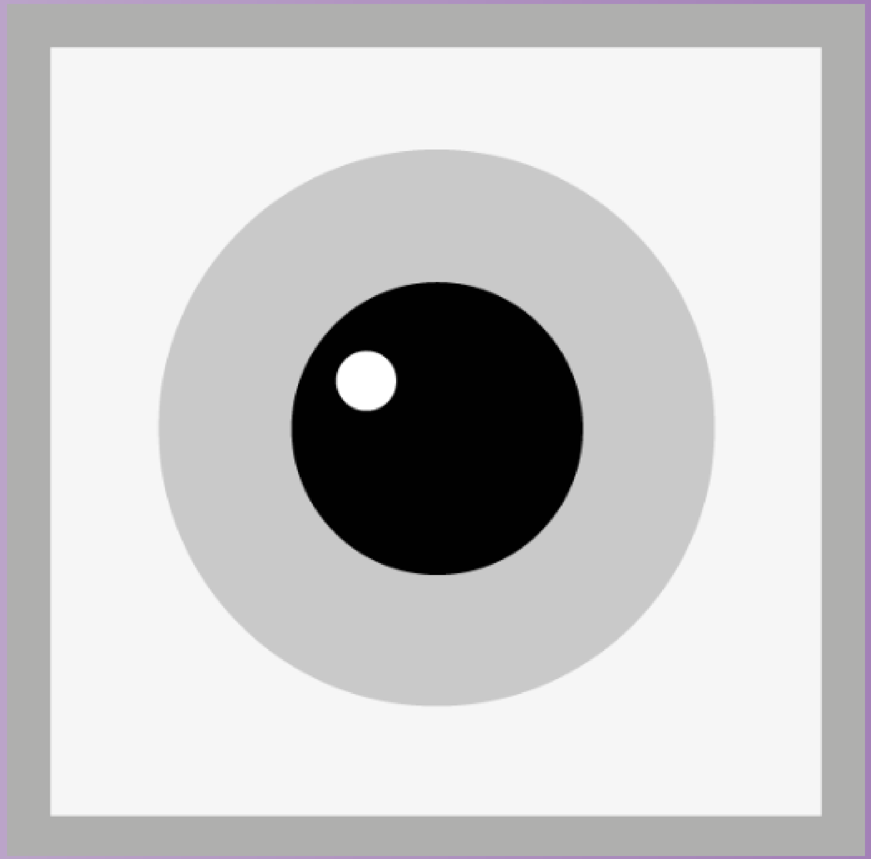

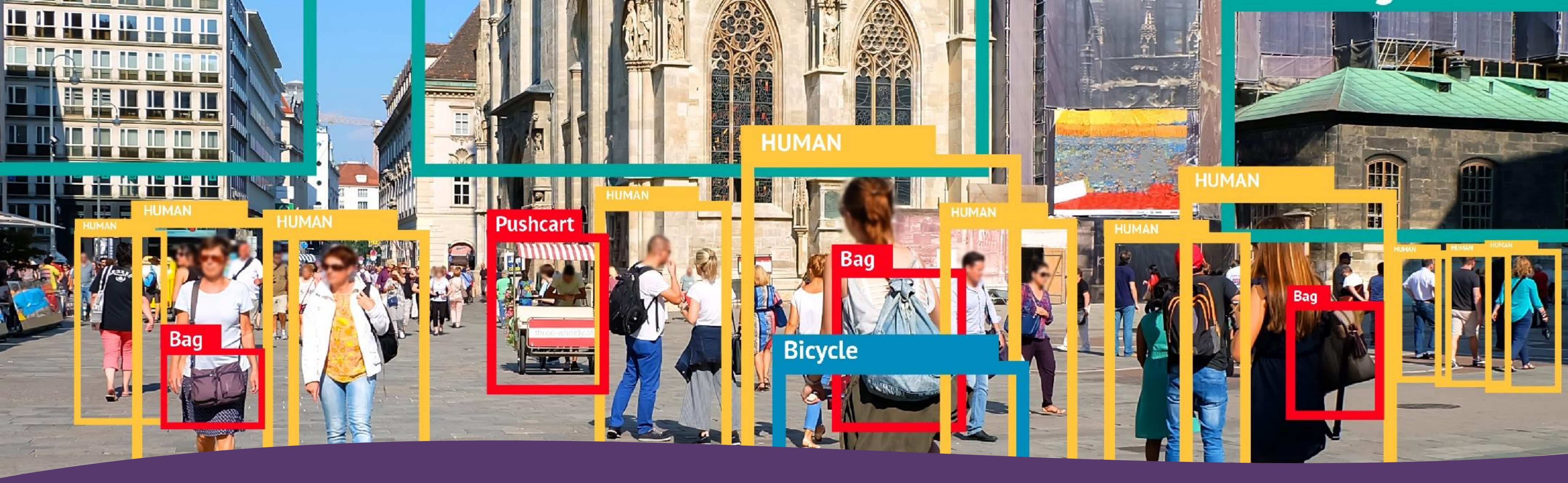

The visual world is made up of things in places

Our brain has developed ways of knowing what *it's looking at and* where *things are*

The brain breaks down the visual world with feature detectors

Retina

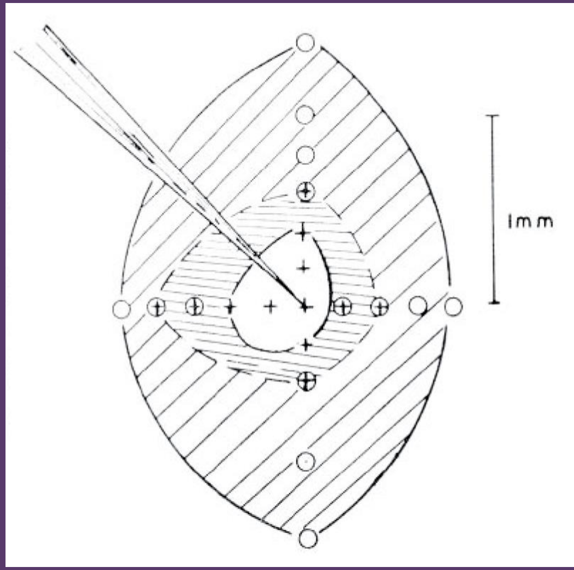

Kuffler, 1953

Visual Cortex

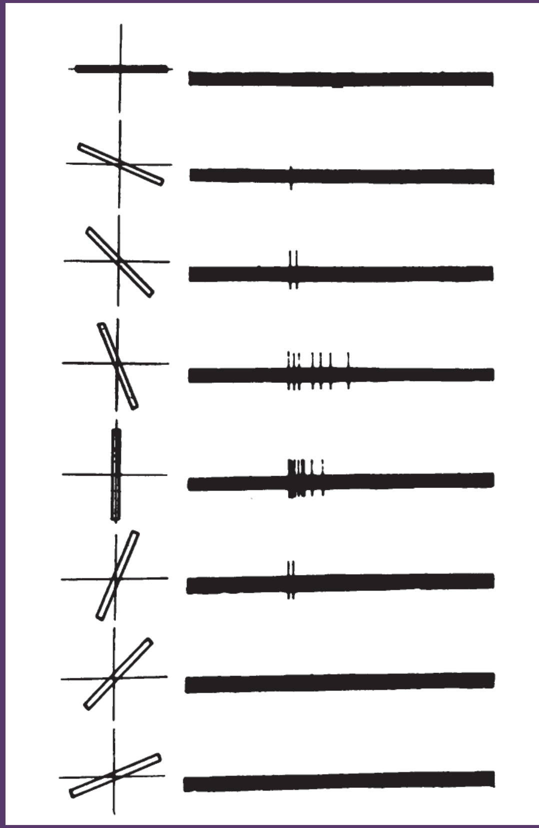

Hubel and Wiesel, 1959

Temporal Lobe

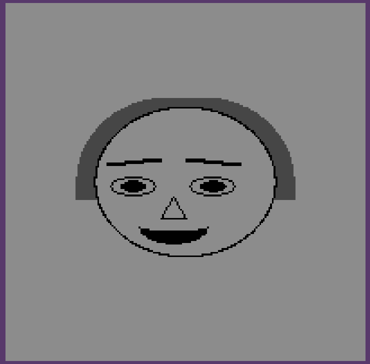

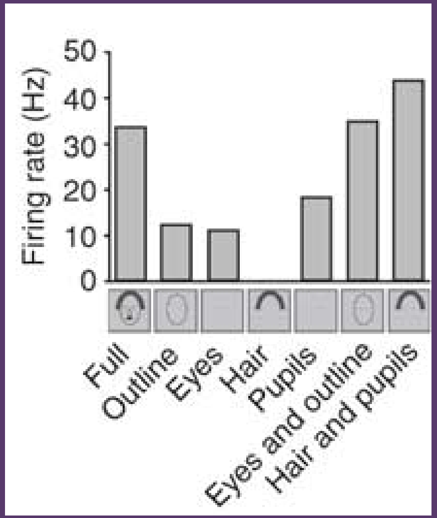

Freiwald et al., 2009

Seeing starts in the eye

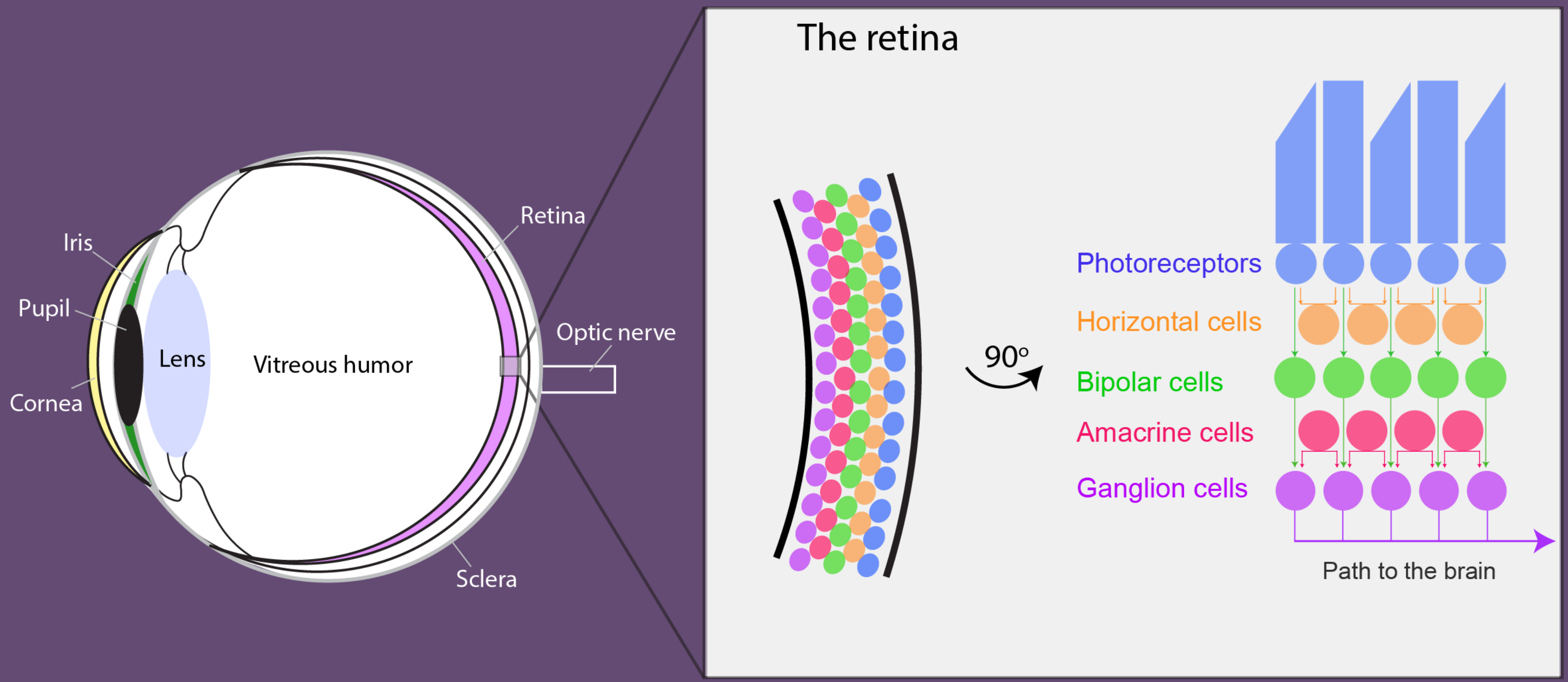

The retina

Photoreceptors are arranged in an array in the retina.

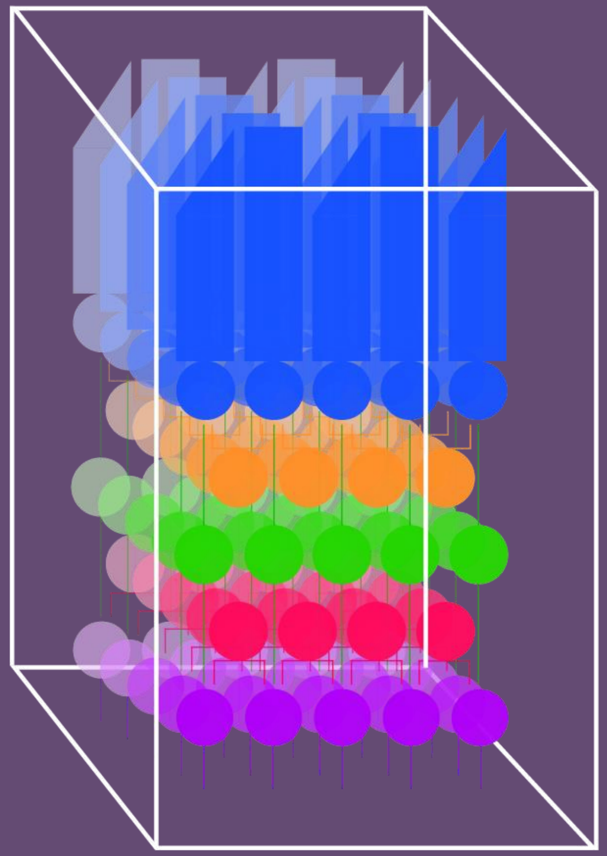

RetINaBox is a simplified model of the visual system

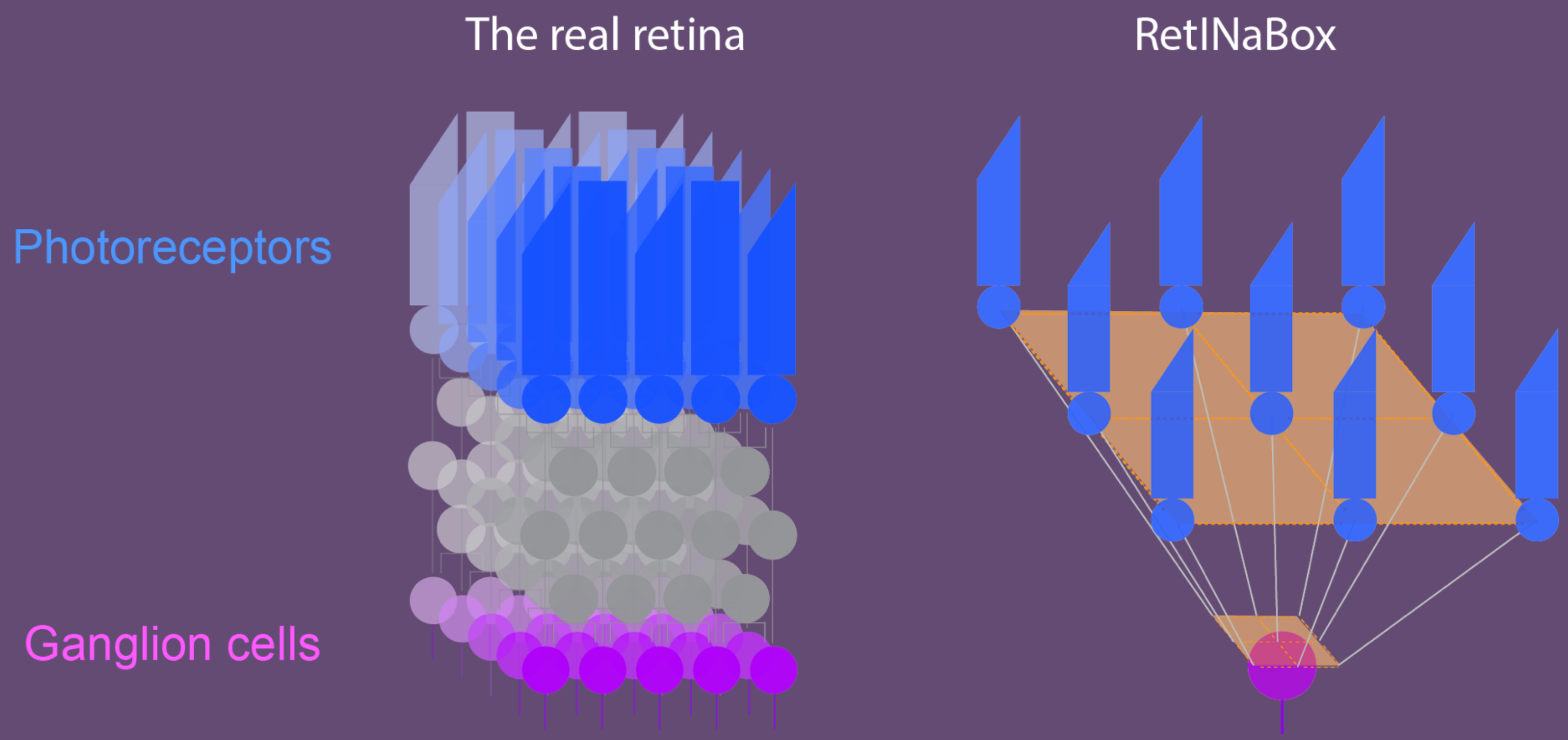

*Note that while in RetINaBox, photoreceptors directly connect to RGCs, this does not happen in the real retina. In RetINaBox, the contribution from other retinal neurons (such as horizontal cells, bipolar cells and amacrine cells) to RGC responses are included in sign, delay and ON/OFF transfer functions.

Today we'll use RetINaBox to explore how the brain generates visual feature detectors

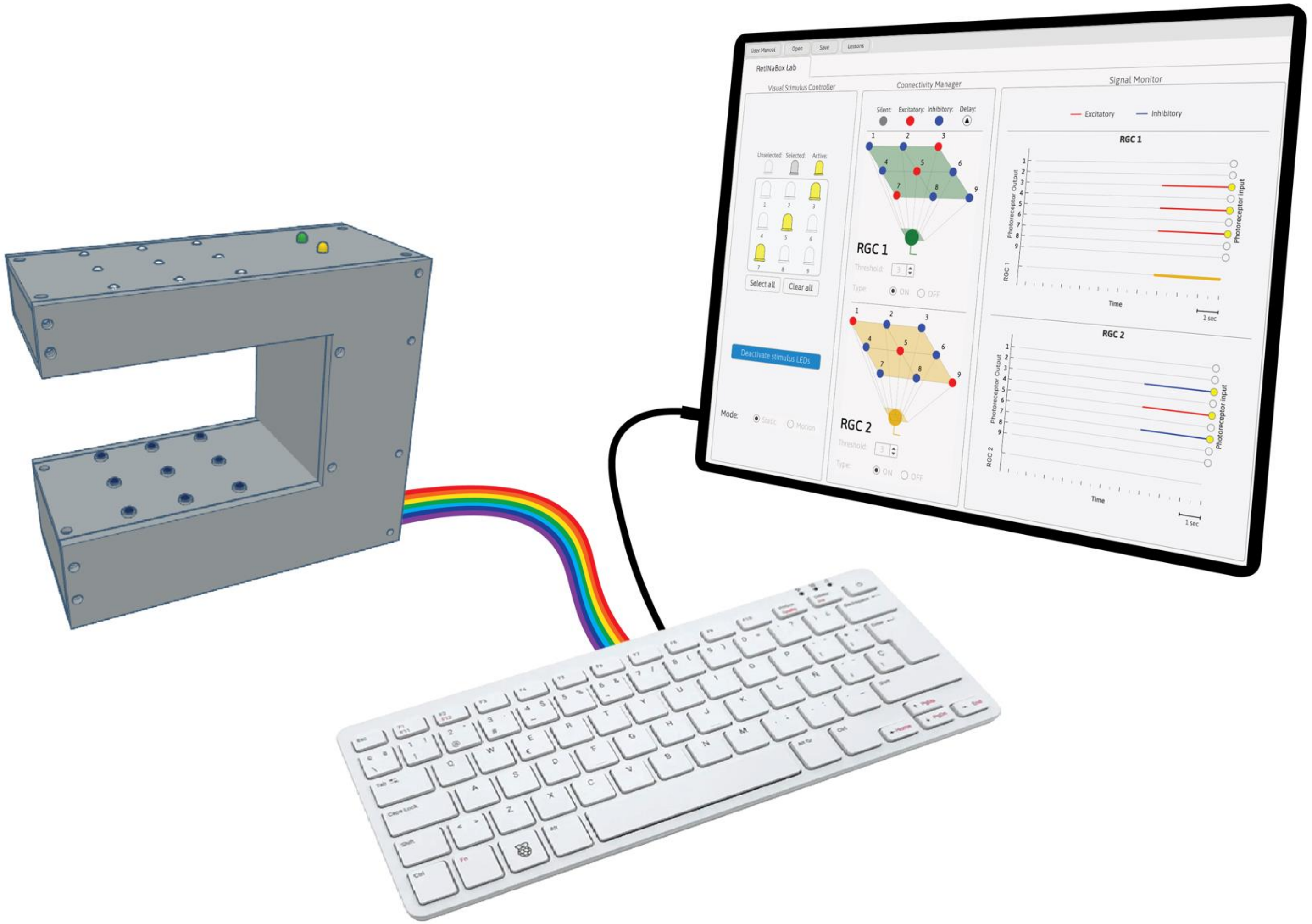

RetINaBox

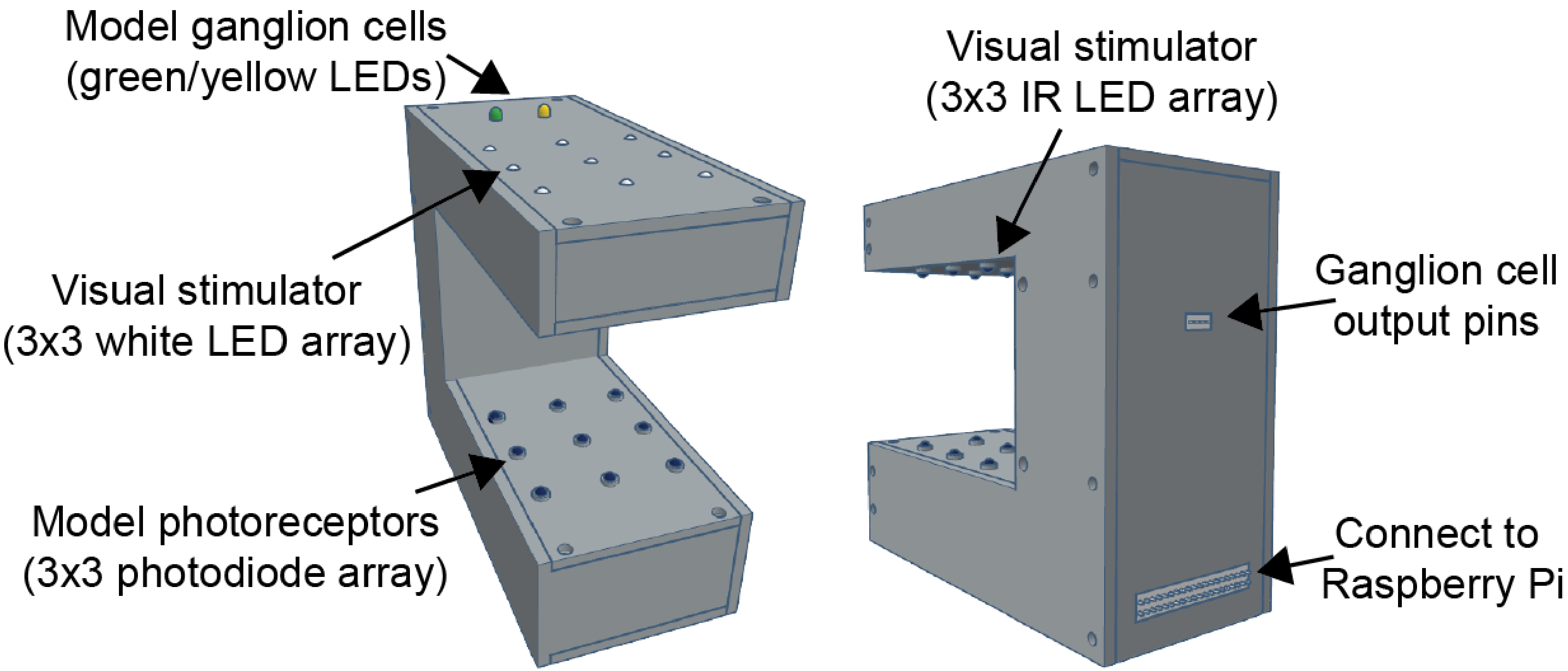

Model ganglion cells
(green/yellow LEDs)
Visual stimulator
(3x3 white LED array)
Model photoreceptors
(3x3 photodiode array)
Visual stimulator
(3x3 IR LED array)
Ganglion cell
output pins
Connect to
Raspberry Pi

RetINaBox

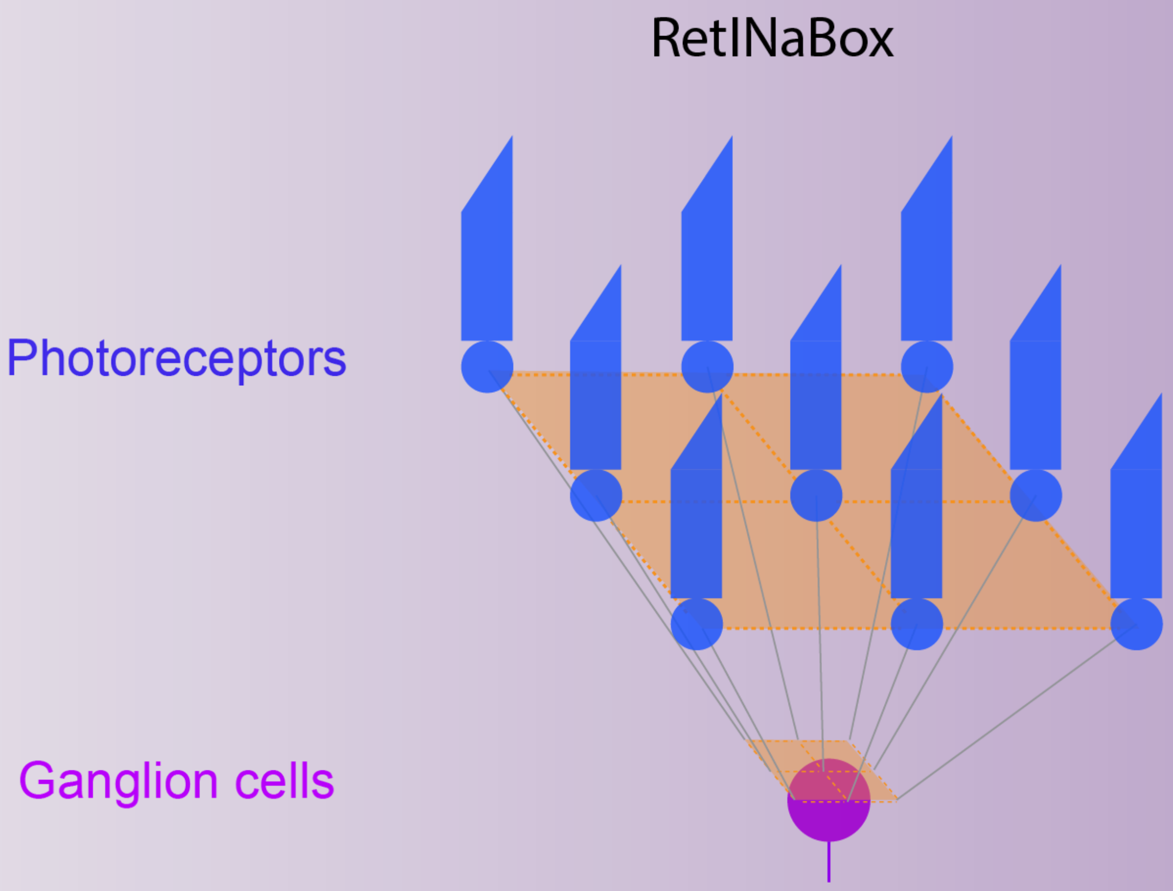

Each ganglion cell responds when it receives positive inputs from a sufficient number of photoreceptors.

Each photoreceptor can connect to either or both ganglion cells.

RetINaBox

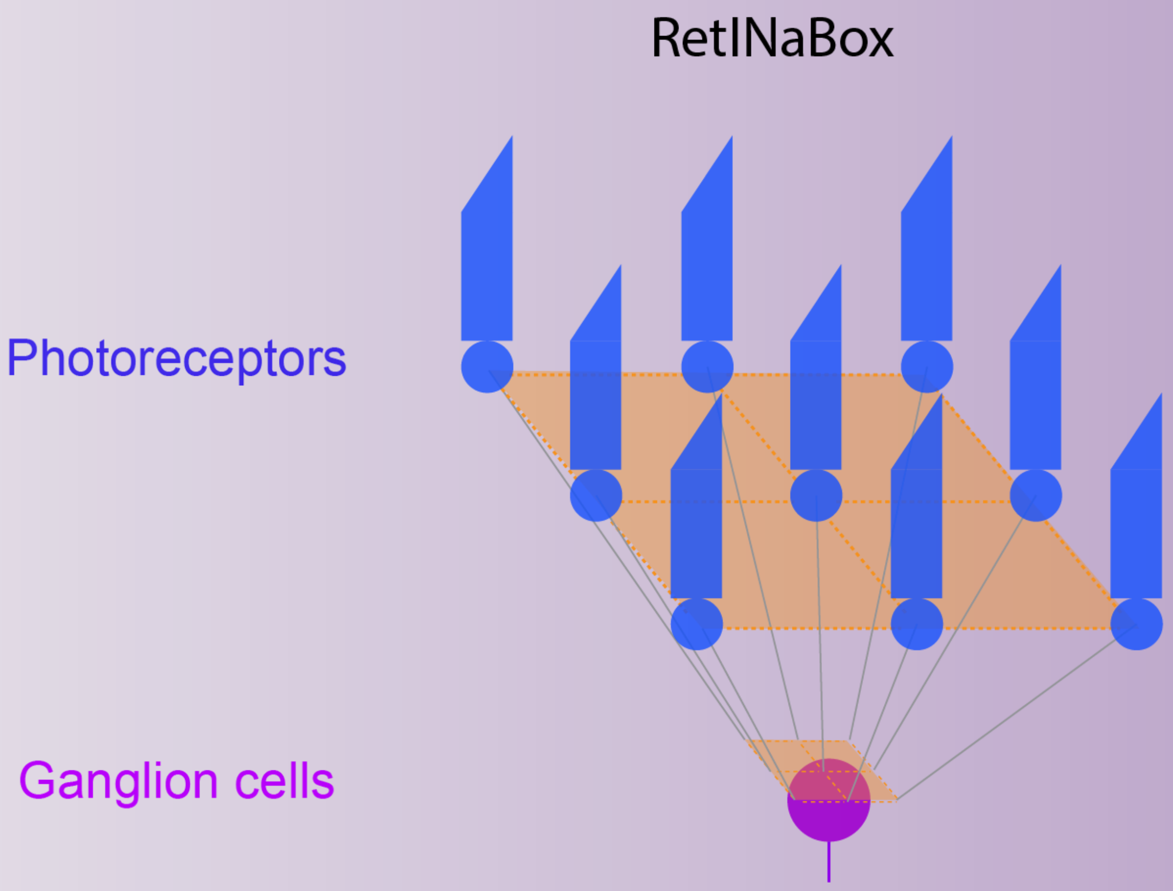

For each photoreceptor, you get to set:

- Polarity (+/-)
- Time delay

For each ganglion cell, you get to decide:

- Threshold
- Type: ON or OFF

RetINaBox Graphical User Inerface (GUI)

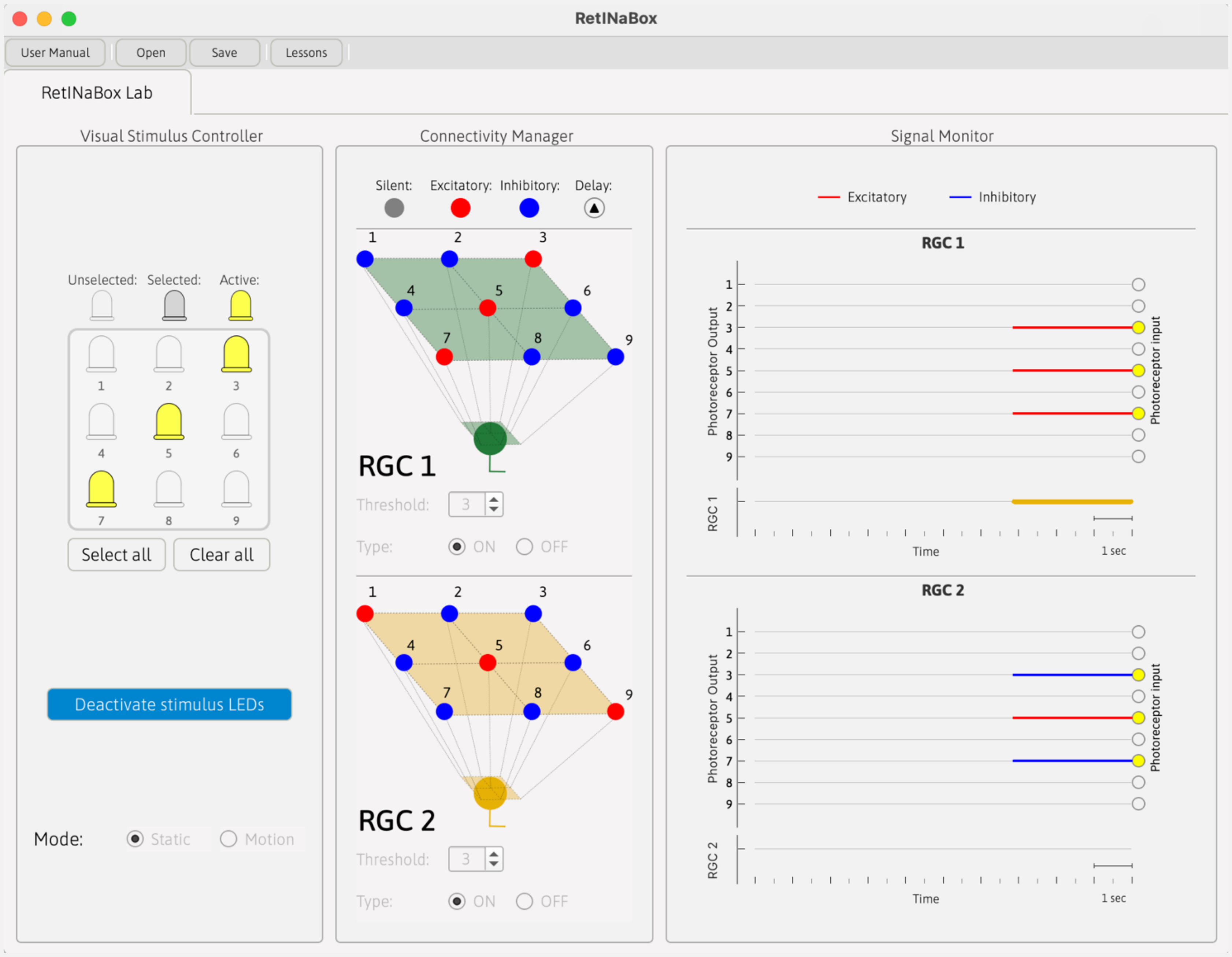

Visual Stimulus Controller

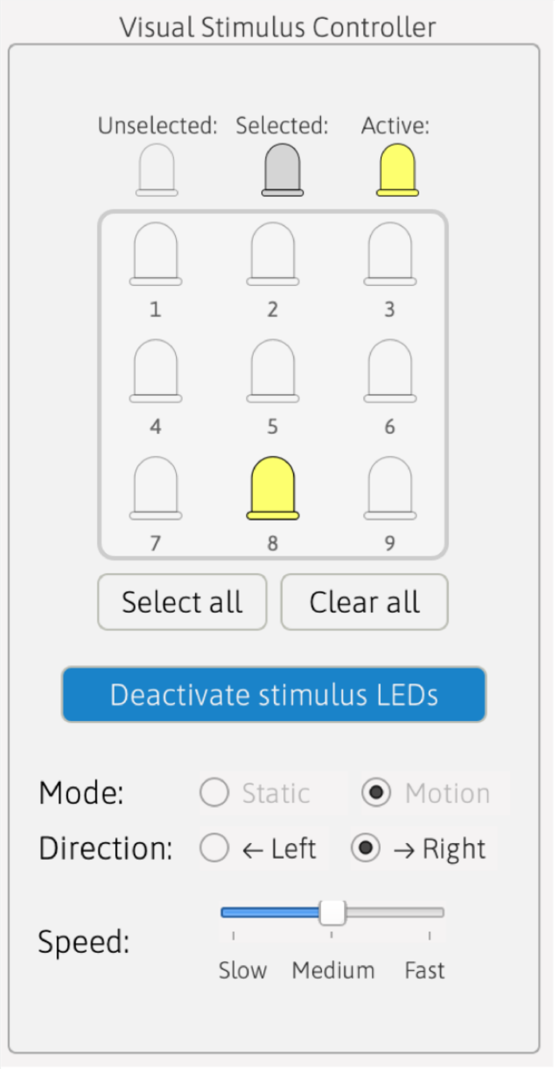

Connectivity Manager

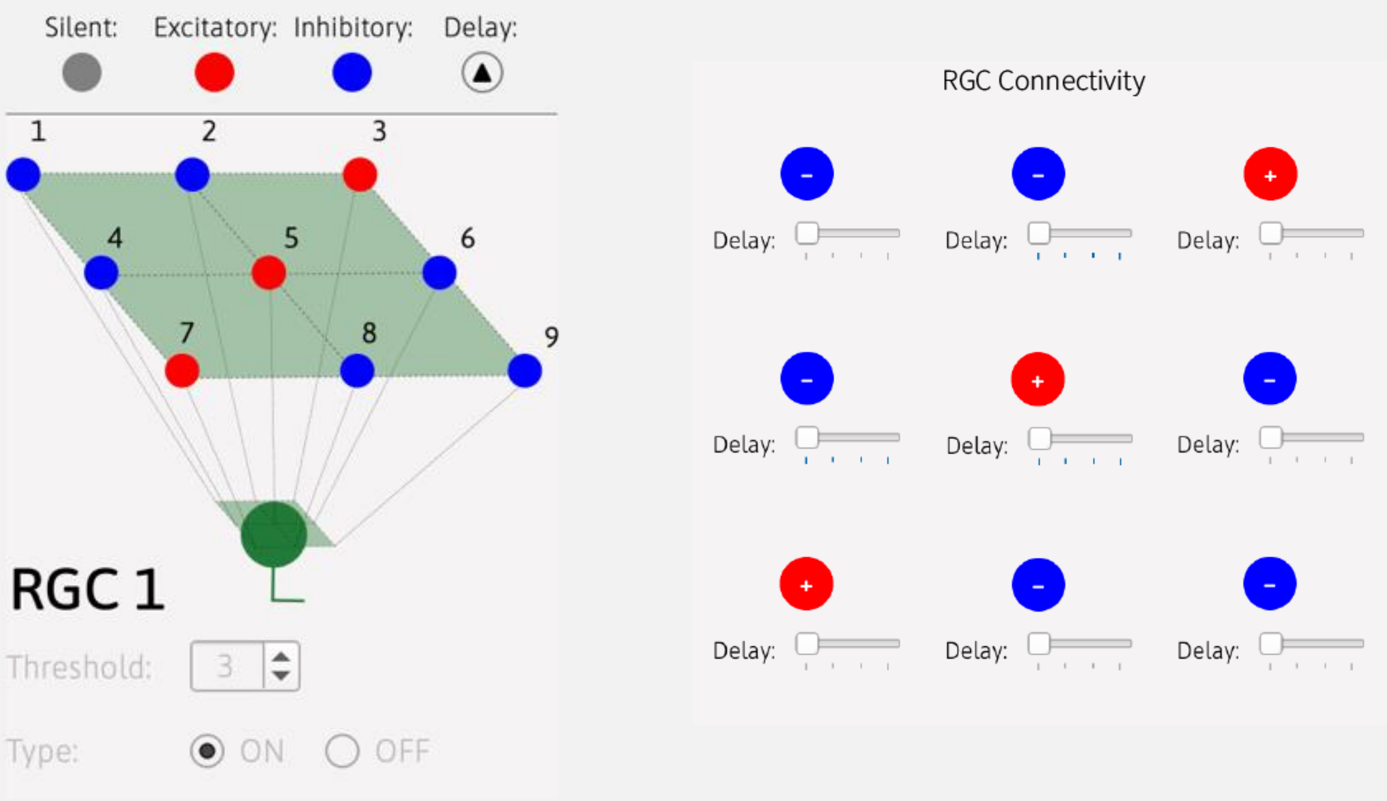

Signal Monitor

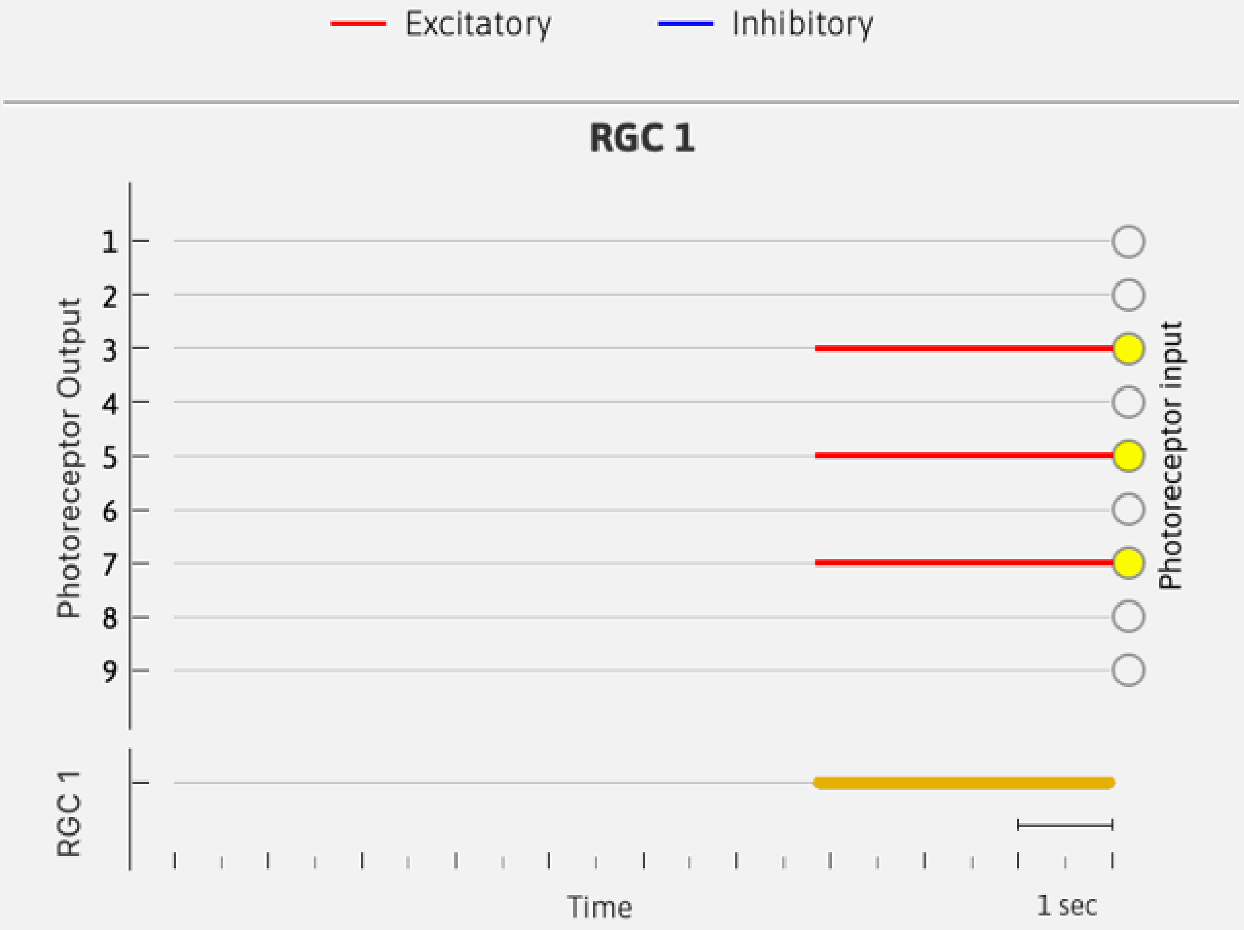

Objectives

Build

Build simple circuits that mimic retinal ganglion cells with different visual preferences

Test

Test your circuit by activating different LEDs to check the ganglion cell's selectivity

Real-world stimulation

Use the Visual Stimulus Tool or shapes cut out of paper/cardboard to see how robust your circuit is

Testing your circuits

LED activation

Manually activate different combinations of LEDs in the Visual Stimulus Controller to stimulate photoreceptors

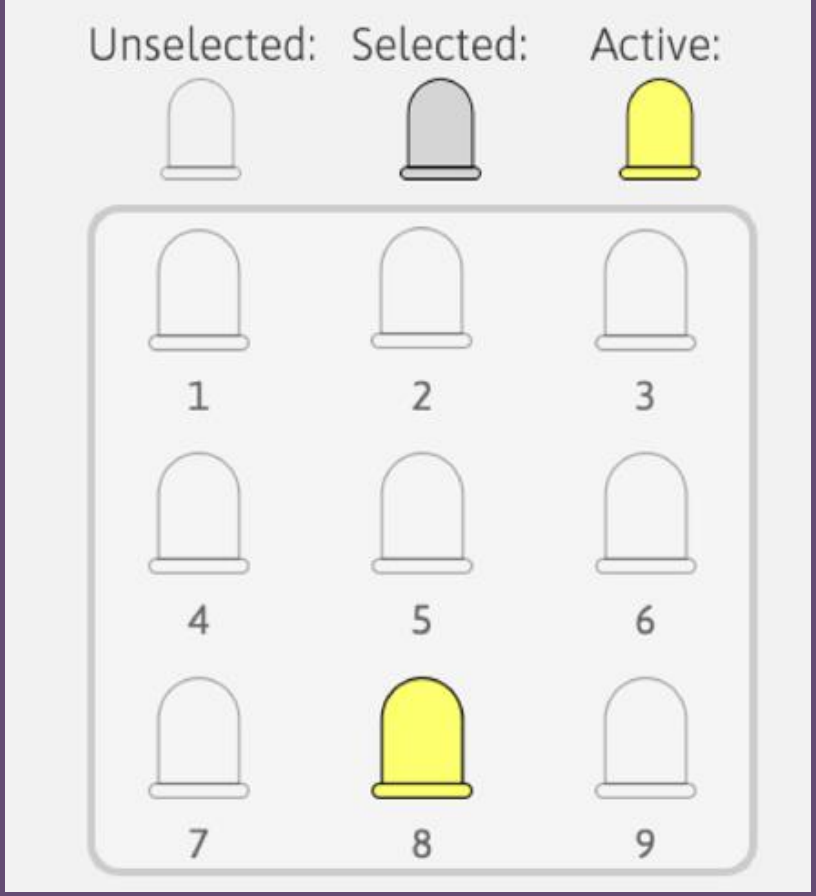

Testing your circuits
Manual stimulation

Turn on the entire LED array

Use the **Visual Stimulus Tool** (modeling clay on a clear board) or **shapes cut out of paper/cardboard** (stencils provided in the User Manual) to selectively block light from certain photoreceptors

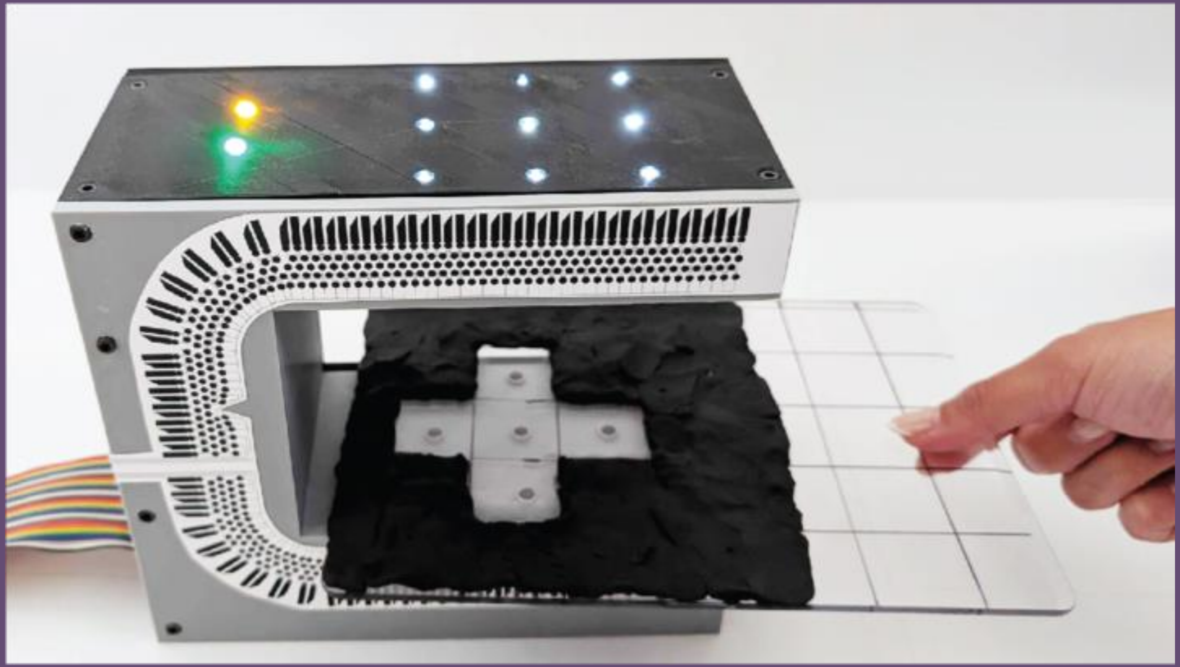

Lesson 1

ON/OFF and Center-Surround

How do neurons in your visual system know what to respond to in the visual world?

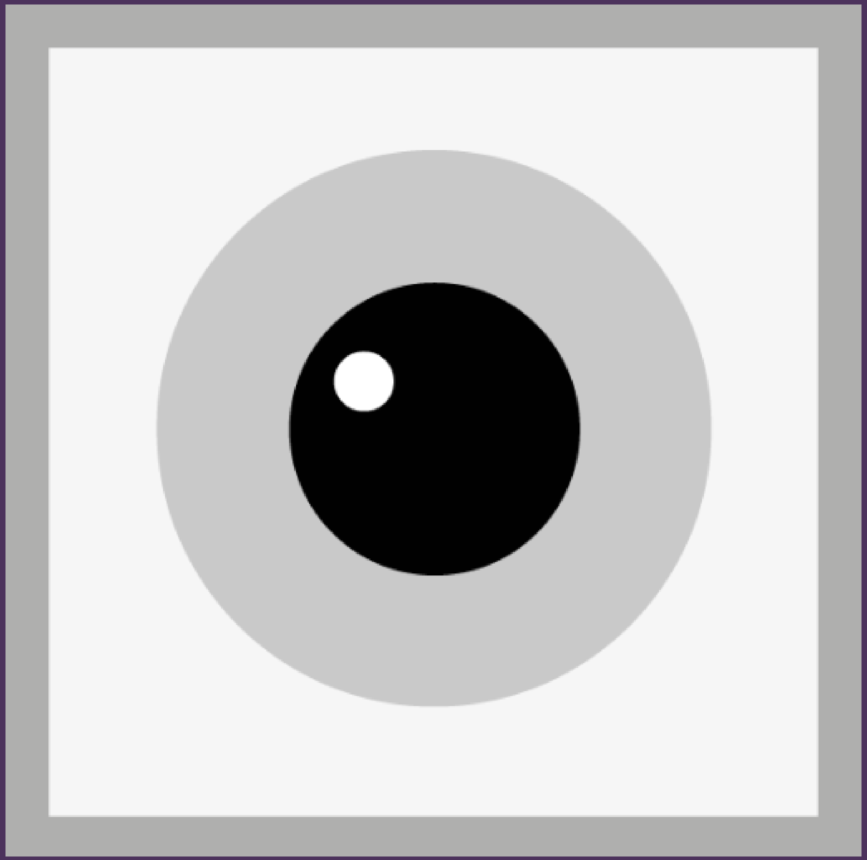

Each visual neuron has a **receptive field**: a specific location in the visual field that it can see.

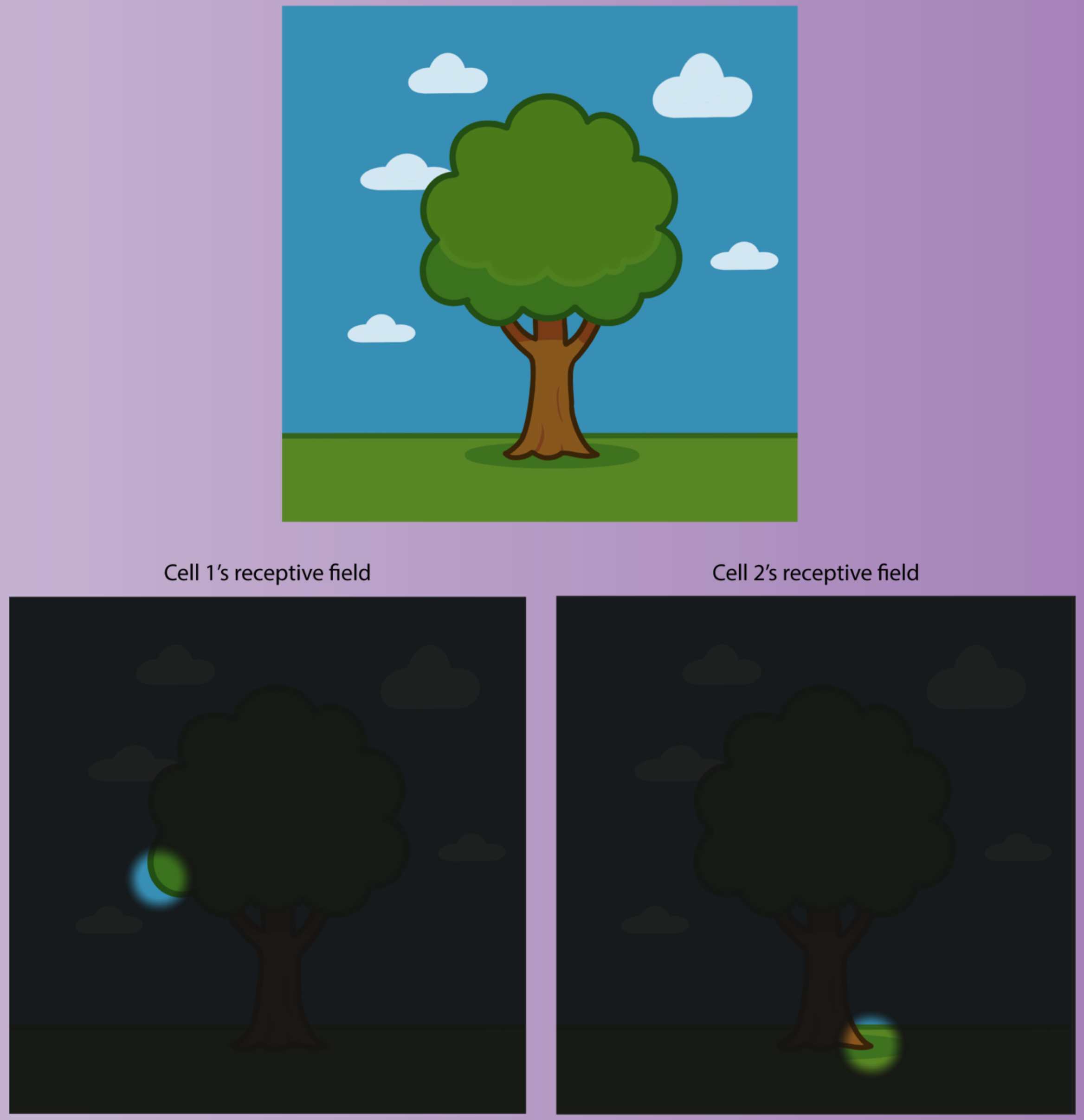

ON and OFF neurons

Within their receptive fields, some neurons prefer **bright stimuli** (increases in luminance), while some prefer **dark stimuli** (decreases in luminance)

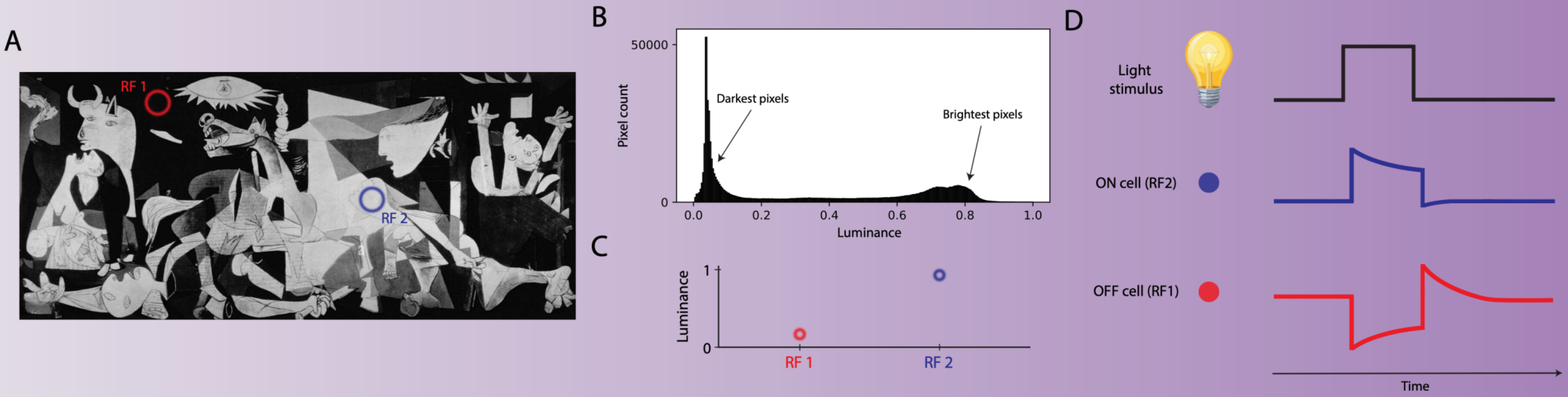

An **ON neuron** responds when the **luminance increases** within its receptive field.

An **OFF neuron** responds when the **luminance decreases** within its receptive field

ON / OFF responses in the real retina

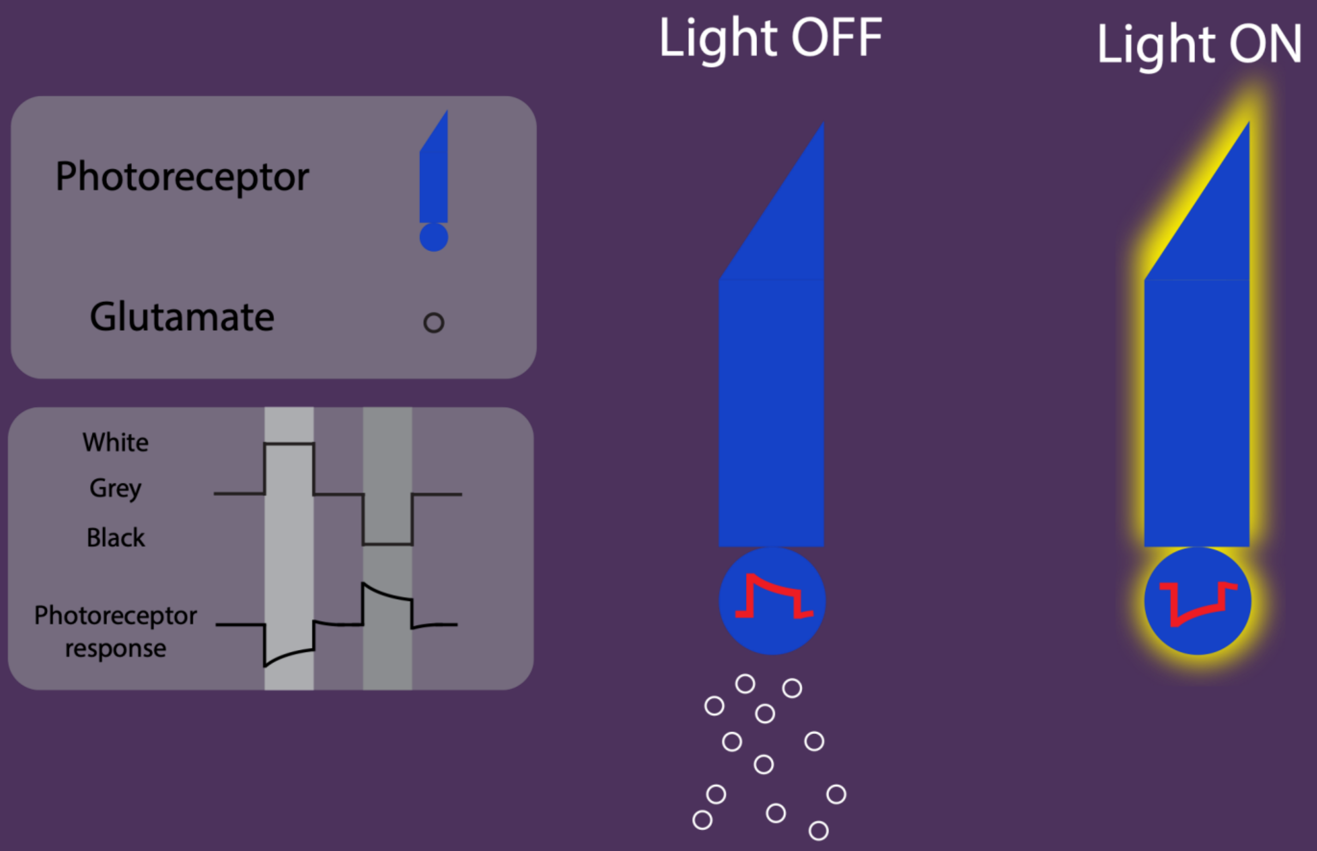

Photoreceptors are ***hyperpolarized*** by light - they release less glutamate in the light

ON / OFF responses arise in bipolar retinal neurons

OFF BCs have ionotropic glutamate receptors. They are ***sign-conserving***; when PRs hyperpolarize, OFF BCs hyperpolarize, and vice versa.

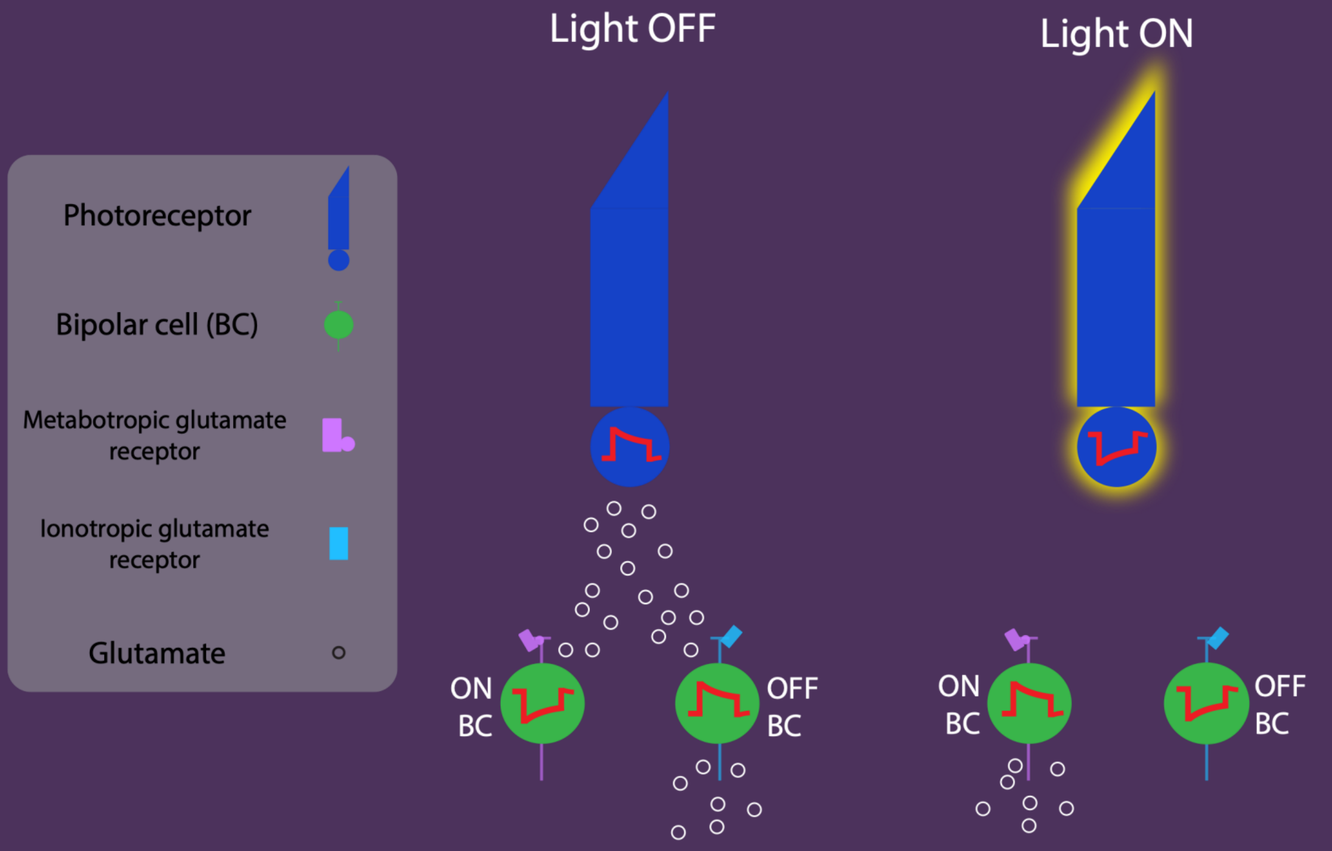

ON BCs have metabotropic glutamate receptors. They are ***sign-inverting***; when PRs hyperpolarize, ON BCs depolarize, and vice versa.

Center-surround receptive fields

Neurons with center-surround tuning respond when, within the small part of the visual scene that they see, one part of their receptive field is bright and another parts is dark.

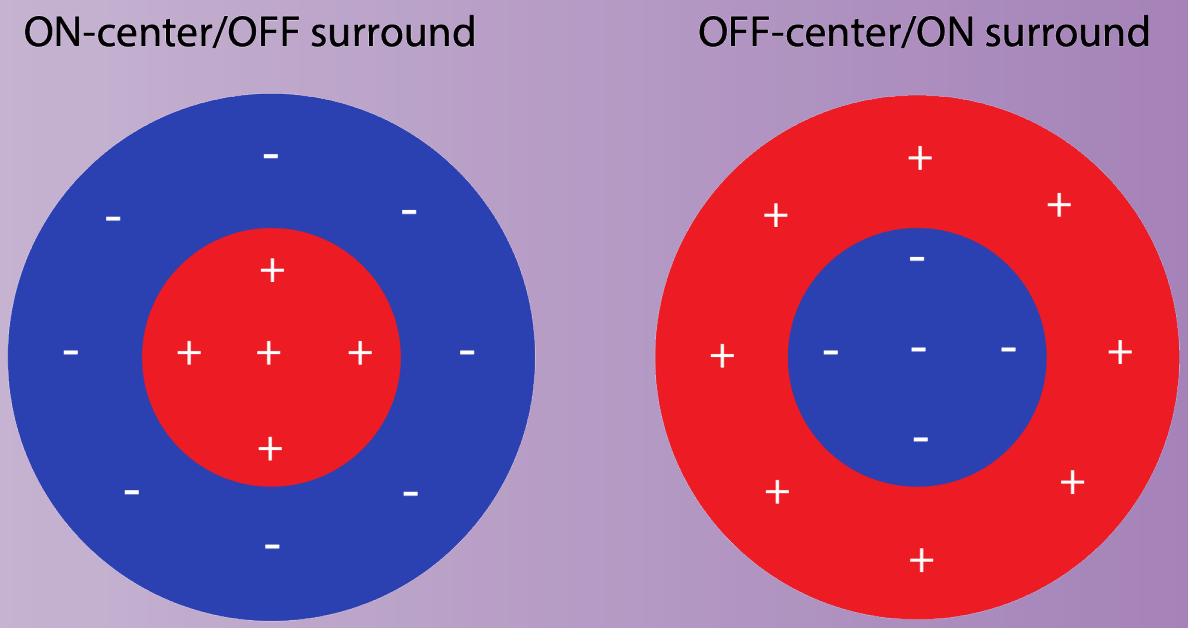

Center-surround receptive fields make neurons sensitive to luminance contrast

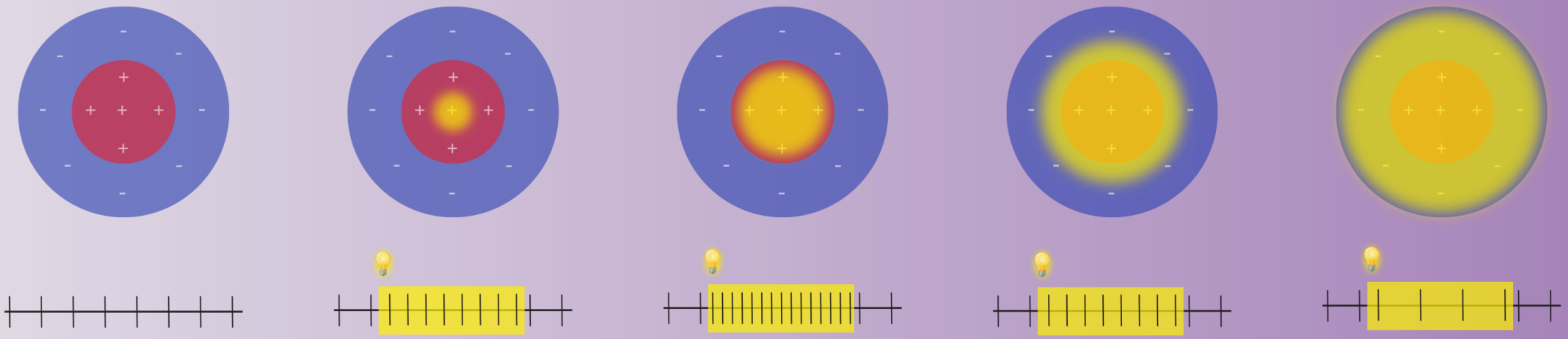

Light in the excitatory center drives the neuron

Light in the inhibitory surround reduces the neuron's response

The size of the center-surround receptive field controls the spatial frequency of luminance contrast that activates a neuron

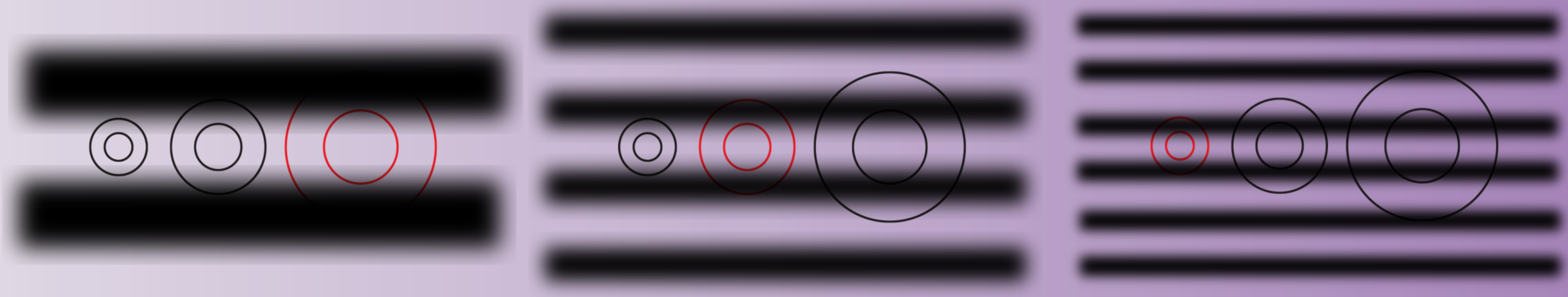

Visual neurons do not respond well to homogeneous scenes (e.g. a solid white wall)

Center-surround tuning in the real retina

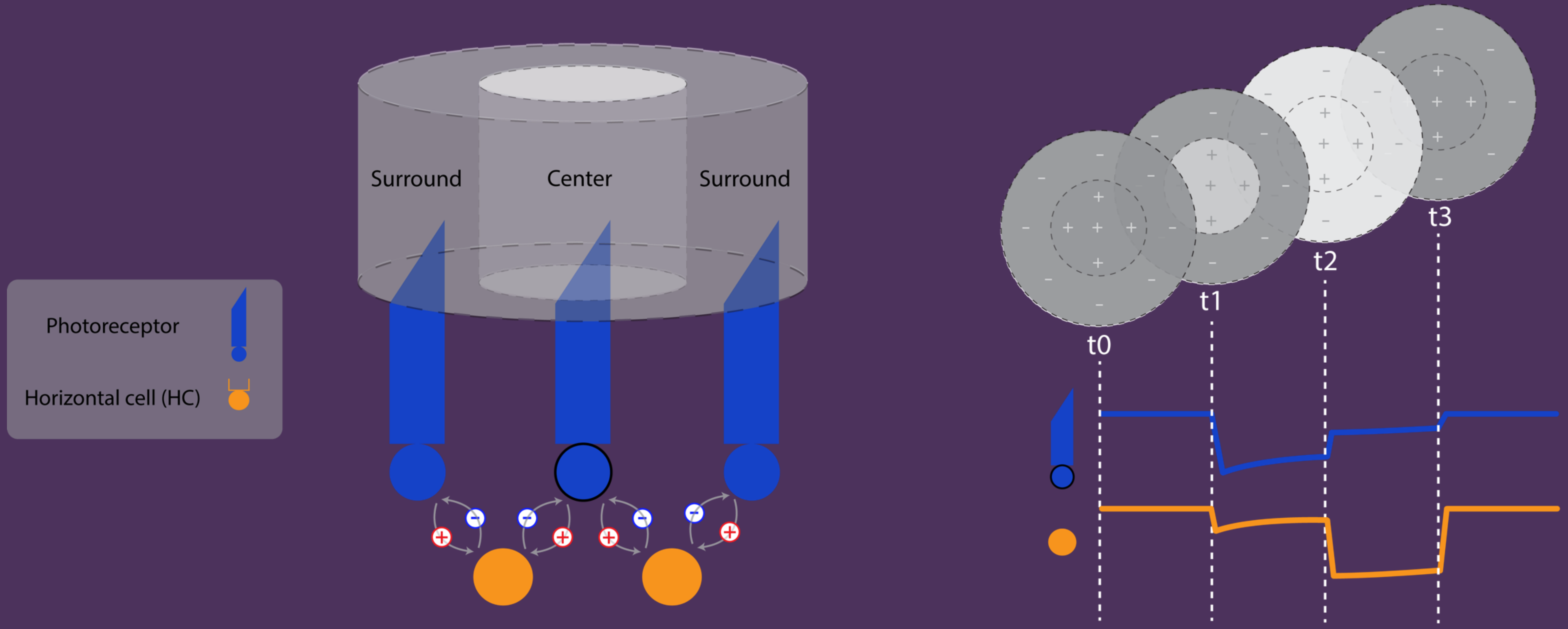

Horizontal cells provide negative feedback to photoreceptors

Center-surround tuning in the real retina

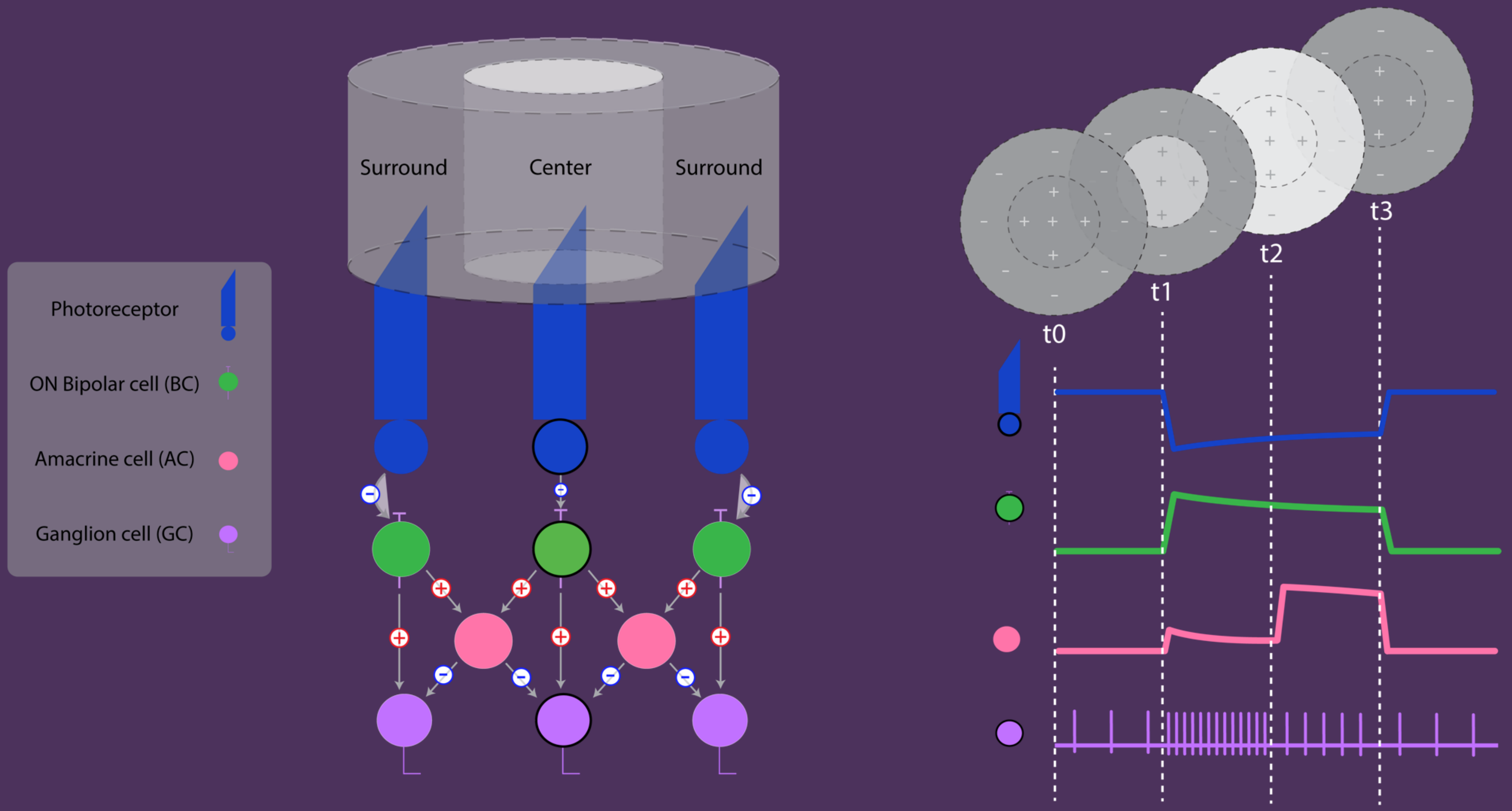

Amacrine cells provide inhibitory input that mediates RGC surround responses

Activity 1

Build a spot detector with an ON-center/OFF-surround receptive field (activated by light in the center of the receptive field but inhibited by light in the surrounding area)

Use the GUI to build a circuit (RGC1) with an ON-center/OFF-surround ganglion cell that responds only when the center model photoreceptor of the 3x3 array is activated by light, but not when any of the surrounding model photoreceptors are also activated, and not when a small spot is located above any other model photoreceptor.

Activity 2

Build a second spot detector with a different receptive field location

Build an ON-ganglion cell (RGC2) with the same size preference (as in Activity #1), but whose receptive field is in a different position on the photoreceptor array.

By moving the Visual Stimulus Tool, or a piece of paper/cardboard with a small circle cut out, you should now be able to activate each RGC, but only one at a time, based on stimulus position.

Activity 3

Build a second spot detector with the opposite polarity

Re-wire RGC2 such that it detects a *dark spot* instead of a light spot (i.e., make it an OFF cell). This cell should have the same receptive field center/surround as RGC1 built in *Activity #1*, but with the opposite polarity. RGC2 should be activated by light in the surround and an *absence* of light in the very center of its receptive field (i.e., it should be activated by a small dark spot in the center of the photoreceptor array).

Activity 4

Build two spot detectors with preferences for spots of different sizes

Generate two different ON-ganglion cells (RGC1 and RGC2) with the same receptive field location (center) but tuned to spots of light of different sizes.

One ganglion cell should detect a small spot of light, while the other should detect a slightly larger spot of light.

If you make two spots of different sizes with your Visual Stimulus Tool or paper/cardboard with circles cut out, you should only be able to activate RGC1 with the small spot, and only activate RGC2 with the larger spot.

Challenge

Codebreaking with center-surround receptive fields

You will be given a series of **visual stimuli**; each represented as a 3×3 grid of photoreceptors. Some photoreceptors are activated (yellow), while others are not activated (black). Each stimulus corresponds to **one letter** of the secret message.

You will also be provided with a **cipher**, which will help you decode the message. The cipher will tell you the visual feature preferences for RetINaBox's two ganglion cells, **RGC1 and RGC2. The cypher will also provide you with a way to decode the activity of RetINaBox ganglion cell activity into four letters (0 means a ganglion cell is inactive; 1 means a ganglion cell is active).**

Make sure that it is possible for both ganglion cells to be activated simultaneously by a single visual stimulus!

Codebreaking with center surround receptive fields

Example

To begin the challenge: Lessons > Lesson 1 > Code Breaker

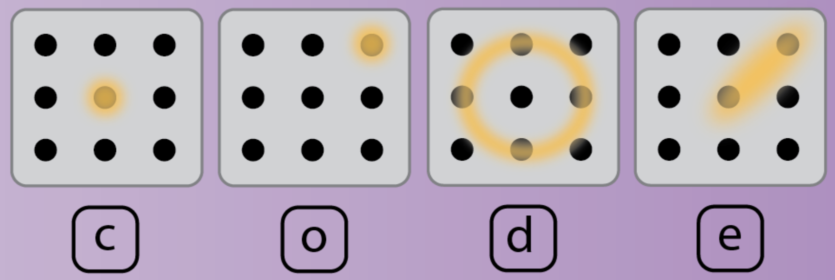

Cipher

RGC1
Preferred visual stimulus

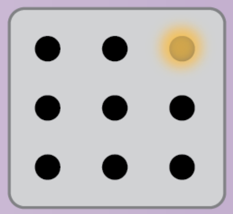

RGC2
Preferred visual stimulus

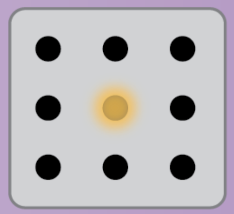

RetINaBox Output

RGC1	RGC2		
0	0	=	D
1	0	=	O
0	1	=	C
1	1	=	E

Lesson 2

Orientation Selectivity

How do neurons in the visual system
allow us to recognize things in the world?

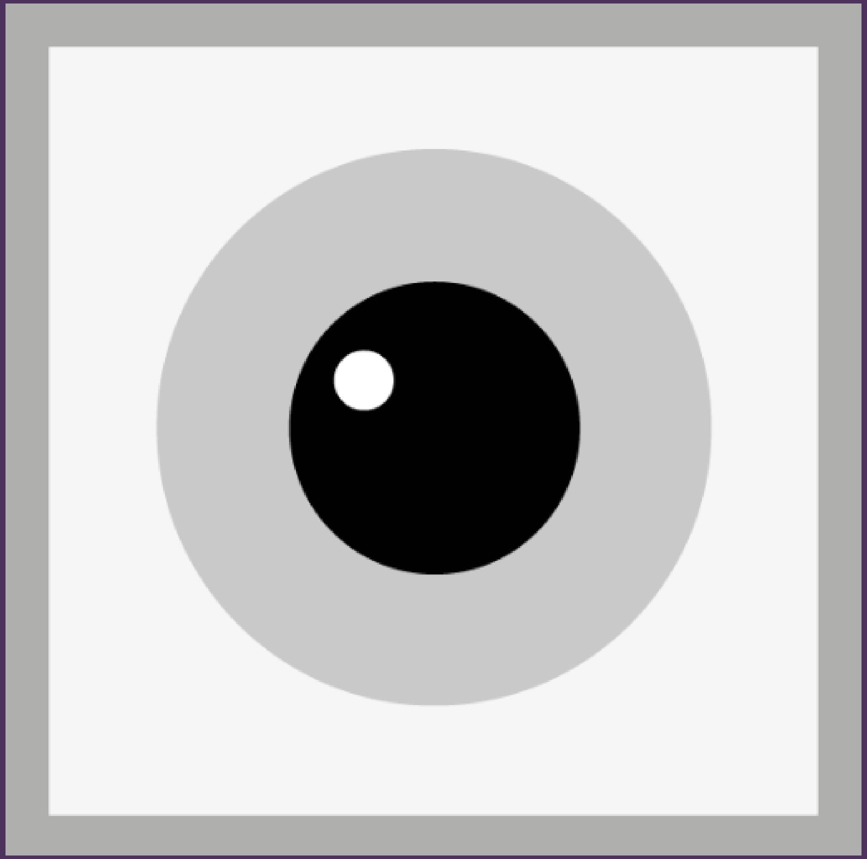

Most things in the visual world can be decomposed into a combination of lines of different orientations

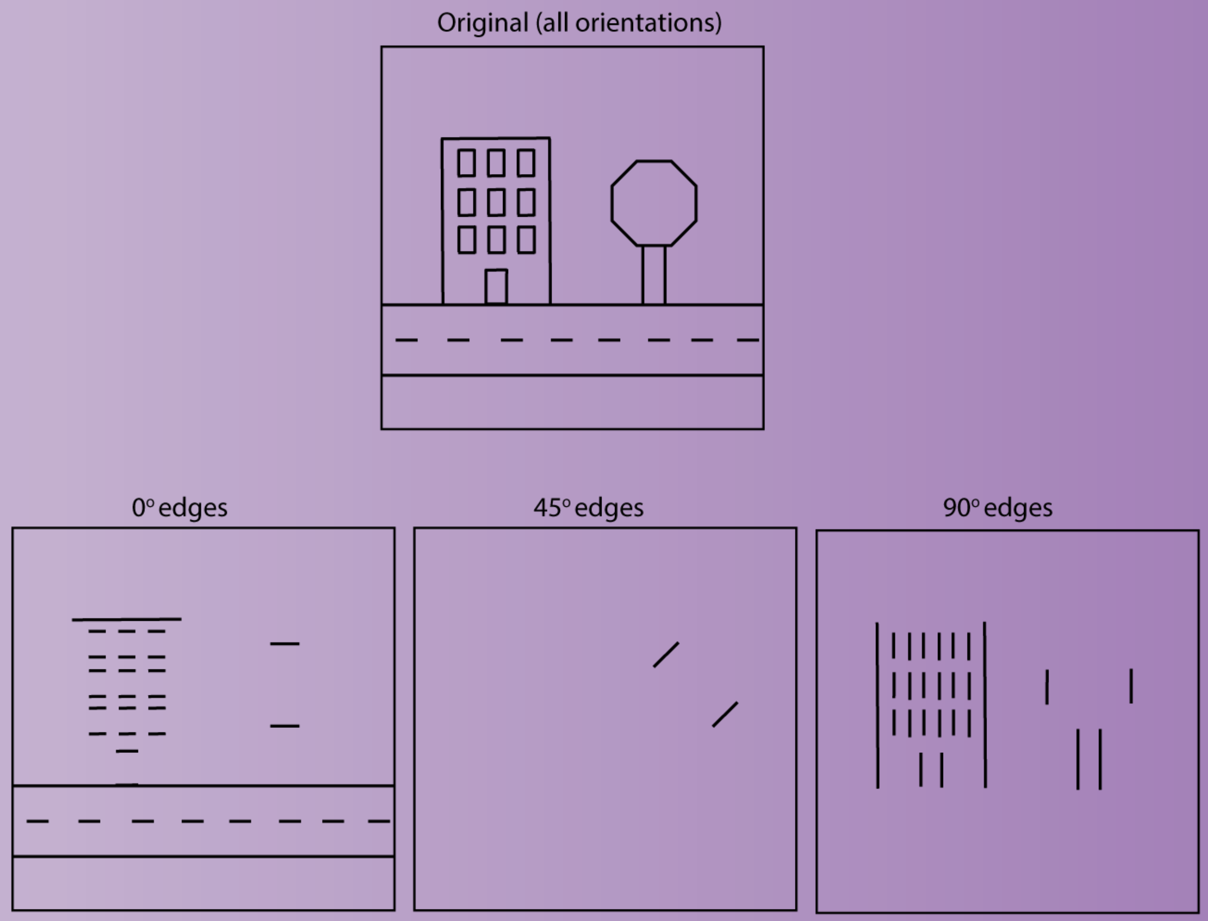

Our visual system has evolved neurons that respond to lines oriented at specific angles - it decomposes the visual world into its fundamental building blocks: oriented lines of varying orientation.

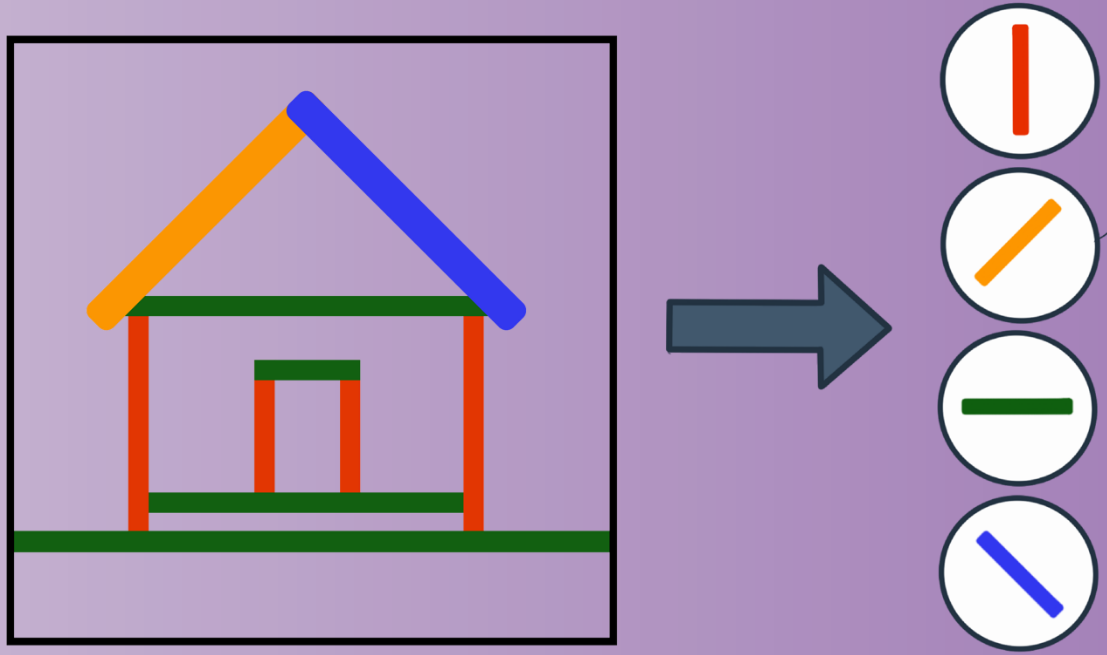

Orientation tuning in the brain

Several spatially-offset lateral geniculate neurons with center-surround receptive fields provide input to a single V1 neuron.

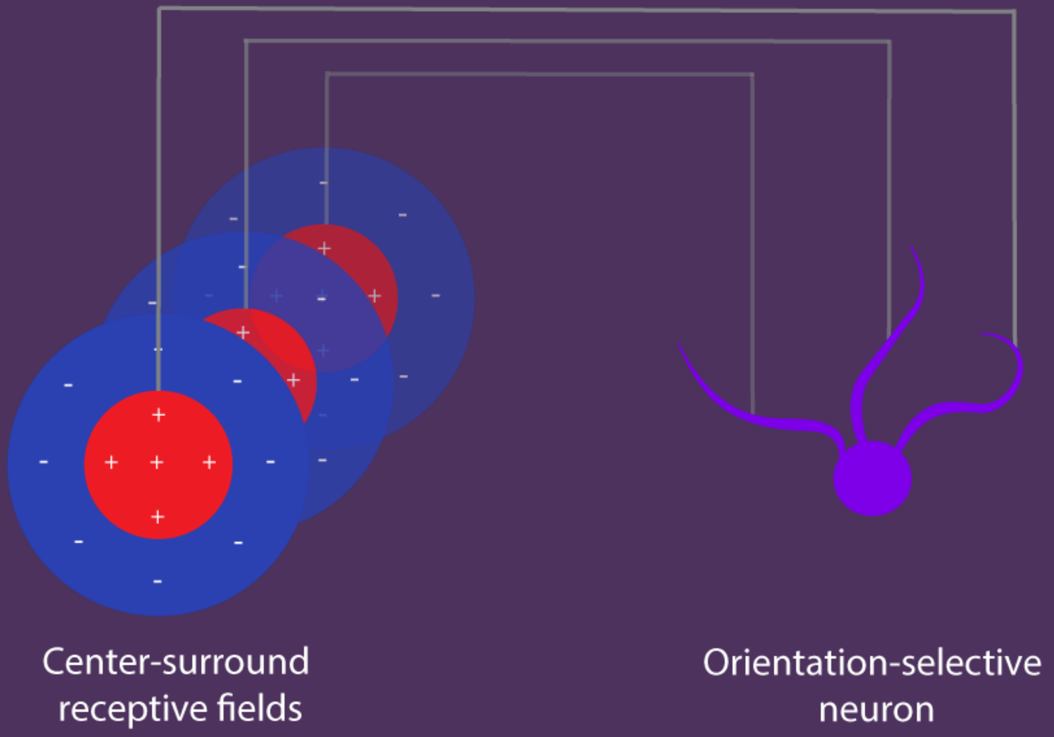

Orientation tuning in the brain

The aligned inputs cause the V1 neuron to respond selectively to an elongated bar of light oriented along the same axis.

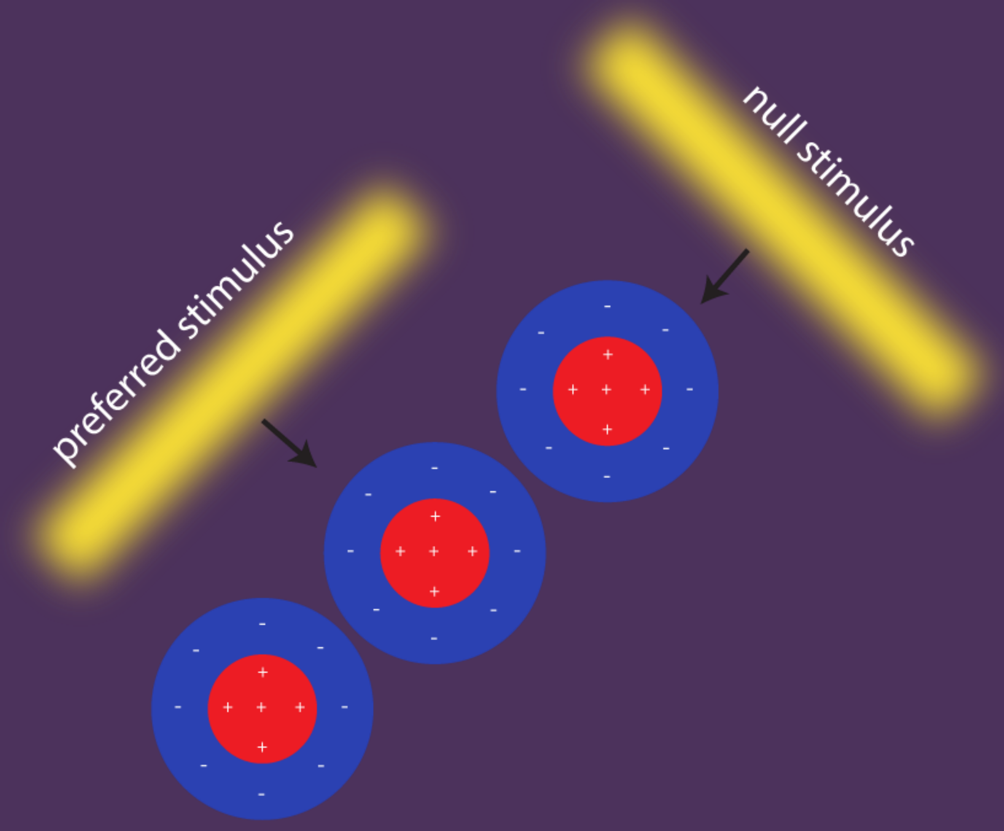

Activity 1

Build an ON-ganglion cell that detects a vertical line

Configure a circuit for RGC1 such that it only responds to a thin *vertical* line of light located in a specific part of the photoreceptor array.

The ganglion cell should not respond to a spot of light. The ganglion cell should not respond to a line of the same length of any other orientation/thickness or centered in a different part of the photoreceptor array

Activity 2

Build an OFF-ganglion cell that detects a vertical line

Configure a circuit for RGC2 such that it has the same receptive field as RGC1 in Activity #1 (i.e., responds to a thin vertical line).

However, rather than responding to a bright vertical bar, this ganglion cell should respond to a dark vertical bar—an absence of light in the same region—and should be inhibited by light in this area.

Activity 3

Build a second ON ganglion cell that detects a diagonal line

Build a circuit for RGC2 that responds to a line of the same thickness as Activity #1 but only responds when the line is diagonal to the first ganglion cell's preferred stimulus.

This second ganglion cell should not respond to a line of any other orientation or thickness, or to a line of the same orientation centered on a different part of the photoreceptor array.

Activity 4

Build two ON ganglion cells that detect vertical lines of different thicknesses

Configure RGC1 and RGC2 such that both cells respond to vertical lines of light, but with one selectively responding to a thin line and the other selectively responding to a thick.

Activity 5

Build two ON ganglion cells that detect vertical lines with different lengths

Configure RGC1 and RGC2 such that both cells respond to vertical lines of light with the same approximate spatial location, but with different lengths.

This activity emphasizes the concept of **end-stopping**, in which an orientation selective neuron responds only to an oriented line of a specific length.

Challenge

Build a shape detector with orientation selective receptive fields

Combine the outputs of two orientation selective ganglion cells to detect a specific shape (i.e., an X, +, T, or L): the shape arising from the combination of the lines that activate ganglion cell 1 and ganglion cell 2.

Build a buzzer that sounds only when the target shape is present (i.e., when both ganglion cells are activated).

Build a shape detector with orientation selective receptive fields

Building the buzzer circuit

Once you've built the buzzer circuit (please consult the RetINaBox manual), connect the outputs of the two ganglion cells (the 3.3V digital out pins on the back of RetINaBox) and one of the grounds, to the buzzer circuit.

The buzzer should only sound when you present your target shape.

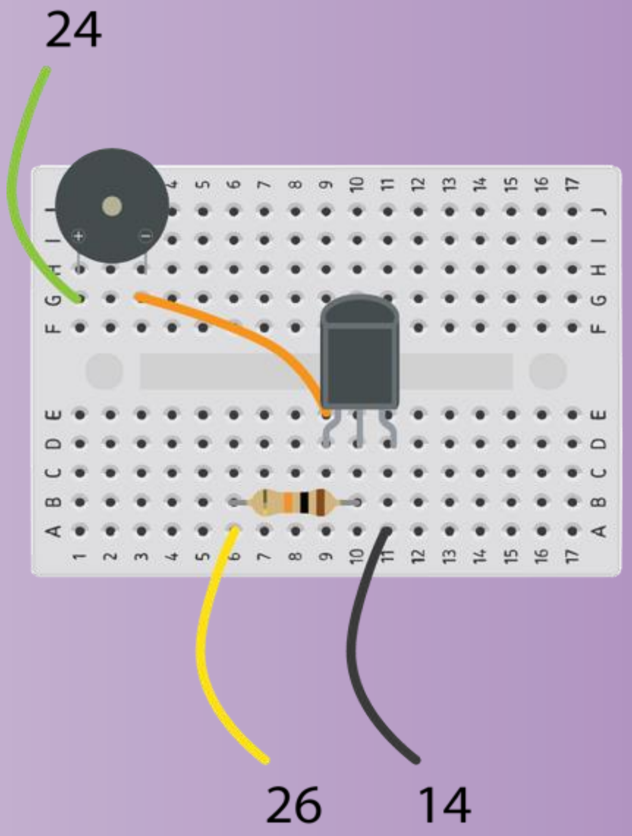

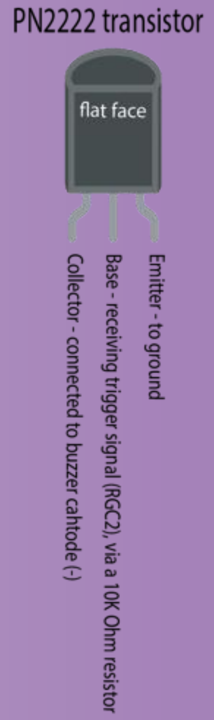

Lesson 3

Direction Selectivity

How do neurons in the visual system detect motion?

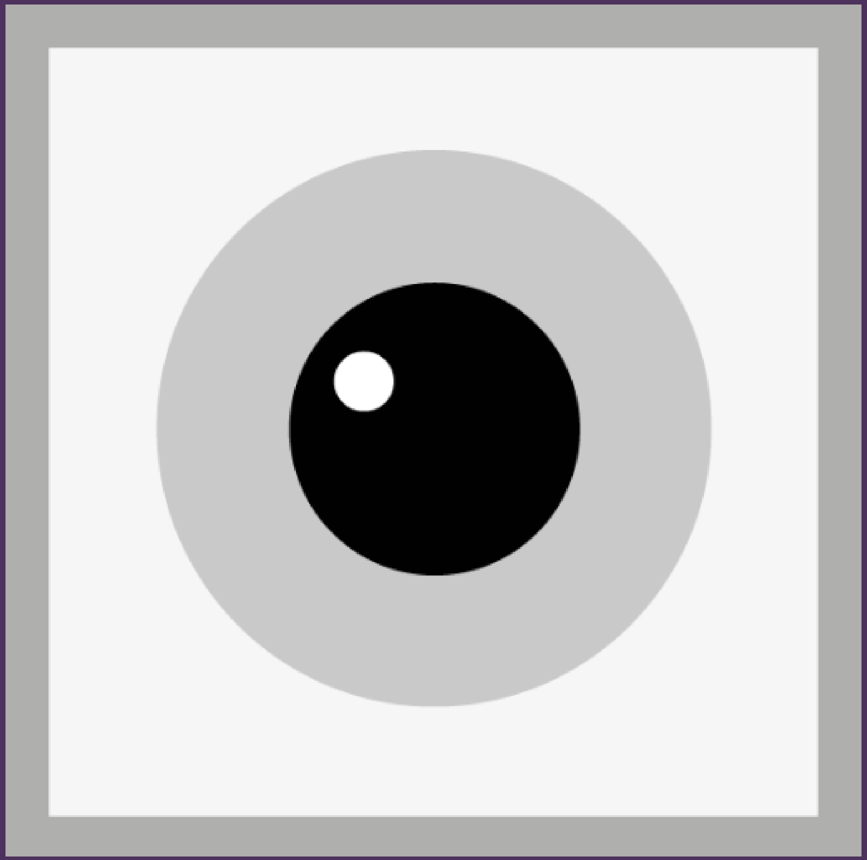

Visual neurons with **direction selective receptive fields** help our brain know if something is approaching, moving away, or moving tangentially to us.

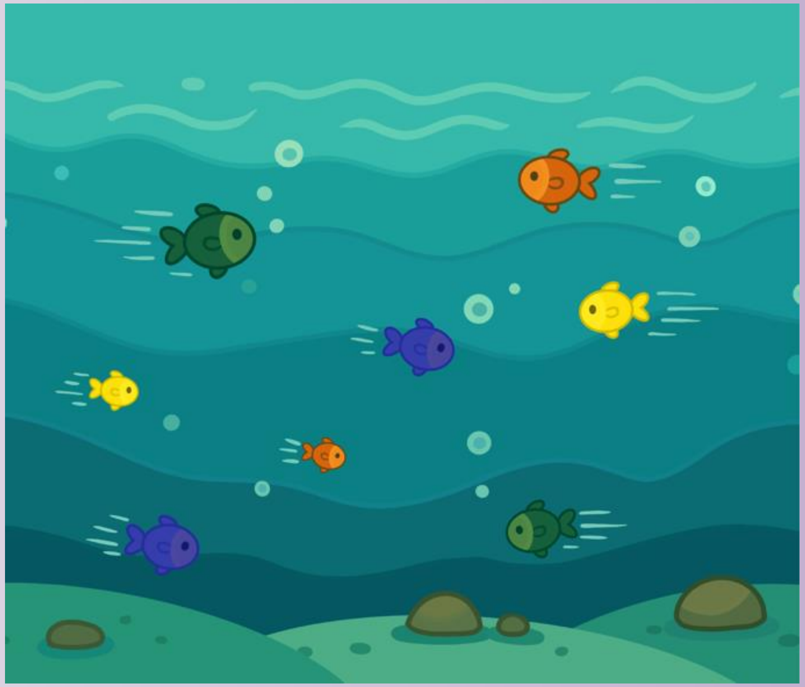

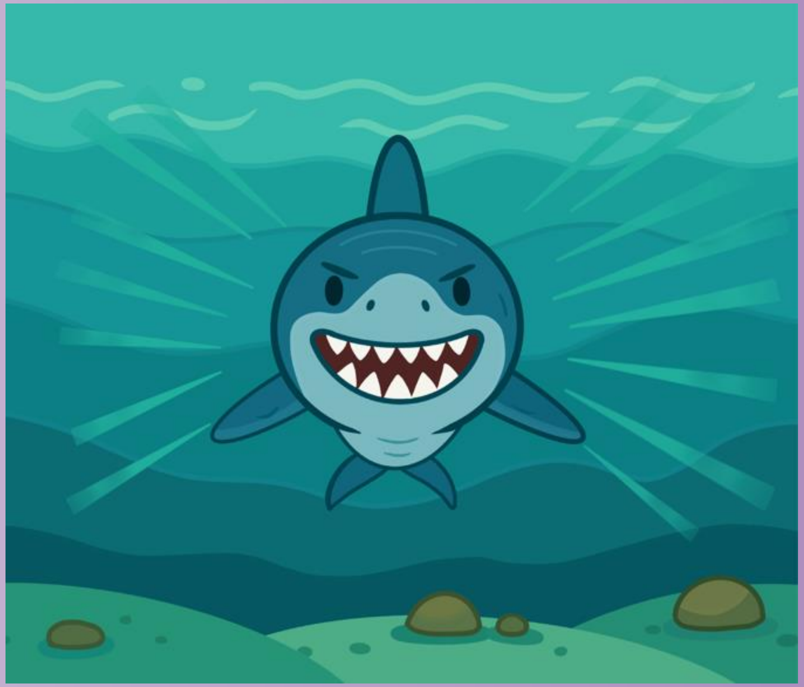

This ability is vital for **survival**—it helps animals track prey, avoid predators, and navigate through the world.

This can also help us differentiate movement in the visual world that we generate (by moving our bodies/heads/eyes) from motion that is external to us (like a bird flying in the sky)

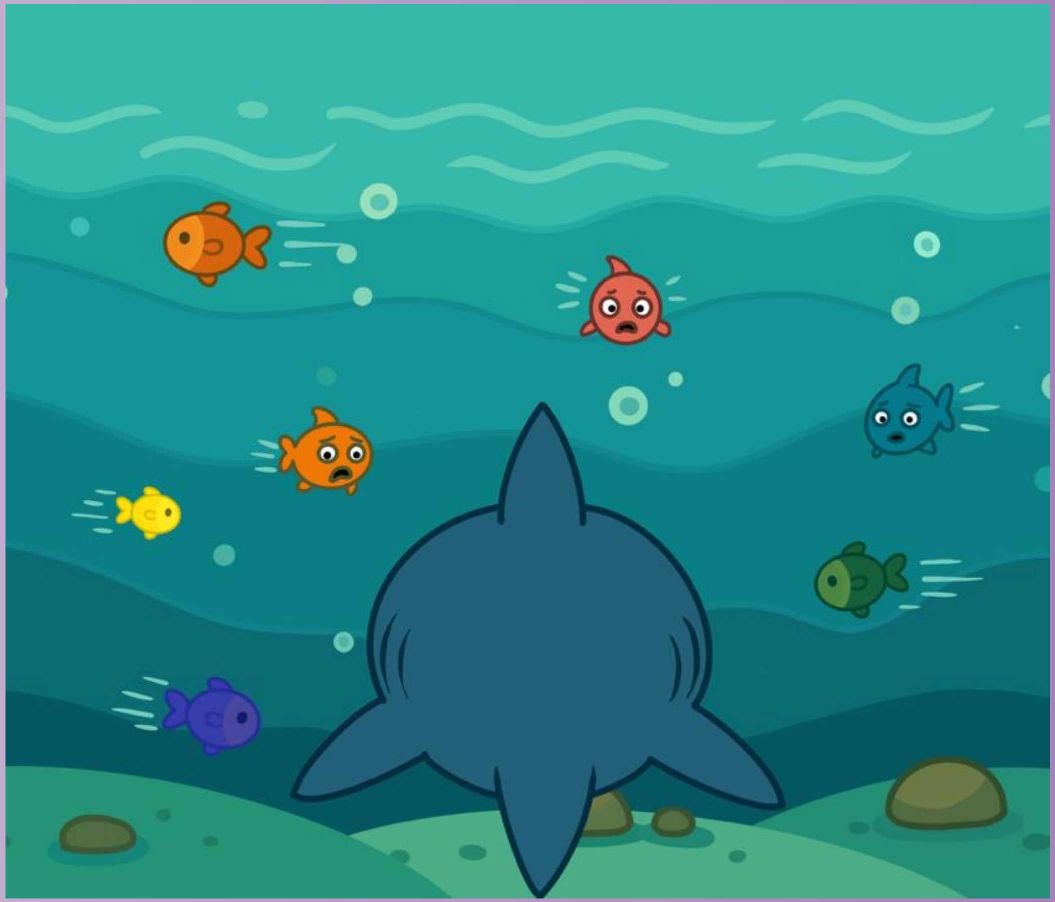

As something moves across your visual field, different photoreceptors with spatially offset receptive fields are activated in sequence.

Photoreceptors at the leading edge of the motion respond first, followed by those in the middle, and finally the trailing edge.

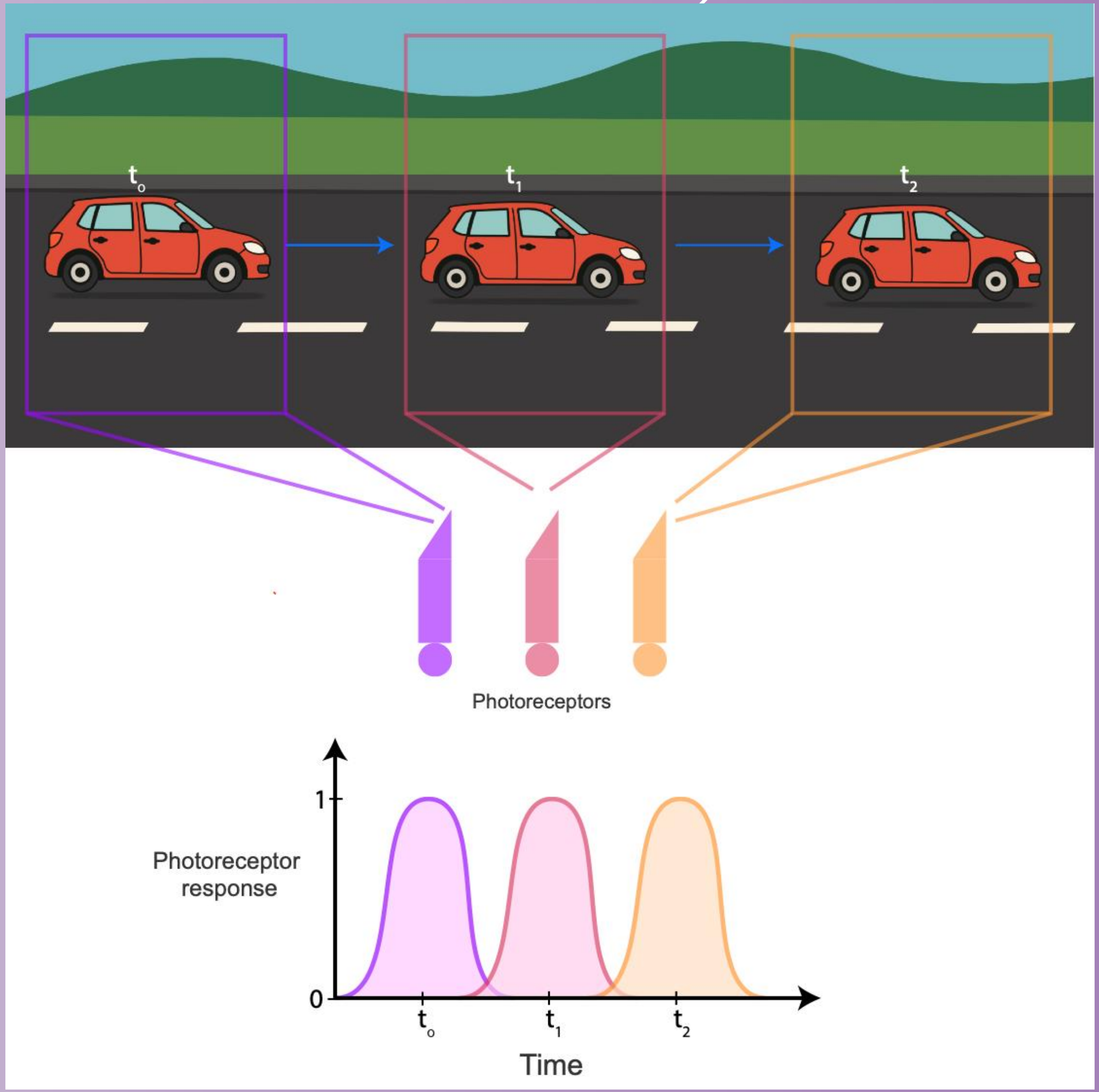

Direction selective tuning in the real retina

The brain leverages a time delay between two excitatory inputs to generate directionally selective responses of a neuron that *maximally* responds to rightward motion.

This delay can occur if one of the presynaptic neuron's signal takes longer to travel to the postsynaptic neuron, or if it travels more slowly through the postsynaptic neuron's dendrites before reaching the cell body.

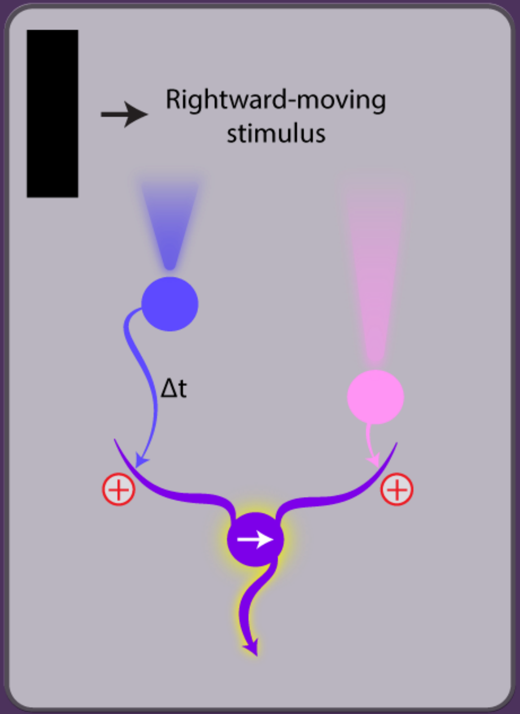

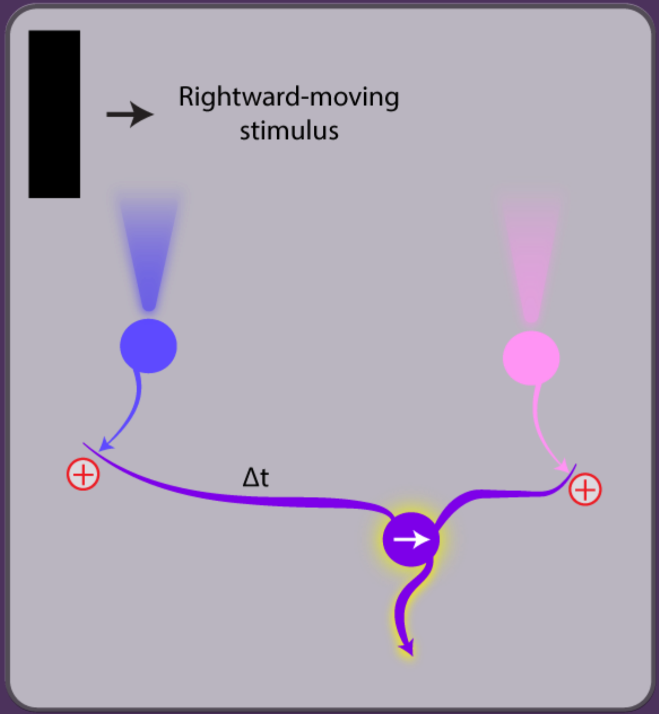

Direction selective tuning in the real retina

In addition to utilizing delays in excitatory circuits, delays with an inhibitory circuit element can enhance direction selective processing

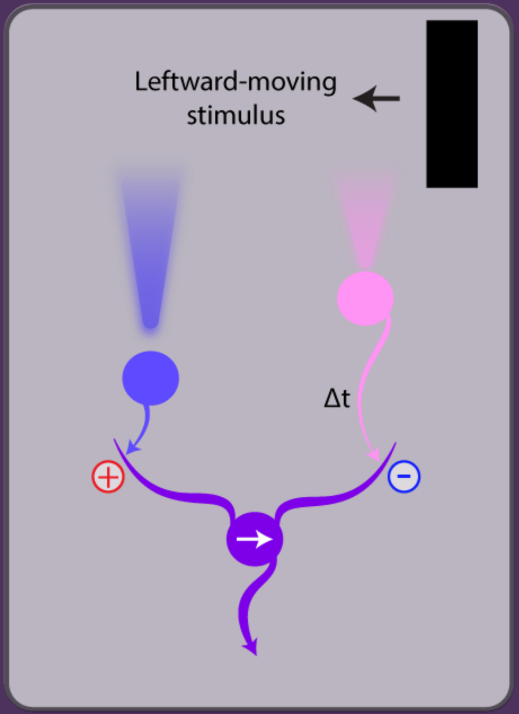

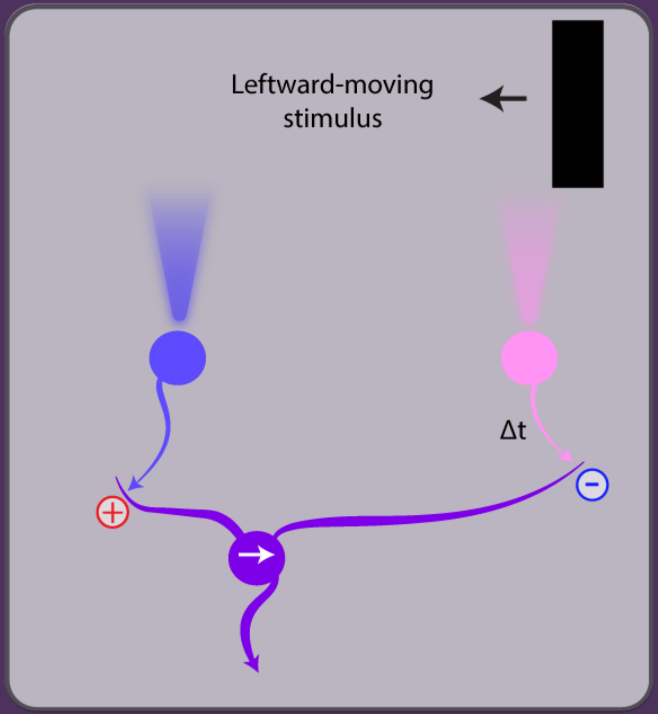

Activity 1

Build a left-preferring direction selective ganglion cell

Build a circuit where RGC1 responds to a vertical line of light moving leftwards.

Test your circuit by moving your hand across your array in both left and right directions.

Begin with a short temporal delay for your first motion-selective circuit. Afterwards, experiment with longer delays tune cells to different speeds of motion.

Activity 2

Build a right-preferring direction selective ganglion cell

Build a second circuit where RGC2 responds to a vertical line of light moving rightwards.

Test your circuit by moving your hand across your array in both right and left directions.

Activity 3

Build a slow/fast-moving right direction selective ganglion cell

Build a circuit where one ganglion cell responds to a vertical line of light slowly moving rightwards, while the second ganglion cell responds to a vertical line moving in the same direction, but more quickly.

Test your circuit by moving your hand rightwards across your array at various speeds.

Challenge

Block Breaker

Players move a paddle to bounce a ball to break several rows of bricks. The goal is to clear all the bricks without letting the ball drop.

Your task is to configure two ganglion cells with opposite direction selectivity and use them as the input controls for the game inside the RetINaBox GUI.

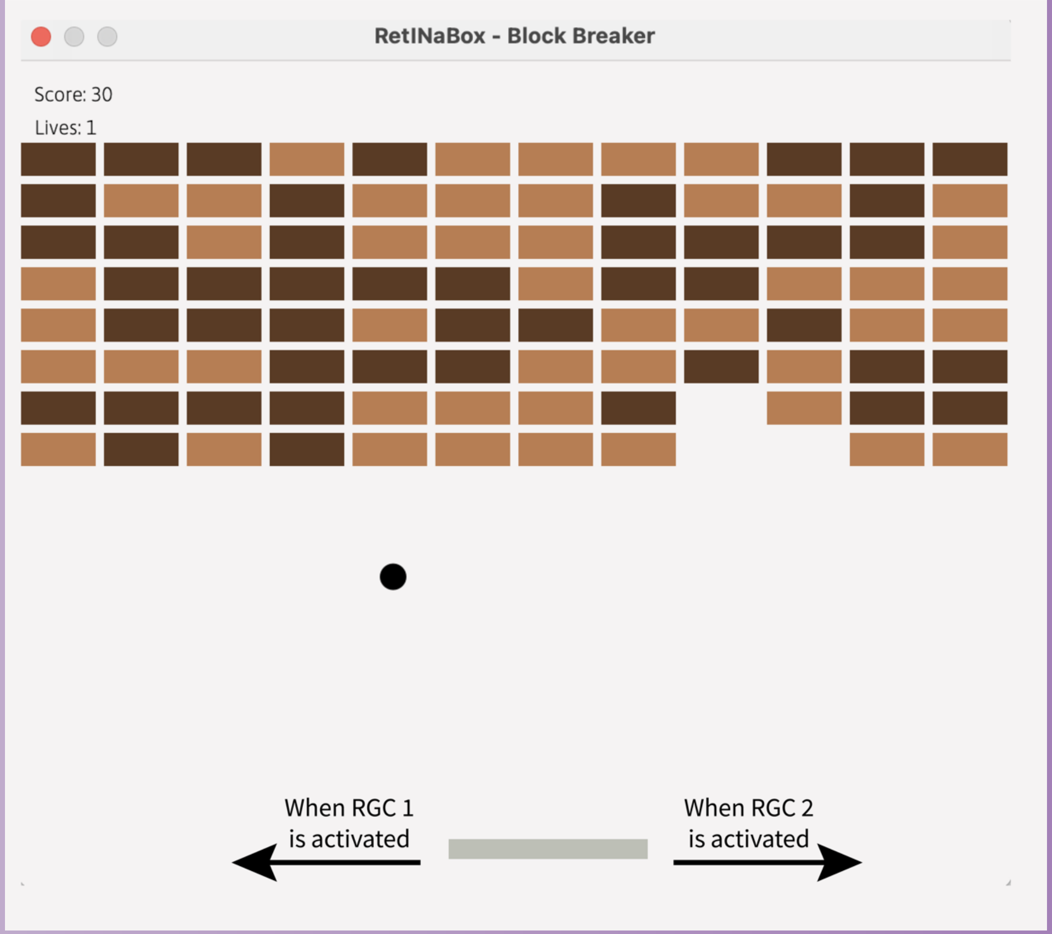

Block Breaker

Step 1

Configure both ganglion cell circuits so that RGC1 is selective only for **leftward motion** and RGC2 is selective only for **rightward motion.**

Step 2

Load the game. From the Menu tab in the GUI, navigate to 'Block Breaker' (**Lessons > Lesson 3 > Block Breaker**).

Step 3

You're now **ready to play**! The block breaker paddle is controlled by you sweeping a visual stimulus (your hand) leftward and rightward across RetINaBox's field of view.

Lesson 4

Discovery Mode

It’s your turn discover which visual stimuli best activate visual neurons and discover what circuit connectivity properties underlie such feature selectivity.

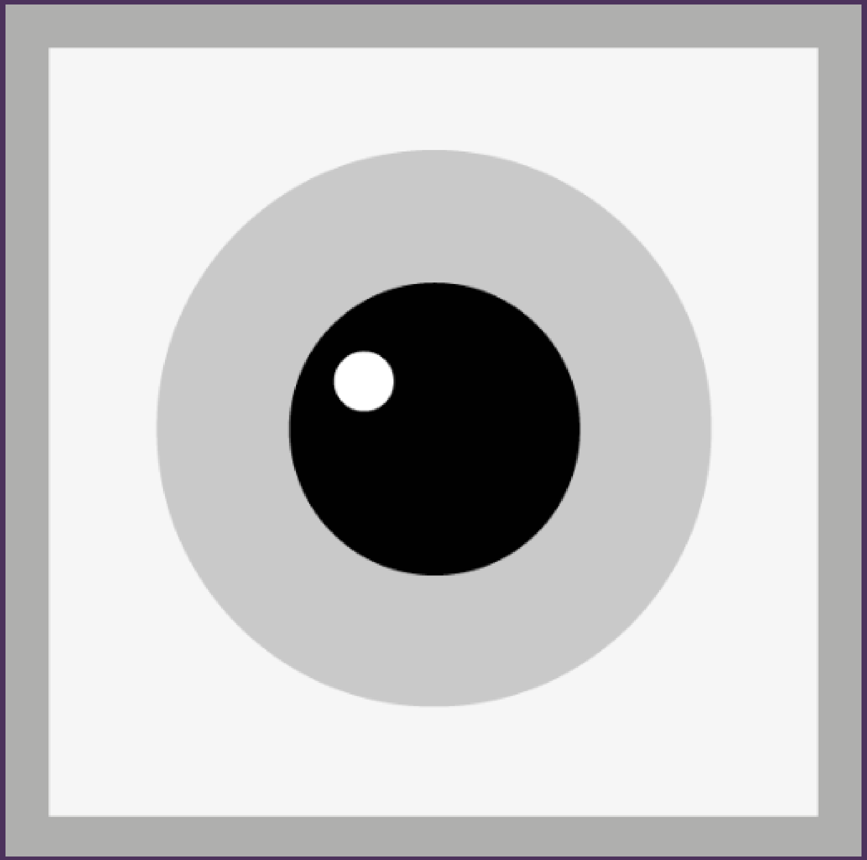

In Discovery Mode, there are three levels of difficulty (**Easy, Medium, Hard**), each with their own set of challenges.

For each challenge, you'll start with 100 points. Incorrect answers cost you 5 points.

Your goal is to complete each challenge with the highest score possible.

From the Menu tab, navigate to 'Discovery Mode' (Lessons > Lesson 4 > Discovery Mode). Then, select a mystery circuit from the drop-down menu.

Phase 1: Discover the Preferred Stimulus

Figure out what each ganglion cell responds to (i.e., a specific shape, or a direction of motion, etc.).

Use your Visual Stimulus Tool or paper/cardboard with different shapes out to test different stimuli to figure out what the ganglion cell is tuned to.

Phase 2: Discover the Circuit Connectivity

Figure out how the ganglion cell obtains this selectivity.

How are the photoreceptors connected to the ganglion cell? What kinds of **delays**, **polarities**, or **spatial arrangements of the photoreceptors** give rise to this selectivity?

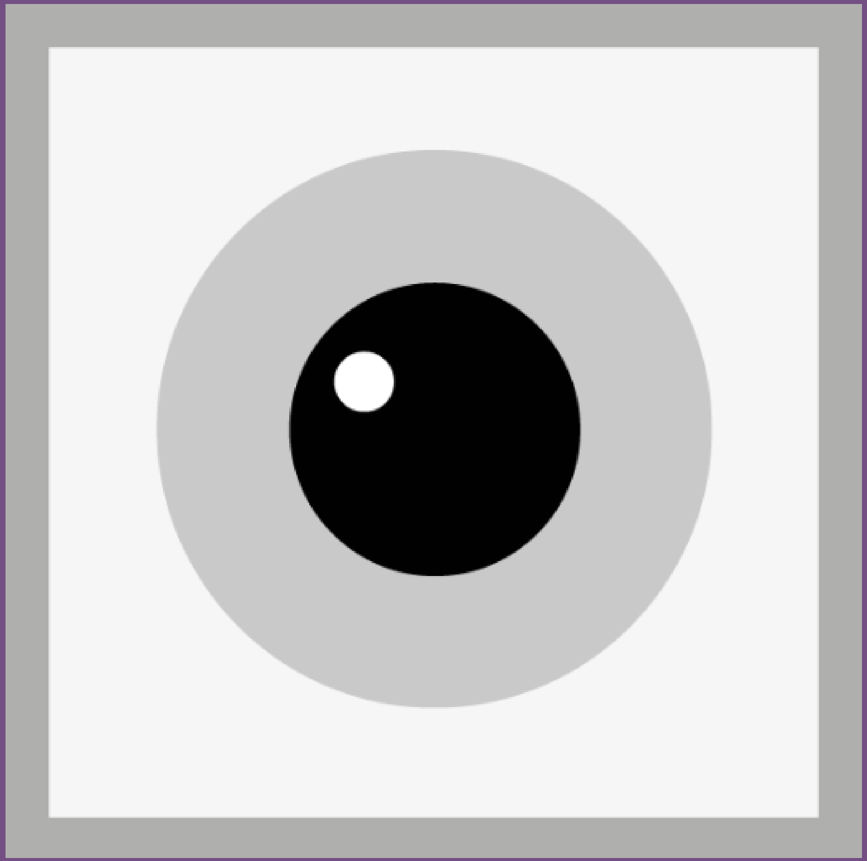

neuro
Montreal Neurological Institute-Hospital

McGill

Thank You!

Developed by the Trenholm Lab at McGill University

Solutions and additional details are provided in the Lesson Plan. Instructions for setting up hardware and software are available in the RetINaBox User Manual: *http://www.trenholmlab.com/retinabox.html*

Access code and materials: *https://github.com/Trenholm-Lab/RetINaBox*

For a more detailed overview of the retina and the visual system, visit theopenbrain.org and https://www.webvision.pitt.edu/
