# Supplementary material for: RetINaBox: A Hands-On Learning Tool for Experimental Neuroscience
Source: eNeuro. 2026 Jan 2;13(1):ENEURO.0349-25.2025. doi: 10.1523/ENEURO.0349-25.2025 (PMC12813302; doi:10.1523/ENEURO.0349-25.2025)
Supplement: Data 1 — Download Data 1, ZIP file. [file eneuro-13-ENEURO.0349-25.2025-s003.zip › RetINaBox_UserManual.pdf]

RetINaBox User Manual

Instructions for setting up hardware and software

Version 1.2

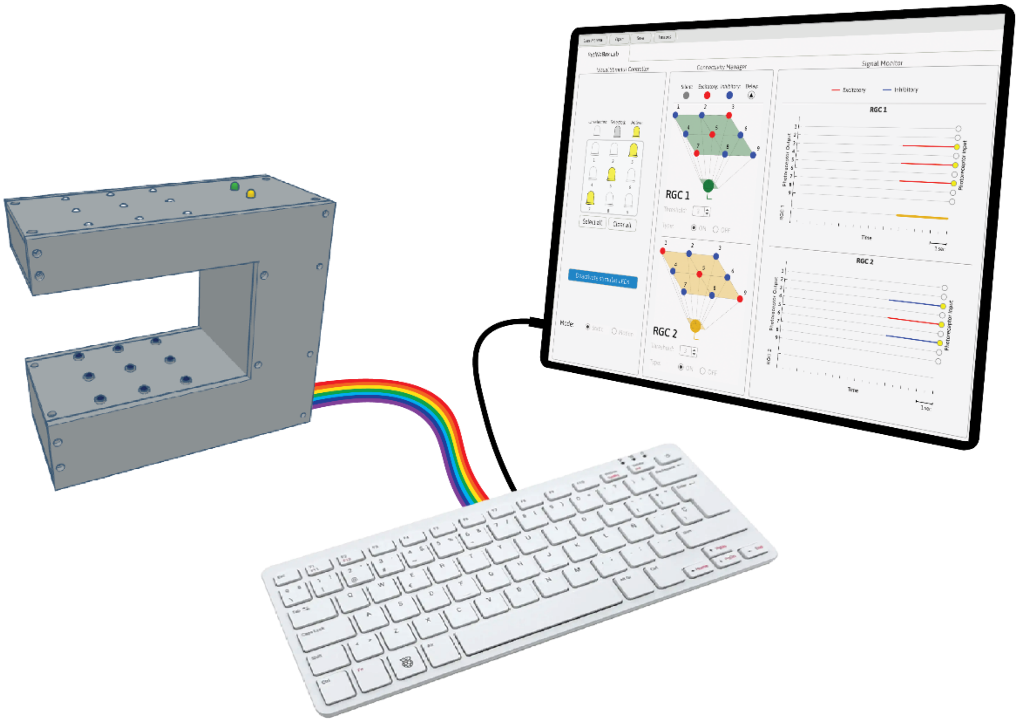

Table of contents

1. Introduction
2. Building RetINaBox
3. Generating Visual Stimuli
4. Installing the Software
5. Connecting RetINaBox to the Raspberry Pi
6. Using the Software

A1: Appendix 1: Components List
A2: Appendix 2: Troubleshooting
A3: Appendix 3: Costs

1. INTRODUCTION

Welcome to the RetINaBox User Manual. Here you'll find point-by-point instructions for building and assembling RetINaBox and for installing and using the software. In addition, we provide a full components list required to build RetINaBox. For those unfamiliar with working with electronics, don't worry, you don't need to solder anything. For those already familiar with electronics, the design of RetINaBox is simple, and it should be possible to build it with different LEDs, photodiodes, resistors, etc. than the exact ones specified here as long as those electronics components are compatible with the Raspberry Pi. Lastly, we provide a troubleshooting section in case you get through the manual and things still are not working correctly.

2. BUILDING RetINaBox

RetINaBox is built from a 3D-printed case that houses LEDs, photodiodes, and a bunch of wires that connect these components to the Raspberry Pi. The case is composed of six 3D printed pieces joined together, mostly with M3 screws (as outlined below). Although the case does not have to be assembled in the order indicated in the manual, the order we outline below ensures that case assembly is as straightforward as possible. Wiring the different components will be outlined as well, with electronics components being integrated into the case during the assembly process. Before starting, it is recommended to have all the necessary components and tools on hand. The assembly steps are as follows:

a) Wiring

- Wiring the photodiodes
- Wiring the LEDs arrays
    - IR LEDs arrays
    - White LEDs arrays
    - Coloured LEDs

b) Case Assembly

- Description of the 3D printed case parts
- Putting together the 3D printed parts

c) Connecting electronics to Raspberry Pi GPIO

- Connecting the photodiodes to the GPIO
- Connecting 3.3V out pins to the GPIO
- Wiring the buzzer (for Lesson 2)
- Connecting the LED panels to the GPIO
    - White and IR LED panels
    - Coloured LEDs

a) WIRING

1. <u>Wiring the photodiodes</u> (~30 min)

   Materials needed: 9 infrared (IR) photodiodes, jumper wires, wire cutters

   a. Have 9 photodiodes at hand. Remove or fold back the clear LED from the photodiodes (**Fig. 1**; these LEDs will not be used in RetINaBox). These photodiodes will be powered with a constant 3.3V from the Raspberry Pi, and the Raspberry Pi will also independently read the output signal from each photodiode (i.e. whether the photodiode is detecting light or not). Each photodiode board contains a potentiometer, which will be used to adjust light sensitivity (outlined below).

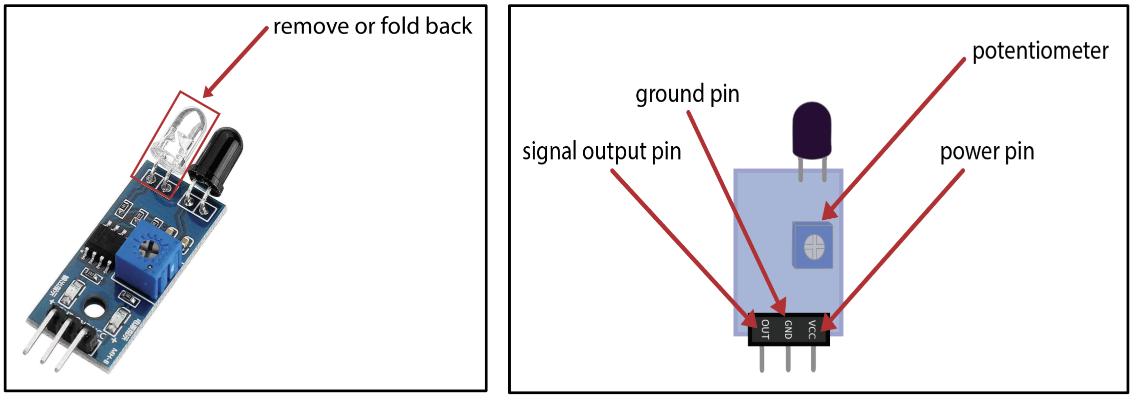

Figure 1. IR photodiodes.

   b. Put together 4 mini breadboards following the orientation of the diagram below (**Fig. 2**).

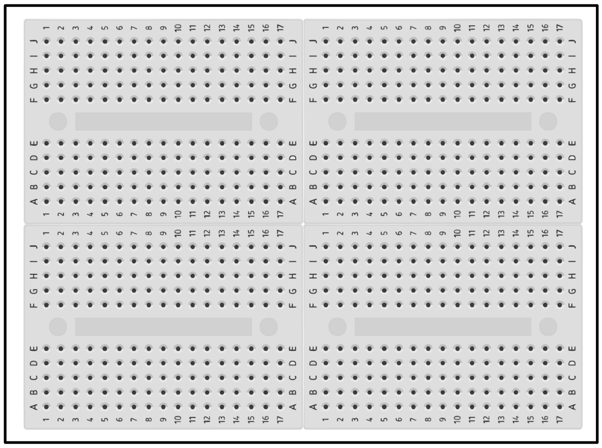

Figure 2. 4 mini breadboards.

c. Place the photodiodes on the mini breadboards as shown in the figure below (**Fig. 3**). It is important to respect the exact positioning as this ensures the photodiodes properly fit into the 3D printed case. **For the middle row, it may be easier to place the wiring first and then the photodiodes after*.

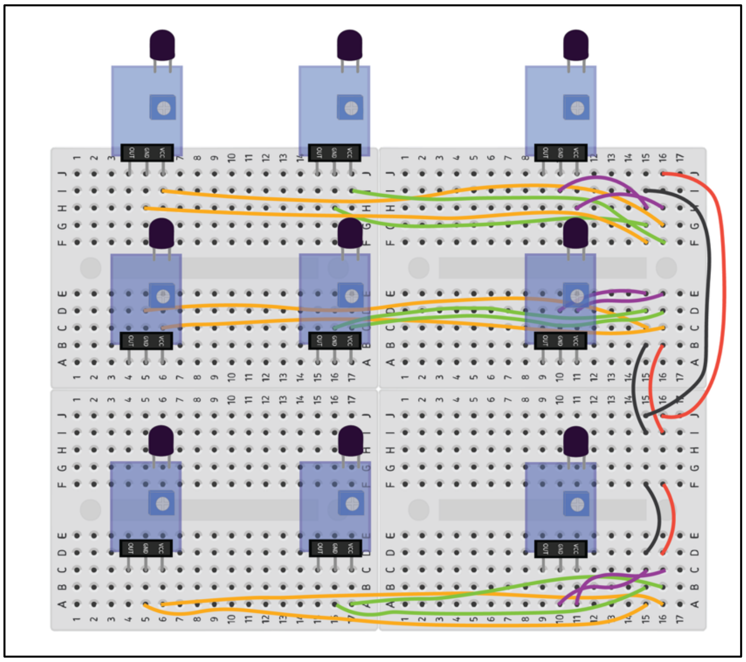

Figure 3: Placement of photodiodes and local wiring on the breadboards.

2. <u>Wiring the LEDs</u> (~90 mins)
   Materials needed: 9 white LEDs, 9 IR LEDs, jumper wires, wire cutters
   a. IR and white LEDs
      i. Put together 2 sets of 4 mini breadboards following the orientation of the diagram below (**Fig. 4**). Ensure that the orientation of the breadboards is exactly like the figure, as the IR and white LEDs will sit on top of each other in the case.
      ii. With the wire cutters, cut the LED leads short enough that the LED plastic case sits flush with the breadboard surface (or no more than 5 mm above the breadboard surface).
      iii. Place the LEDs on the mini breadboards as shown in the figure below. It is important to respect the exact positioning as this ensures that the 3D printed pieces that cover the LED panels fit properly. **Make sure to orientate LEDs with correct polarity (+/-). In* ***Fig. 4****, the white LEDs on the left breadboard have the + pin on the left, whereas the IR LEDs on the right breadboard have the + pin on the right.*

iv. Wire the LEDs as shown in the figure. This is the first part of the wiring and can be done with the mini breadboard panels side by side.

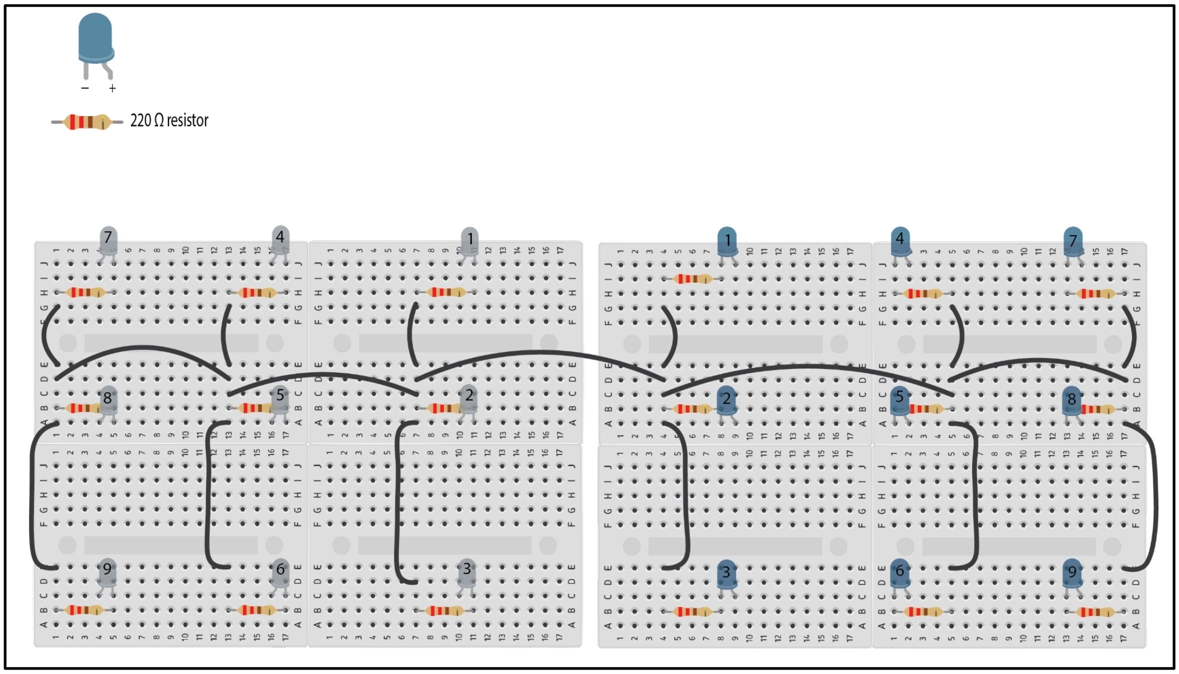

Figure 4. LED wiring. Here, the wiring of the white LEDs is indicated on the left, and the IR LEDs on the right. Note that these are mirror-symmetrical wirings, as these two arrays will be 'folded' to rest one on top of the other, with white LEDs facing upward and IR LEDs facing downward (as per **Fig. 5**).

v. Remove the sticker cover from the base of each mini breadboard. Stick the two panels together so that white LED 1 and IR LED 1 are aligned vertically, with white LEDs pointing up and IR LEDs pointing down (**Fig. 5**).

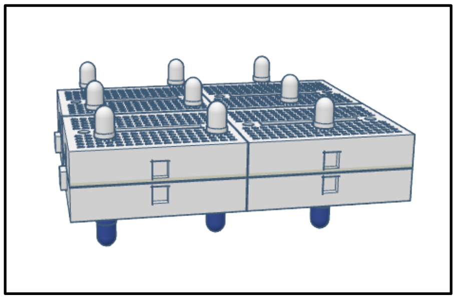

Figure 5. Orientation of white and IR LEDs. Note that, vertically, each white LED has a matching IR LED. *For simplicity, wiring is not shown here (for wiring see **Fig. 4**).

vi. After sticking together the white and IR LED panels, finish wiring the LEDs by adding the wiring shown in green below (**Fig. 6**). The figure below shows the panels side by side for ease of visualization (as a result the green wiring appears to be longer than it actually is). However, the wiring should now be done with the panels on top of one another (this will help ensure stability of the wiring). Note that these wires (indicated in green below) put each white LED on the same electrical circuit as its vertically corresponding IR LED.

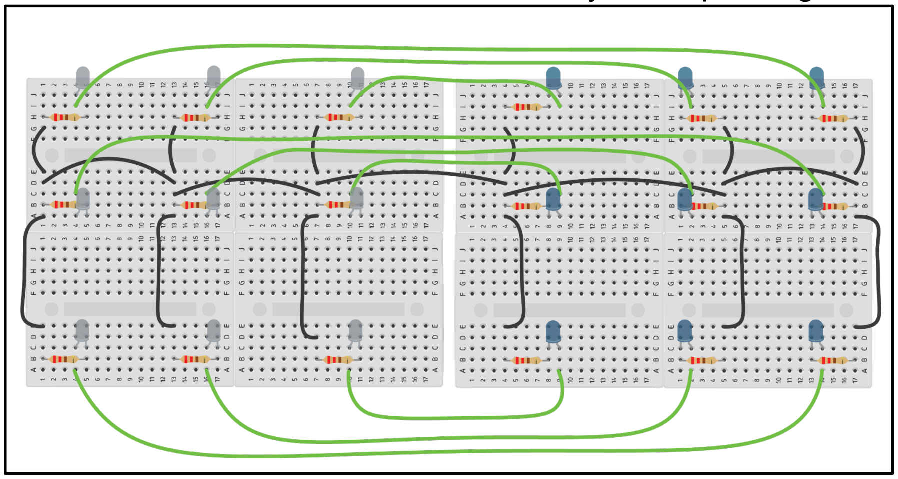

Figure 6. Wiring between the white and IR LED arrays (this should be done with these two arrays vertically stacked on top of one another (as in **Fig. 5**)).

b. Coloured LEDs (representing the output of the 2 retinal ganglion cells)
Materials needed: 1 yellow LED, 1 green LED, 2 mini breadboards

i. Cut the LED pins to a length of between ~10 mm. This is the height the base of the LEDs should sit above the breadboard so that these LEDs fit firmly into their corresponding holes in the 3D printed case.

ii. Place the two LEDs in one of the breadboards as shown in **Fig. 7**. Stack this mini breadboard on top of another mini breadboard. Both breadboards should be facing upward and can be taped together (the bottom breadboard only serves to prop the top breadboard higher, so the LEDs can fit appropriately into the 3D printed case.

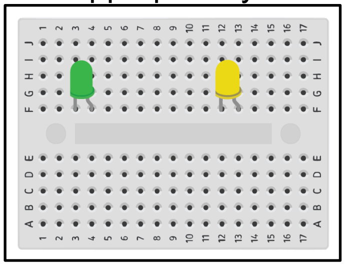

Figure 7. Placement of colored LEDs on a mini breadboard. These LEDs will represent the outputs of retinal ganglion cell 1 (RGC 1, green) and RGC 2 (yellow).

b) CASE ASSEMBLY (~30 mins)
The case must be 3D printed before assembly (.stl files are provided). It was designed in 6 separate parts to enable simple printing and easy assembly (**Figs. 8** and **9**).

Description of the 3D printed parts for the case:
<u>P1 - part 1</u>: This piece is the backbone of the case. It holds the photodiode array, the LED panels, the output pins, and the GPIO interface board.
<u>P2 - part 2</u>: Placed on top of the photodiodes, each photodiode should fit in one of the holes of this piece.
<u>P3 - part 3</u>: Placed underneath the IR LED panel. Each IR LED should fit in one of the holes of this piece.
<u>P4 - part 4</u>: Interfaces with pieces 1-3 and provides structural support and covers the wiring that goes through the middle portion of the case.
<u>P5 - part 5</u>: Placed at the very top of the structure, covering the white LED panel as well as the two coloured LEDs.
<u>P6 - part 6</u>: Covers the C shaped opening on the side of the case. This is the last piece that should be attached. Much of the RetINaBox system testing and troubleshooting stages require P6 to be unattached.

**3D printed pieces will be referred to as P1, P2, and so on throughout the manual*.
**P2 is smaller in length than P3*

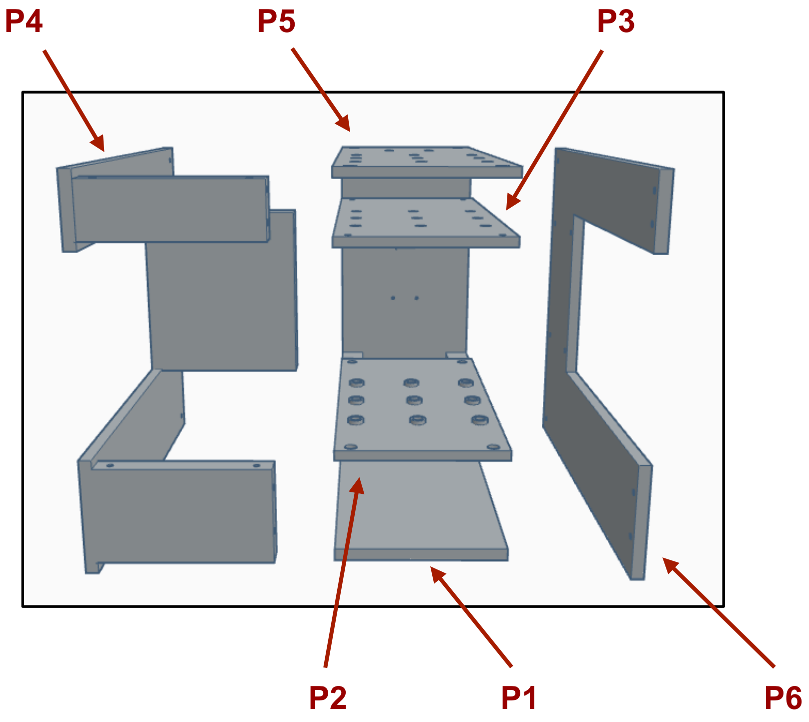

Figure 8. RetINaBox 3D printed pieces.

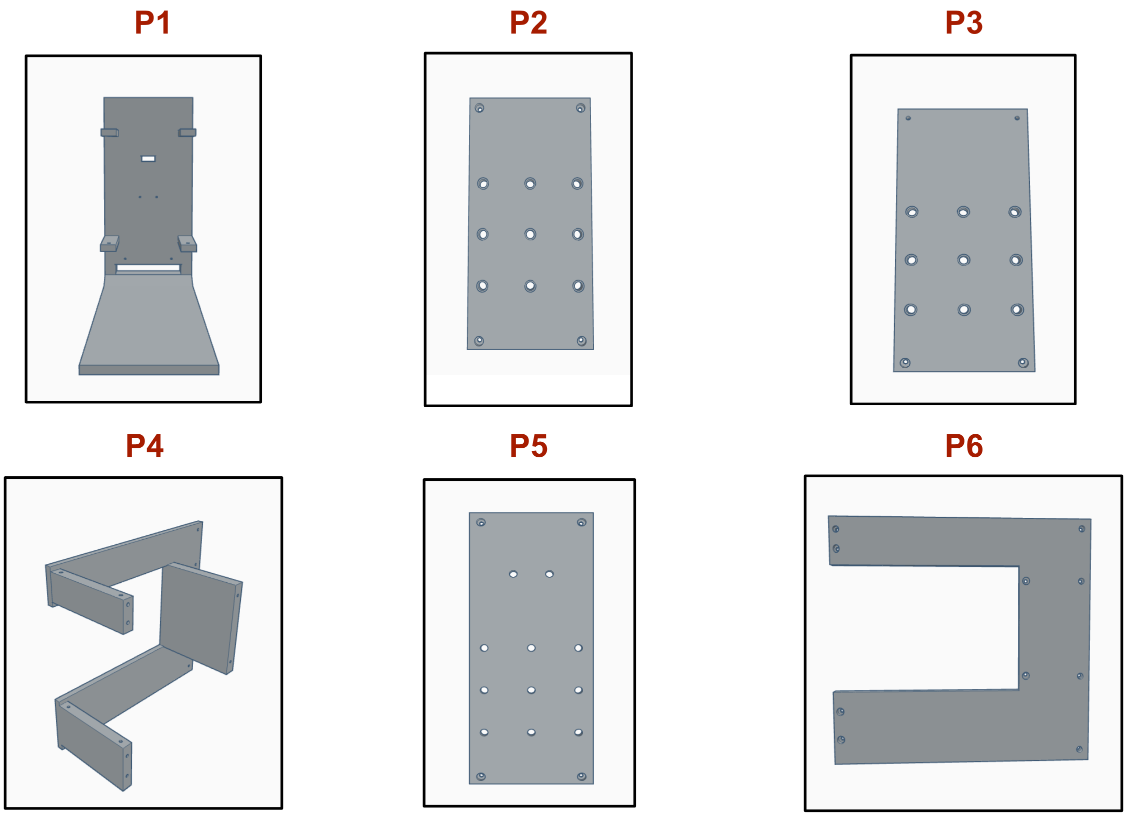

Figure 9. Individual RetINaBox 3D printed pieces.

Here (**Fig. 10**) we show the case fully assembled from a couple viewing angles.

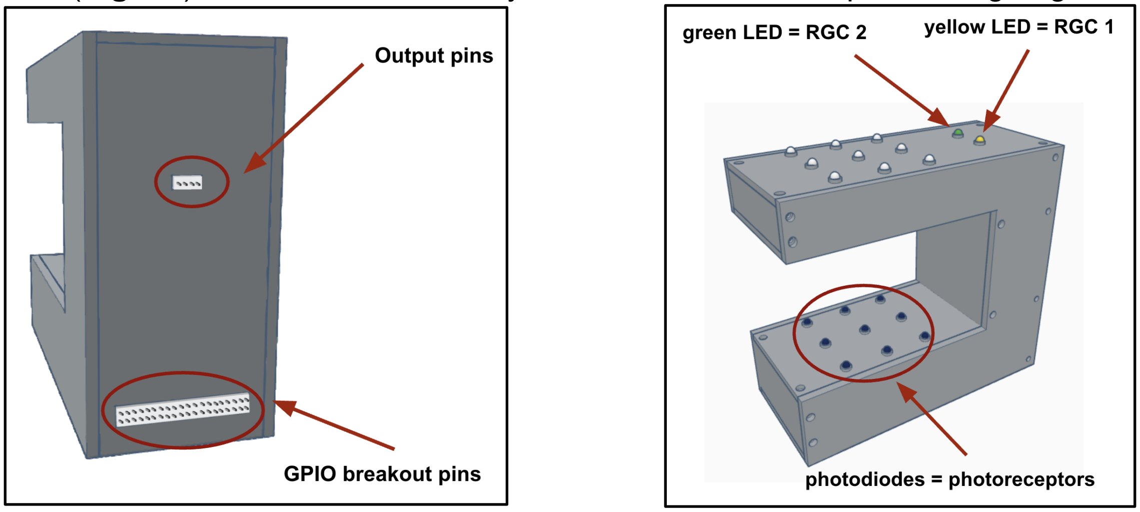

Figure 10. Fully assembled RetINaBox.

c) CONNECTING ELECTRONICS TO Raspberry Pi GPIO (~45 mins)

GPIO (general purpose input/output)

RetINaBox includes a GPIO interface to enable simple connectivity between RetINaBox and the Raspberry Pi with a single cable. Secure the GPIO interface board in the corresponding hole in P1 and attach it using four 6mm M2 screws (**Fig. 11**). **The following steps occur at the same time as Case Assembly*.

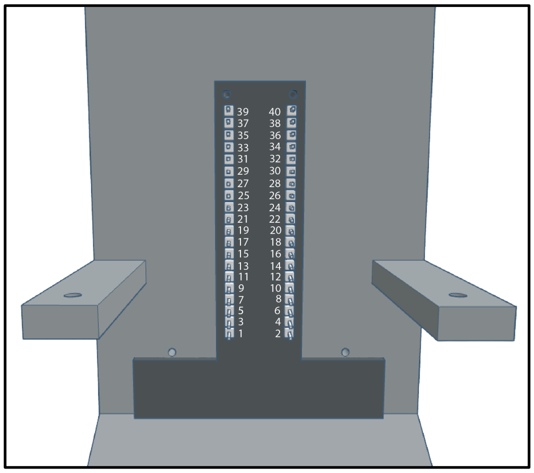

Figure 11. Installation of RetINaBox GPIO adapter.

While shown in more detail below in the subsequent assembly steps, here we provide the complete wiring logic for the RetINaBox electronics (**Fig. 12**). Note that all the wires going from the GPIO board to the LEDs panel should pass through a gap inside the case (**Fig. 13**).

GPIO.setmode(GPIO.BOARD)			
PHOTODIODES GROUND	39	40	IN_PHOTODIODE_9
RGC2_LED	37	38	IN_PHOTODIODE_8
RGC1_LED	35	36	IN_PHOTODIODE_7
OUT_LED_9	33	34	GROUND
OUT_LED_8	31	32	IN_PHOTODIODE_6
OUT_LED_7	29	30	GROUND
	27*	28*	
	25	26	RGC2_3.3V_OUT
OUT_LED_6	23	24	RGC1_3.3V_OUT
OUT_LED_5	21	22	IN_PHOTODIODE_5
OUT_LED_4	19	20	GROUND
3V3 POWER	17	18	IN_PHOTODIODE_4
OUT_LED_3	15	16	IN_PHOTODIODE_3
OUT_LED_2	13	14	GROUND
OUT_LED_1	11	12	IN_PHOTODIODE_2
GROUND (WHITE LEDS PANEL)	9	10	
3V3 POWER	7	8	
	5	6	
	3	4	
	1	2	

photodiodes
white leds
green led
yellow led
out pins
* DO NOT USE!

Figure 12. Connections between GPIO and RetINaBox electronics.

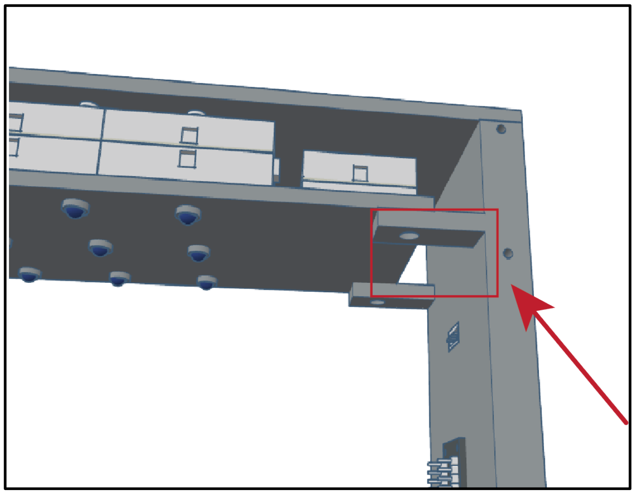

Fig 13. Wiring from the GPIO board to the LED arrays should pass through the internal gap in the case indicated by the red rectangle.

1. Connecting the photodiodes to the GPIO:
   Materials needed: 11 female-to-male dupont wires, 2 x 6mm-long M3 screws
   a. Place the photodiode panel on P1 as indicated in **Fig 14**. Do not remove the plastic cover from the bottom of the mini breadboards, the photodiode panel shouldn't stick to P1. It will get held firmly in place by P2.

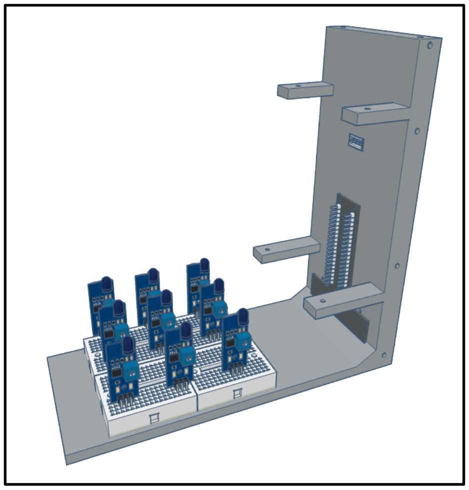

Figure 14. Placement of the photodiode containing breadboards onto part P1.

b. Use female-to-male dupont wires to connect the signal output pin of each photodiode to the GPIO, using connectivity instructions listed below (**Fig. 15**). Each number in **Fig. 15** corresponds to a GPIO pin (**Figs. 11** & **12**).

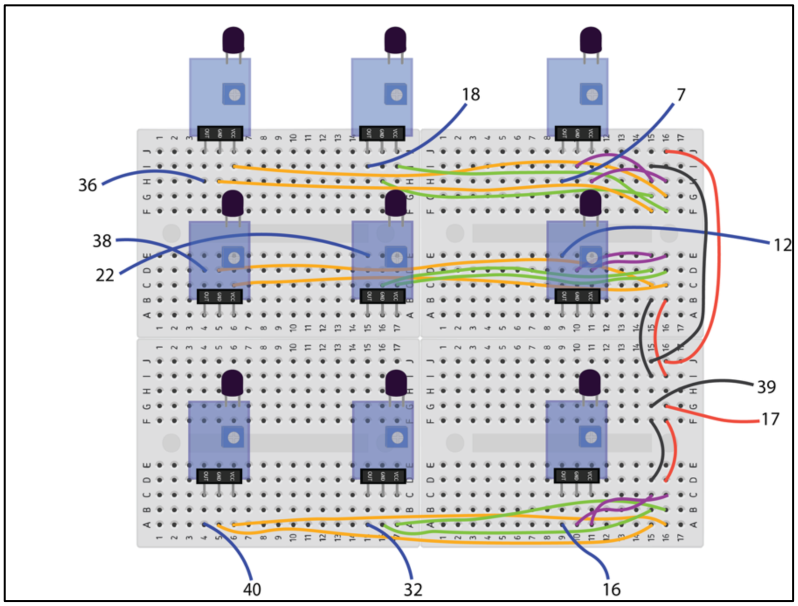

Figure 15. Logic for connecting photodiodes to the GPIO board.

c. Attach part P2 on top of the photodiodes. Carefully fit all 9 photodiodes into the holes in P2. Then use 2x6mm long M3 screws to secure the piece in place (**Fig. 16**). **Do not tighten it fully yet*; *P2 should be orientated with the raised LED guides (i.e. the bumps at each of the LED holes) facing up.*

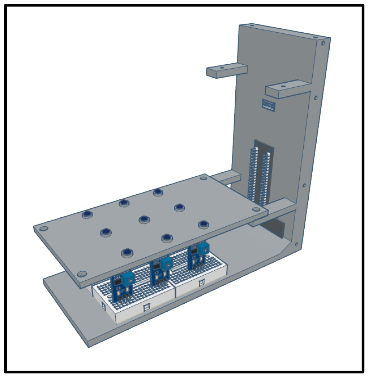

Figure 16. Assembly progress, with P1 and P2, the photodiodes and the GPIO interface board.

2. Connecting the 3.3V output pins (which are RGC digital outputs) to the GPIO:

   Materials needed: 4 female-to-female dupont wires, 4 wires from the Connector Pre-Crimped Cable Kit (see Appendix), electrical tape

   a. Put together a 4-pin connector (4 pins stick out on each side) and socket from the Connector Pre-Crimped Cable Kit. This allows wires to be connected on both sides. One side should have visible pins sticking out. On the other side, the pins are covered by the socket.
   b. Place it in the middle hole of P1 as shown in **Fig. 17**. The side that has the pins covered by the socket should face the interior of the case. **The socket should be on the inside part of the case and the pins on the outside*. *The socket connector should fit tightly into the hole in the case.*
   c. Attach wires from the kit to the 4 socket pins on the inside of the RetINaBox case (**Fig. 17**).
   d. Daisy chain these wires with female-to-female dupont wires (use electrical tape to secure this connection) and connect to the corresponding GPIO pins (according to the logic in **Fig. 17**).

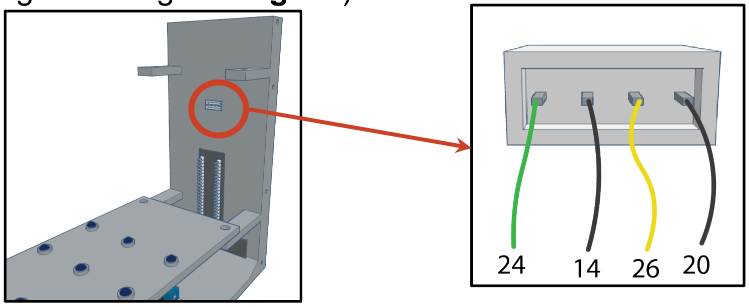

Figure 17. Connecting the 3.3V RGC output pins to the GPIO. Note that the socket is not shown in the connectivity cartoon.

3. Connecting the buzzer:

   Materials needed: 1 mini breadboard, 10k resistor, PN2222 transistor, Elegoo active buzzer, 1 jumper wire, 3 wires from the Connector Pre-Crimped Cable Kit, 3 M-F dupont wires

   a. On a mini breadboard, place the buzzer, the transistor, the 10K ohm resistor and the jumper wire as shown in **Fig. 18**.

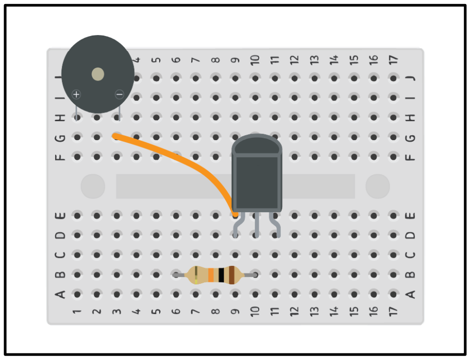

Figure 18. Placement of buzzer, transistor, jumper wire between transistor and buzzer, and resistor.

b. Wire the components as shown in the **Fig. 19**. First connect 3 wires from the Connector Pre-Crimped Cable Kit to the corresponding output pins, then, daisy chain these wires with female-to-male dupont wires (use electrical tape to secure these connections). Note that the buzzer is connected to the GPIO breakout board through the output pins that face the back of the box (refer to **Figs. 10, 12 and 17**). With this circuit, the buzzer will only sound when both pin 24 and 26, which correspond to RGC1 and RGC2, send out 3.3V at the same time, which happens when RGC1 and RGC2 are co-activated.

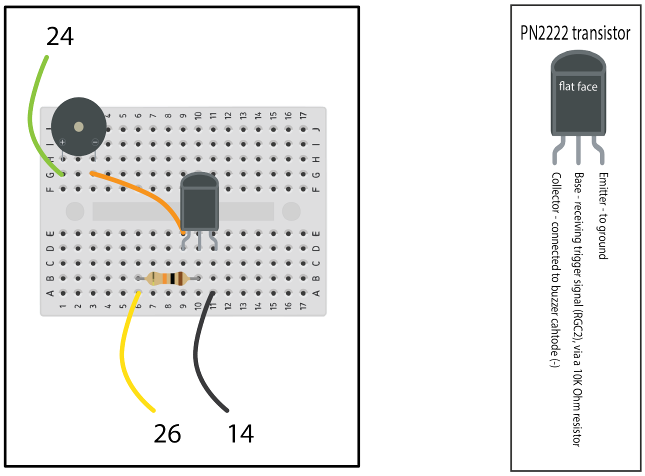

Figure 19. Circuit wiring of the buzzer (related to Lesson Plan 2).

4. Connecting the LEDs to the GPIO board:

Materials needed: electrical wire, wire cutter, 10 female-to-female dupont wires, 2x 6mm-long M3 screws

a. Attach part P3 with 2 x 6mm-long M3 screws (**Fig. 20**). **P3 should be orientated with the raised LED guides (i.e. the bumps at each of the LED holes) facing down.*

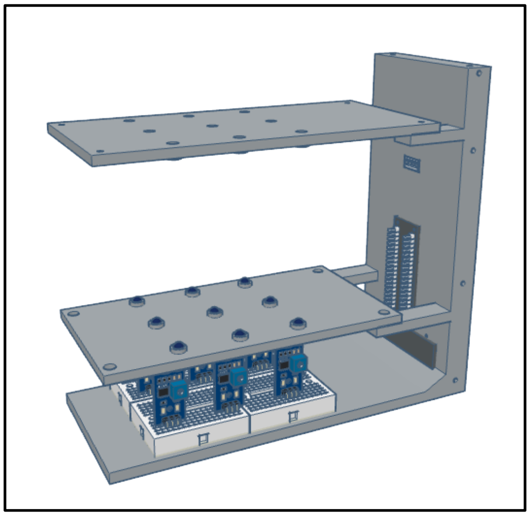

Figure 20. Attaching part P3.

b. Place breadboards containing LEDs onto P3 (**Fig. 21**; wiring is not shown here for ease of visualization). The bottom breadboard should not be taped to P3. These LEDs will be held in place once P5 is added. **Follow steps c) and d) before completing b)*.

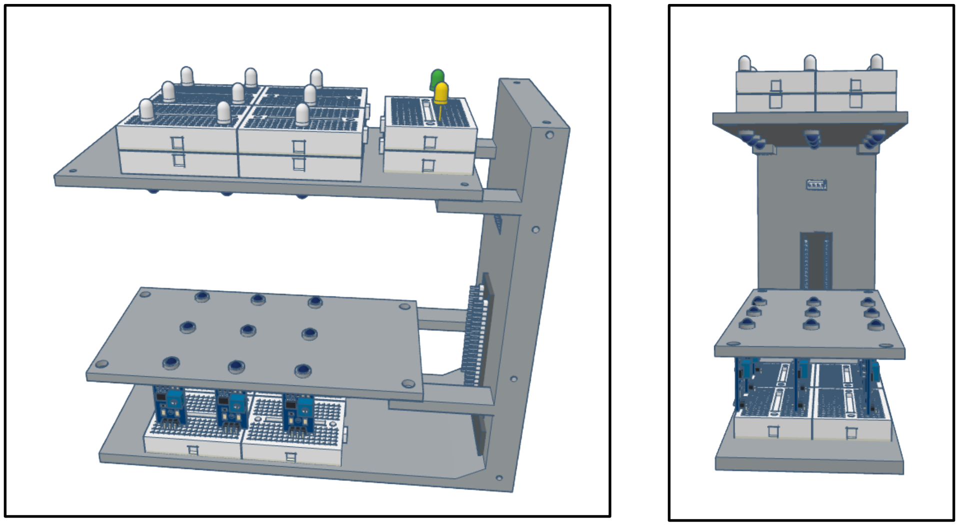

Figure 21. Attaching LEDs onto part P3.

c. Connect each of the 9 white+IR LED pairs to the GPIO (via the 9 orange wires (+) and black wires (-, GND; black hook-up wires) in **Fig. 22**. For the orange wires (orange here is for visual display—in reality, here we used the red hook-up wire), cut the electrical wire to an appropriate length (~5cm) and daisy chain it to a female-to-female dupont wire, which will then connect to the GPIO board (according to the logic in **Fig. 22**). **Hook-up electric wire (as opposed to dupont wires) is used here due to the limited space between the LEDs and the 3D printed pieces that these LEDs arrays will fit between.*

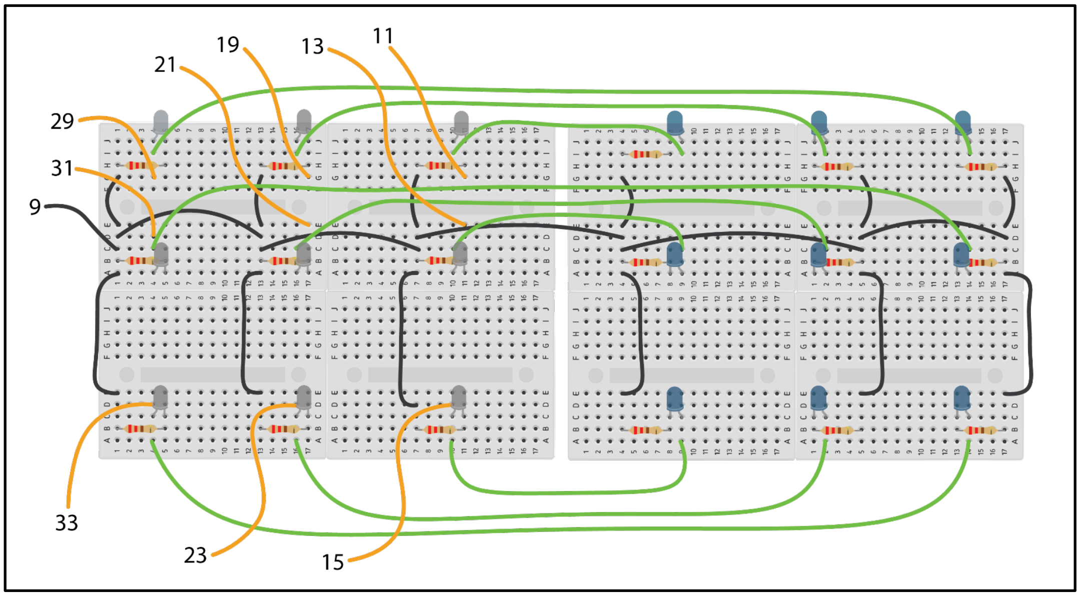

Figure 22. Connecting white and IR LEDs to the GPIO.

d. Connect the two color (green and yellow) LEDs, which represent the outputs of ganglion cells 1 and 2, to the GPIO, using the following logic (**Fig. 23**). Similarly to the step above, red hook-up wire is also used here and daisy chained to female-to female dupont wires.

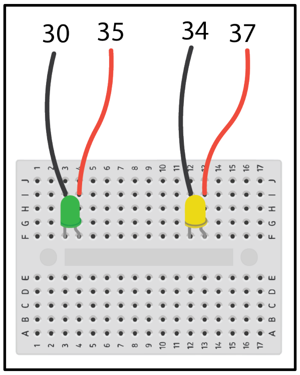

Figure 23. Connecting green and yellow LEDs to the GPIO.

e. Attach part P4 by connecting it to P2 and P3 (**Fig. 24**). This will stabilize the case and facilitate testing. Attaching parts P5 and P6, which will close up RetINaBox, should be performed later, once RetINaBox has been connected to the Raspberry Pi and the electronics are tested and the photodiode sensitivities optimized (outlined below).

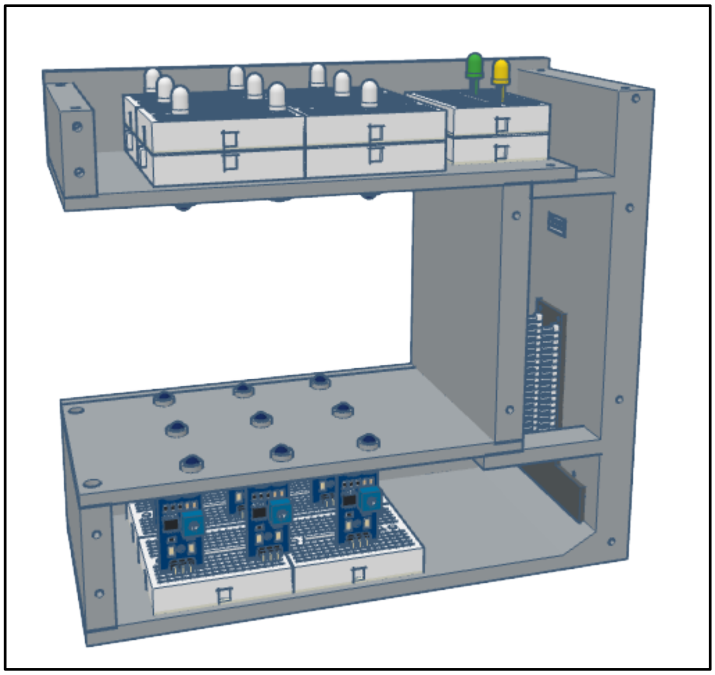

Figure 24. Attaching part P4.

3. GENERATING VISUAL STIMULI (~15 mins)

While visual stimuli can be generated directly with the Visual Stimulus Controller within RetINaBox's GUI. However, to better mimic hands-on visual neuroscience experiments, users can turn all of RetINaBox's stimulus LEDs and put real-world shapes in between RetINaBox's LEDs and photodiodes (i.e. block out light in specific patterns) to make visual stimuli and test feature selectivity of the model RGCs. Here we provide a couple methods for generating visual stimuli to test RetINaBox with.

a) Visual Stimulus Tool

Materials Needed: clear plastic sheet, permanent marker, modeling clay, ruler (see Appendix)

To delivery various patterns of visual stimuli to RetINaBox, we recommend that users create a Visual Stimulus Tool. To do this, take the clear plastic sheet and use a fine-tipped permanent marker and a ruler to draw a grid covering the entire plastic sheet (**Fig. 25**). Each square in the grid should be 3 cm by 3 cm wide, roughly corresponding to the receptive field area of a single RetINaBox photodiode.

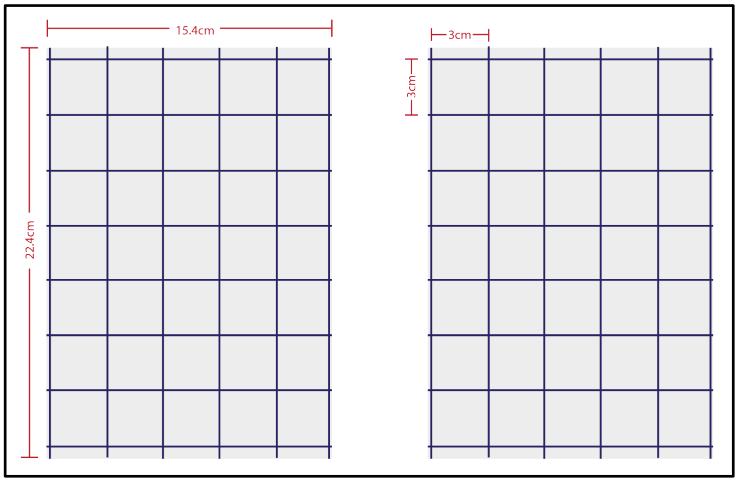

Figure 25. Visual Stimulus Tool with grids for aligning stimuli with the model photoreceptor array.

Next, place modeling clay over the plastic sheet. Leave specific regions without clay in order to make your visual stimulus (i.e. light passing/being block out of specific regions is your stimulus). You can use the clay to create differently shaped visual stimuli to present to RetINaBox (**Fig. 26**).

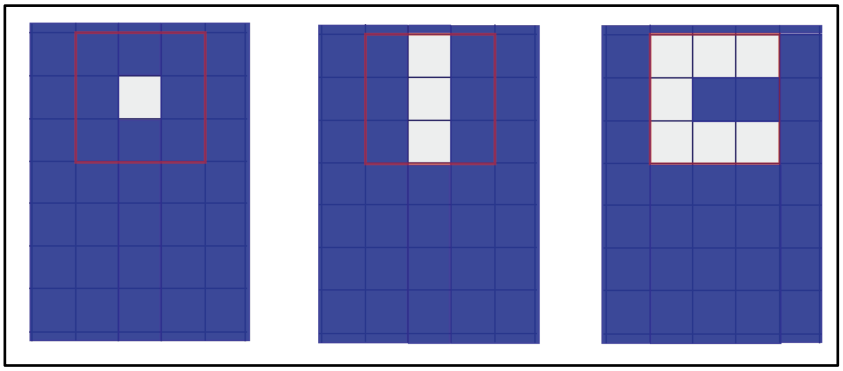

Figure 26. Example visual stimuli. The red highlighted region indicates the size of RetINaBox's field of view.

The Visual Stimulus Tool works by controlling which model photoreceptors (i.e photodiodes) are activated by letting light pass from the stimulus LEDs to the photodiodes (**Fig. 27**).

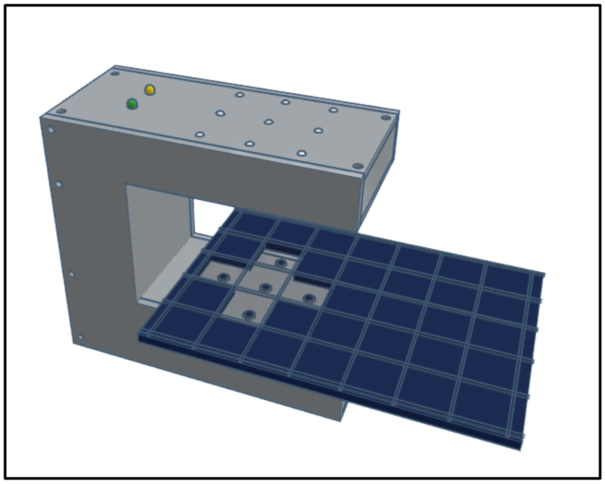

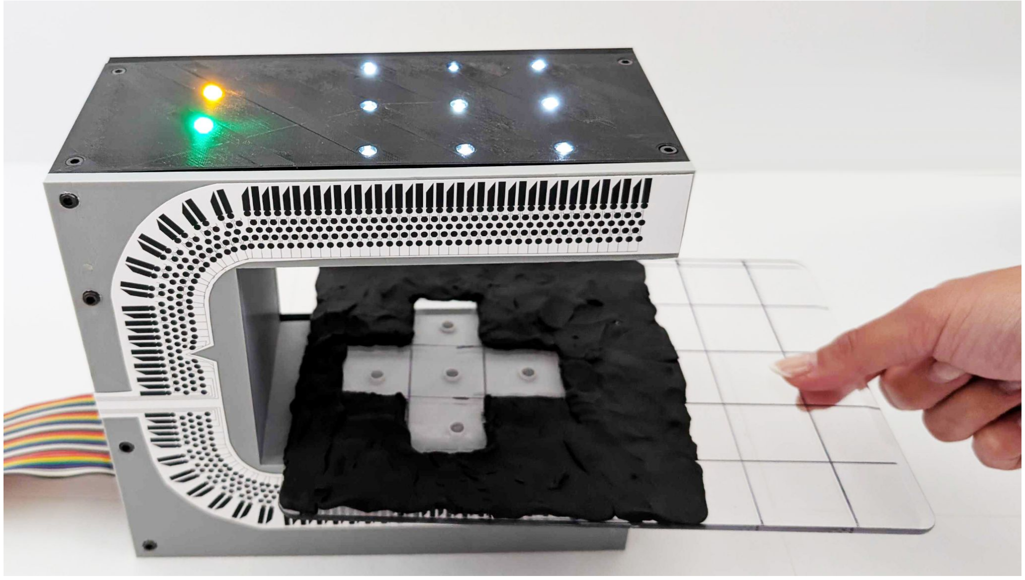

Figure 27. Visual Stimulus Tool in action (this example is related to Lesson 2).

b) Visual stimulation with shapes cut out of paper/cardboard

While the Visual Stimulus Tool is a great option as it allows users to make essentially any shape, in either ON or OFF versions, it can take a bit of time to switch between different stimuli. As an alternative method for more quickly testing out visual responses in RetINaBox, users can cut shapes out of paper/cardboard. To get started, users can access a PDF document containing a set of shape templates that can be printed and cutout here. These shapes, as shown in **Fig. 28**, can be used to work through many of the lesson plan activities. Users can also generate other shapes as they see fit.

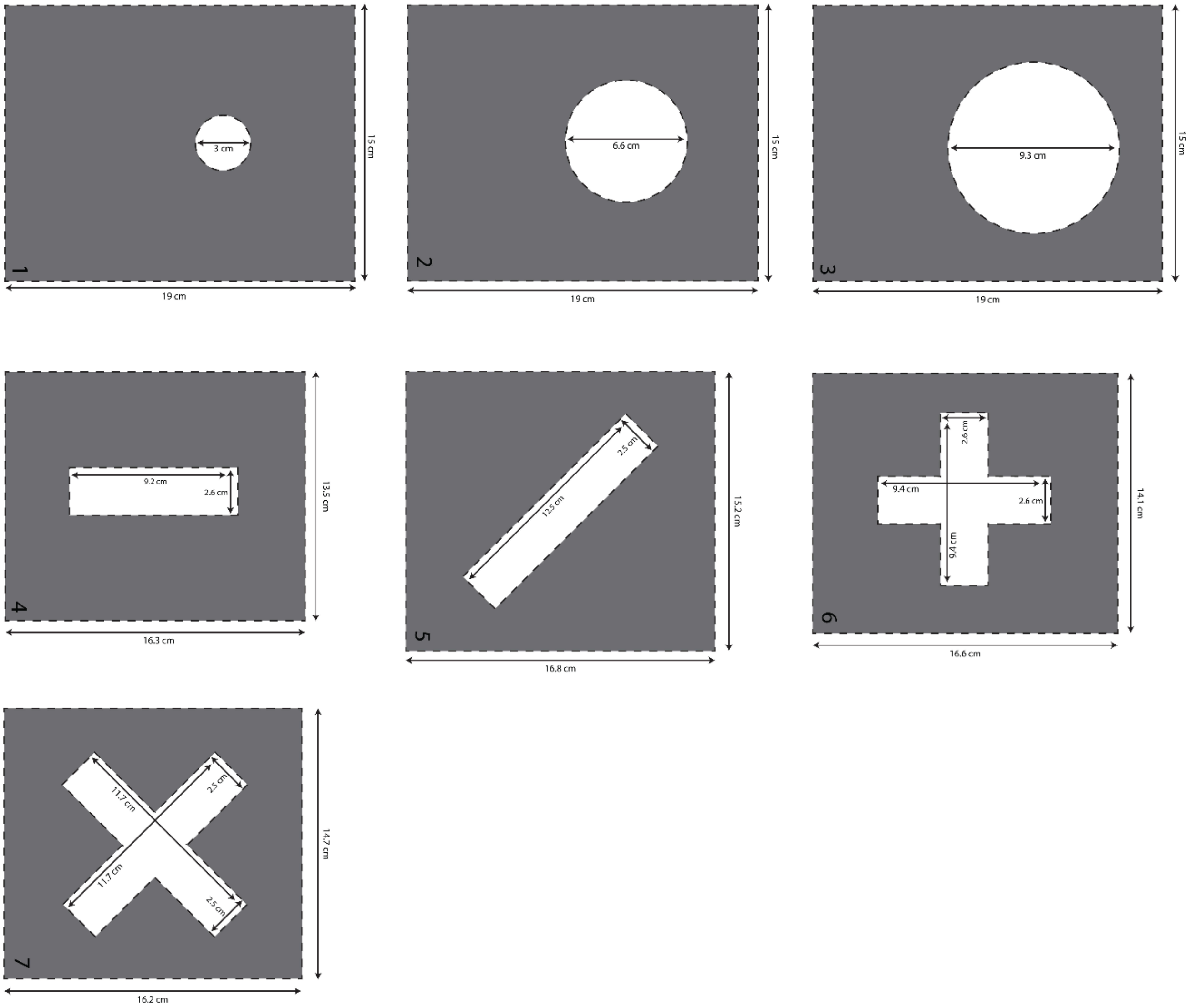

Figure 28. Visual stimulus shapes that can be printed and cutout from paper/cardboard.

4. INSTALLING THE SOFTWARE (~5 mins)

a. On your Raspberry Pi, use the web browser to access the Trenholm Lab RetINaBox GitHub page: https://github.com/Trenholm-Lab/RetINaBox. **We strongly recommend using a Raspberry Pi 500 (or Raspberry Pi 5). We tested the software on a Pi 400, and though it works if you deal with some compatibility hiccups, it is a rather laggy.*

b. Download the repository ZIP by clicking on the green ‘Code’ button, then the ‘Download ZIP’ button (**Fig. 29**). Once downloaded, the ZIP file will appear in your downloads folder as RetINaBox-main.zip.

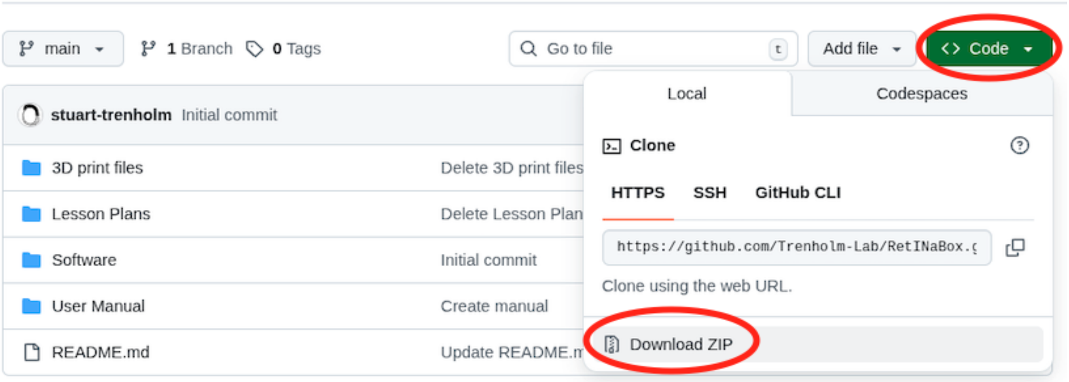

Figure 29. Downloading RetINaBox software from GitHub.

c. Unzip the code files to your device by right-clicking on the RetINaBox-main.zip file and selecting ‘Extract To…’. Select your Desktop as the ‘Extract to:’ save path and leave all other options untouched as outlined below in **Fig. 30**. After this step, your Desktop will now contain a folder titled RetINaBox-main. Do not modify or move this folder. You can now close the File Manager.

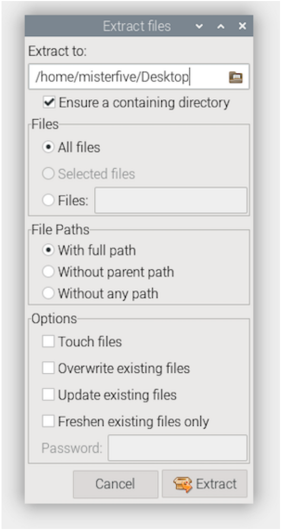

Figure 30. Raspberry Pi Extract Files Menu.

d. Open the terminal and type the following commands followed by the enter key as shown in **Fig. 31**.

```
cd Desktop/RetINaBox-main/Software/RetINaBox
chmod +x install_RetINaBox.py
python3 install_RetINaBox.py
```

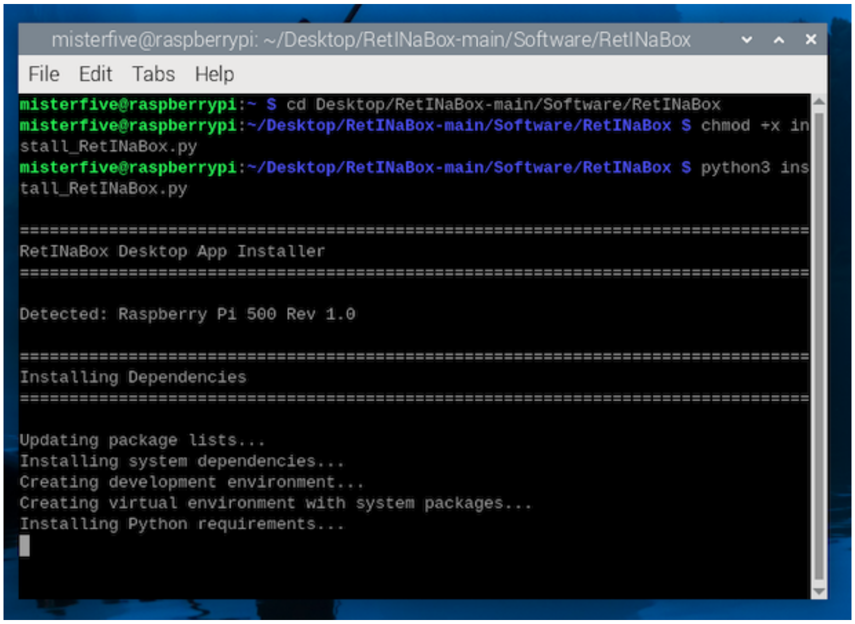

Figure 31. Terminal during RetINaBox code installation.

e. Following the successful installation of RetINaBox, you will see a new app on your Desktop. To access the GUI, double click on the new RetINaBox Desktop app and click on Execute (**Fig. 32**). Welcome to the RetINaBox Lab!
   i. Should installation not be successful, please refer to the User Manual section A2: Appendix 2: Troubleshooting.

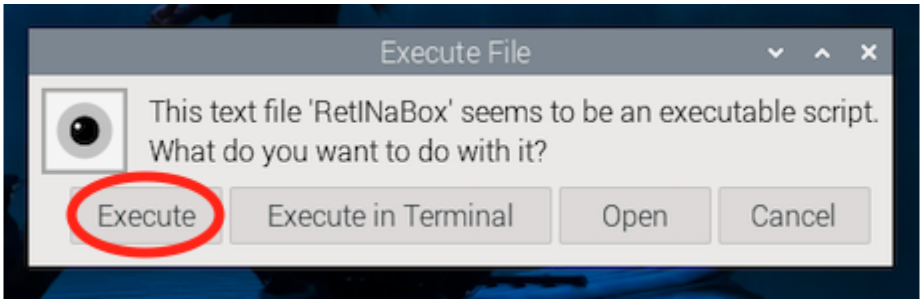

Figure 32. RetINaBox Execute File Message.

5. CONNECTING RetINaBox TO THE Raspberry Pi

a. Connect RetINaBox to the Raspberry Pi using the GPIO rainbow connection cable. ***IMPORTANT: the grey connectors at both ends of the rainbow connection cable have a small raised plastic notch on one side of the connector's horizontal axis - it is very important to connect these appropriately to the RetINaBox and Raspberry Pi, otherwise the connections between RetINaBox and Raspberry Pi will be incorrect and the Raspberry Pi or electronic elements could get burned out! The sockets in both the RetINaBox and Raspberry Pi have little notches cut out, indicating the proper orientation that the rainbow connection cable should be attached!***

b. Connect the Raspberry Pi to power (i.e. plug in the Raspberry Pi). We recommend using an extender cable for the rainbow connection cable, which doubles its length and makes it easier to orient the RetINaBox next to the Raspberry Pi 500.

c. If the photodiodes have been connected correctly, the right-most red LED on each of their circuit boards should light up (**Fig. 33**), indicating they are receiving power.

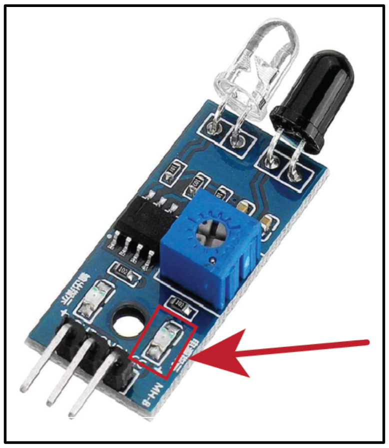

Figure 33. Powering ON the Raspberry Pi should result in power going to all 9 photodiodes, which should turn on the right-most red LED on each photodiode circuit board.

d. The next step is to adjust the sensitivity of the IR photodiodes such that they are only activated when the IR LEDs are turned on. First, take a small screwdriver, and for each photodiode turn the potentiometer in a clockwise manner until the leftmost LED lights up (**Fig. 34**). Then turn the potentiometer roughly a half turn back counterclockwise so that the leftmost LED turns off. Next, open the RetINaBox software (see Section 6 below) and turn on all 9 LEDs. If the LEDs have been connected correctly, the 9 white LEDs on the top of RetINaBox should now light up (visible by eye), and the 9 IR LEDs should also light up, but not be visible by eye. Turning the LEDs on should activate the photodiodes and turn on the leftmost LED on each photodiode. If this doesn't happen, adjust the photodiode sensitivity with the potentiometer until the leftmost red LED only turns on when the IR LED directly above that photodiode is turned on (**Fig. 34**). If a photodiode is powered but you are unable to activate by turning on the LEDs, likely the IR LED is not correctly wired (see Troubleshooting section).

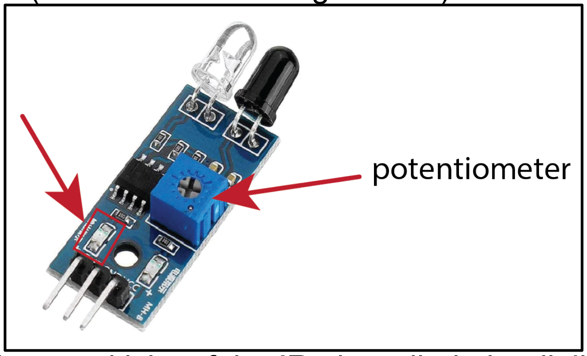

Figure 34. Adjust the sensitivity of the IR photodiode by dialing the potentiometer back and forth until the leftmost red LED on the circuit board only turns on when the IR LEDs are turned on in the RetINaBox software.

e. Attach parts P5 and P6 (**Fig. 35**). You have now finished building RetINaBox!

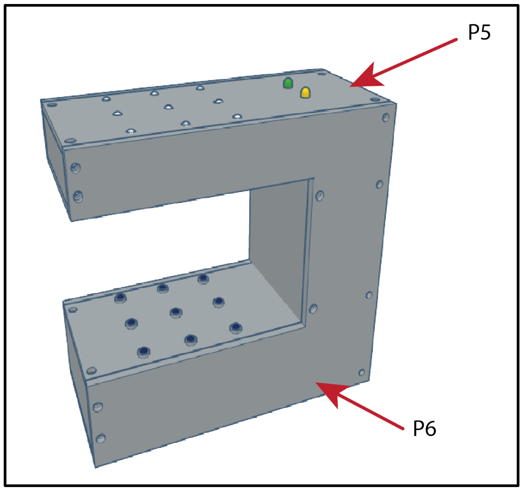

Figure 35. Completed RetINaBox

6. USING THE SOFTWARE

a. Double click on the "RetINaBox" logo on the desktop, or in Visual Studio Code, run the file "main.py"

b. This will open the welcome screen (**Fig. 36**). Select "Enter the Lab" to start working with RetINaBox. You can also access the user manual and lesson plans.

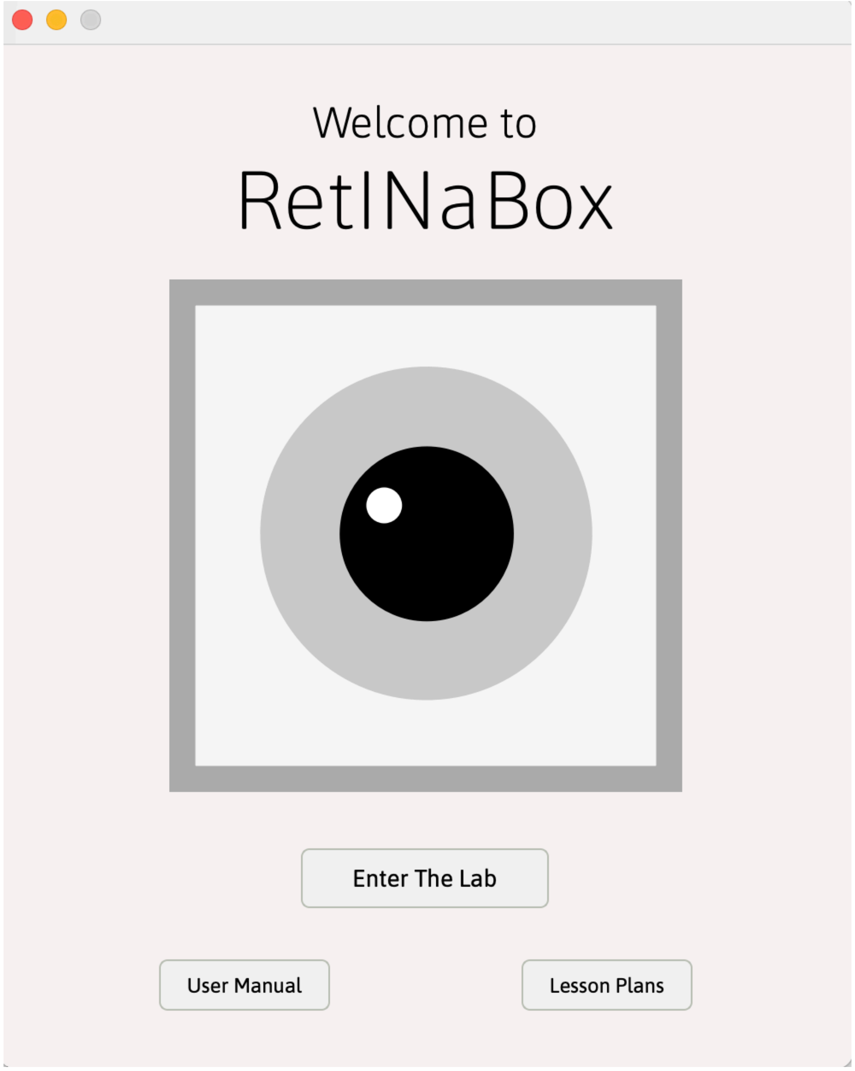

Figure 36. RetINaBox welcome screen.

c. You will now see the main RetINaBox graphical user interface (GUI; **Fig. 37**). The software contains 4 menu items (the GUI can be maximized to full screen):
   i. User Manual: Opens a PDF of the user manual
   ii. Lessons: Here you can access the lesson plans. Also, for each of the 4 lessons you will find preset RetINaBox settings, and lesson-specific challenges.
   iii. Save: Click this to save your current RetINaBox settings.
   iv. Open: Click here to open previously saved RetINaBox settings.

The RetINaBox GUI is split into 3 sections (we'll go into each of these in more detail next):

Visual Stimulus Controller: This is where you control RetINaBox's stimulus LEDs

Connectivity Manager: This is where you control the connectivity of the model photoreceptors to the model ganglion cells.

Signal Monitor: This shows you when photoreceptors are active, what signal they're passing to the ganglion cells, and when the ganglion cells are active.

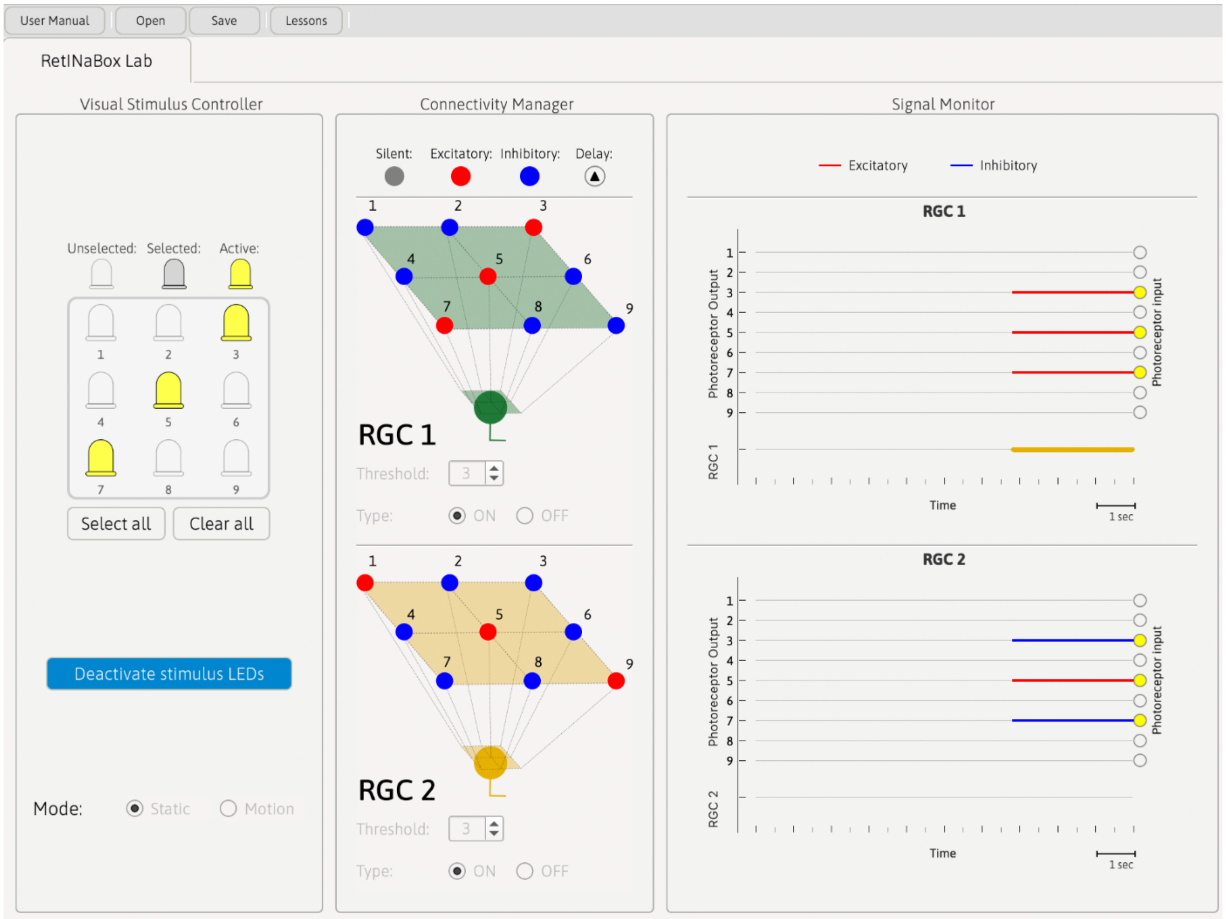

Figure 37. The main RetINaBox graphical user interface.

d. Visual Stimulus Controller (**Fig. 38**).

   i. At the top, you'll see the 3 x 3 LED array. Before turning on the LEDs, you'll need to activate (by clicking on them) the LEDs you want to turn on. This will turn them grey in the display. There are also "Select all" and "Clear all" buttons.

   ii. Next, you can decide if you want to present a static (still) stimulus, or if you want it to move leftward or rightward. To start with, leave it in Static mode

   iii. Now, click the "Activate stimulus LEDs" button. You'll see in the display that the LEDs you selected are now active (i.e. yellow). If you're connected to the RetINaBox, you'll also see the LEDs you selected turn on. The LED numbering in the software corresponds to when you're directly looking at the front of RetINaBox.

   iv. Related to the direction selectivity lesson (lesson 3), you may wish to have your visual stimuli move leftward or rightward. To do this, simply activate a pattern of LEDs, select Motion mode, select direction (left or right arrow), and select speed (slow, medium, or fast).

   v. Lastly, for all the lessons in the lesson plan, at some point you'll want to test real-world stimuli, using the Stimulus Presenter Tool, the paper cutouts, or your hands. To do this, activate all 9 LEDs, select Static Mode, and turn the LEDs on.

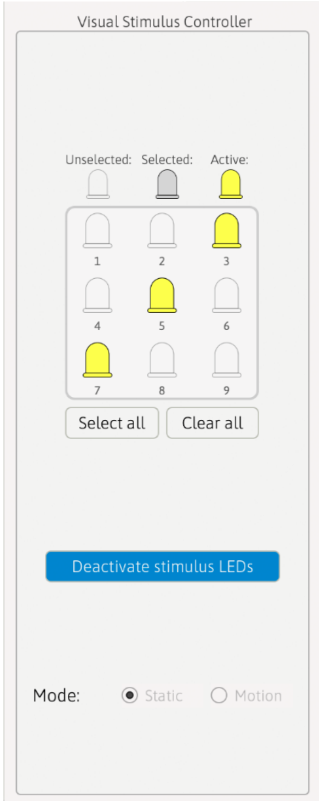

Figure 38. The Visual Stimulus Controller.

e. Connectivity Manager (**Fig. 39**).

i. The Connectivity Manager allows users to set the connectivity between each of the 9 model photoreceptors (i.e. the photodiodes) and 2 different model retinal ganglion cells (RGCs).

ii. Independently for each of the 2 RGCs, each photoreceptor can be set as:

1. Silent: it provides no signal to the ganglion cell
2. Excitatory: when activated, this photoreceptor provides a signal of +1 to the ganglion cell
3. Inhibitory: when activated, this photoreceptor provides a signal of -1 to the ganglion cell
4. Delay: you can add a time delay between when the photoreceptor is activated and when it sends a signal to the ganglion cell. The delay options are: none, short, medium, and long. These delays can be helpful for generating direction selective responses and are meant to help model temporally asymmetric circuit connectivity.

iii. To change the connectivity to one of the ganglion cells, simply click on its circuit (**Fig. 39**, *left*). This will open a pop-up window (**Fig. 39**, *right*) where you can change the settings for each model photoreceptor.

iv. Set the RGC's Threshold. At each point in the time, the RGC will sum up all its photoreceptor inputs, and it needs to meet this threshold to become activated.

v. Set the RGC Type as ON or OFF. For ON RGCs, photoreceptors are considered activated when the photodiode detects light. For OFF RGCs, photoreceptors are considered activated when the photodiode does not detect light.

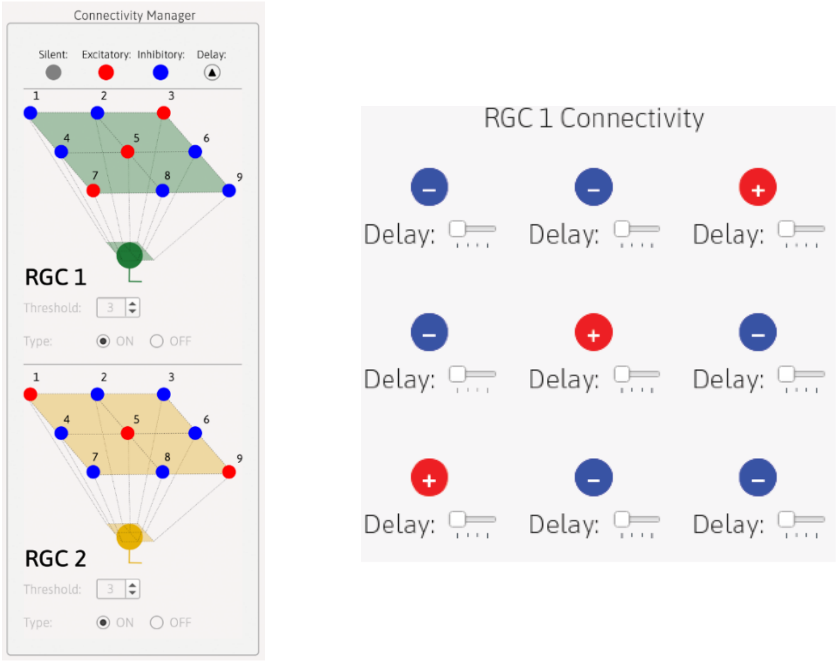

Figure 39. Connectivity Manager in main GUI (*left*) and its pop-up window (*right*).

f. Signal Monitor (**Fig. 40**).
   i. The Signal Monitor provides a real-time readout of:
      1. Which model photoreceptors (photodiodes) are detecting light. This can be seen when the circles on the right-hand side turn yellow.
      2. The polarity of the signal being sent, either excitatory (red) or inhibitory (blue), by each activated model photoreceptor to each retinal ganglion cell.
      3. When each ganglion cell (RGC1, *top*; RGC2, *bottom*) is activated. This occurs when the sum of all its photoreceptor inputs meets or exceeds the threshold set in the Connectivity Manager.

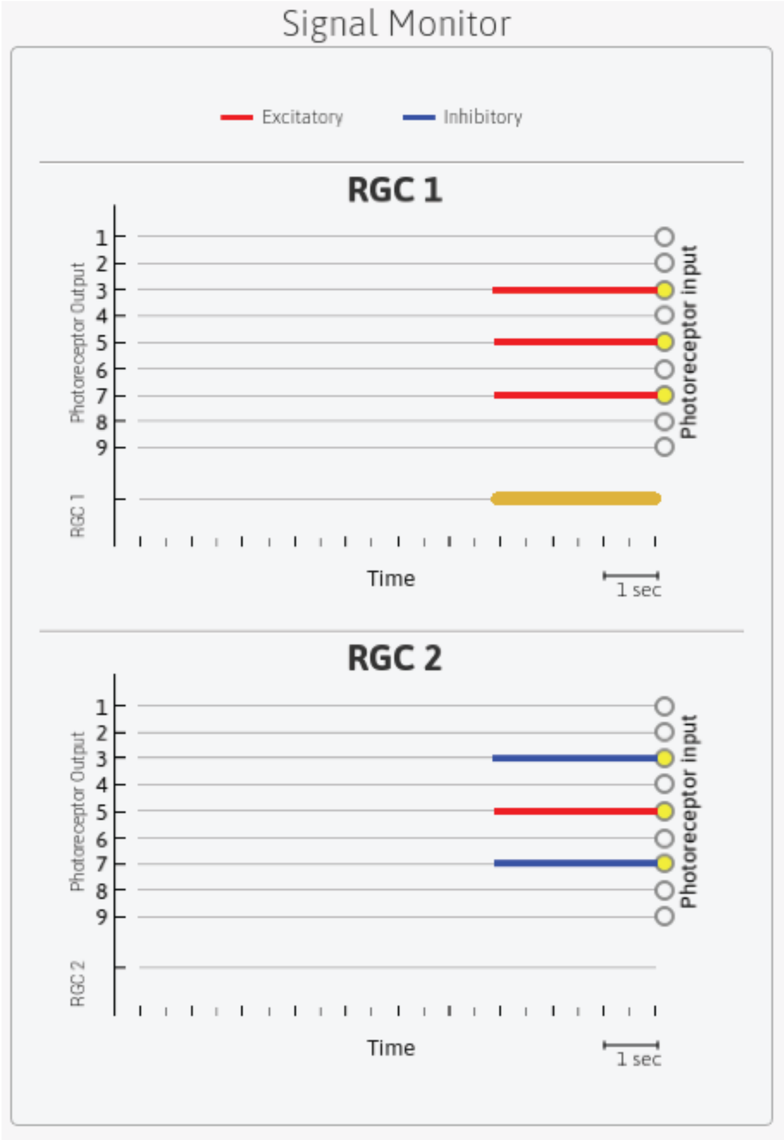

Figure 40. The Signal Monitor.

g. Additional software features

i. Code breaking activity:

1. In Lesson 2, users are tasked with using center-surround receptive fields to crack codes. Accessed via the Lessons tab in the menu bar (Lessons > Lesson 2 > Code Breaker), selecting the Code Breaker activity will load a new tab (**Fig. 41**).
2. Using cipher information, users must toggle back to the main RetINaBox GUI and set the Connectivity Manager so that RGC1 and RGC2 exhibit preferred visual stimuli as outlined in the cipher (while ensuring that RGC1 and RGC2 can be co-activated).
3. Next, users should use the Visual Stimulus Tool to present stimuli corresponding to each of the letters in the code, and, referencing the decoder in the cipher, enter the correct letter in the codebreaker for each stimulus code.
4. Once users have solved the code, they can check their answer.

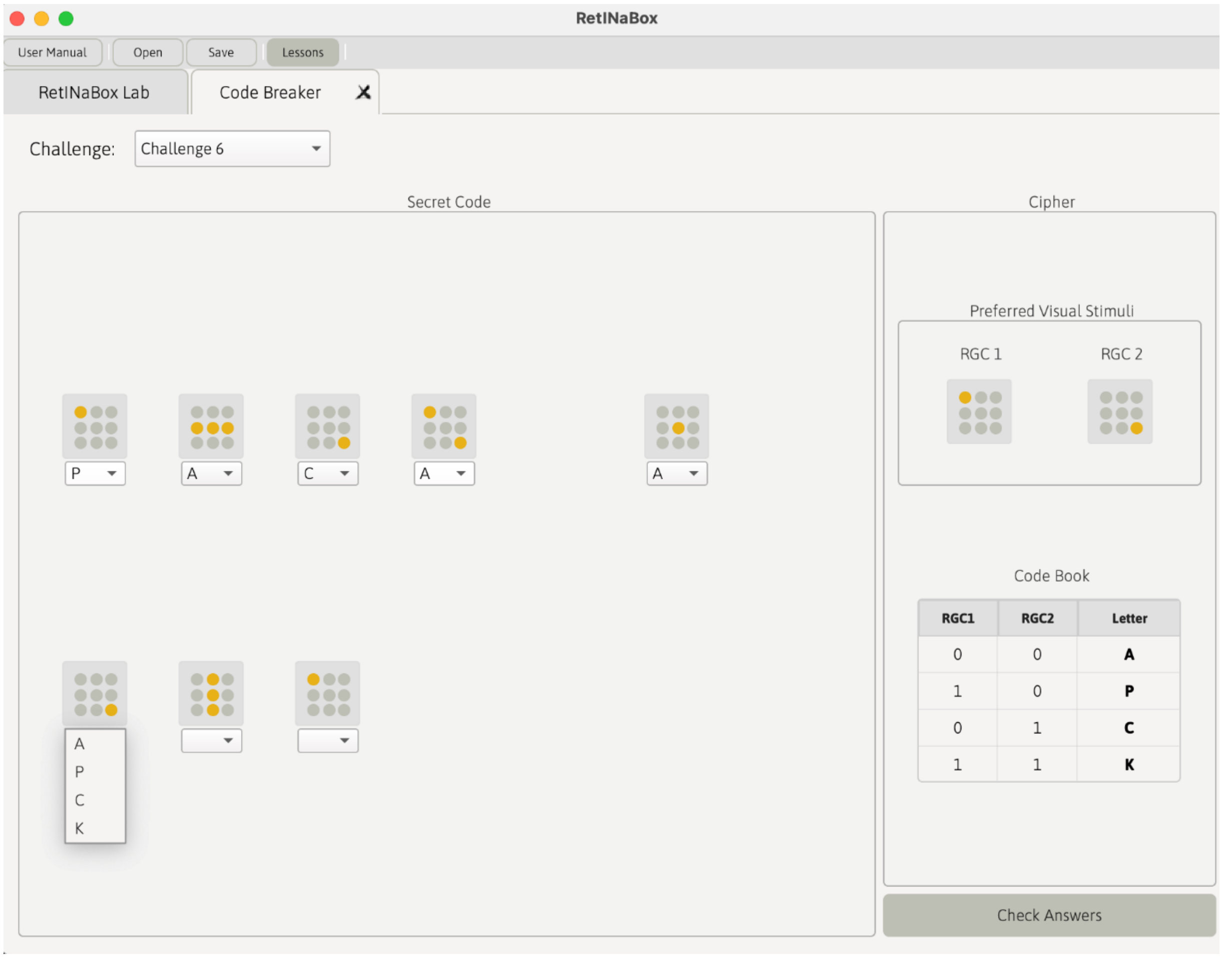

Figure 41. Code breaking activity related to Lesson 2.

ii. Block breaker video game:

1. In Lesson 3, after generating direction selective ganglion cells selective for leftward and rightward motion, users are instructed to open the block breaker game (accessed via the Lessons tab in the menu bar). This will open the game (**Fig. 42**). Opening the game automatically turns on all the stimulus LEDs on RetINaBox.
2. The game is programmed to take the RetINaBox output from RGC1 to move the game paddle to the left, and the RetINaBox output from RGC2 to move the game paddle to the right
3. Users then sweep their hand across RetINaBox's field of view, leftward and rightward, to move the paddle and play the game.

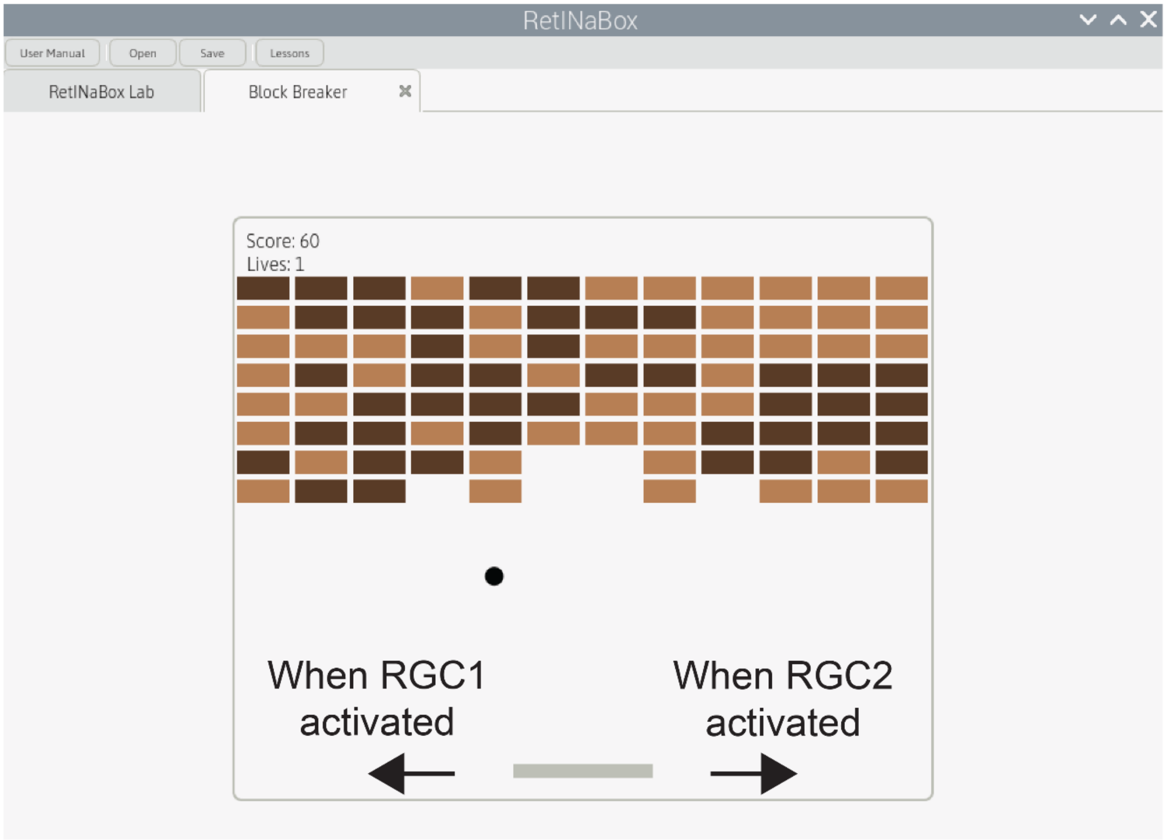

Figure 42. Brick breaker game related to Lesson 3.

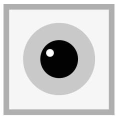

iii. Discovery Mode:

1. Lesson 4 is Discovery Mode, which can be accessed via the Lessons tab in the menu bar. It opens the Discovery Mode tab (**Fig. 43**). Opening Discovery mode automatically turns on all the stimulus LEDs on RetINaBox.
2. Next, users select a Challenge from the menu.
3. In Phase 1, users use the Visual Stimulus Tool, or their paper cutouts, or their hand, to present various static and moving stimuli to RetINaBox until they are able to activate RGC1.
4. Once users find the preferred visual stimulus for a given challenge they enter the solution into the left-hand side of the software (Phase 1) and select 'Test Stimulus.' If they are correct, they move onto Phase 2.
5. In Phase 2, users set the connectivity between model photoreceptors and the model RGC in order to account for the visual stimulus selectivity they discovered in Phase 1. Once the user is confident in their solution, they click 'Test Connectivity.'

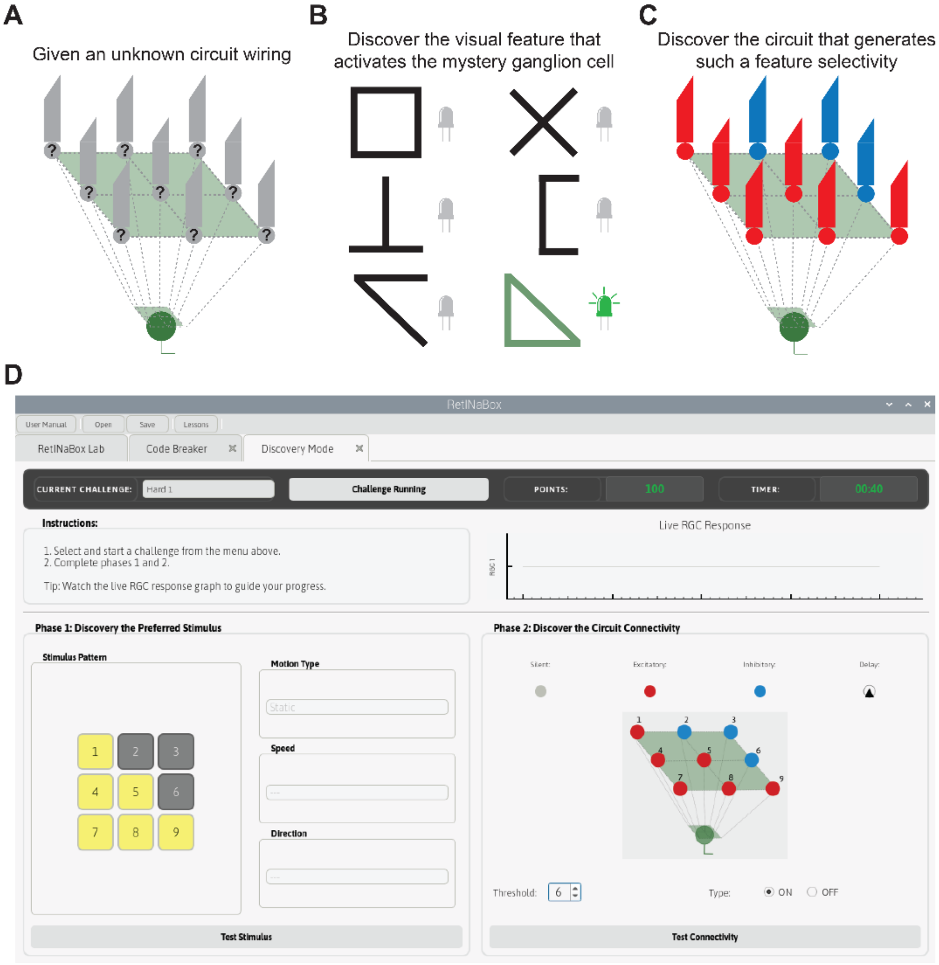

Figure 43 Discovery Mode graphical user interface.

A1- APPENDIX 1: COMPONENTS LIST

Category	#	Product	Specifications	Supplier	Notes
RetINaBox	6	Case	3D printed pieces (link)		
	26	M3 screws 6mm			
	4	M2 screws 6mm			Used to secure GPIO board to the case
	9	5mm IR LED	100 mA continuous, 1000 mA pulse Approx 1.6V forward voltage	Adafruit 387	
	9	IR Photodiodes		Amazon	
	1	Elegoo electronics kit	Things you'll need from here: 9 white LEDs, 1 green LED, 1 yellow LED, resistors	Amazon	
	12	Mini Breadboard		Amazon	
	26	Dupont Wires		Amazon	
		Jumper wires kit		Amazon	
	1	GPIO Breakout Kit for Raspberry Pi		Amazon	
	1	Rainbow connection extender	Optional – adding an extra cable or two lets you orient RetINaBox more freely next to the Raspberry Pi	Amazon	
	1	elechawk PH 2.0 Connector Pre-Crimped Cable Kit		Amazon	
	1	Roll of electrical tape			

Category	#	Product	Specifications	Supplier	Notes
	1	Red electrical hook-up wire		Digikey	If you have some lying around, great. If not, order something like this, but it need not be exactly this product
	1	Black electrical hook-up wire		Digikey	Same note as above
For testing IR LEDs	1	3.3V USB to Serial Adapter	Optional	Amazon	You can use this to power an IR photodiode and test the IR LEDs independently of the rest of RetINaBox
Visual Stimulus Tool	Set of 2	Clear plastic sheet (15.4x 22.4cm)		Amazon	Put the playdough on this and then stick it in between RetINaBox's IR LEDs and photodiodes
	1	Thin-tipped sharpie	Any thin tipped permanent marker should do		To draw a grid on the Visual Stimulus Tool to guide where to place the modeling clay
	1	Modeling clay	Any brand should do, including play dough		You need enough to thinly cover the Visual Stimulus Tool
Tools	1	2.5mm hex key			Used with M3 screws
	1	1.5mm hex key			Used with M2 screws
	1	Wire stripper/cutter			
	1	Mini screwdriver			For adjusting the photodiode sensitivity

A2- APPENDIX 2: TROUBLESHOOTING

<u>3D printing related</u>:

a. The photodiodes and LEDs don't fit properly fit into holes in the 3D printed parts meant for them to pass through
    i. We recommend printing parts P2, P3 or P5 first, and checking that the LEDs/photodiodes fit snugly into the holes. If not, the pieces should be scaled a couple % in size up or down based on how your printer generates these pieces. Then, all other printed parts should be scaled by the same factor.

<u>Photodiode related</u>:

a. The photodiode power light (right-most LED on the circuit board) does not turn on when RetINaBox is connected to the Raspberry Pi and the Raspberry Pi is turned on.
    i. Check the wiring between the RetINaBox GPIO connector and the photodiode. A multimeter can be handy here.
    ii. Check that you've properly connected the rainbow connection cable.
    iii. You can check that the Raspberry Pi is correctly sending out power to the photodiodes. First, disconnect the rainbow connection cable. Next, connect a spare visible light LED onto a spare mini breadboard. Take a female-to-male dupont cable and connect GPIO pin 17 (which normally provides 3.3V to power the photodiodes) and connect it to the LED's (+) lead. Next, take another female-to-male dupont cable and connect GPIO pin 39 (which normally provides the (-)/GND to the photodiodes) to the LED's (-) lead. If the LED lights up when you turn it on with the RetINaBox GUI, then the Raspberry Pi is sending out power, and the issue is with your wiring. If this doesn't work, you may have somehow burned this pin in your Raspberry Pi.
b. The photodiode is powered, but it doesn't get activated (i.e. its leftmost red LED on its circuit board does not turn on) when the IR LEDs are turned on, or it doesn't get deactivated (i.e. its leftmost red LED on its circuit board does not turn off) when the IR LEDs are turned off.
    i. The photodiode's sensitivity is not properly adjusted. Remove part P6. Using the RetINaBox software, turn on the LED directly above the photodiode you're having an issue with. Adjust that photodiode's potentiometer (see **Fig. 34**) with a screwdriver until the leftmost red LED on

its circuit board turns ON when the LED is ON, and also turns OFF when the LED is turned OFF.

ii. If step 1 doesn't work, there are two other possible issues. First, it could be that the IR LED is not wired correctly. To check this, go to the section below titled "LED related." Second, it could be that the photodiode and its correspond LED are not properly aligned in the 3D printed case. To the best of your ability, each photodiode should point directly at its corresponding IR LED, and each IR LED should point directly at its corresponding photodiode (the LEDs have relatively narrow angle output, and the photodiodes are most sensitive to light pointing directly at them.

LED related:

a. One of the white LEDs does not turn on when it's activated in the software
    i. Check your wiring (and the polarity of your LED)!
    ii. Try replacing the LED.
    iii. Similar to step a-iii in the "Photodiode related" section above, you can directly check that the Raspberry Pi is sending the correct signal. Check with **Figs 12** and **22** to check which GPIO pins correspond to the LED that is not working correctly. Take a female-to-male dupont cable and connect the GPIO pin that provides power to the problematic LED (which normally provides 3.3V to power that LED when the software activates it) and connect it to the breadboard LED's (+) lead. Next, take another female-to-male dupont cable and connect GPIO pin 9 (which normally provides the (-)/GND to the photodiodes) to the breadboard LED's (-) lead. If the LED lights up when you activate this LED in the RetINaBox GUI then the Raspberry Pi is sending out power appropriately, and the issue is with your wiring. If this doesn't work, you may have somehow burned this pin in your Raspberry Pi.
b. One of the IR LEDs does not turn on when it's activated in the software
    i. Check your wiring (and the polarity of your LED)!
    ii. Try replacing the LED.
    iii. Follow the same instructions as outlined in the "LED related" section a-iii. Since you cannot see whether the IR LED is active with your eye, you can either use the corresponding photodiode in RetINaBox as your readout, or you can connect the USB to TTL serial adapter (see recommend components list) to a spare photodiode, and use this photodiode to test for activation of the IR LED.
c. One of the color LEDs (green or yellow) does not turn on when the software is running and the corresponding retinal ganglion cell is activated in the GUI's Signal Monitor.

i. Check your wiring (and the polarity of your LED)!
ii. Try replacing the LED.
iii. Follow the same instructions as outlined in the “LED related” section a-iii, but for the GPIO pins powering these two LEDs (**Figs. 12** and **22**).

d. For a connected pair of white and IR LEDs, only one of them is active (i.e. they are getting powered, but only one of them is active).
   i. Check your wiring (and the polarity of your LED)!
   ii. Try replacing the LED that isn’t working.
   iii. Make sure you have the correct resistors in place (this is important, since the different types of LEDs have different forward voltage (and likely different internal resistance as well), so to ensure both LEDs are activated by the same power source we need to use resistors to standardize current flow through both IR and white LEDs).
e. LEDs are flickering
   i. Check your wiring - most likely it’s a loose connection.

Software installation related:

a. The code does not run when the RetINaBox app is clicked and ‘Execute’ is selected. See troubleshooting suggestions here.

A3: Appendix 3: Costs

Part		Price (CAD)	Price (USD)
3D printed pieces (cost estimate)	piece 1 (130g)	4.03	2.86
	piece 2 (136g)	4.21	2.99
	piece 3 (80g)	2.48	1.76
	piece 4 (38g)	1.18	0.83
	piece 5 (48g)	1.49	1.06
	piece 6 (41g)	1.27	0.90
GPIO Breakout Kit for Raspberry Pi		18.00	12.78
Jumper wires kit		18.99	13.48
Dupont Wires		12.99	9.22
Mini Breadboards		12.99	9.22
Photodiodes		15.99	11.35
Raspberry Pi 500		169.95	120.66
Screen YODOIT		89.99	63.89
M3 screws		11.65	8.27
M2 screws		18.99	13.48
ELEGOO kit		21.99	15.61
Pre-Crimped Cable Kit Compatible		20.99	14.90
IR LEDs		8.40	5.96
Plastic sheet		15.99	11.35
Play doh		3.00	2.13
3.3V USB to TTL Serial Adapter		15.99	11.35
**Total**		**470.55**	**334.096**
